# Supplementary material for: Circular van Krevelen diagram for visualizing metabolic pathways
Source: bioRxiv. 2025 Jun 3:2025.05.31.657198. Preprint. [Version 1] doi: 10.1101/2025.05.31.657198 (PMC12157561; doi:10.1101/2025.05.31.657198)

Steroid metabolism

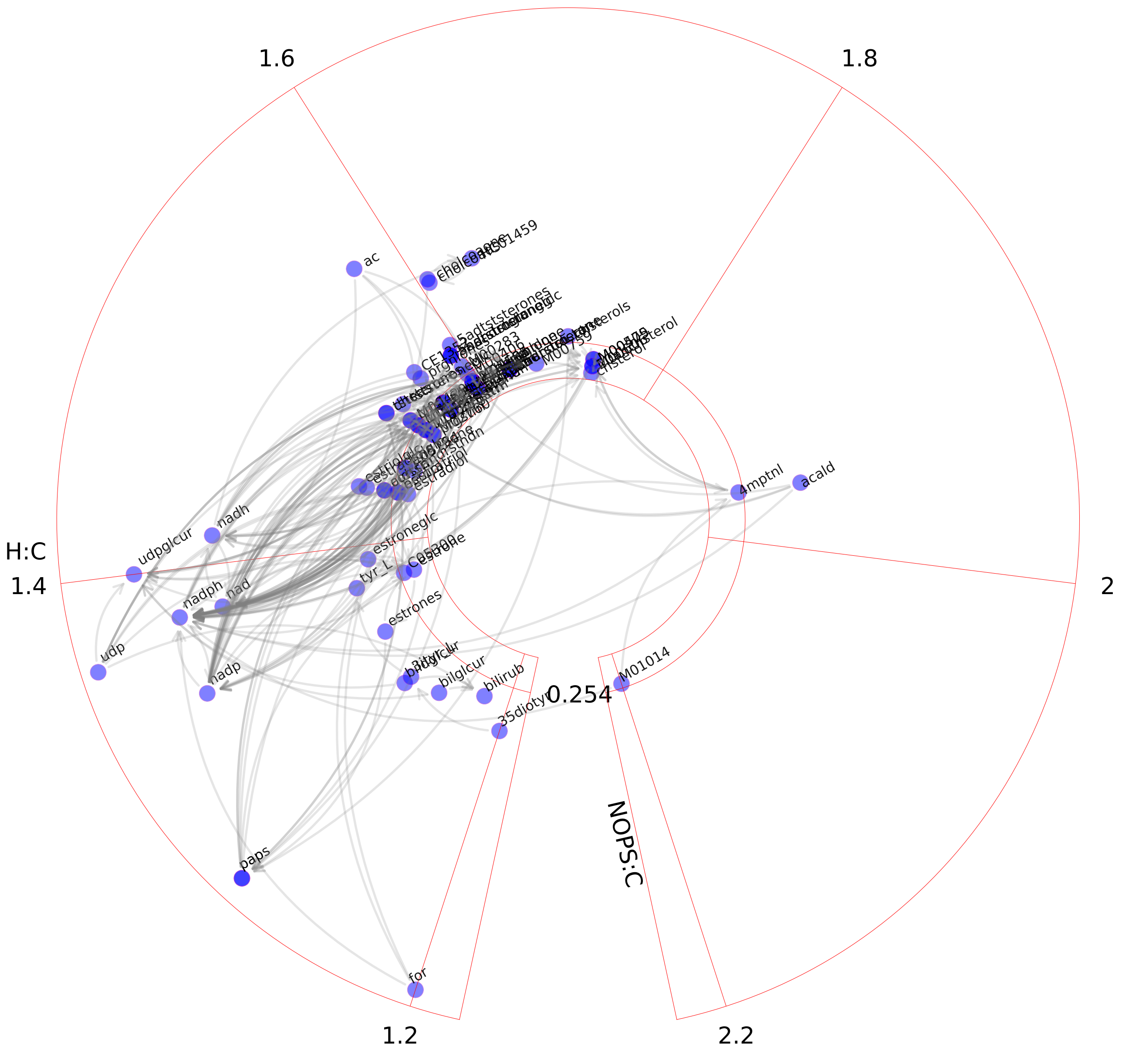

Supplement: Supplement 1 [file media-1.zip › Suppl_File_all_pathways/labeled/Steroid metabolism.pdf]
