# Supplementary figures and images for: Circular van Krevelen diagram for visualizing metabolic pathways (part 2 of 2)
Source: bioRxiv. 2025 Jun 3:2025.05.31.657198. Preprint. [Version 1] doi: 10.1101/2025.05.31.657198 (PMC12157561; doi:10.1101/2025.05.31.657198)

## 1.2

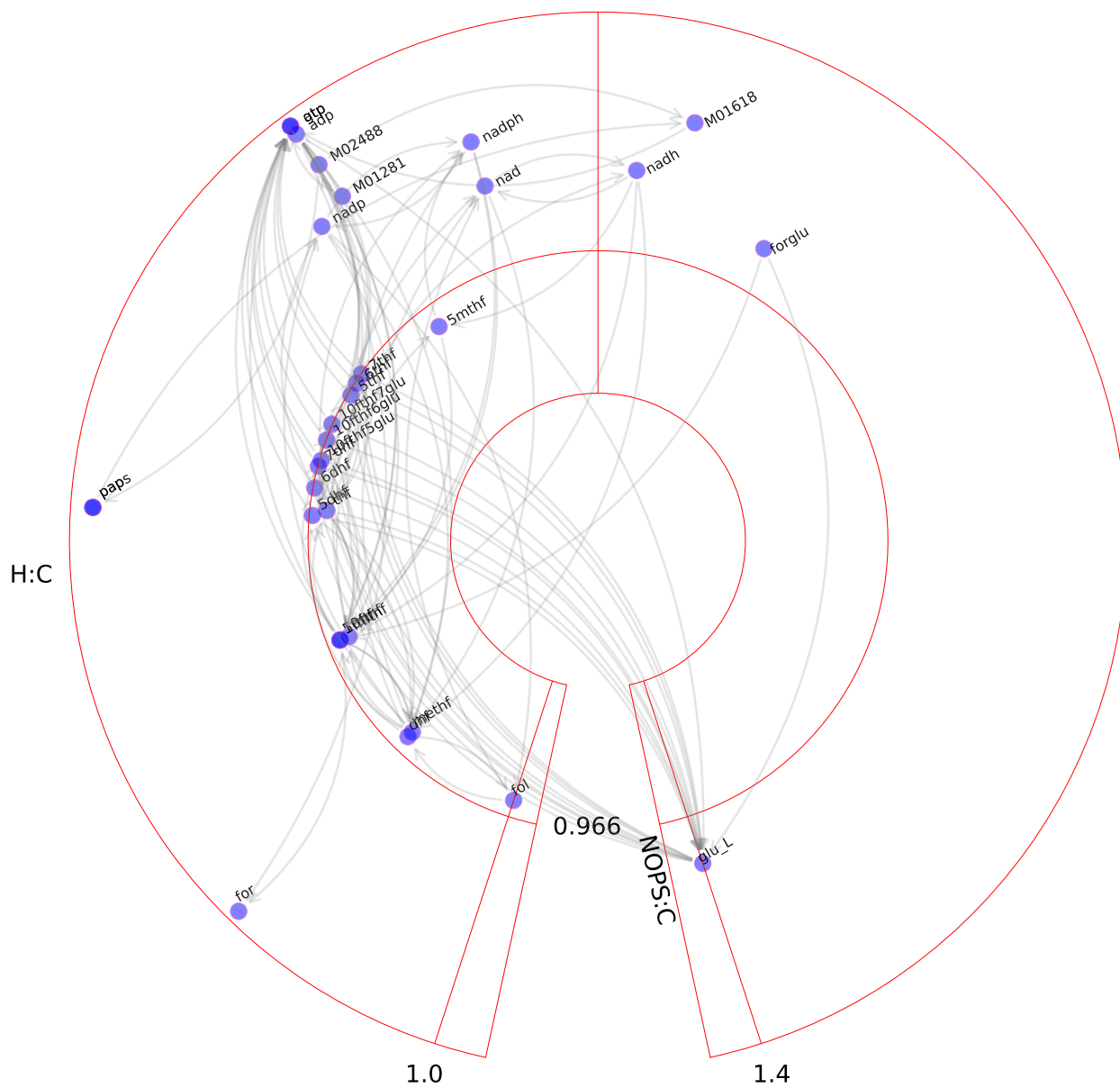

Supplement: Supplement 1 [file media-1.zip › Suppl_File_all_pathways/labeled/Folate metabolism.pdf]

# Glycerophospholipid metabolism

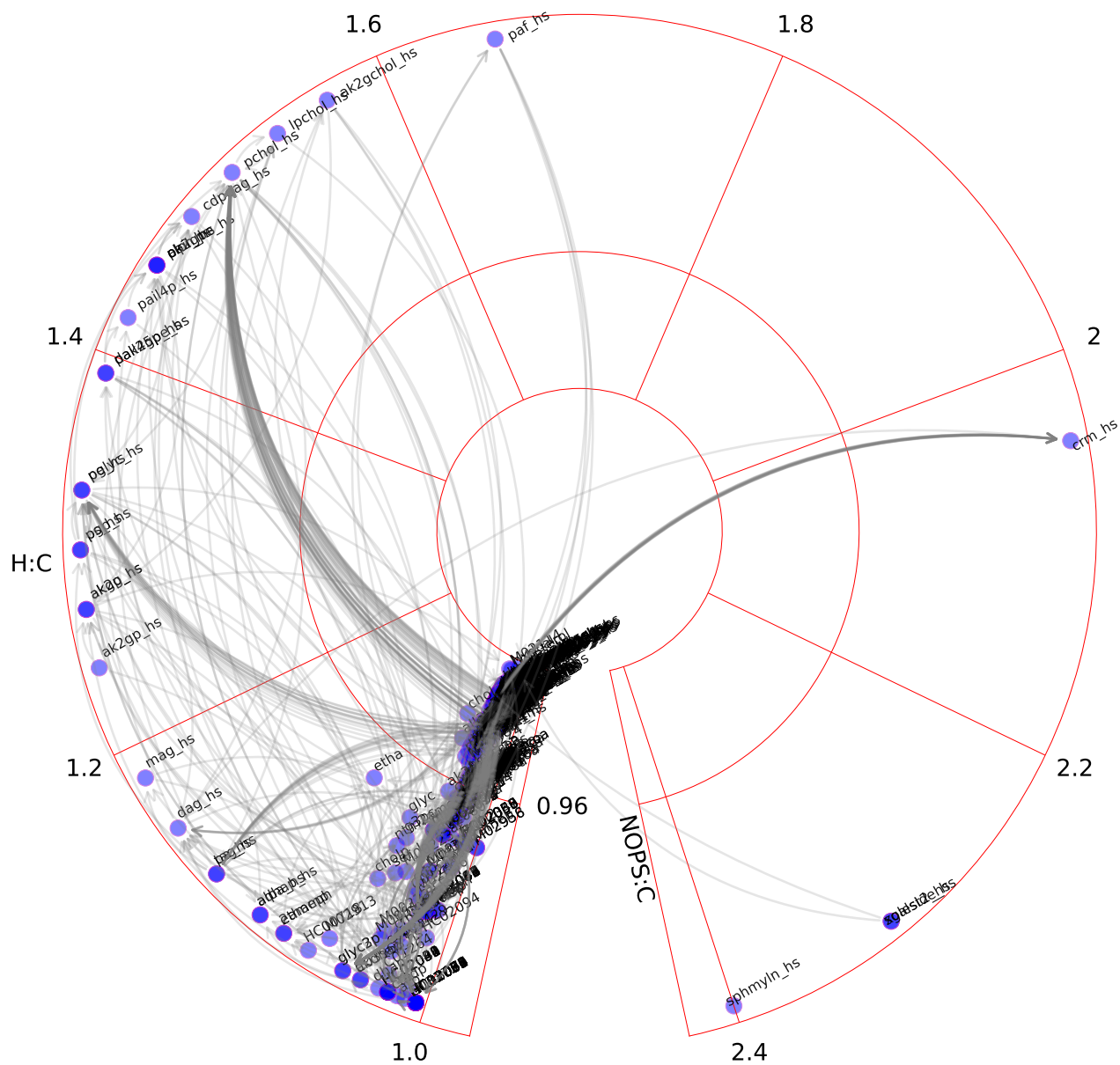

Supplement: Supplement 1 [file media-1.zip › Suppl_File_all_pathways/labeled/Glycerophospholipid metabolism.pdf]

## 1.8

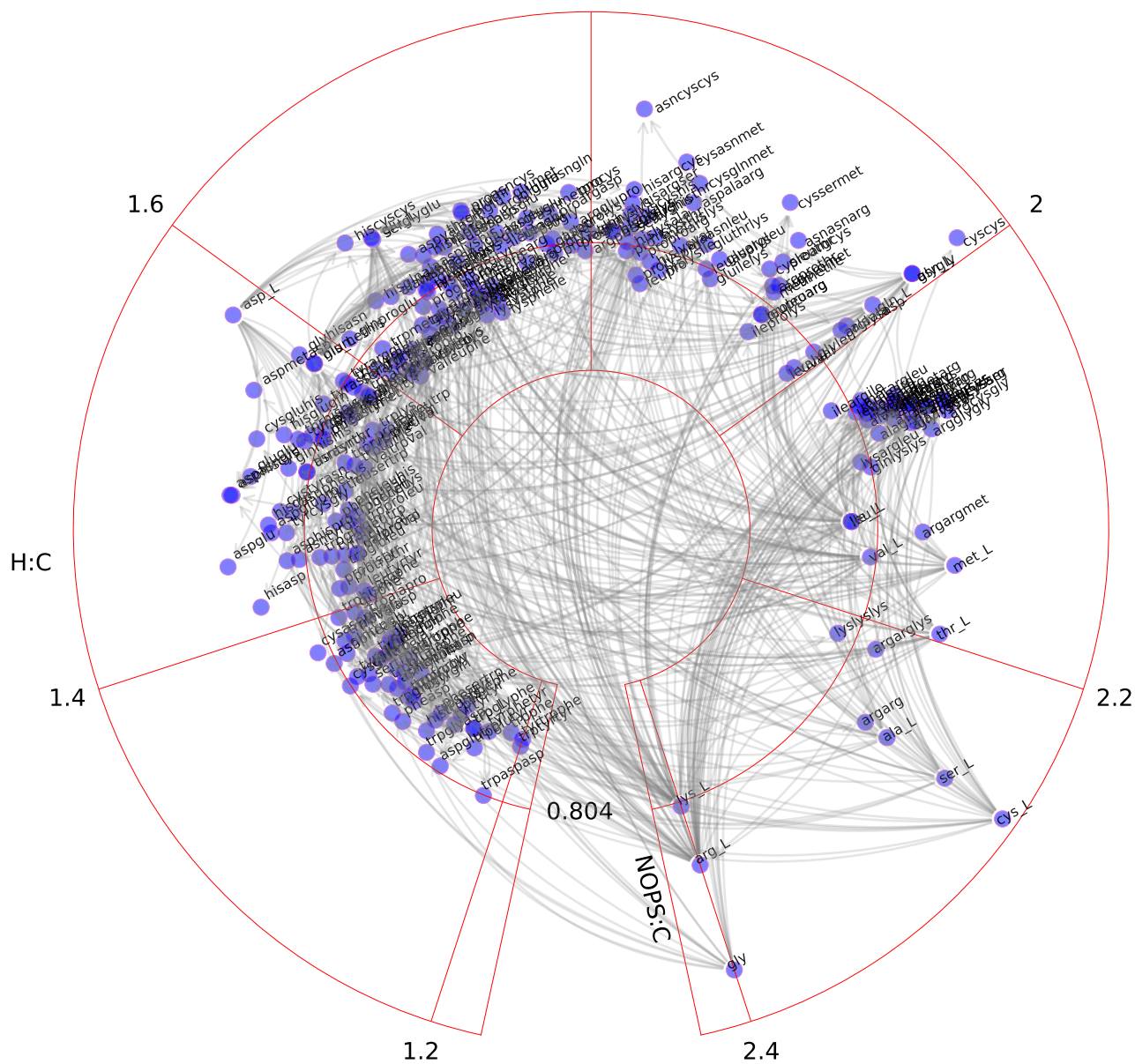

Supplement: Supplement 1 [file media-1.zip › Suppl_File_all_pathways/labeled/Peptide metabolism.pdf]

# Biotin metabolism

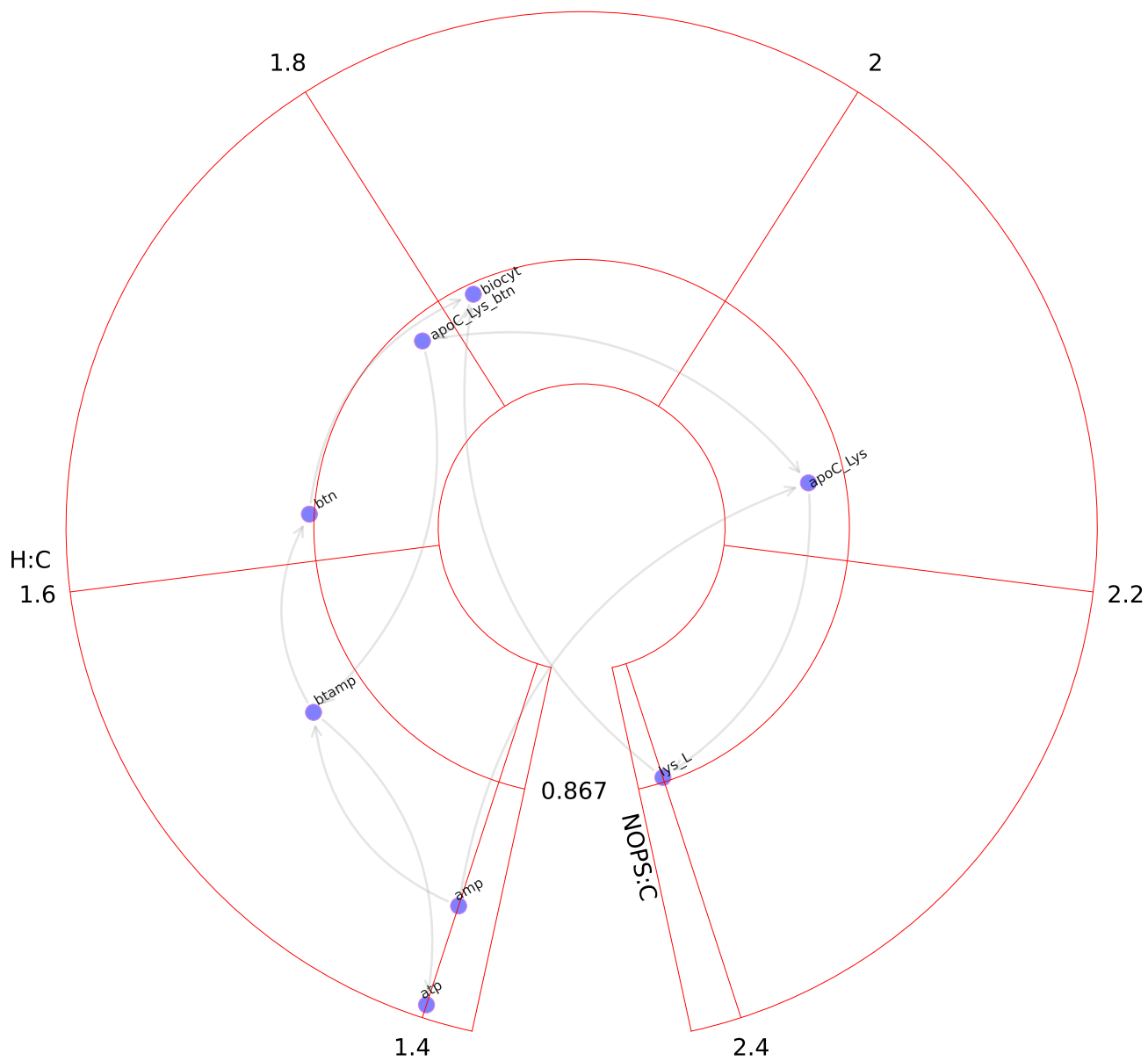

Supplement: Supplement 1 [file media-1.zip › Suppl_File_all_pathways/labeled/Biotin metabolism.pdf]

## Inositol phosphate metabolism

1.8

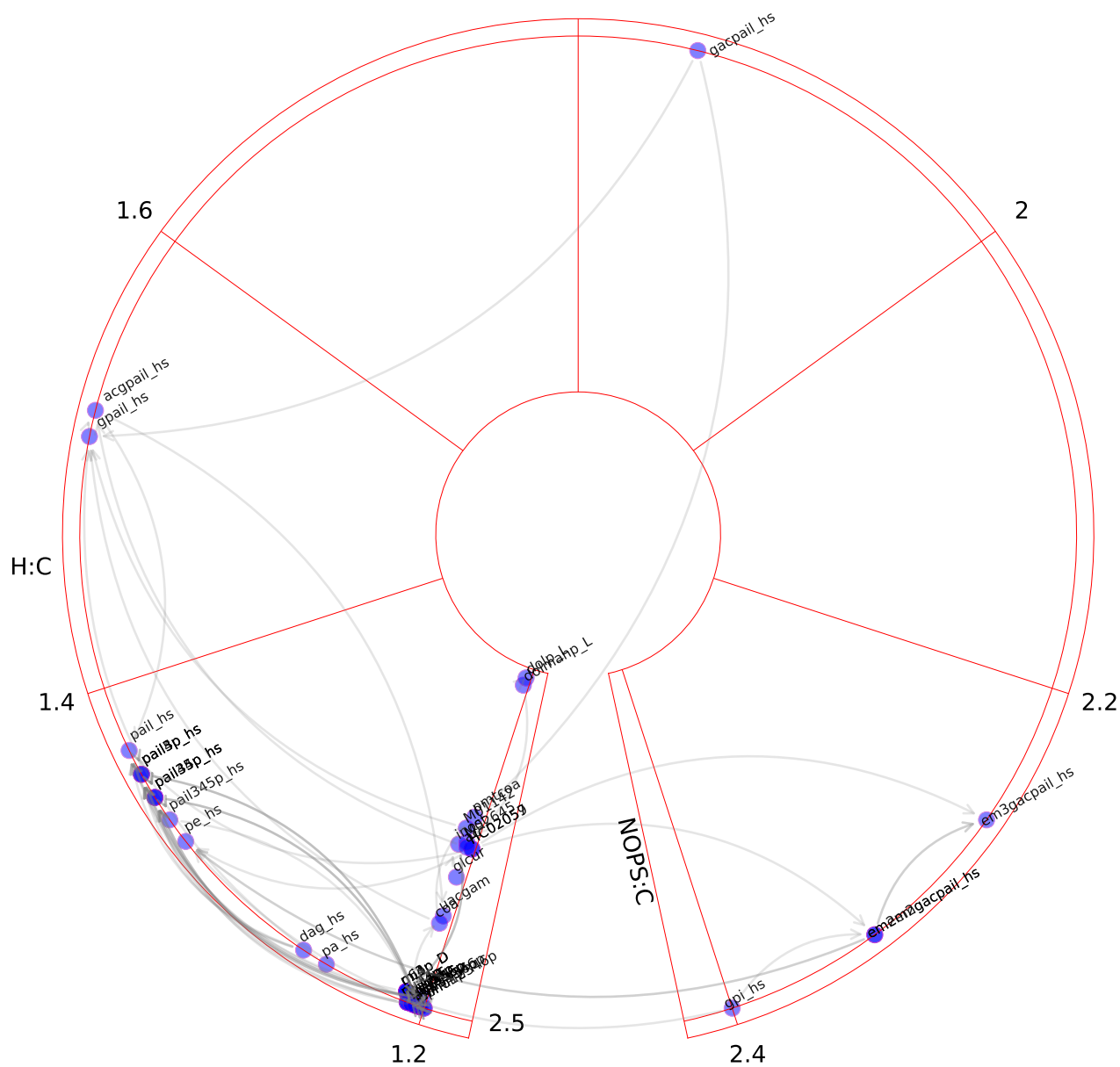

Supplement: Supplement 1 [file media-1.zip › Suppl_File_all_pathways/labeled/Inositol phosphate metabolism.pdf]

## Arachidonic acid metabolism

1.8

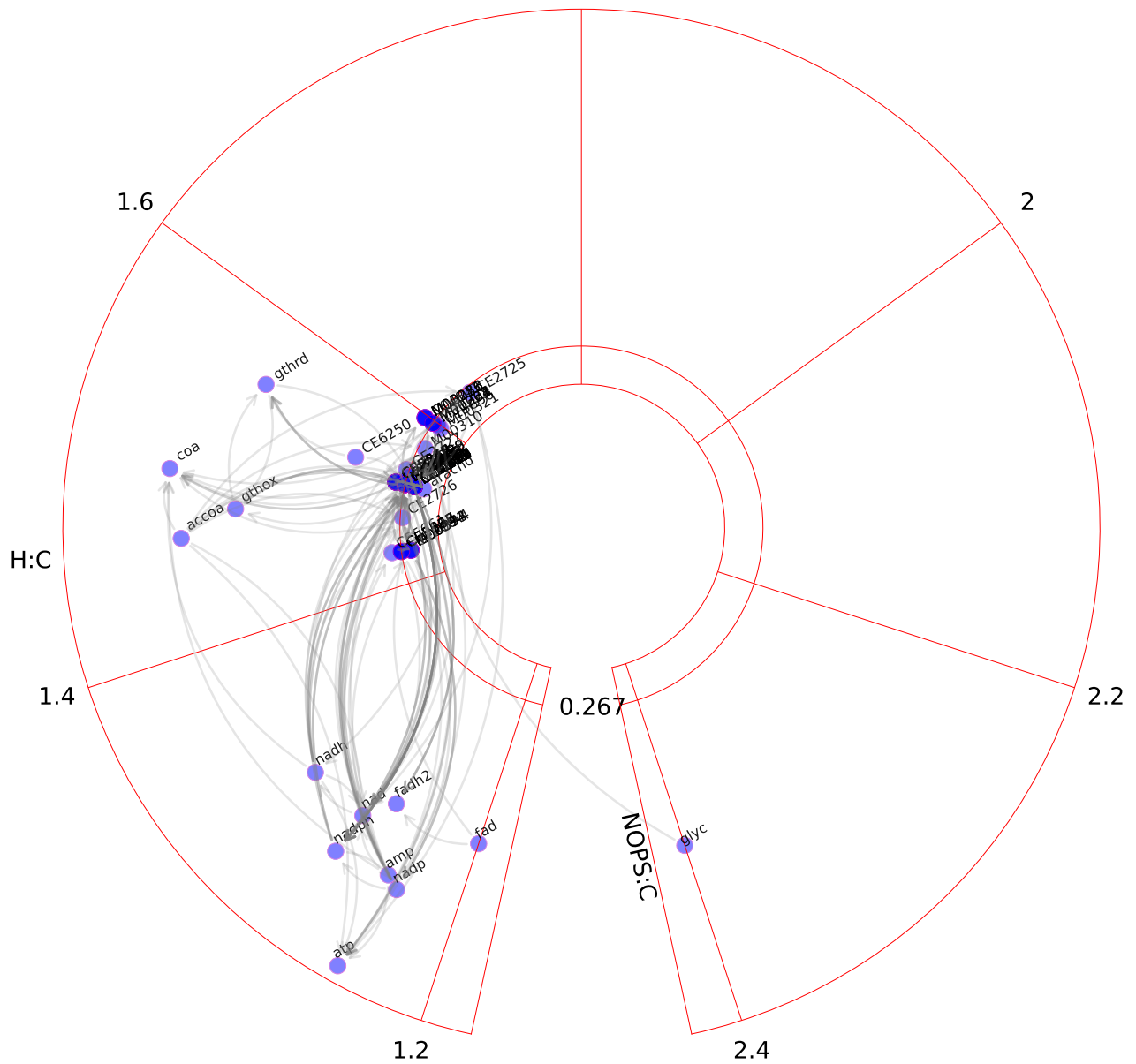

Supplement: Supplement 1 [file media-1.zip › Suppl_File_all_pathways/labeled/Arachidonic acid metabolism.pdf]

## Cytochrome metabolism

## 1.6

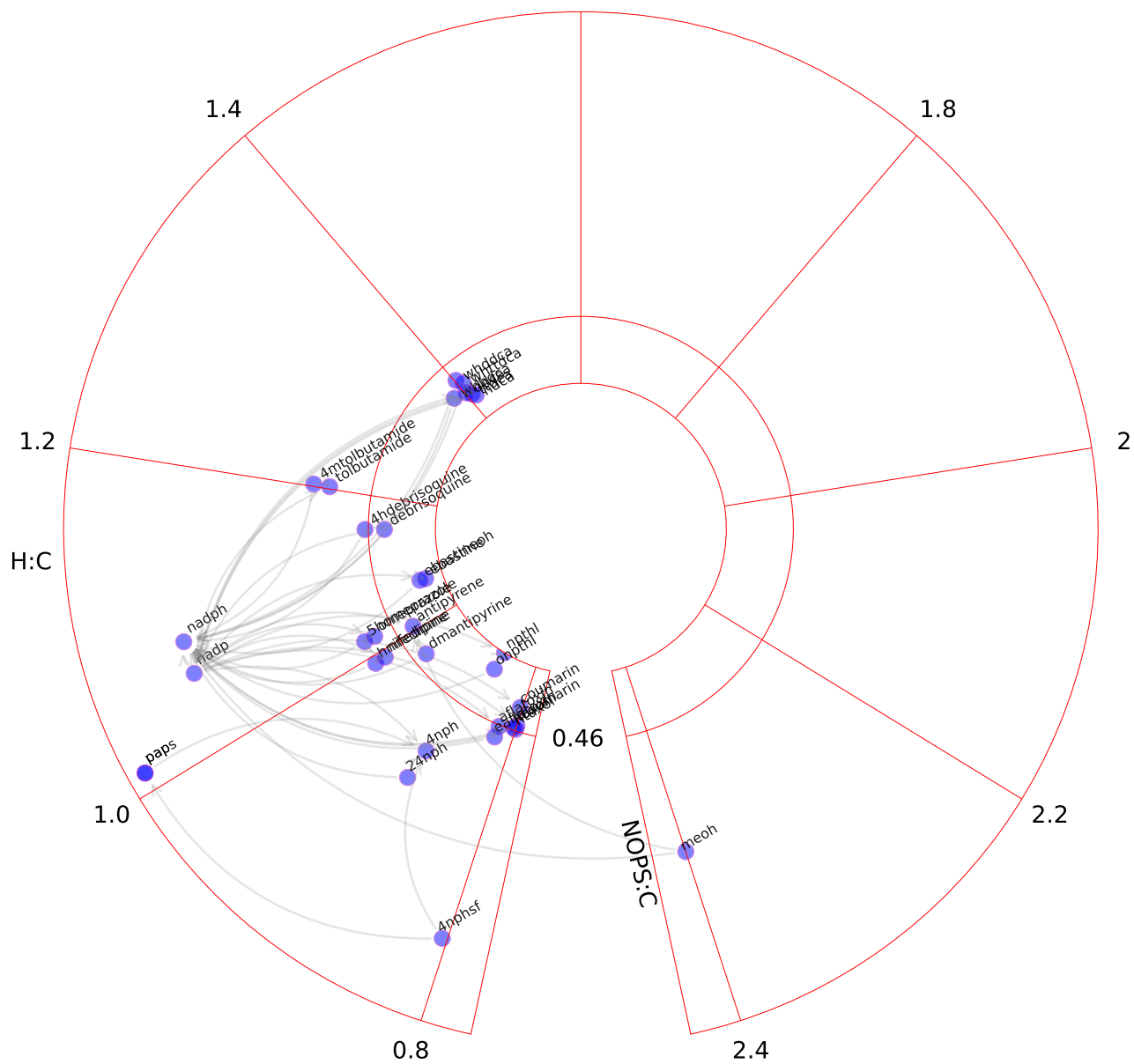

Supplement: Supplement 1 [file media-1.zip › Suppl_File_all_pathways/labeled/Cytochrome metabolism.pdf]

## Selenoamino acid metabolism

1.8

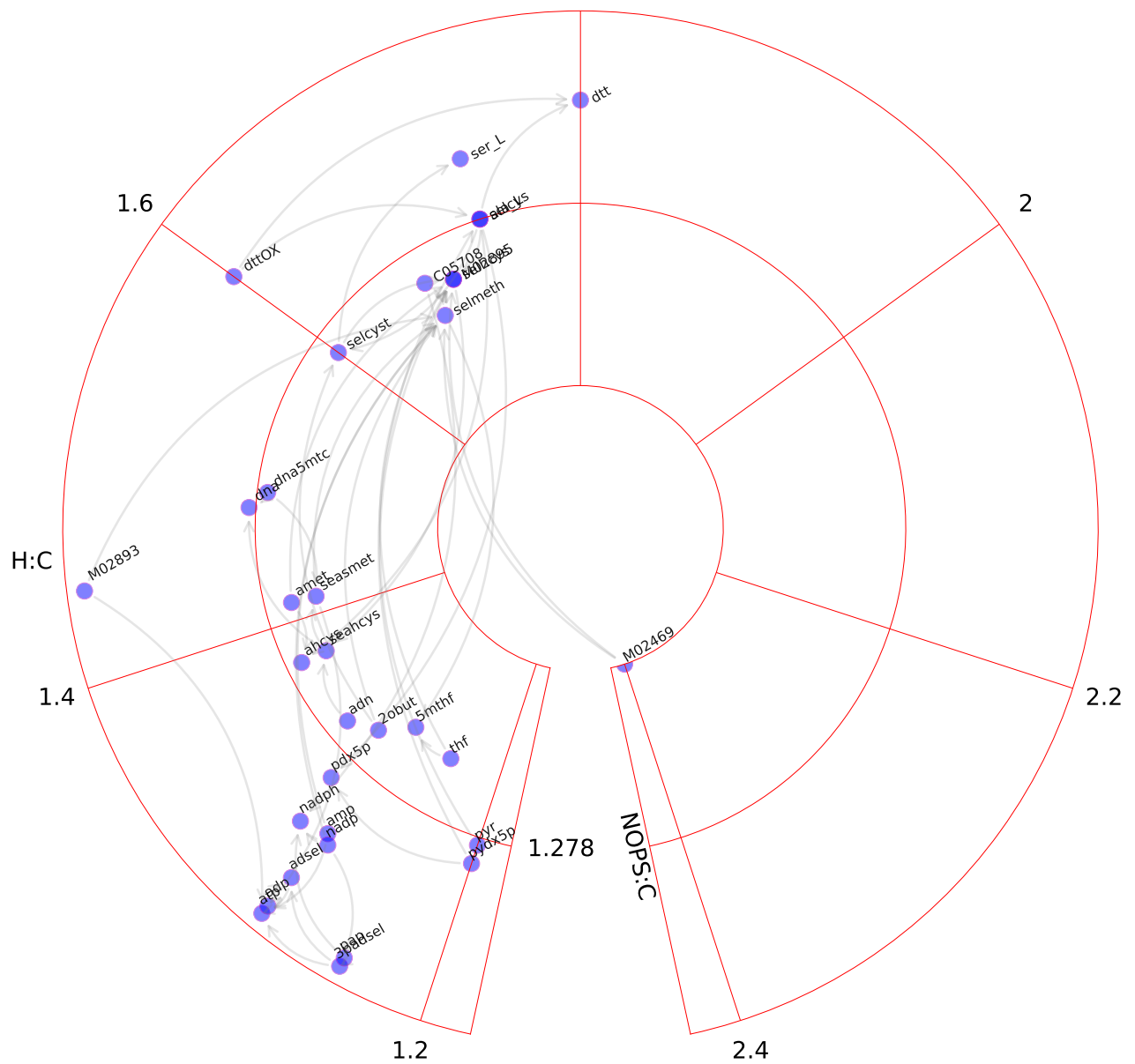

Supplement: Supplement 1 [file media-1.zip › Suppl_File_all_pathways/labeled/Selenoamino acid metabolism.pdf]

## 1.6

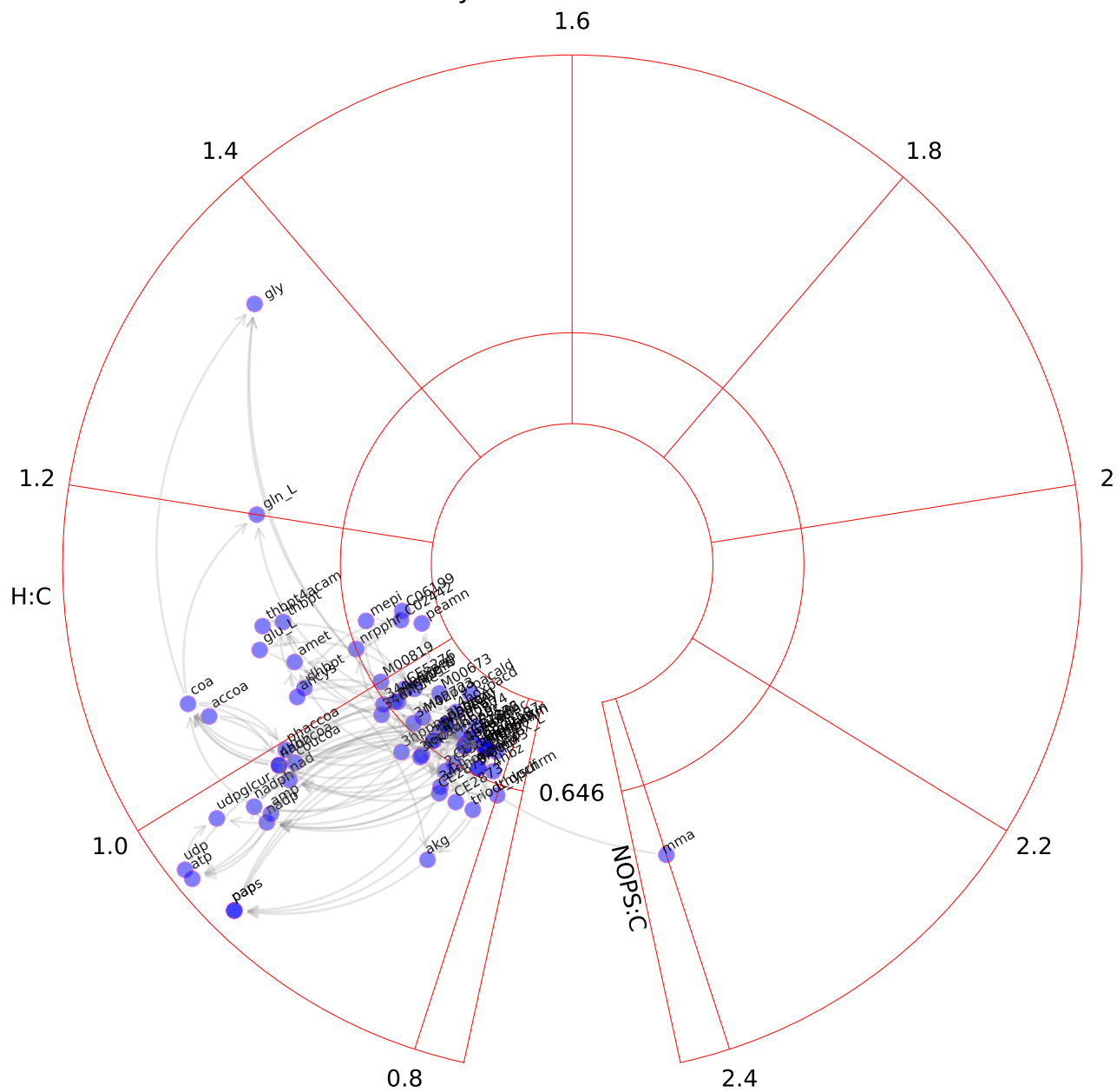

Supplement: Supplement 1 [file media-1.zip › Suppl_File_all_pathways/labeled/Phenylalanine metabolism.pdf]

# Pyruvate metabolism

1.6

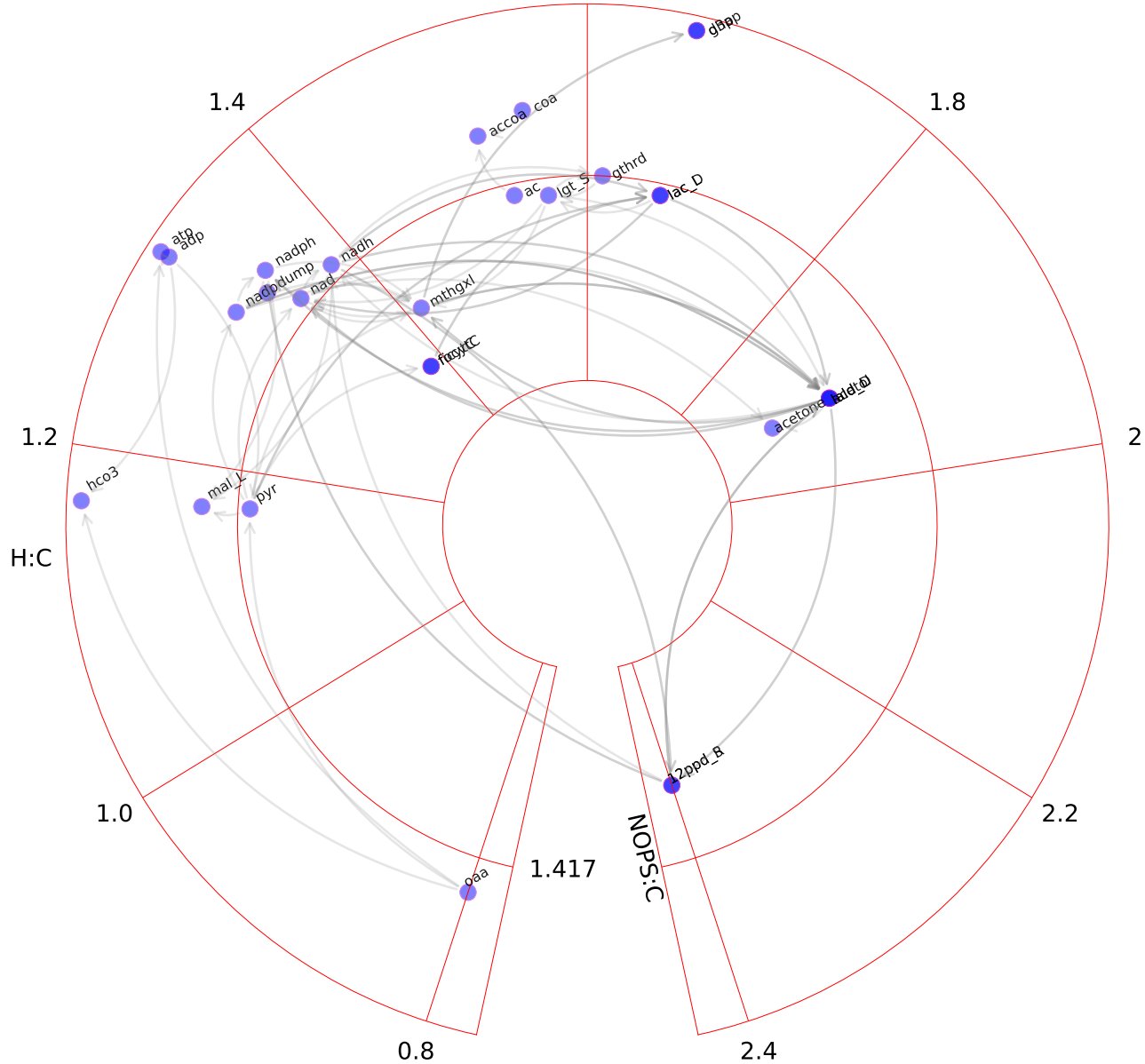

Supplement: Supplement 1 [file media-1.zip › Suppl_File_all_pathways/labeled/Pyruvate metabolism.pdf]

# Glyoxylate and dicarboxylate metabolism

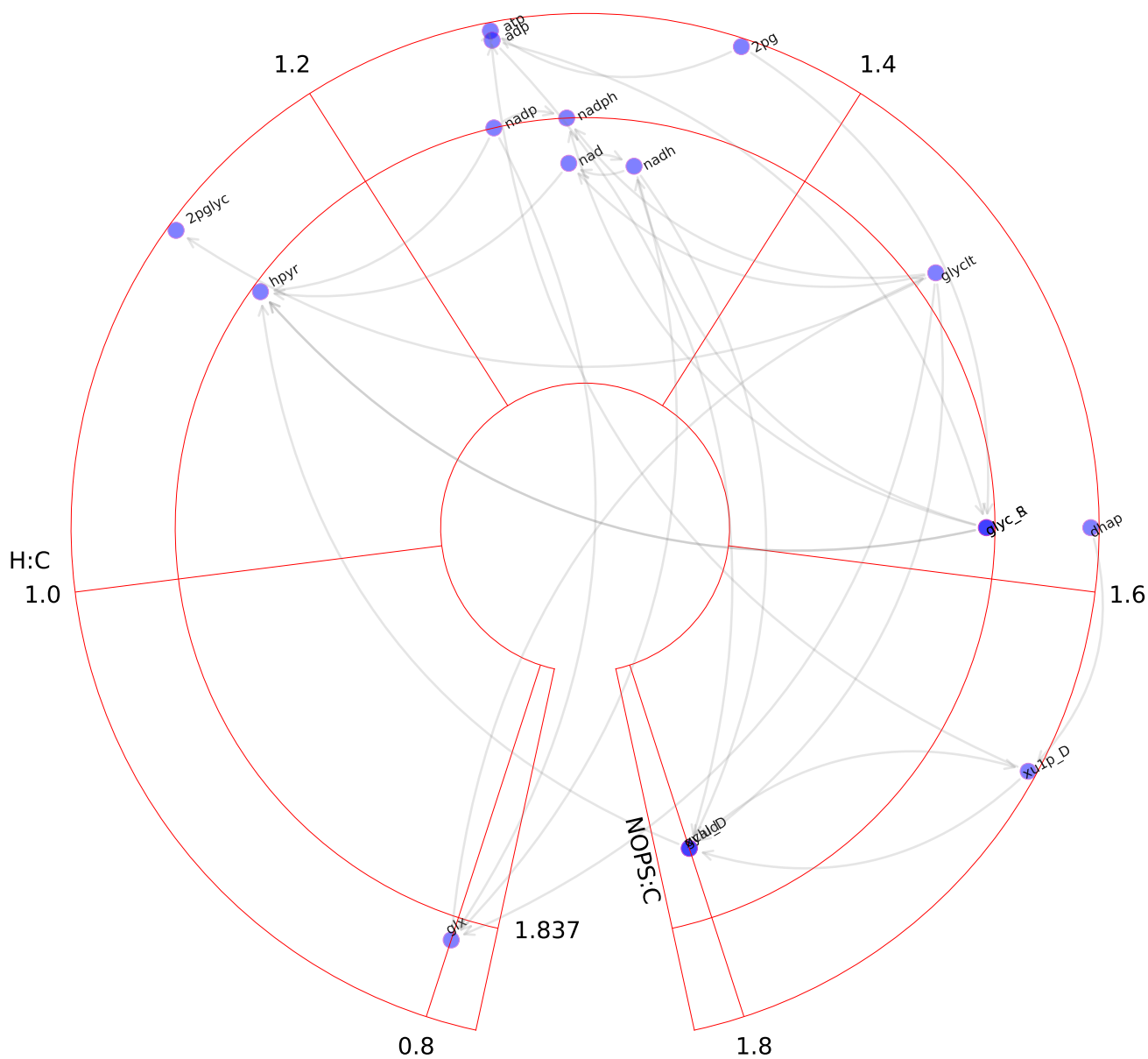

Supplement: Supplement 1 [file media-1.zip › Suppl_File_all_pathways/labeled/Glyoxylate and dicarboxylate metabolism.pdf]

## Hippurate metabolism

1.6

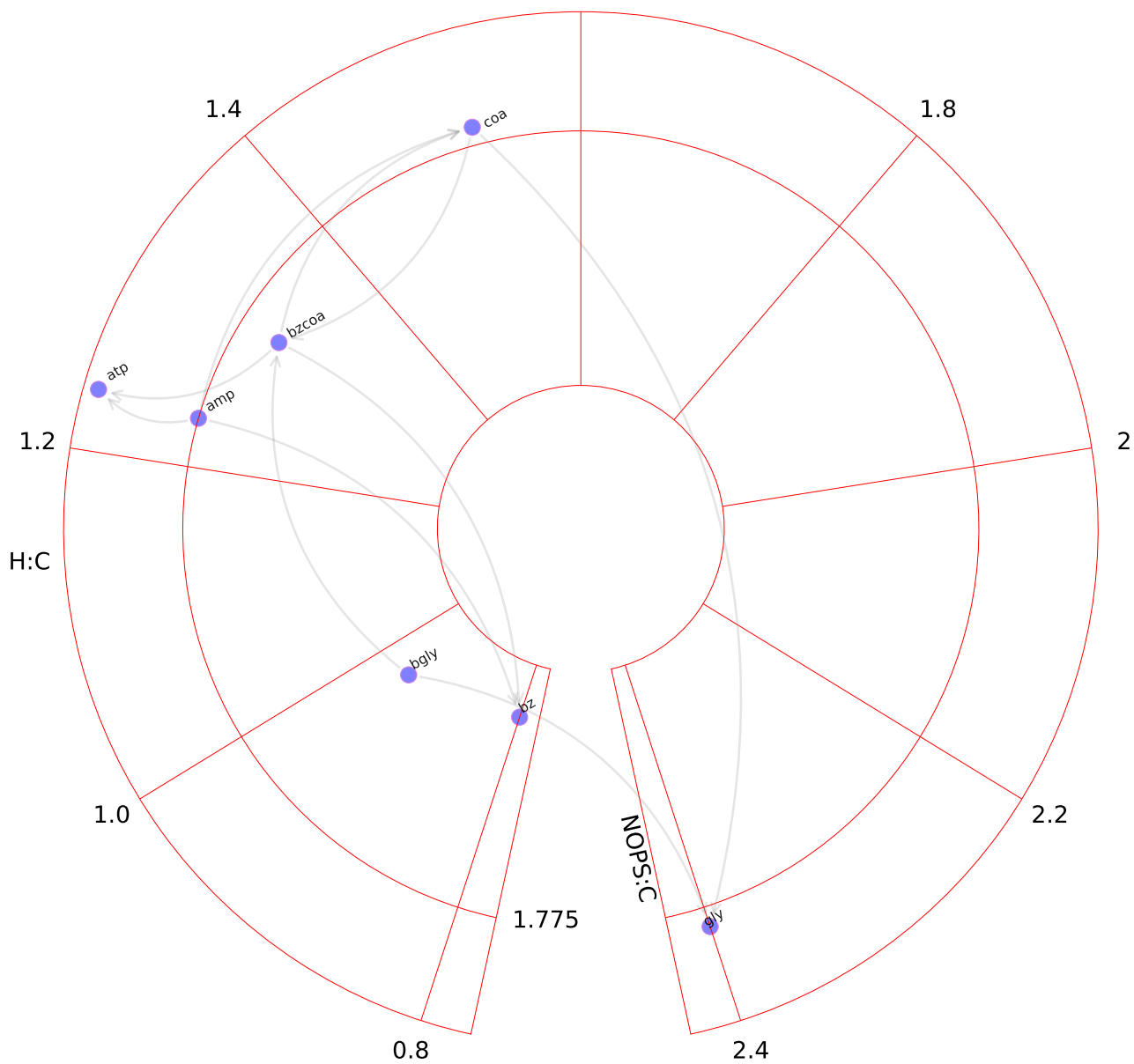

Supplement: Supplement 1 [file media-1.zip › Suppl_File_all_pathways/labeled/Hippurate metabolism.pdf]

## N-glycan synthesis

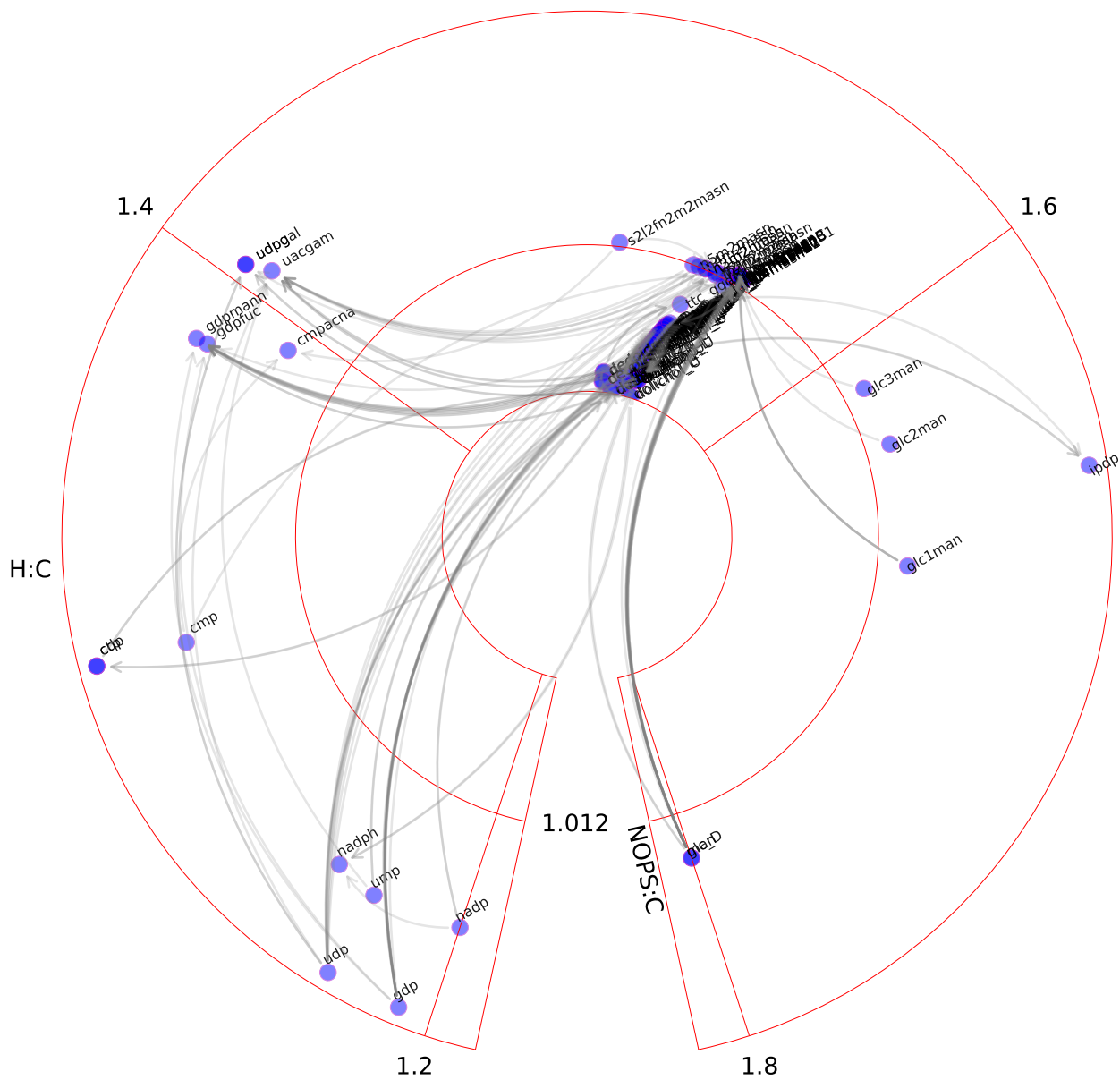

Supplement: Supplement 1 [file media-1.zip › Suppl_File_all_pathways/labeled/N-glycan synthesis.pdf]

## Vitamin A metabolism

1.8

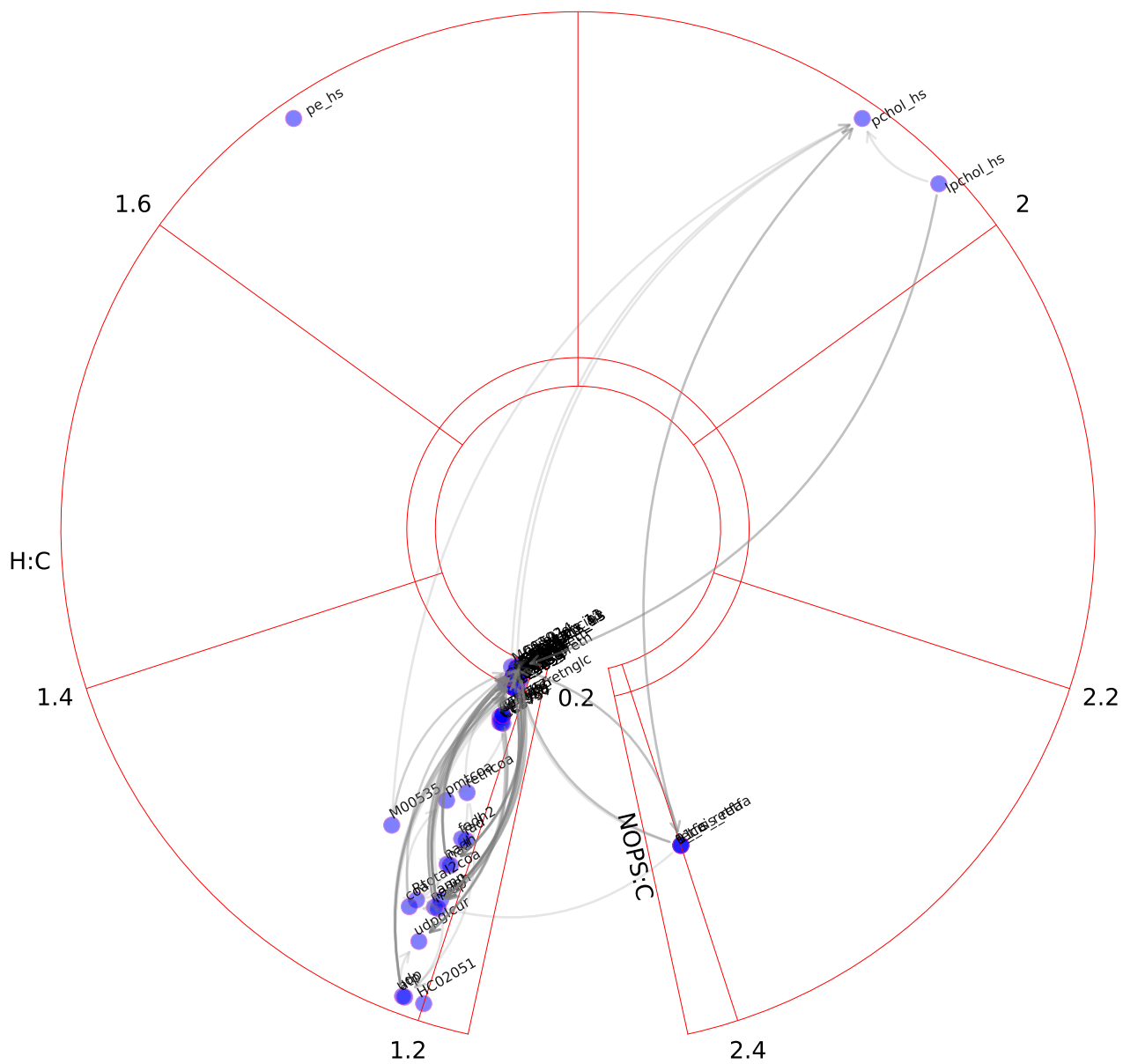

Supplement: Supplement 1 [file media-1.zip › Suppl_File_all_pathways/labeled/Vitamin A metabolism.pdf]

# Butanoate metabolism

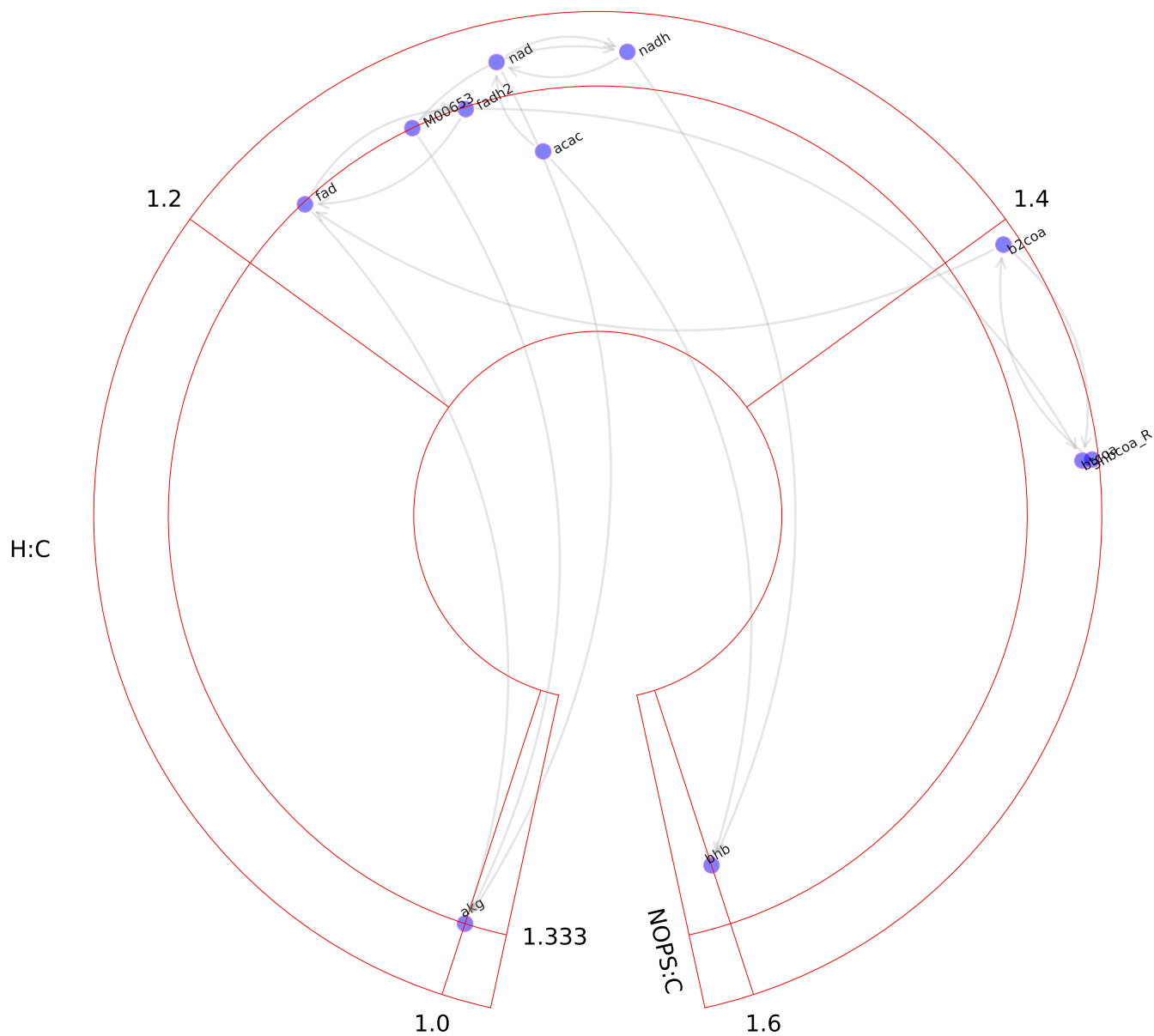

Supplement: Supplement 1 [file media-1.zip › Suppl_File_all_pathways/labeled/Butanoate metabolism.pdf]

## 1.6

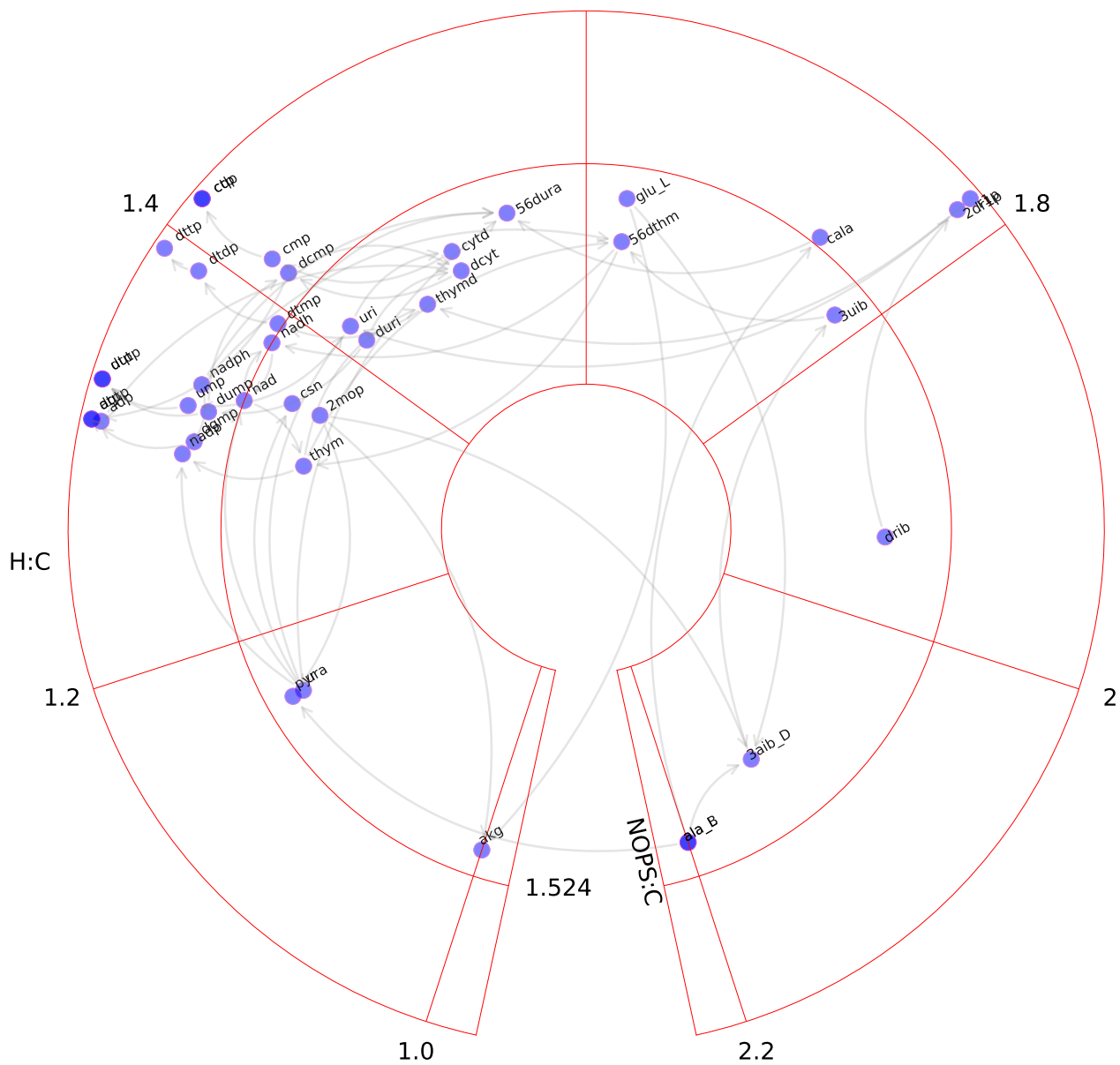

Supplement: Supplement 1 [file media-1.zip › Suppl_File_all_pathways/labeled/Pyrimidine catabolism.pdf]

## Chondroitin synthesis

1.4

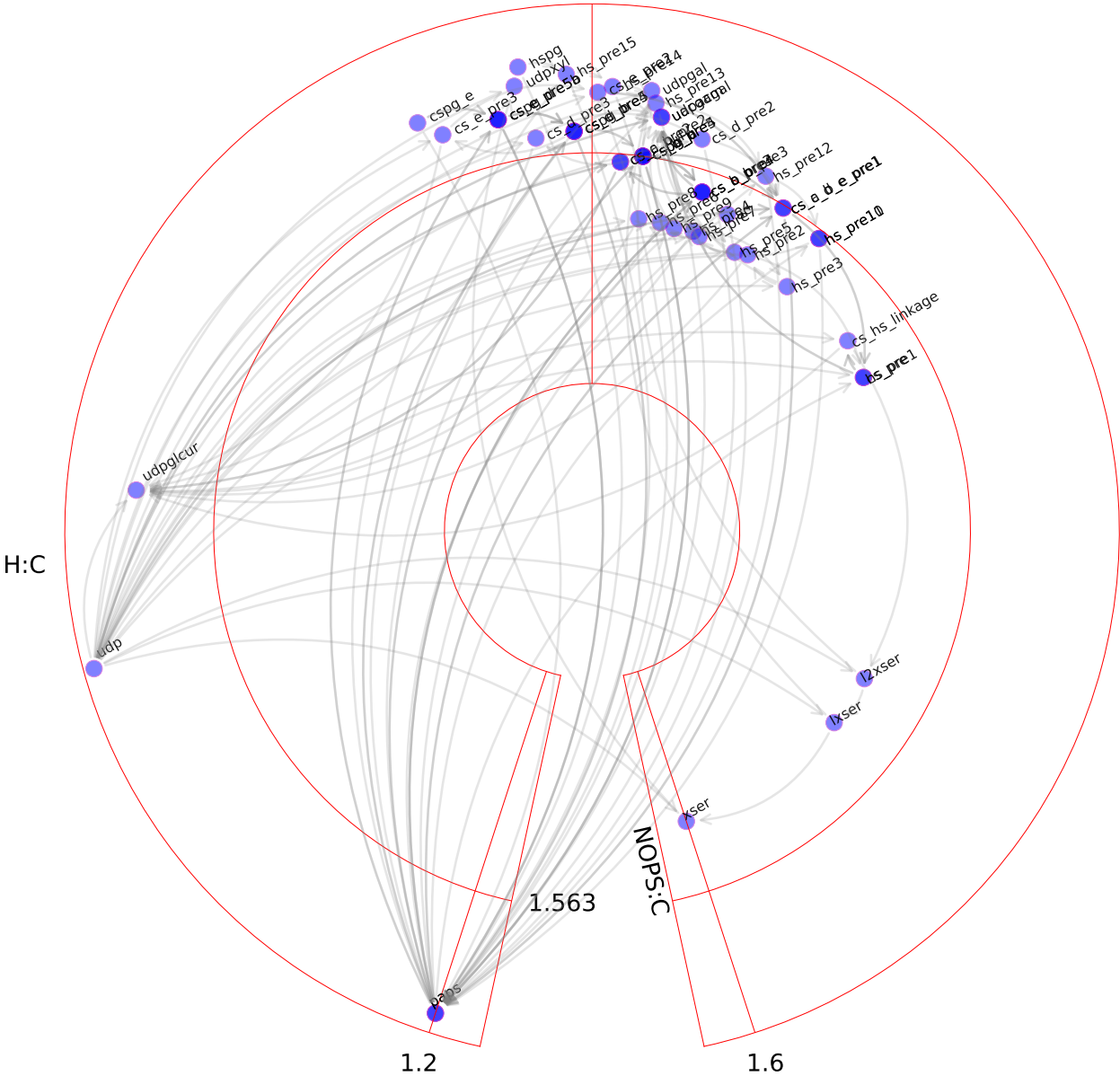

Supplement: Supplement 1 [file media-1.zip › Suppl_File_all_pathways/labeled/Chondroitin synthesis.pdf]

# Fatty acid oxidation

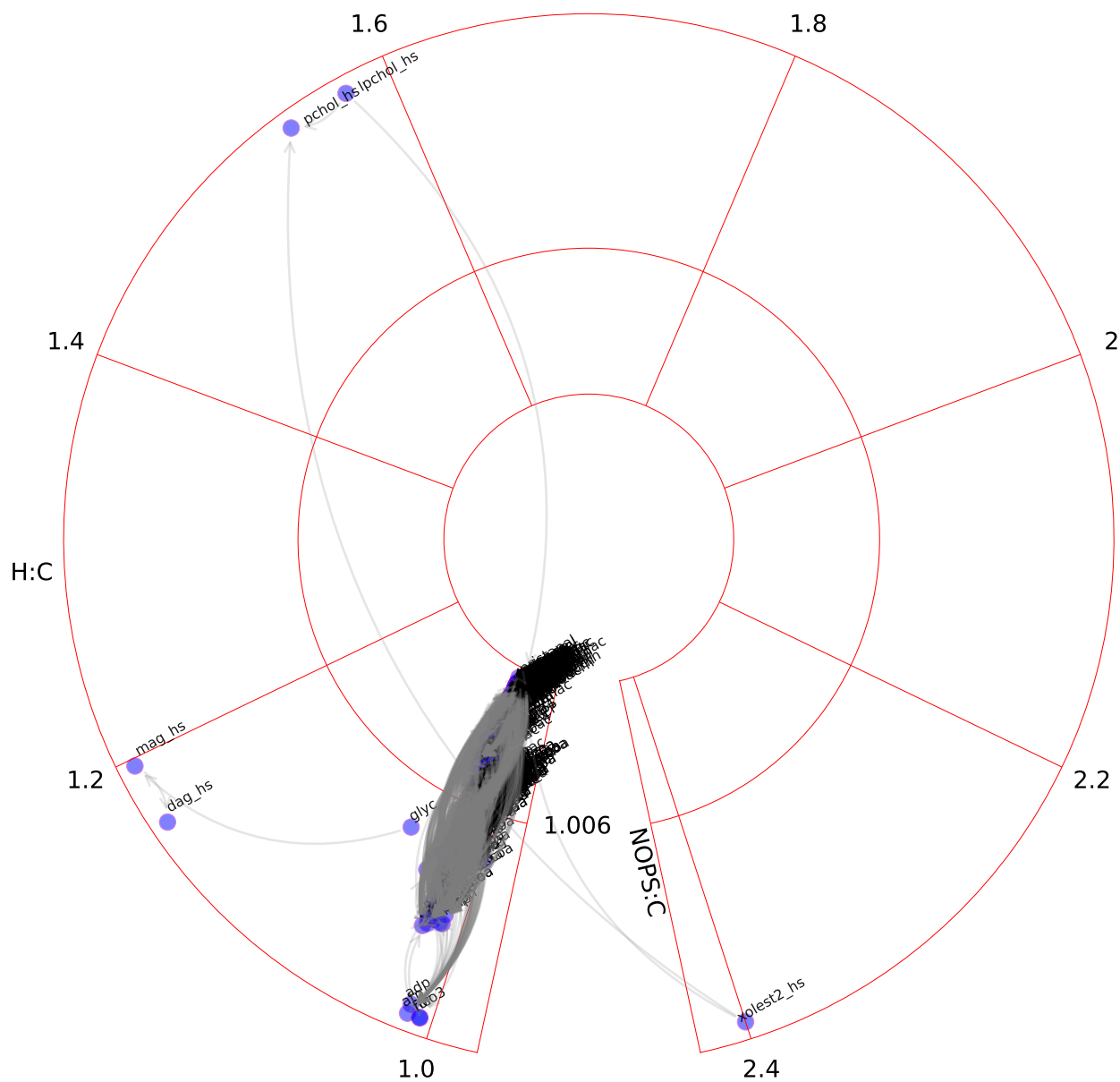

Supplement: Supplement 1 [file media-1.zip › Suppl_File_all_pathways/labeled/Fatty acid oxidation.pdf]

## Vitamin B2 metabolism

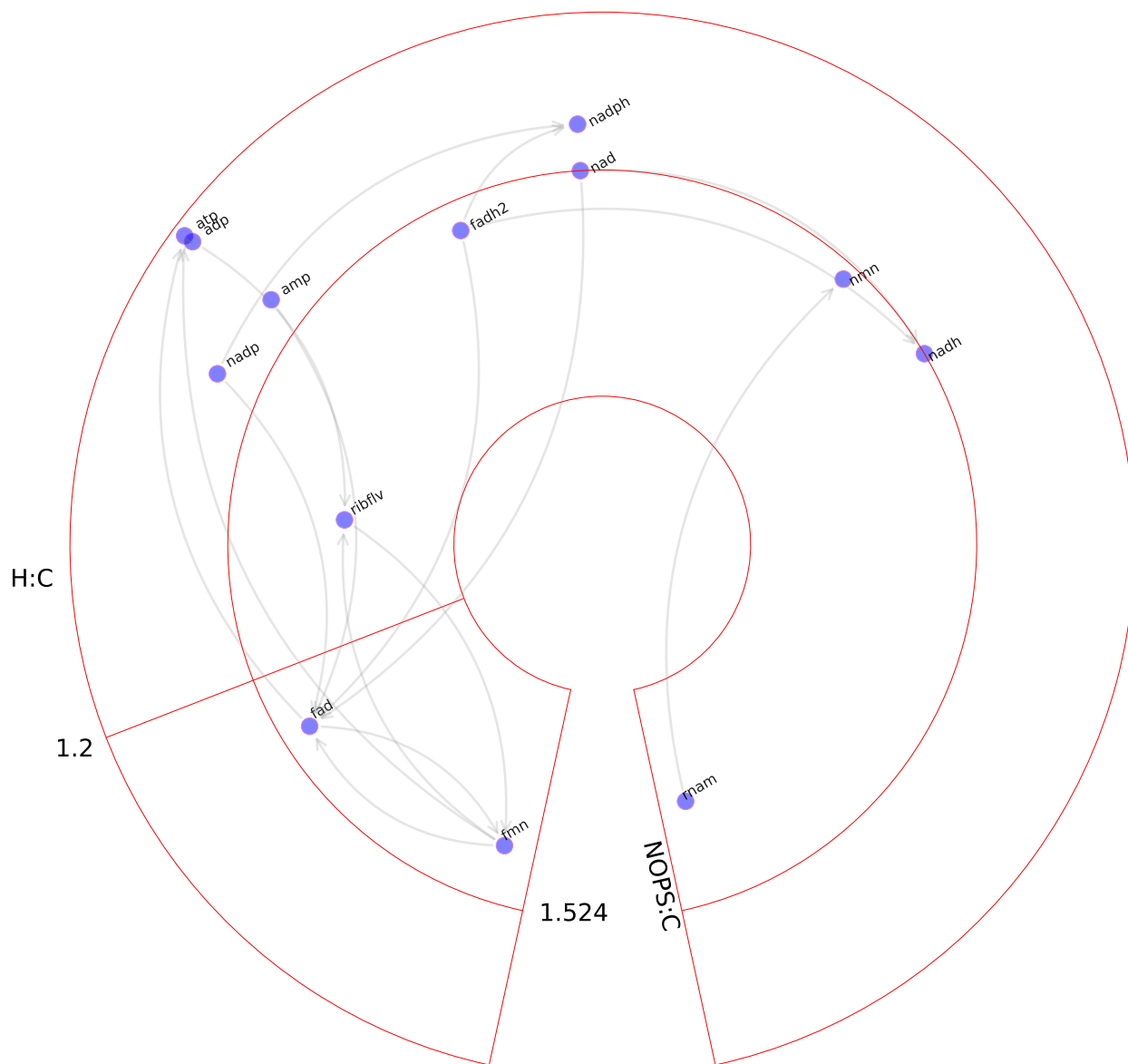

Supplement: Supplement 1 [file media-1.zip › Suppl_File_all_pathways/labeled/Vitamin B2 metabolism.pdf]

# Chondroitin sulfate degradation

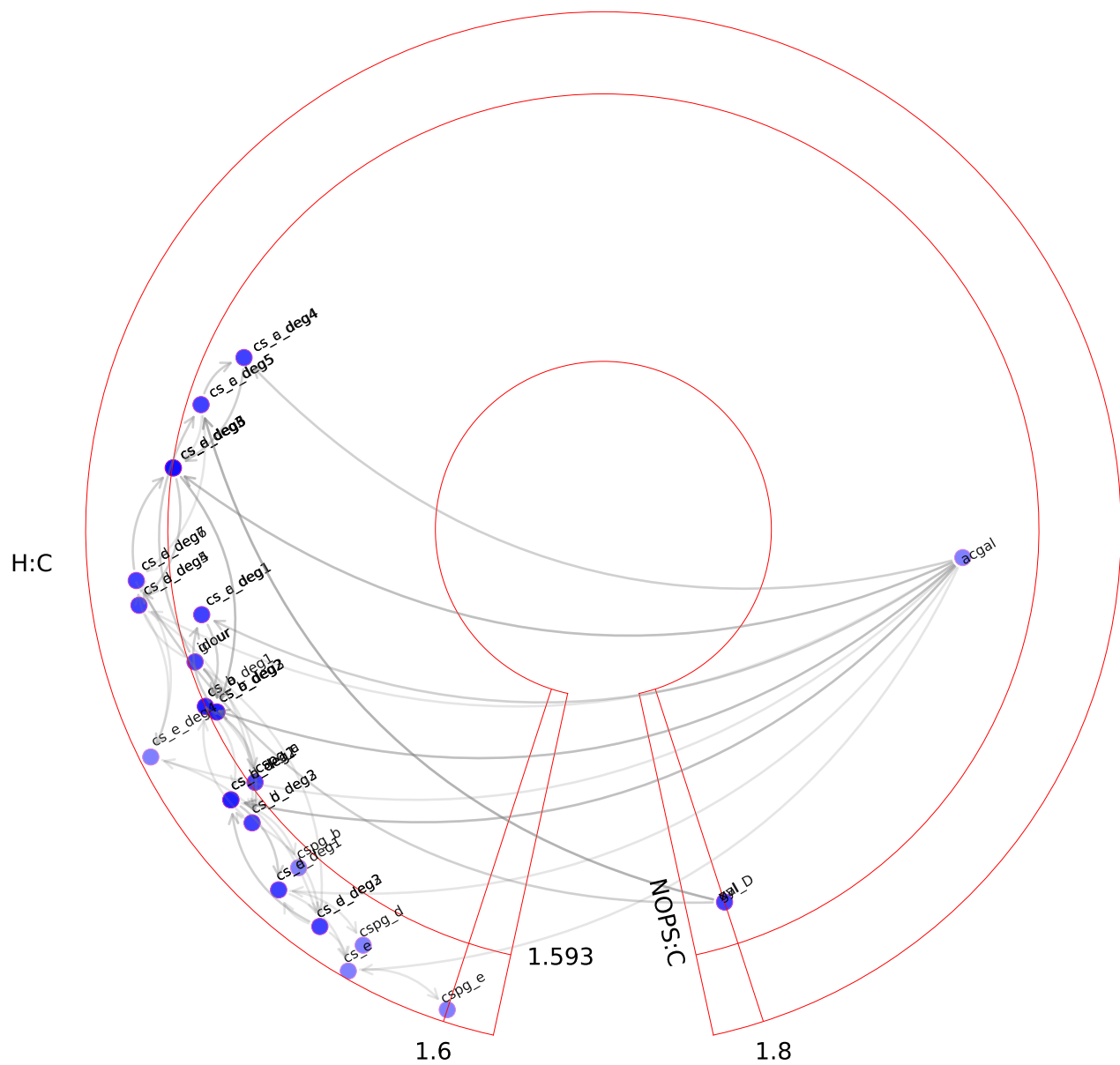

Supplement: Supplement 1 [file media-1.zip › Suppl_File_all_pathways/labeled/Chondroitin sulfate degradation.pdf]

Heme degradation

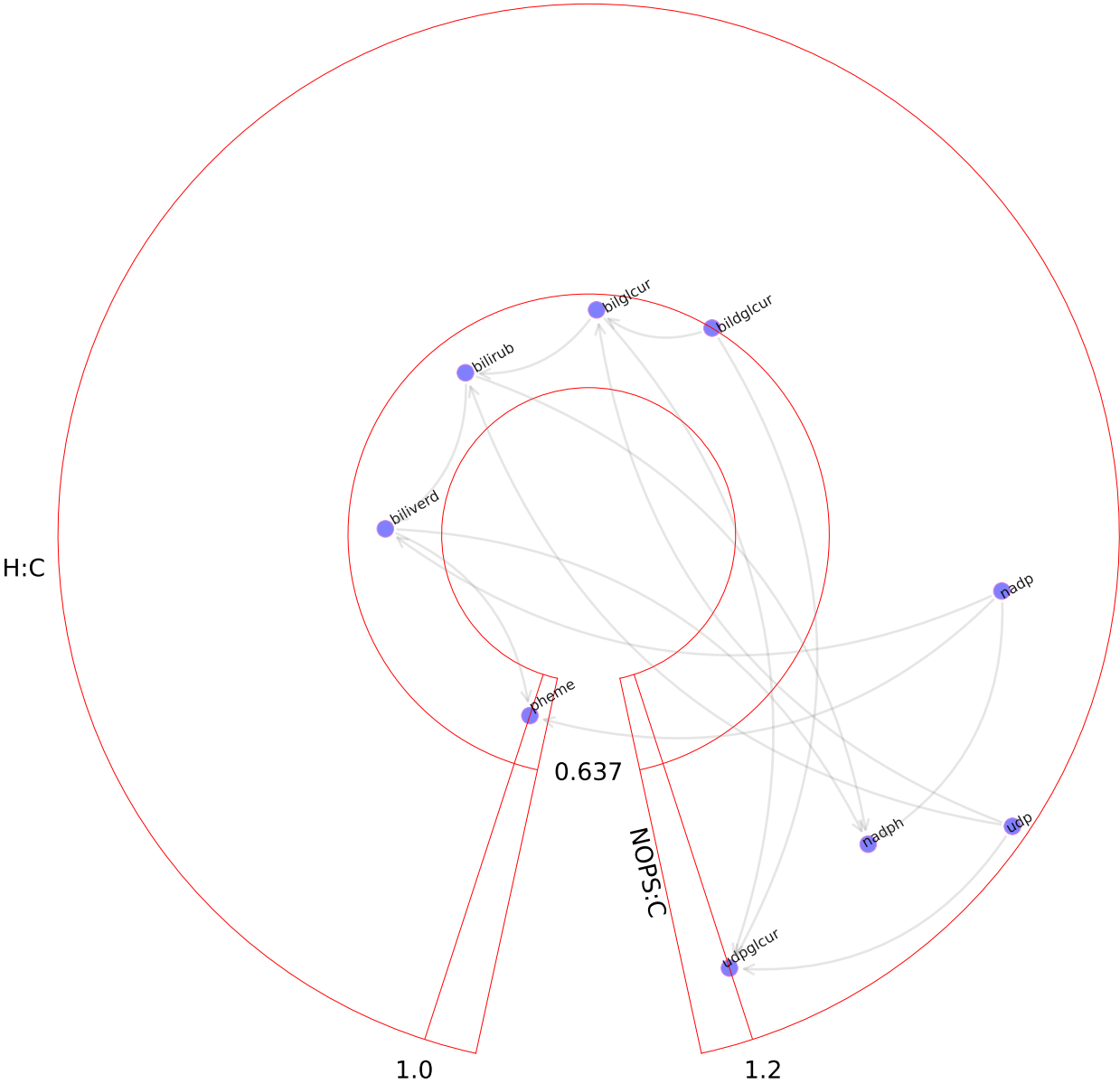

Supplement: Supplement 1 [file media-1.zip › Suppl_File_all_pathways/labeled/Heme degradation.pdf]

## Propanoate metabolism

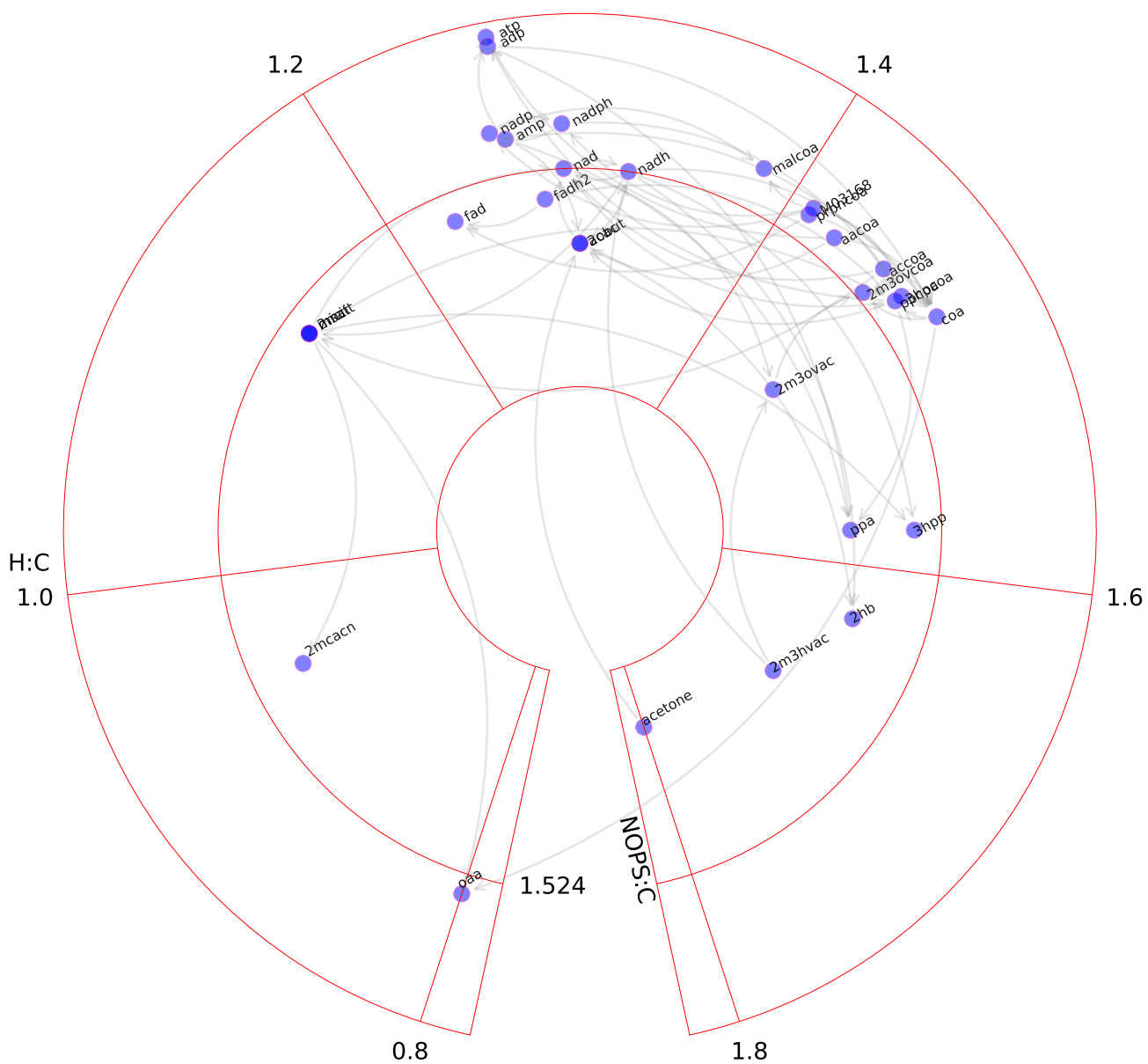

Supplement: Supplement 1 [file media-1.zip › Suppl_File_all_pathways/labeled/Propanoate metabolism.pdf]

## Galactose metabolism

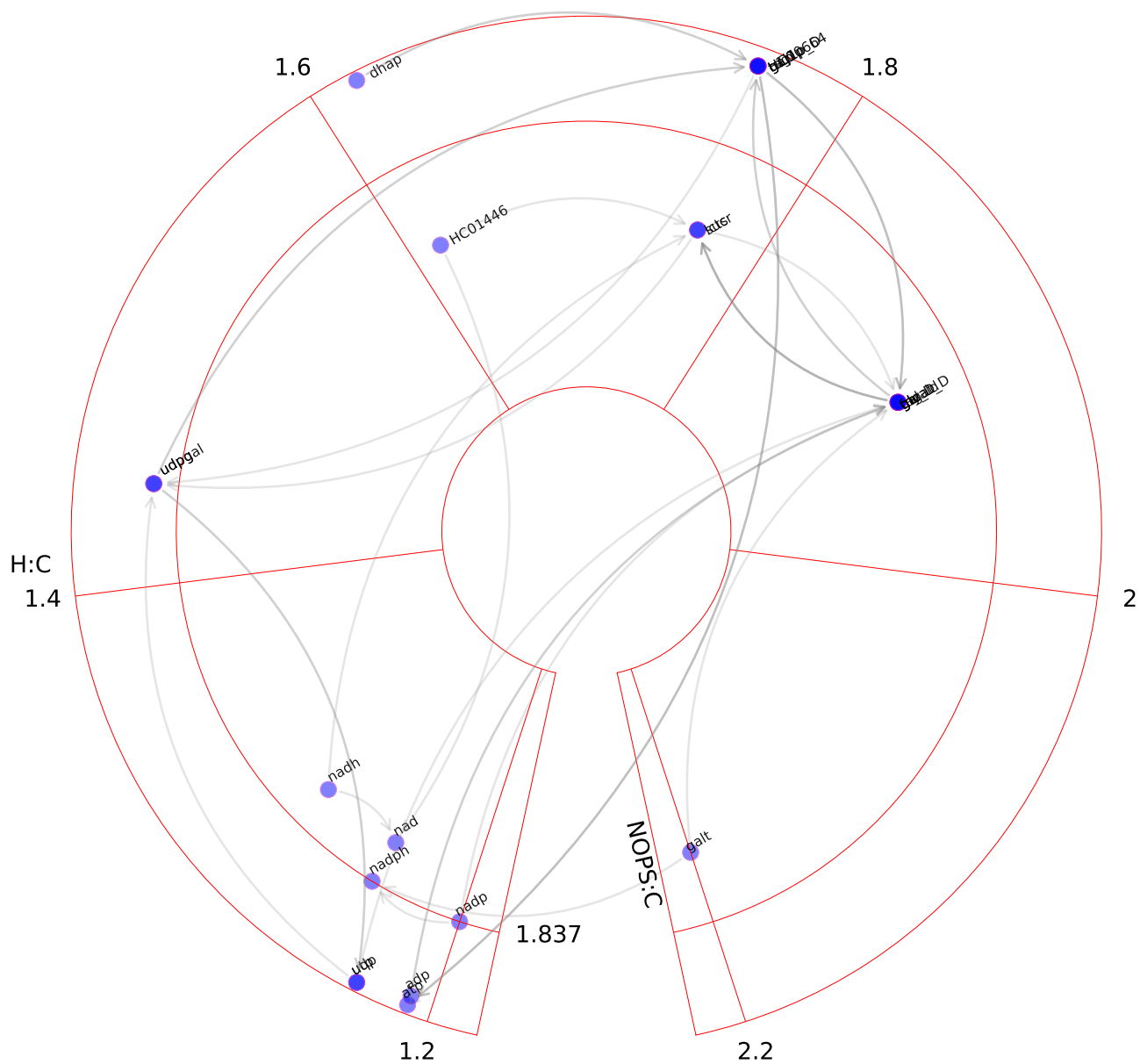

Supplement: Supplement 1 [file media-1.zip › Suppl_File_all_pathways/labeled/Galactose metabolism.pdf]

Pentose phosphate pathway

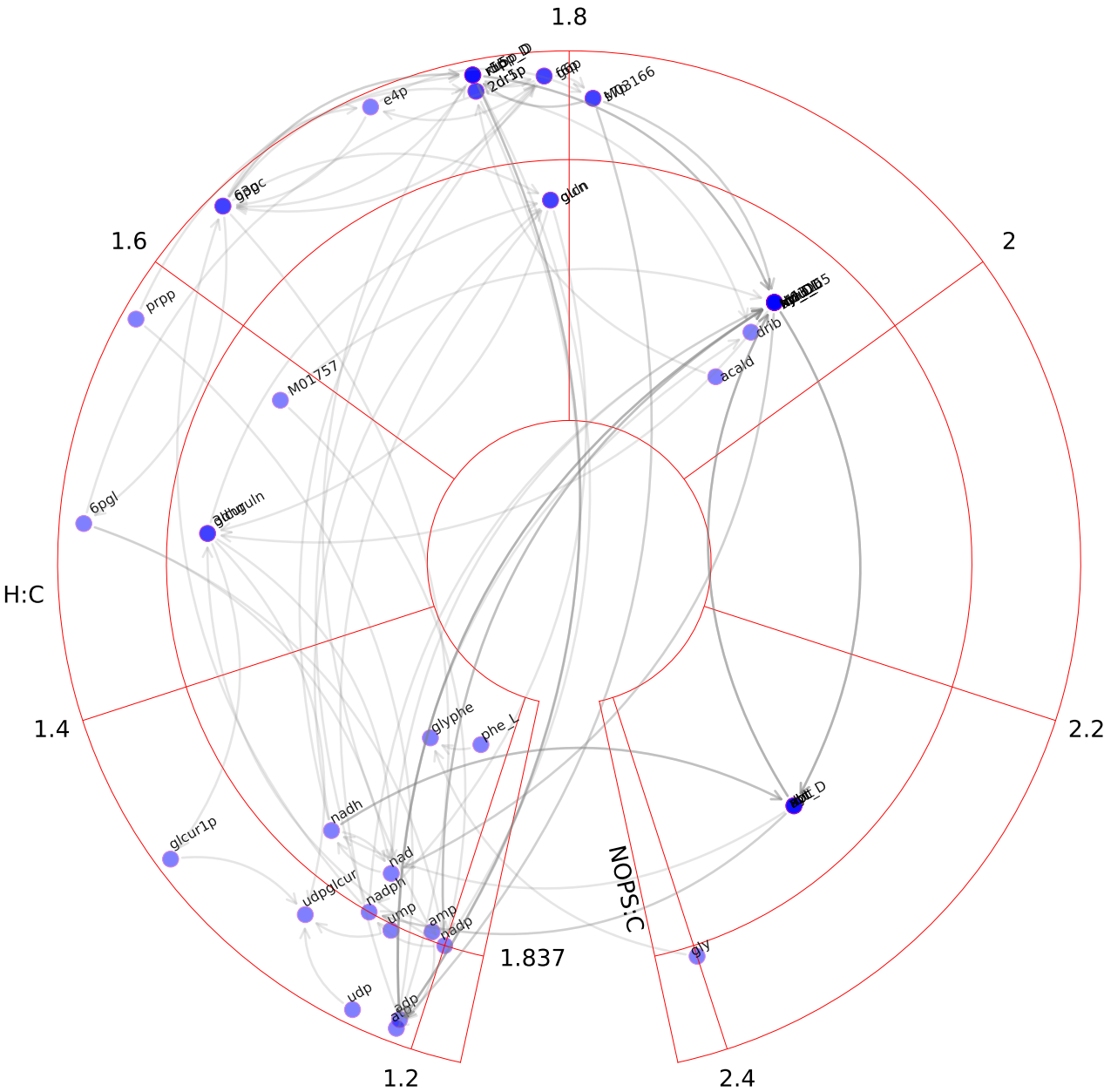

Supplement: Supplement 1 [file media-1.zip › Suppl_File_all_pathways/labeled/Pentose phosphate pathway.pdf]

## Nucleotide interconversion

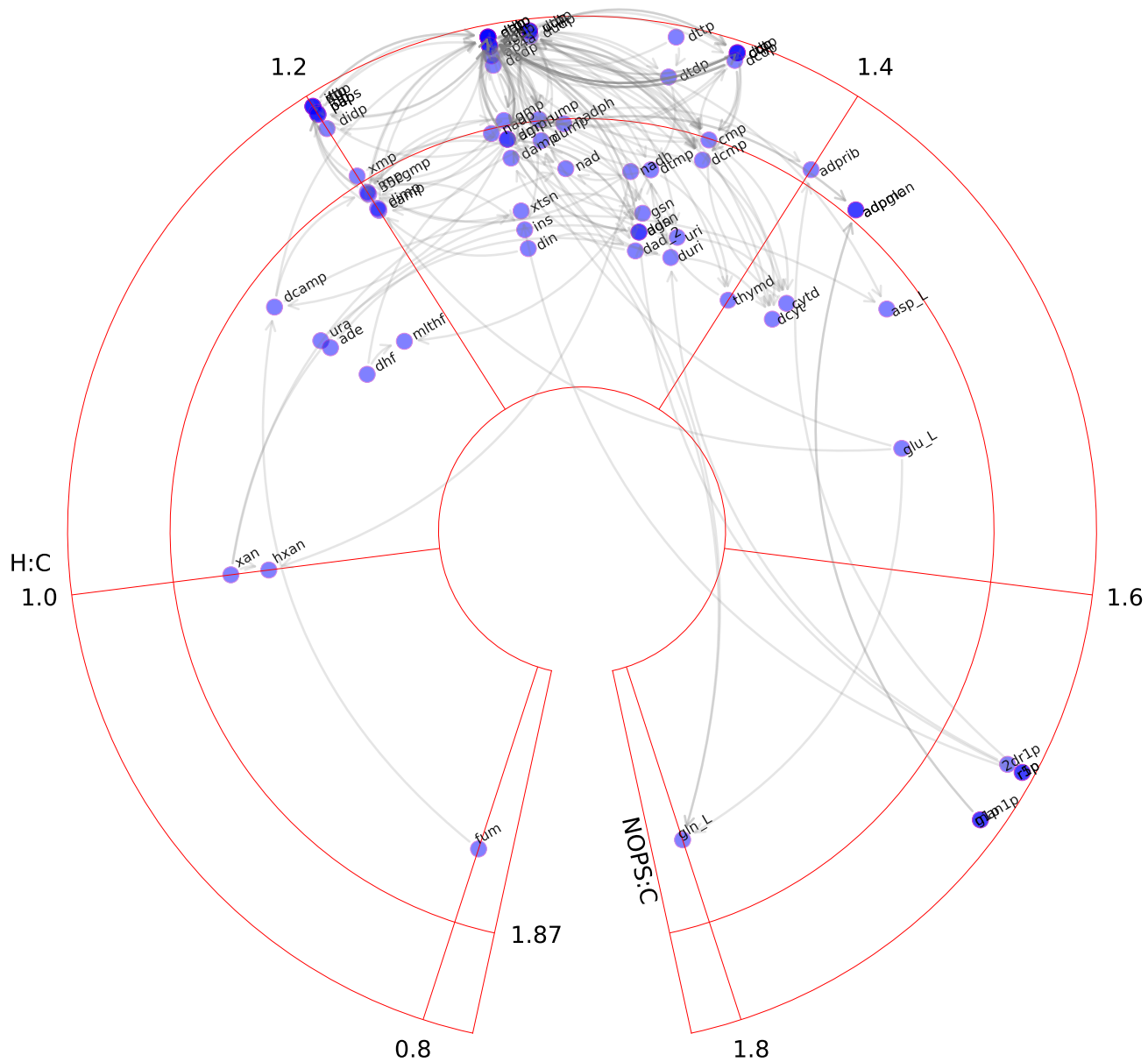

Supplement: Supplement 1 [file media-1.zip › Suppl_File_all_pathways/labeled/Nucleotide interconversion.pdf]

# Alanine and aspartate metabolism

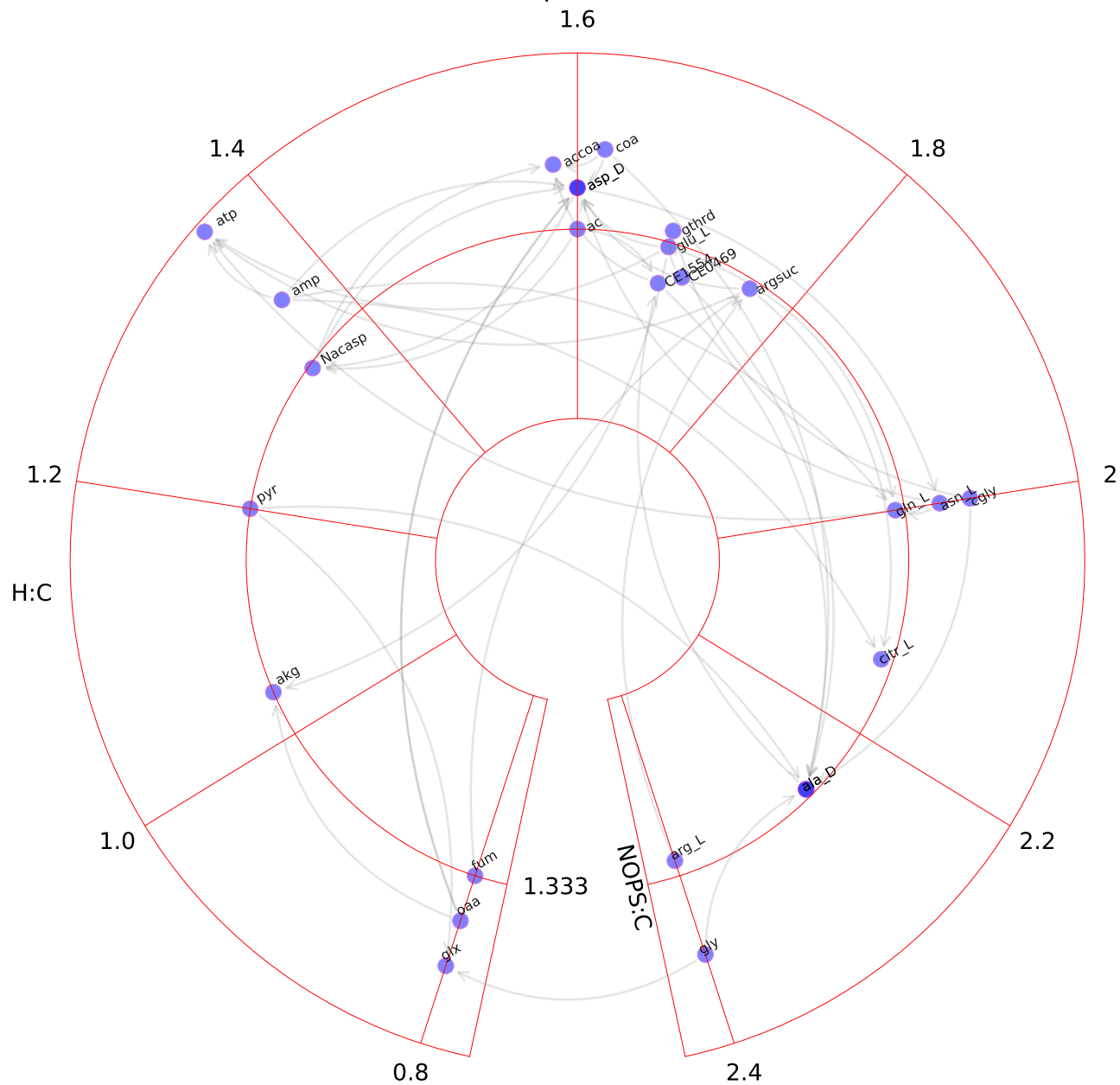

Supplement: Supplement 1 [file media-1.zip › Suppl_File_all_pathways/labeled/Alanine and aspartate metabolism.pdf]

Dietary fiber binding

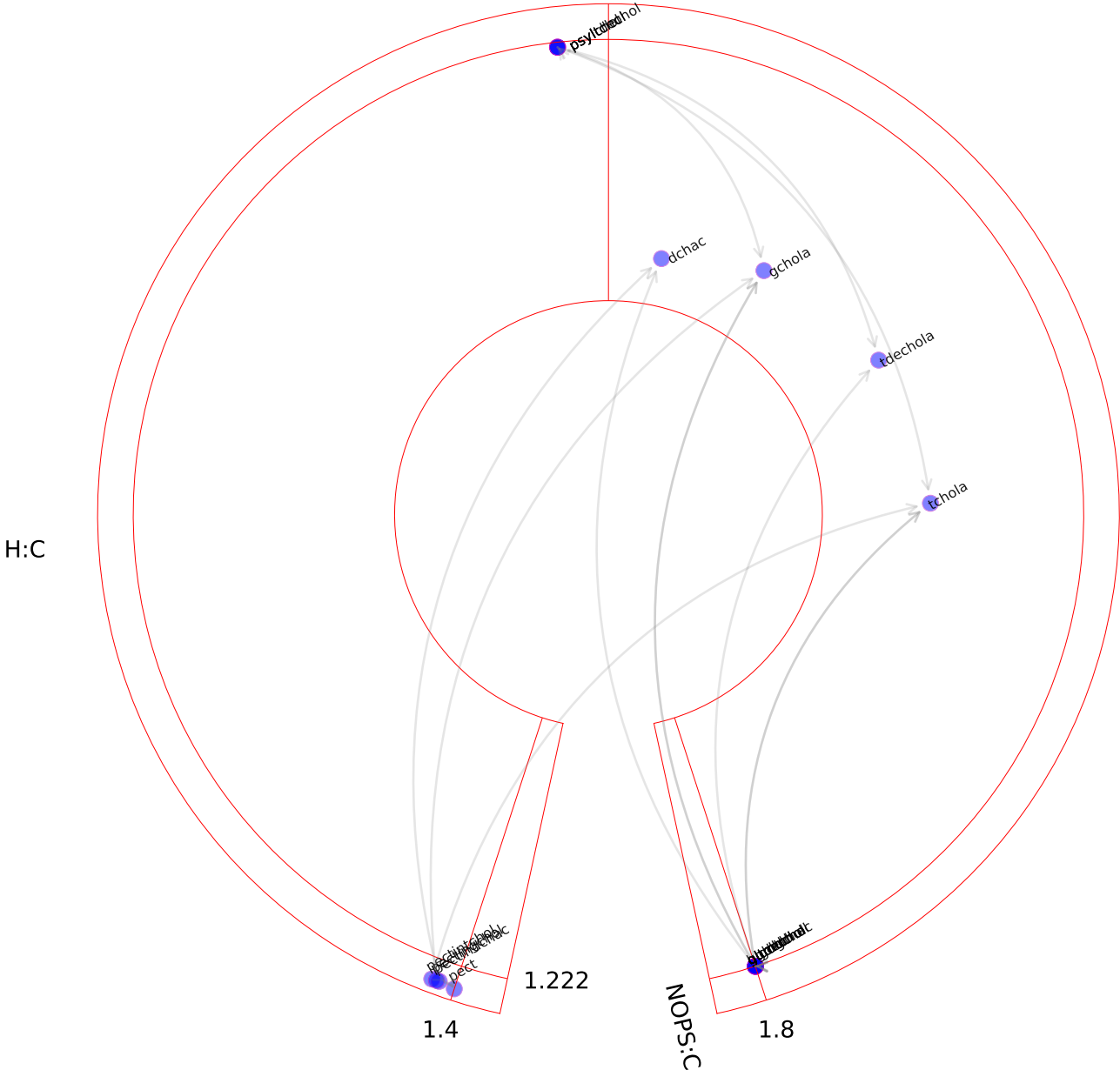

Supplement: Supplement 1 [file media-1.zip › Suppl_File_all_pathways/labeled/Dietary fiber binding.pdf]

## CoA catabolism

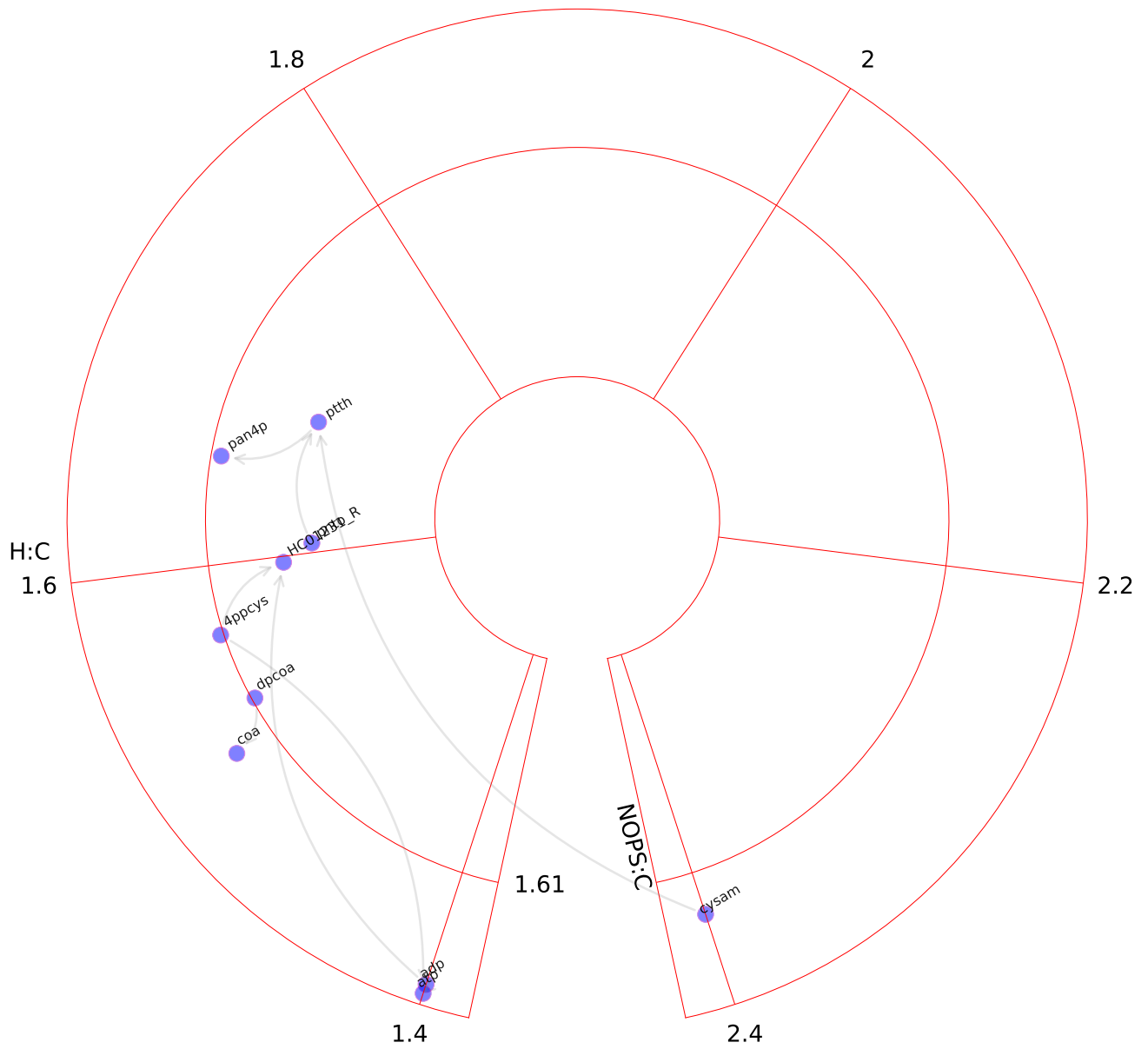

Supplement: Supplement 1 [file media-1.zip › Suppl_File_all_pathways/labeled/CoA catabolism.pdf]

# Limonene and pinene degradation

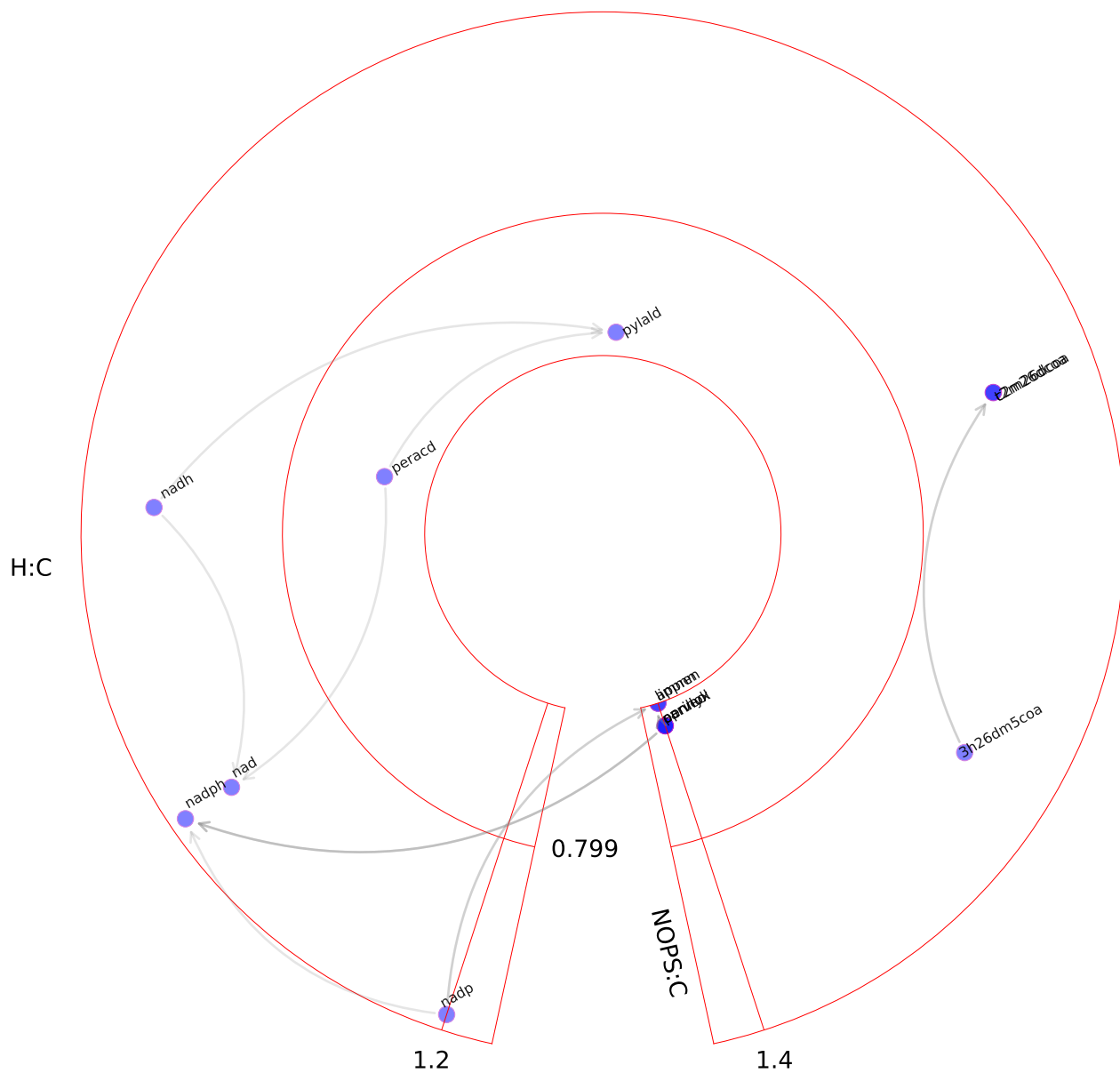

Supplement: Supplement 1 [file media-1.zip › Suppl_File_all_pathways/labeled/Limonene and pinene degradation.pdf]

## Nucleotide sugar metabolism

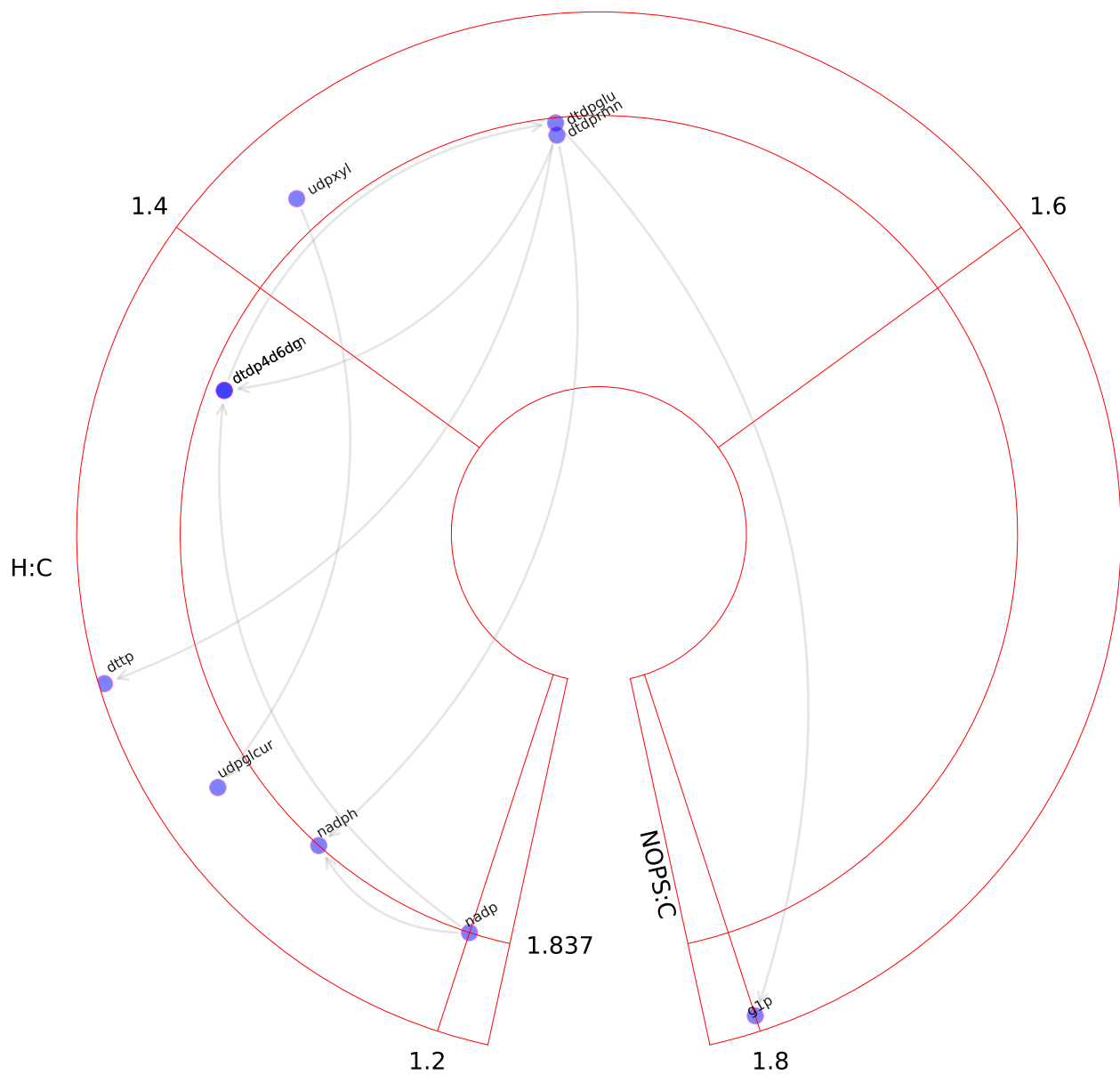

Supplement: Supplement 1 [file media-1.zip › Suppl_File_all_pathways/labeled/Nucleotide sugar metabolism.pdf]

# Fructose and mannose metabolism

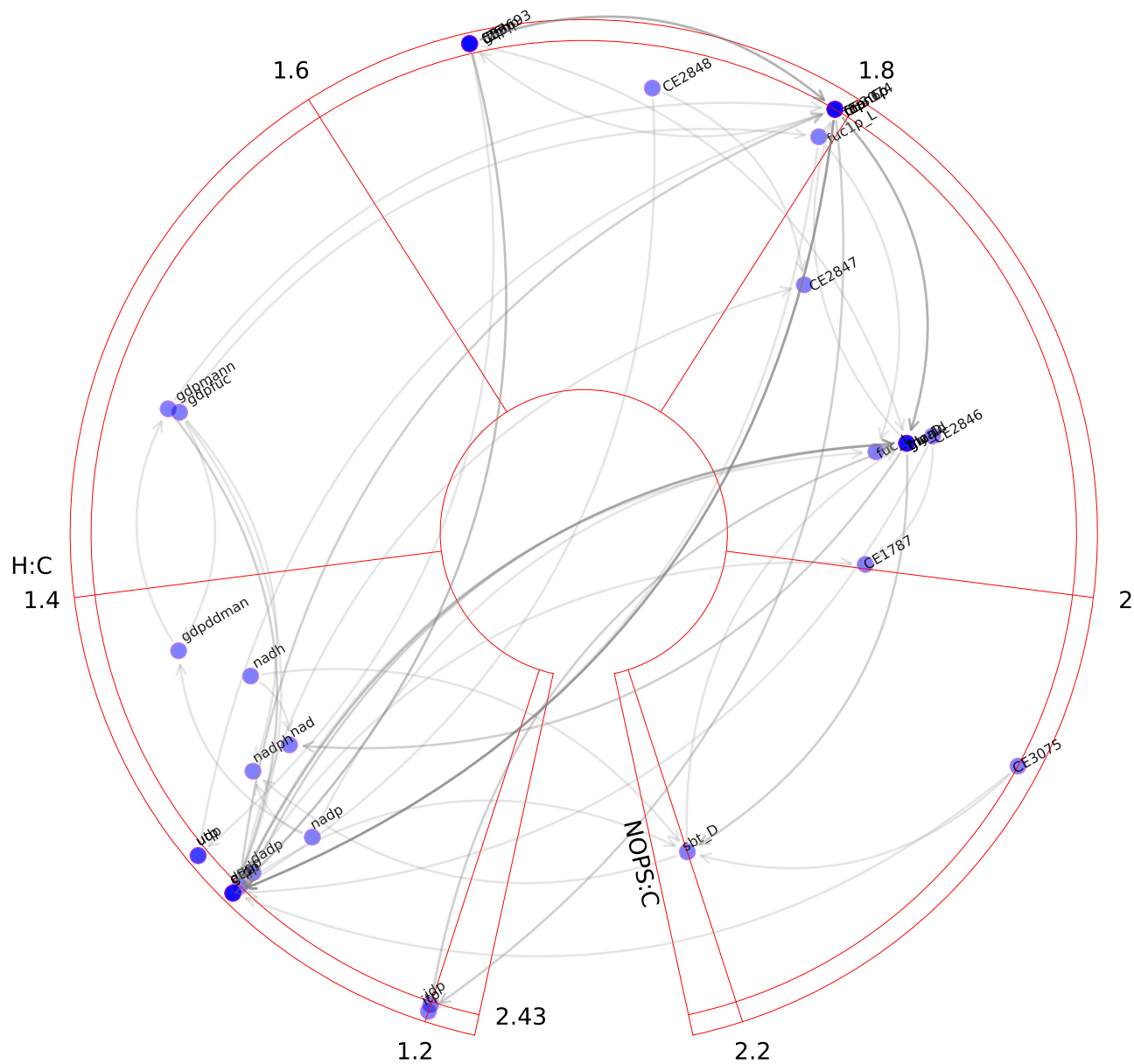

Supplement: Supplement 1 [file media-1.zip › Suppl_File_all_pathways/labeled/Fructose and mannose metabolism.pdf]

## 1.4

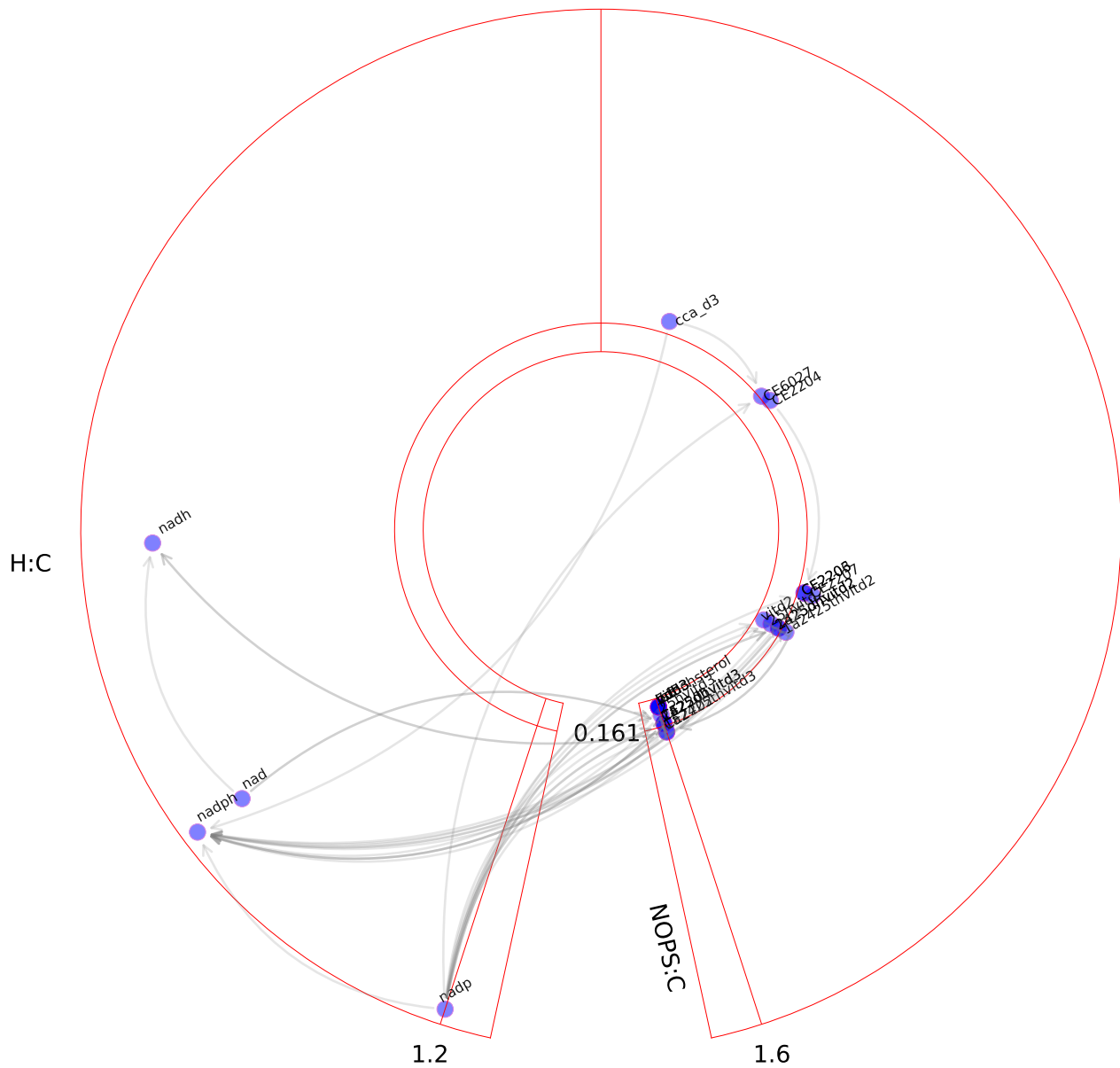

Supplement: Supplement 1 [file media-1.zip › Suppl_File_all_pathways/labeled/Vitamin D metabolism.pdf]

## 1.8

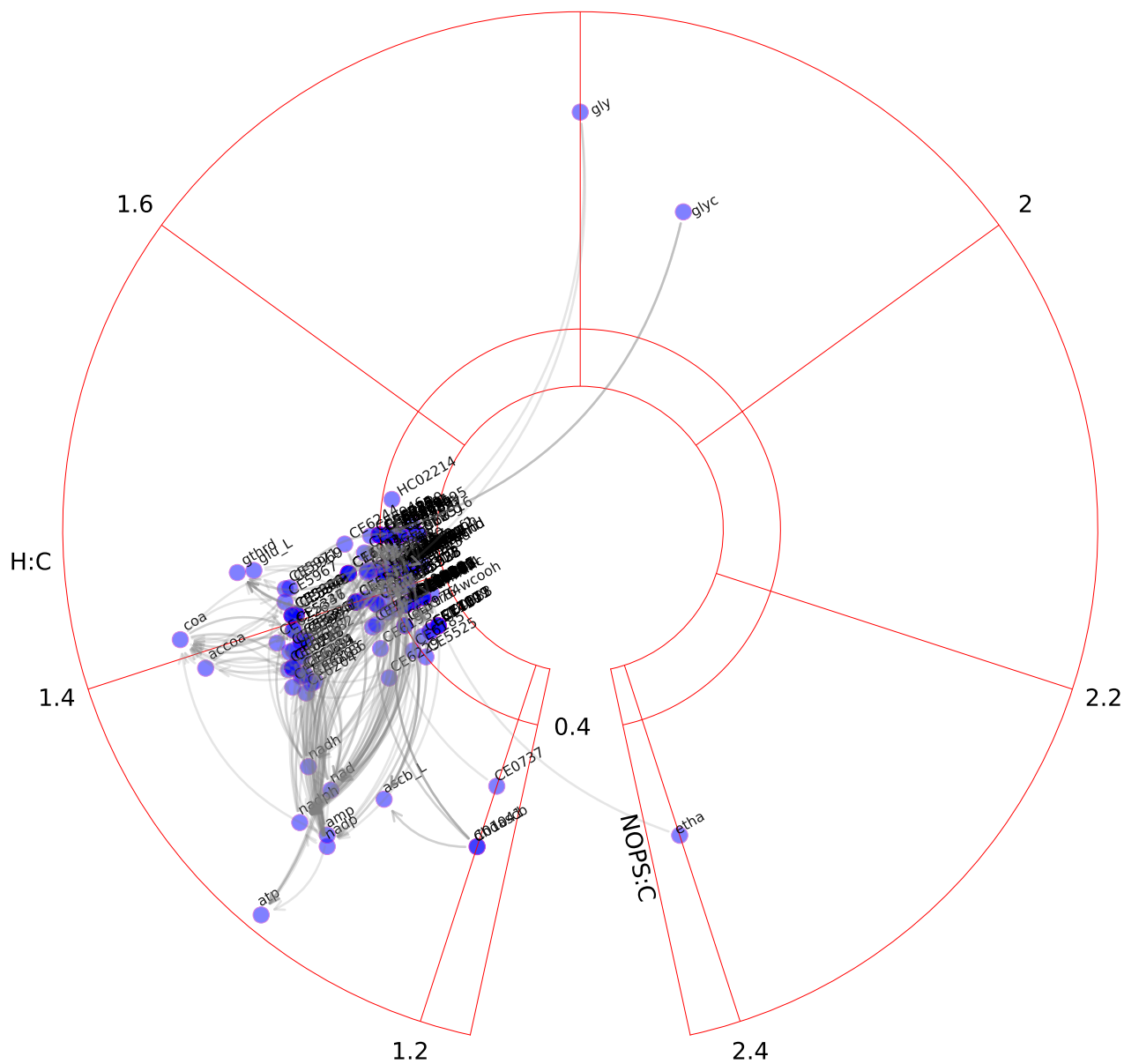

Supplement: Supplement 1 [file media-1.zip › Suppl_File_all_pathways/labeled/Eicosanoid metabolism.pdf]

# Fatty acid synthesis

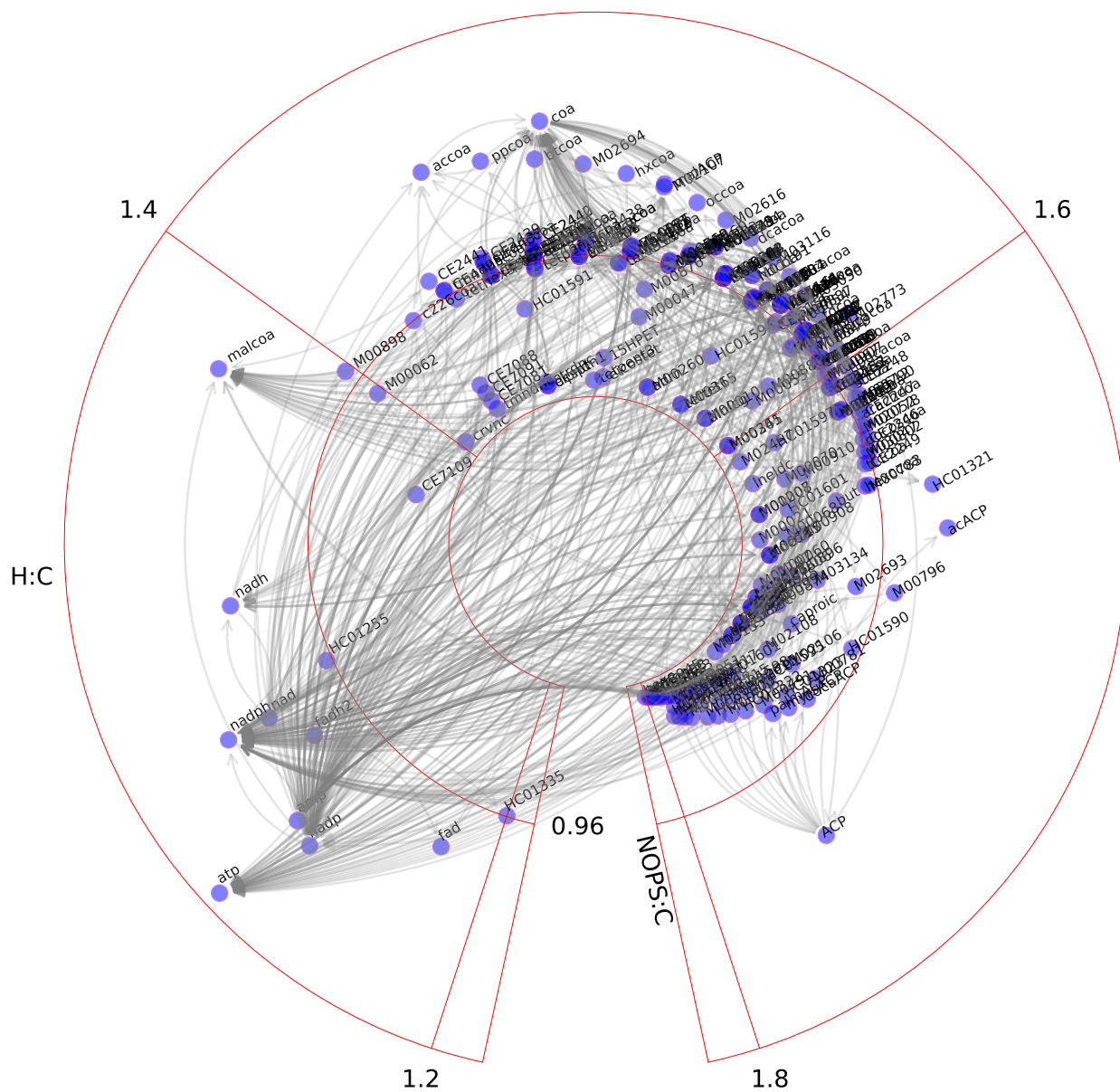

Supplement: Supplement 1 [file media-1.zip › Suppl_File_all_pathways/labeled/Fatty acid synthesis.pdf]

# Vitamin K metabolism

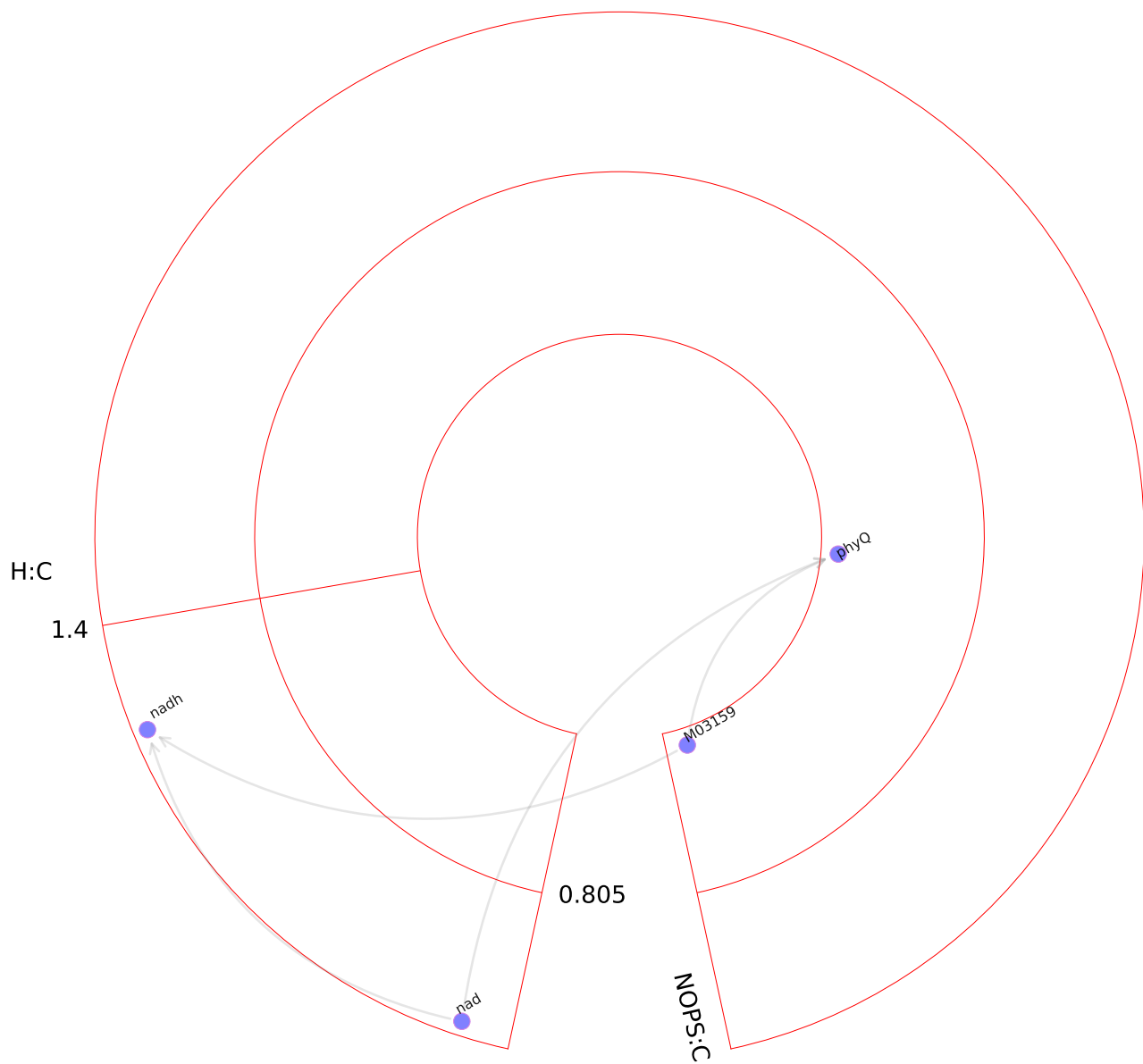

Supplement: Supplement 1 [file media-1.zip › Suppl_File_all_pathways/labeled/Vitamin K metabolism.pdf]

## R group synthesis

1.4

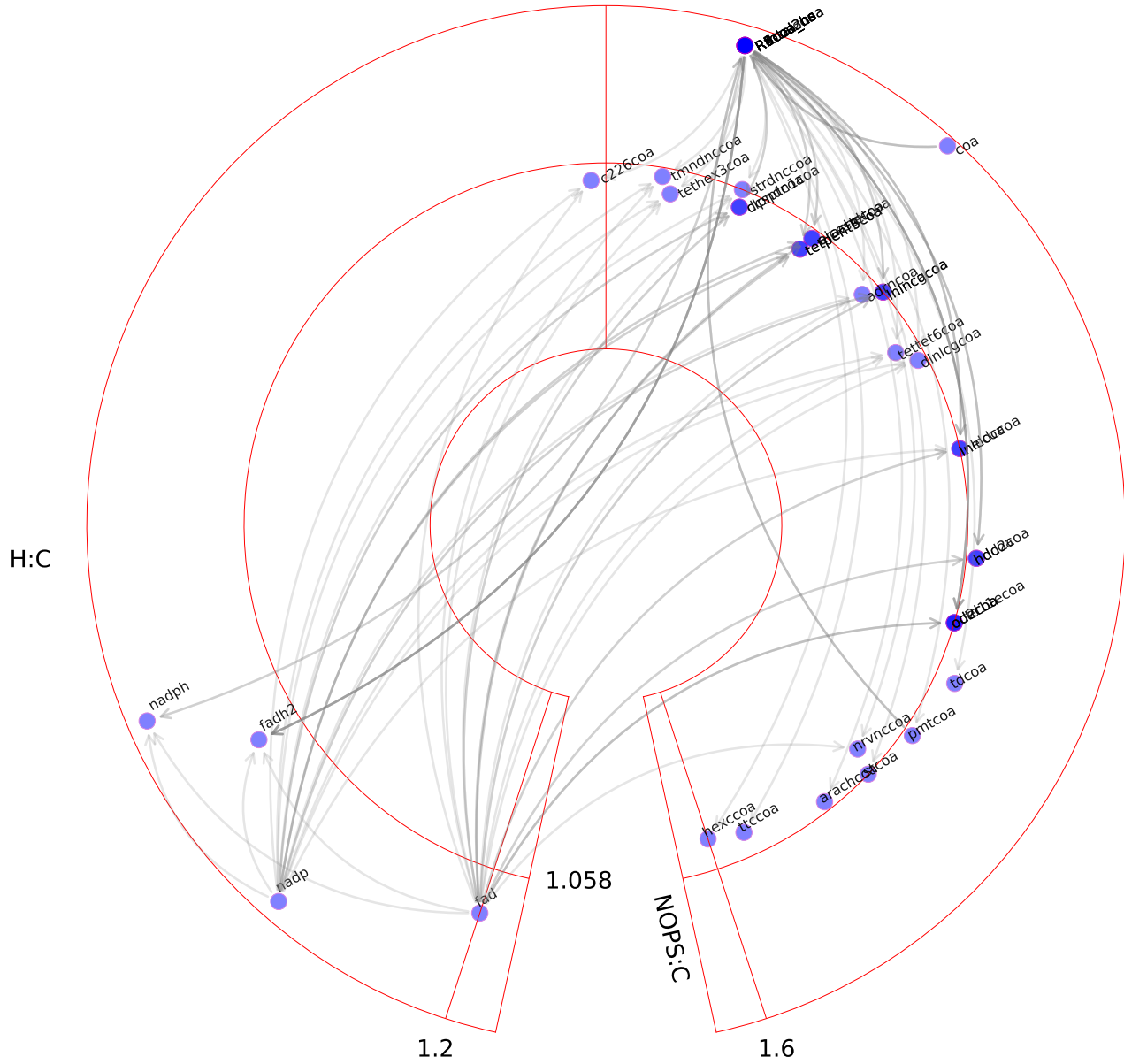

Supplement: Supplement 1 [file media-1.zip › Suppl_File_all_pathways/labeled/R group synthesis.pdf]

# Tetrahydrobiopterin metabolism

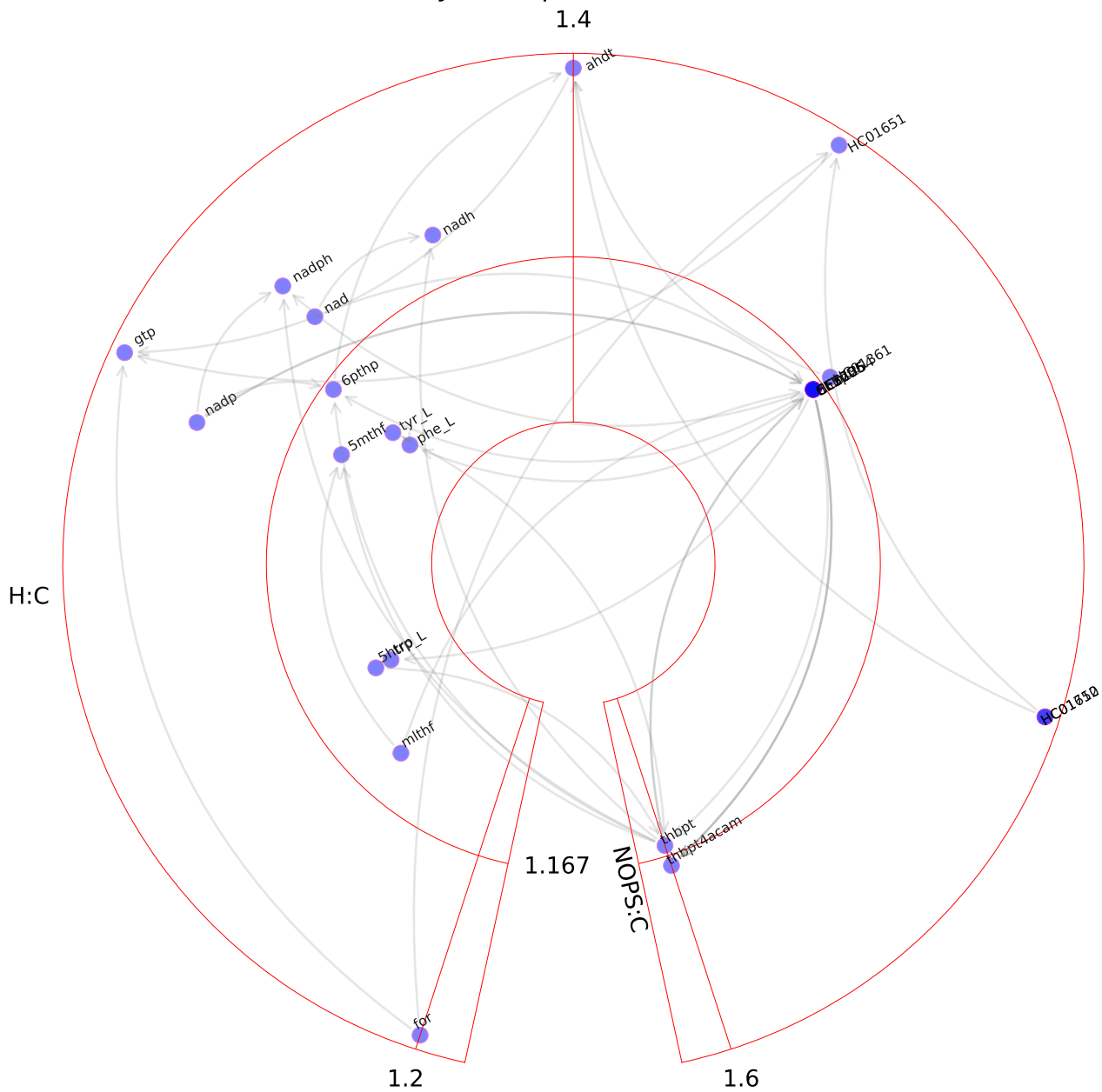

Supplement: Supplement 1 [file media-1.zip › Suppl_File_all_pathways/labeled/Tetrahydrobiopterin metabolism.pdf]

## Lysine metabolism

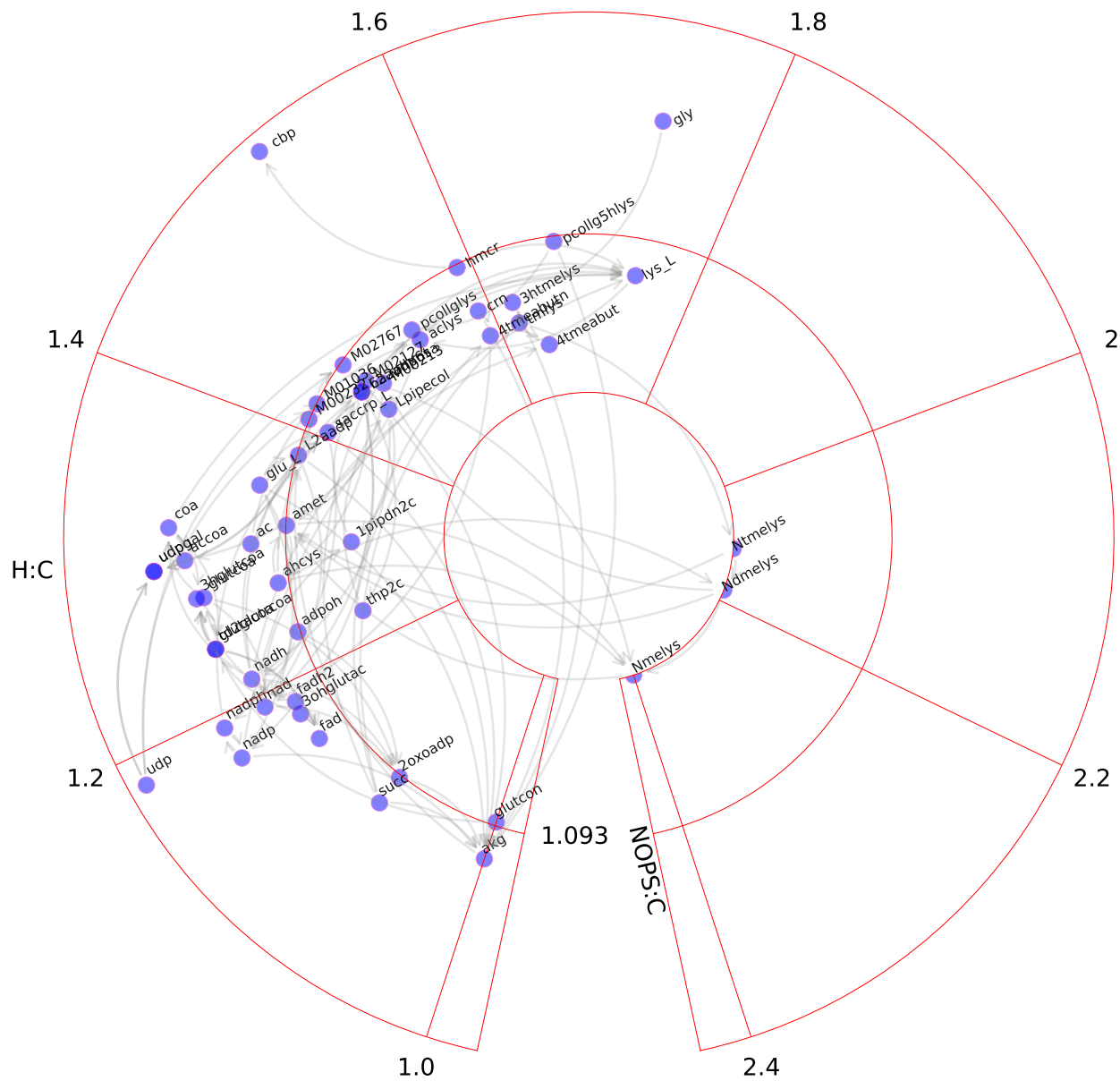

Supplement: Supplement 1 [file media-1.zip › Suppl_File_all_pathways/labeled/Lysine metabolism.pdf]

# Blood group synthesis

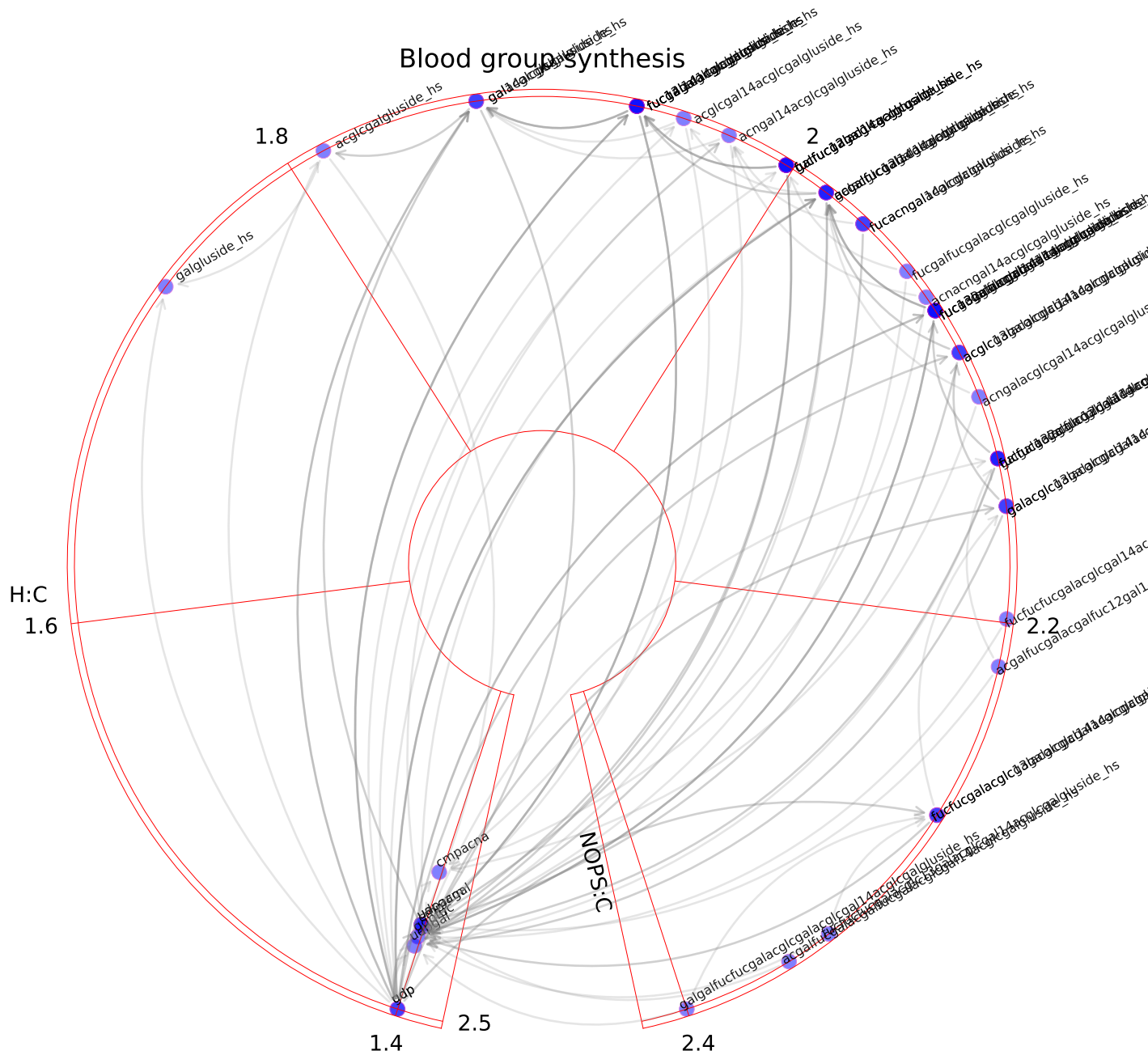

Supplement: Supplement 1 [file media-1.zip › Suppl_File_all_pathways/labeled/Blood group synthesis.pdf]

# Alkaloid synthesis

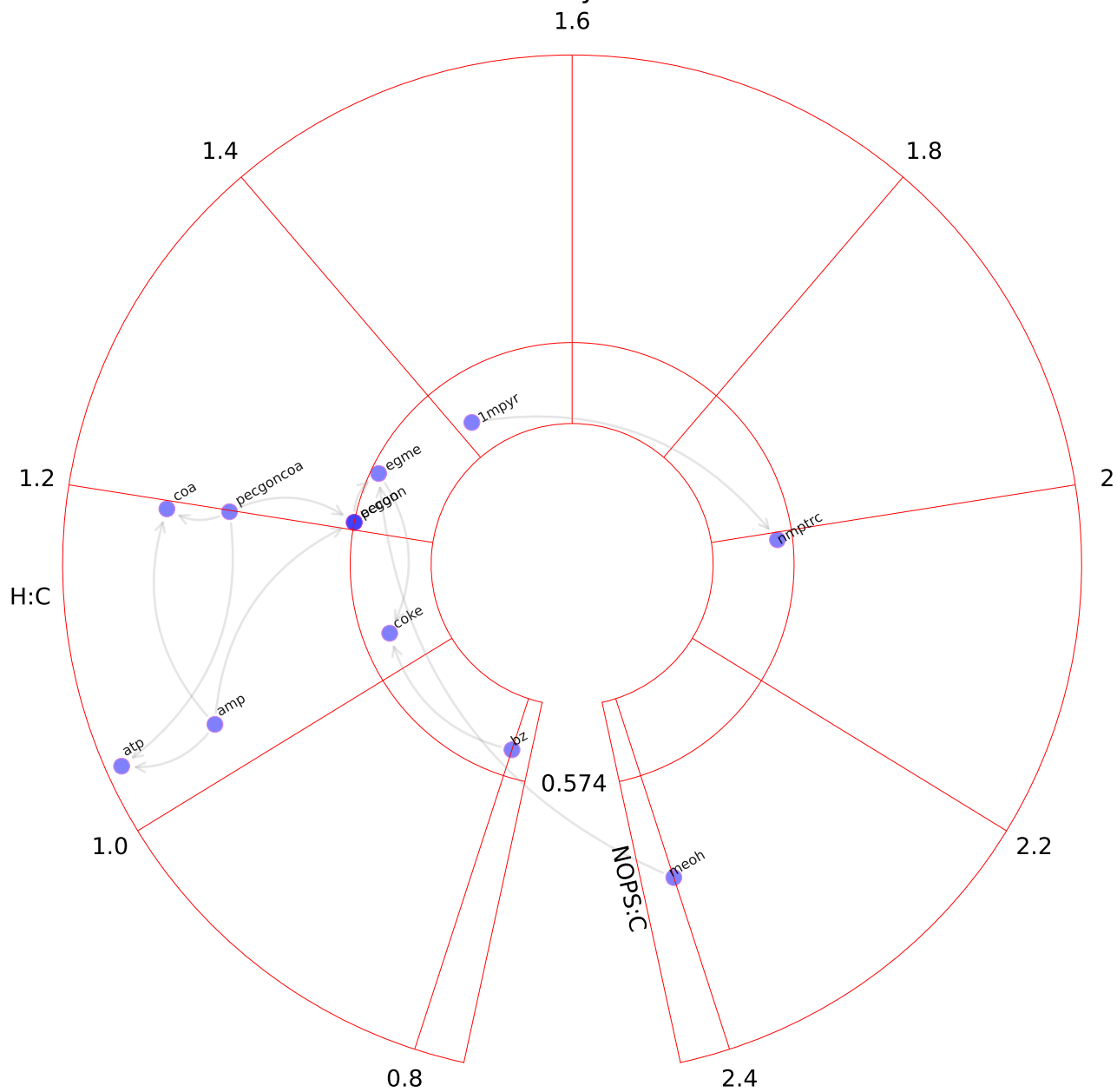

Supplement: Supplement 1 [file media-1.zip › Suppl_File_all_pathways/labeled/Alkaloid synthesis.pdf]

# N-glycan degradation

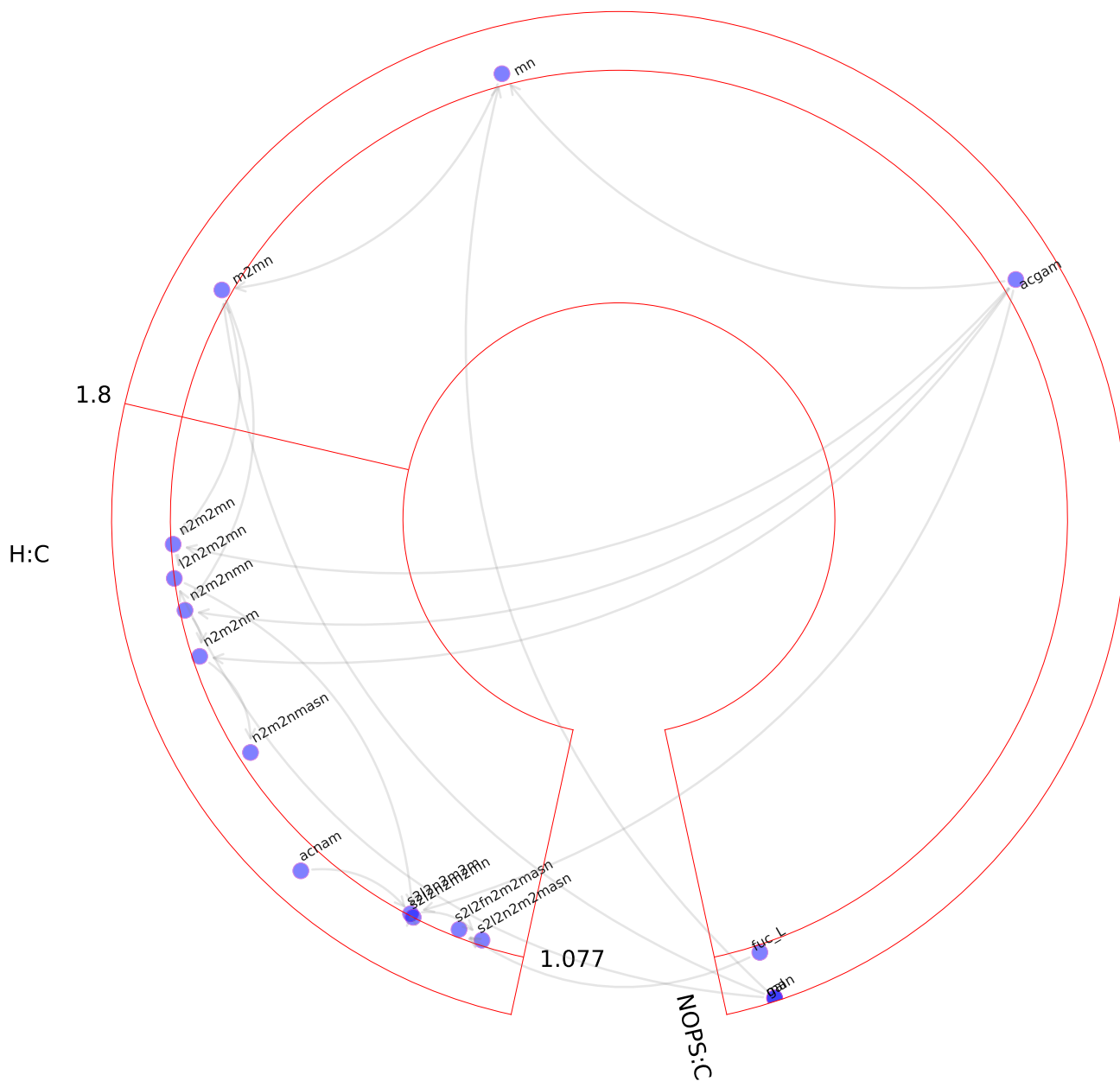

Supplement: Supplement 1 [file media-1.zip › Suppl_File_all_pathways/labeled/N-glycan degradation.pdf]

## Squalene and cholesterol synthesis

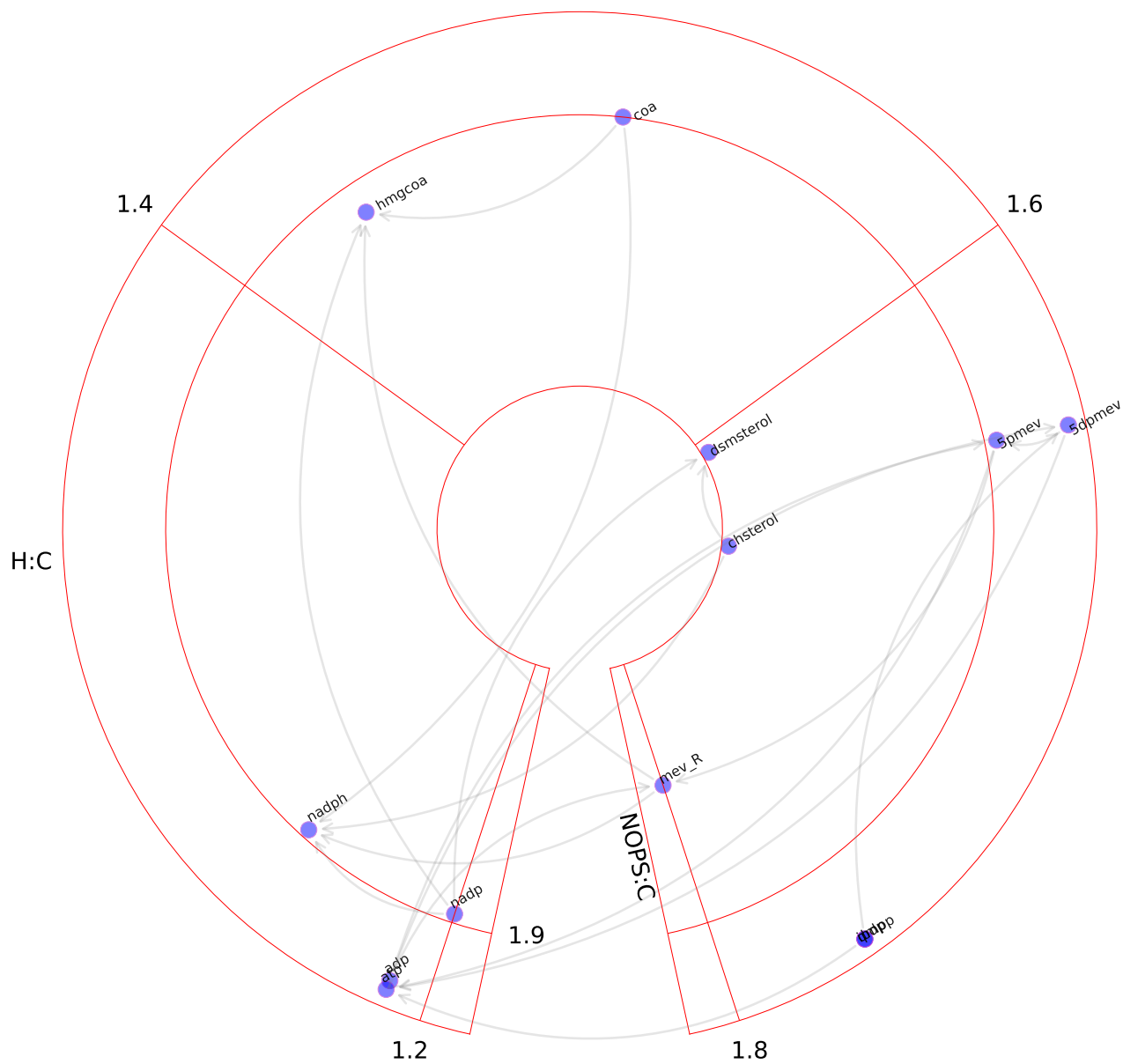

Supplement: Supplement 1 [file media-1.zip › Suppl_File_all_pathways/labeled/Squalene and cholesterol synthesis.pdf]

# O-glycan metabolism

1.8

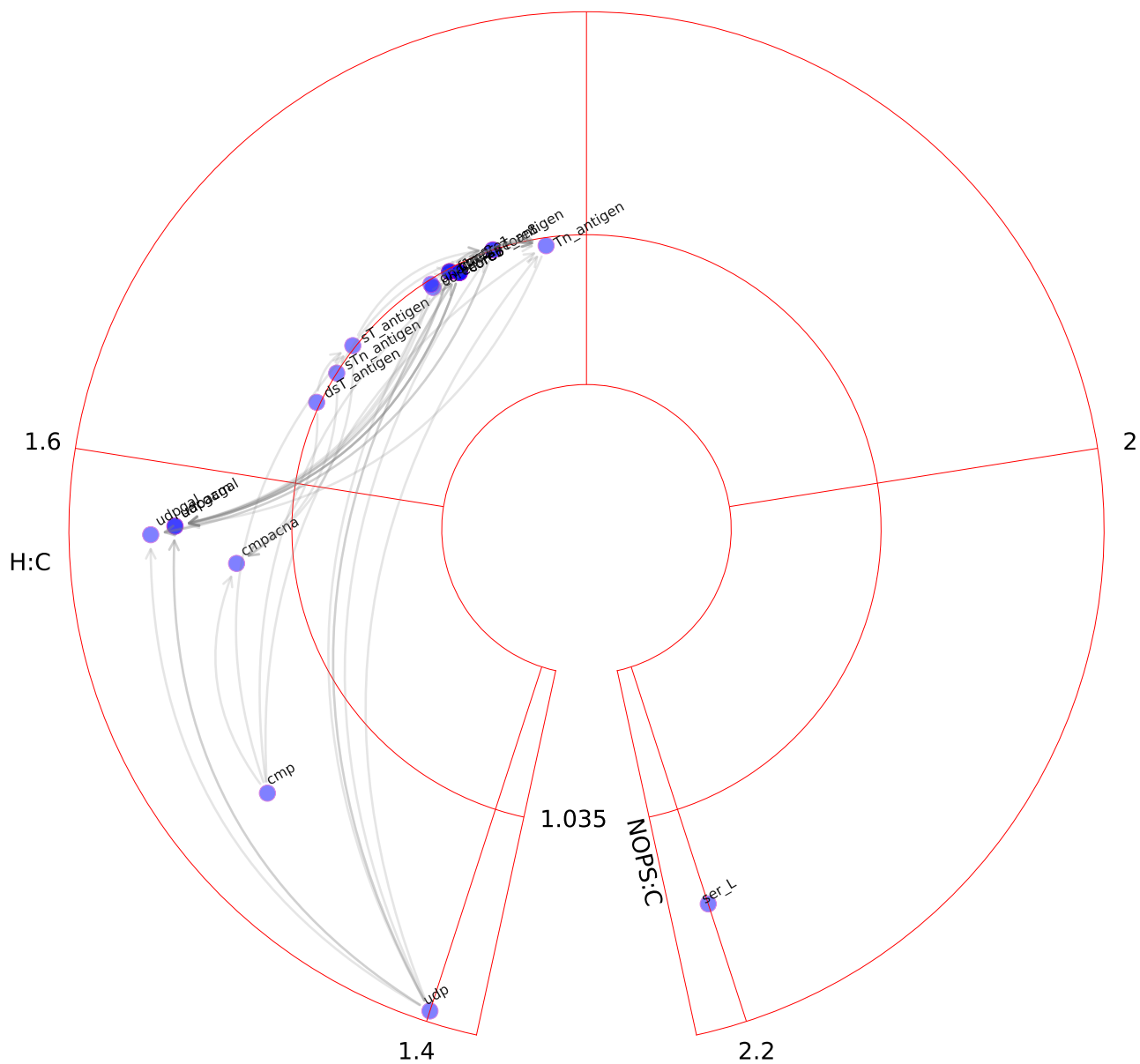

Supplement: Supplement 1 [file media-1.zip › Suppl_File_all_pathways/labeled/O-glycan metabolism.pdf]

# Vitamin C metabolism

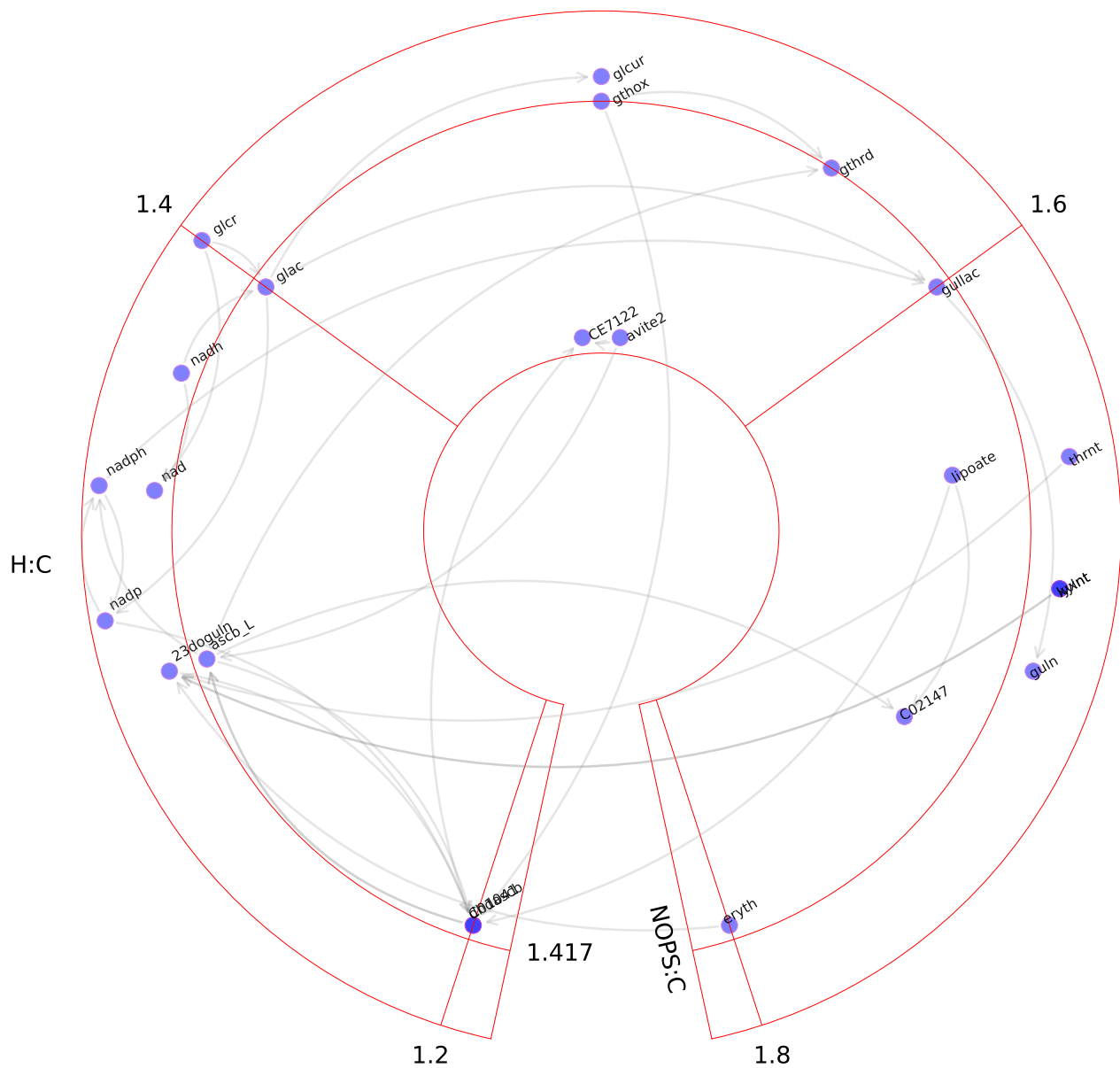

Supplement: Supplement 1 [file media-1.zip › Suppl_File_all_pathways/labeled/Vitamin C metabolism.pdf]

# Citric acid cycle

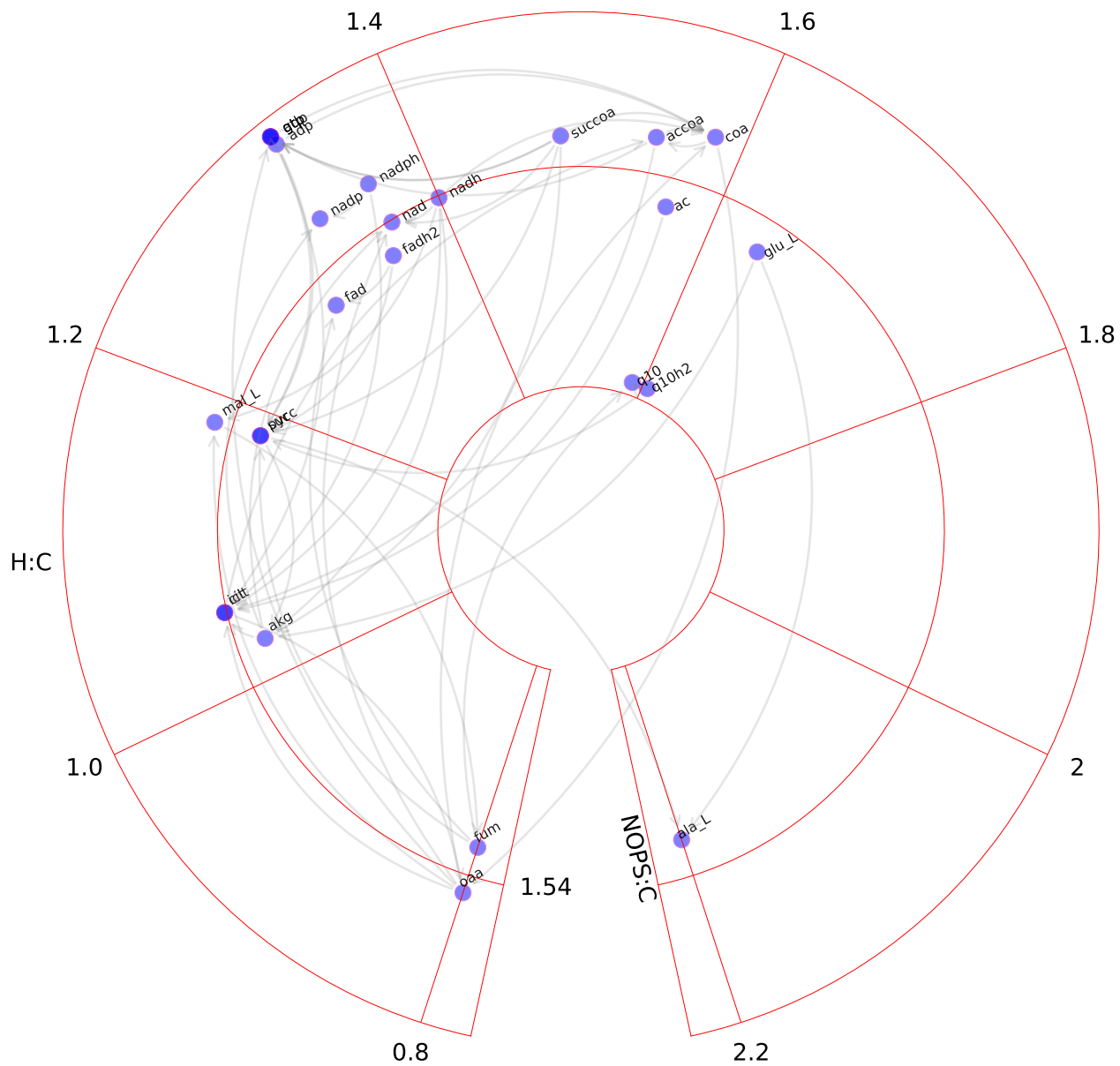

Supplement: Supplement 1 [file media-1.zip › Suppl_File_all_pathways/labeled/Citric acid cycle.pdf]

# Taurine and hypotaurine metabolism

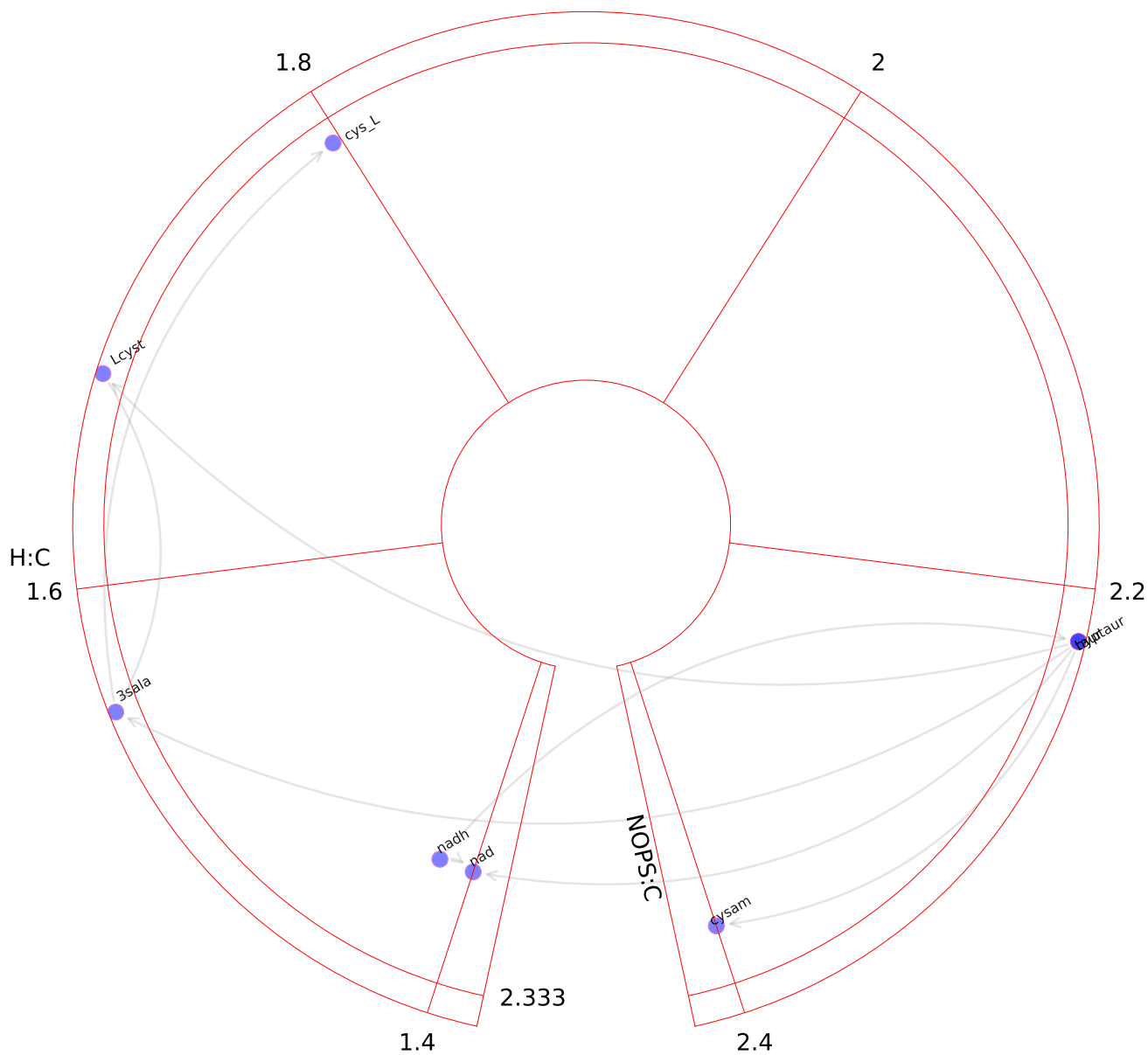

Supplement: Supplement 1 [file media-1.zip › Suppl_File_all_pathways/labeled/Taurine and hypotaurine metabolism.pdf]

# Purine synthesis

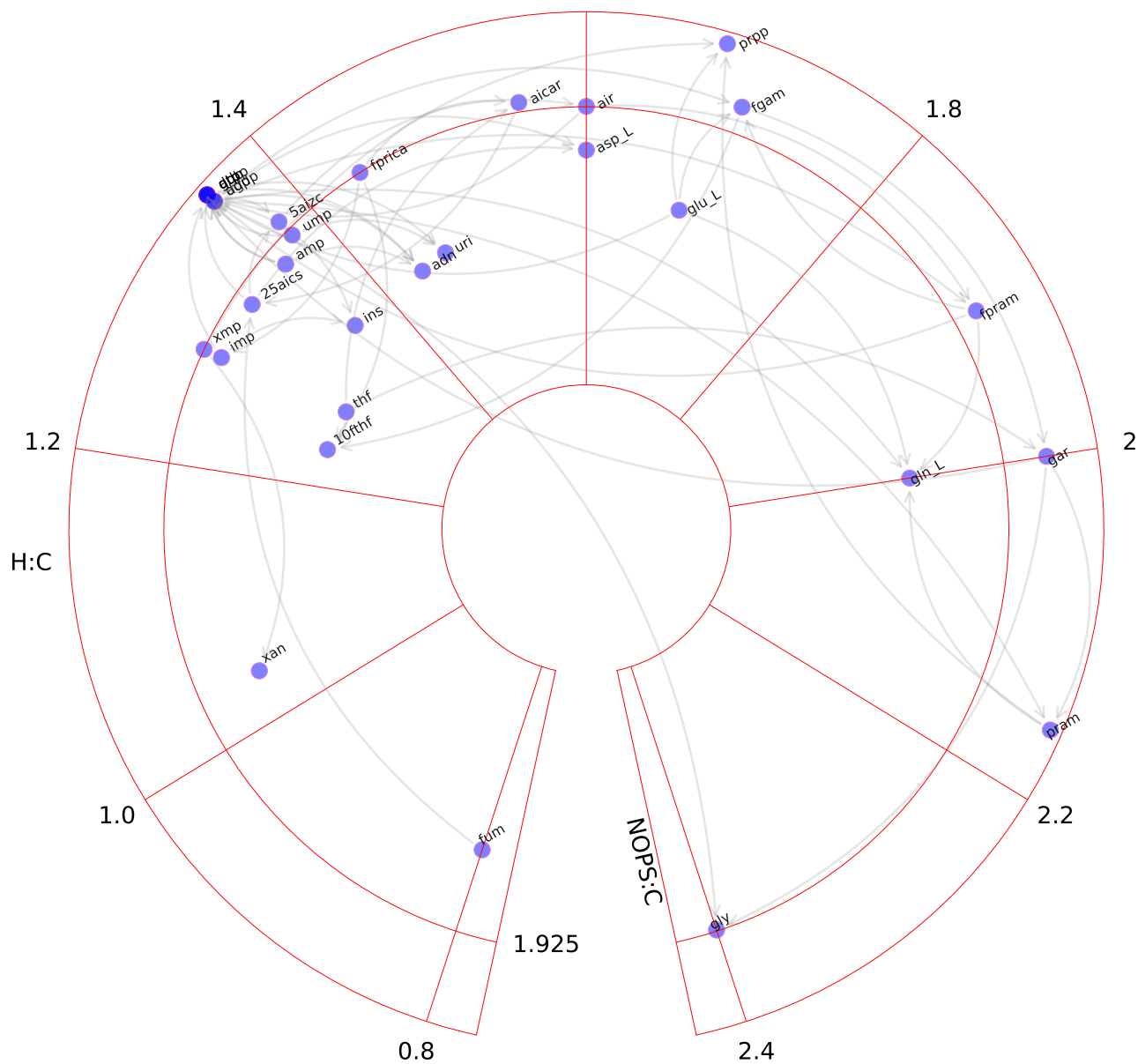

Supplement: Supplement 1 [file media-1.zip › Suppl_File_all_pathways/labeled/Purine synthesis.pdf]

# Hyaluronan metabolism

1.6

H:C

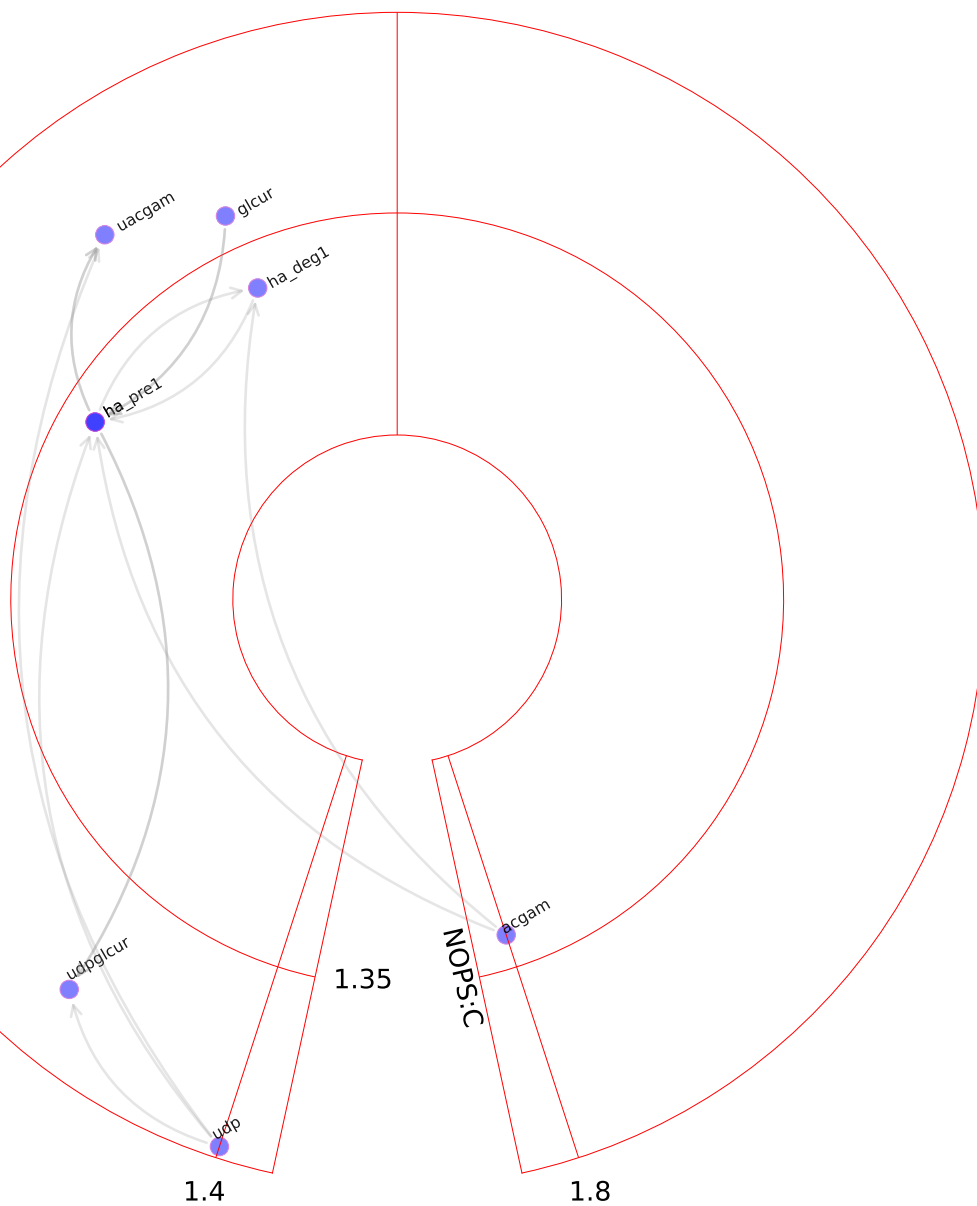

Supplement: Supplement 1 [file media-1.zip › Suppl_File_all_pathways/labeled/Hyaluronan metabolism.pdf]

# Vitamin B6 metabolism

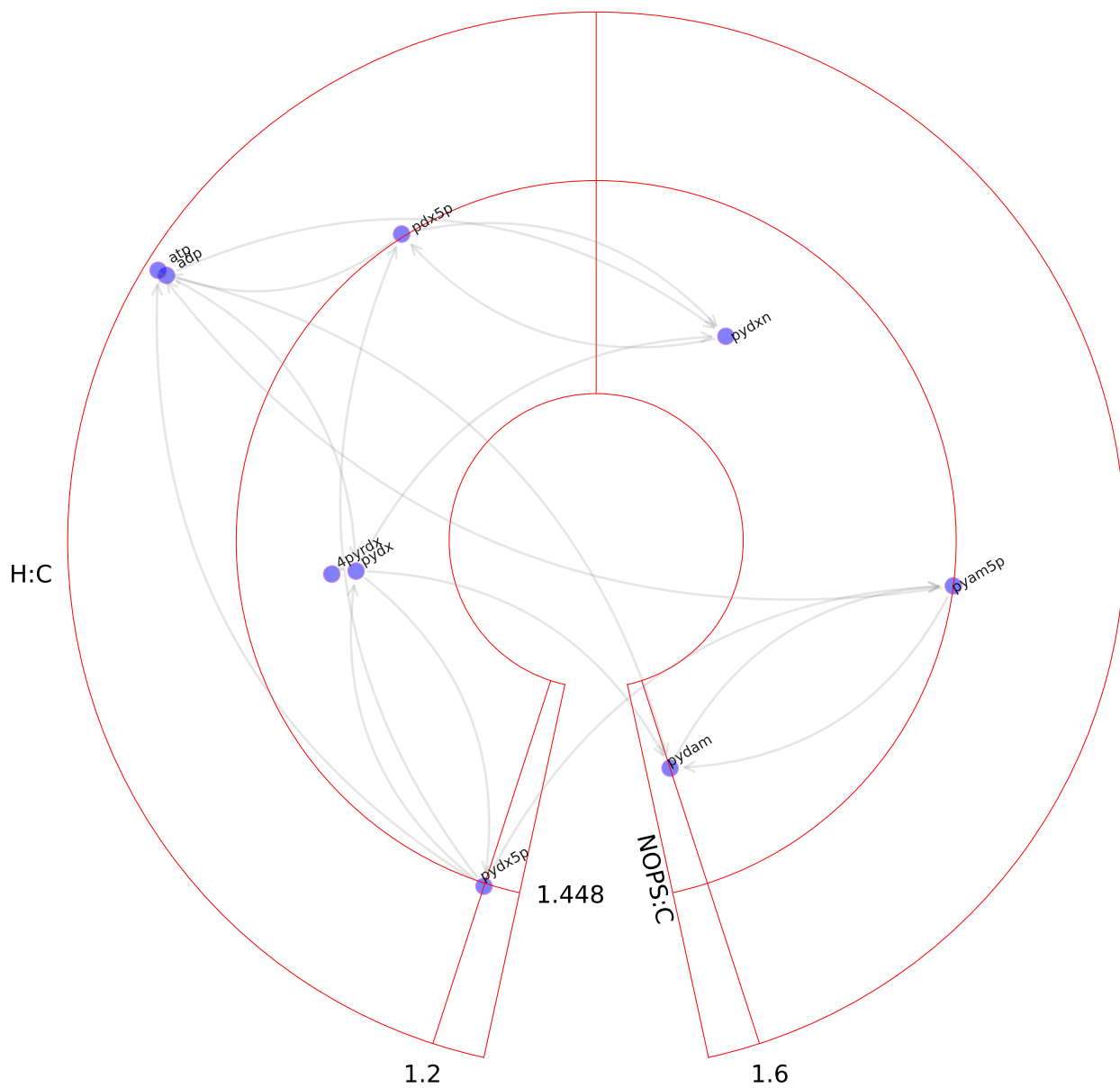

Supplement: Supplement 1 [file media-1.zip › Suppl_File_all_pathways/labeled/Vitamin B6 metabolism.pdf]

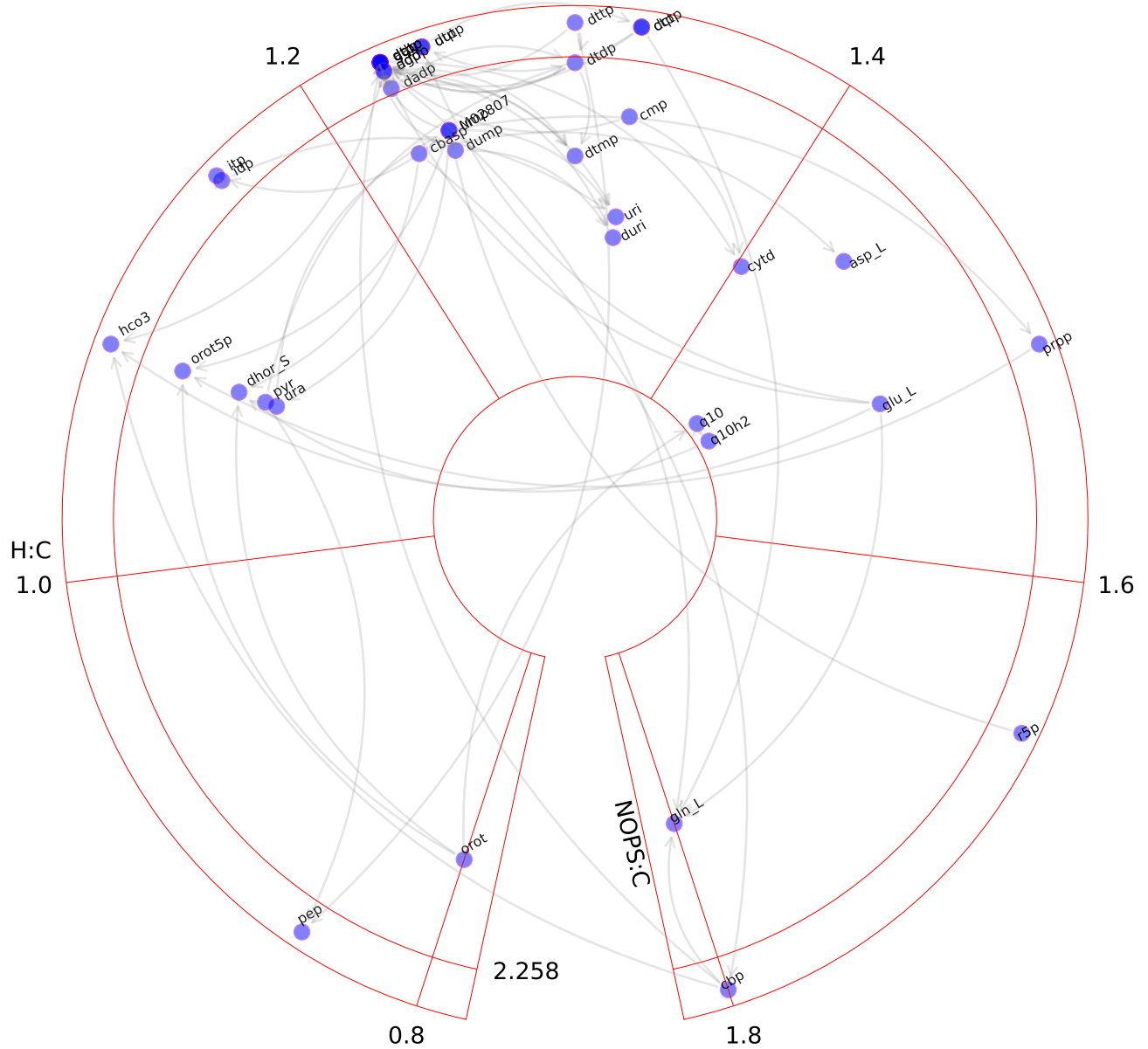

Supplement: Supplement 1 [file media-1.zip › Suppl_File_all_pathways/labeled/Pyrimidine synthesis.pdf]

## Vitamin E metabolism

1.4

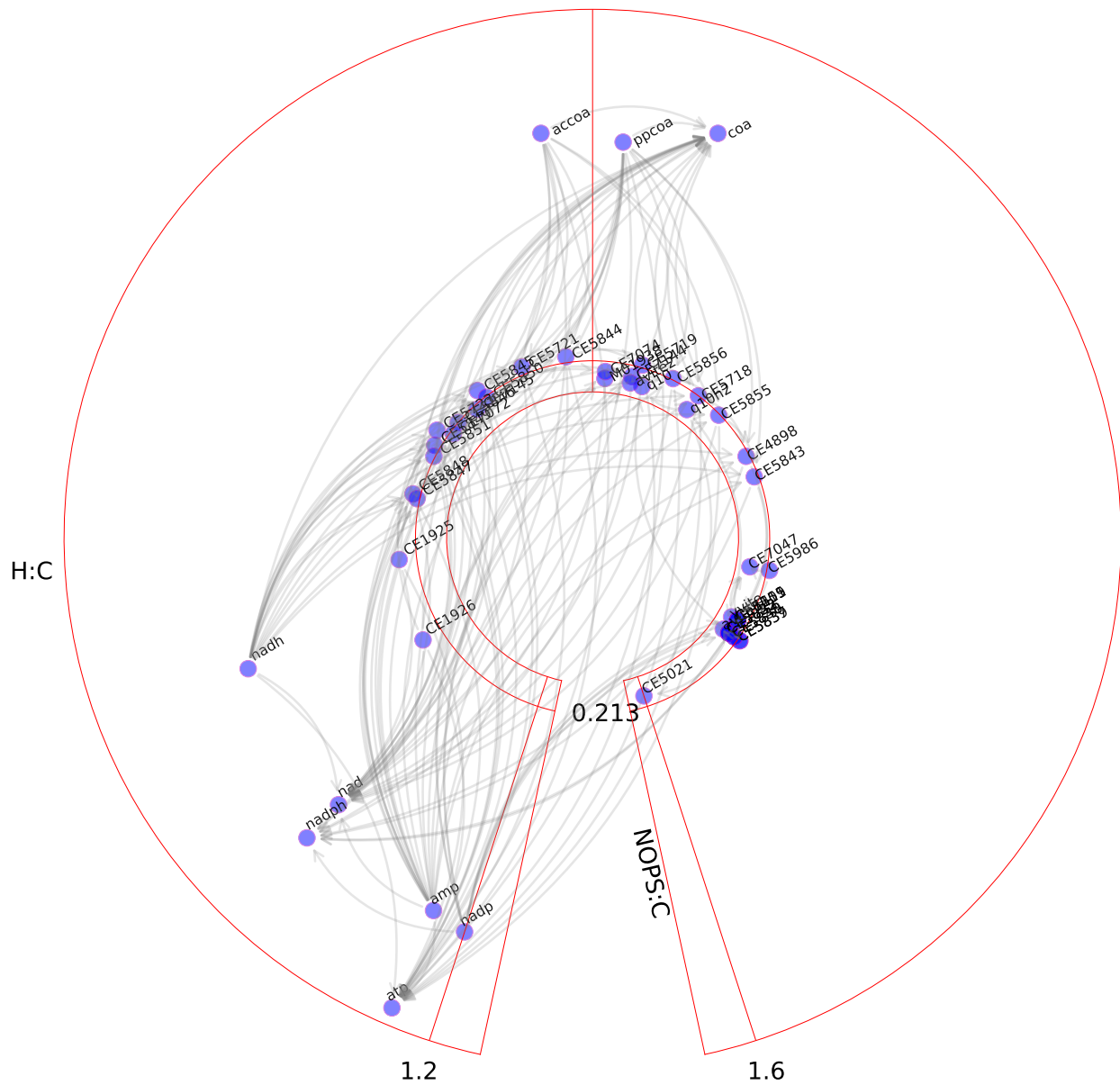

Supplement: Supplement 1 [file media-1.zip › Suppl_File_all_pathways/labeled/Vitamin E metabolism.pdf]

# Aminosugar metabolism

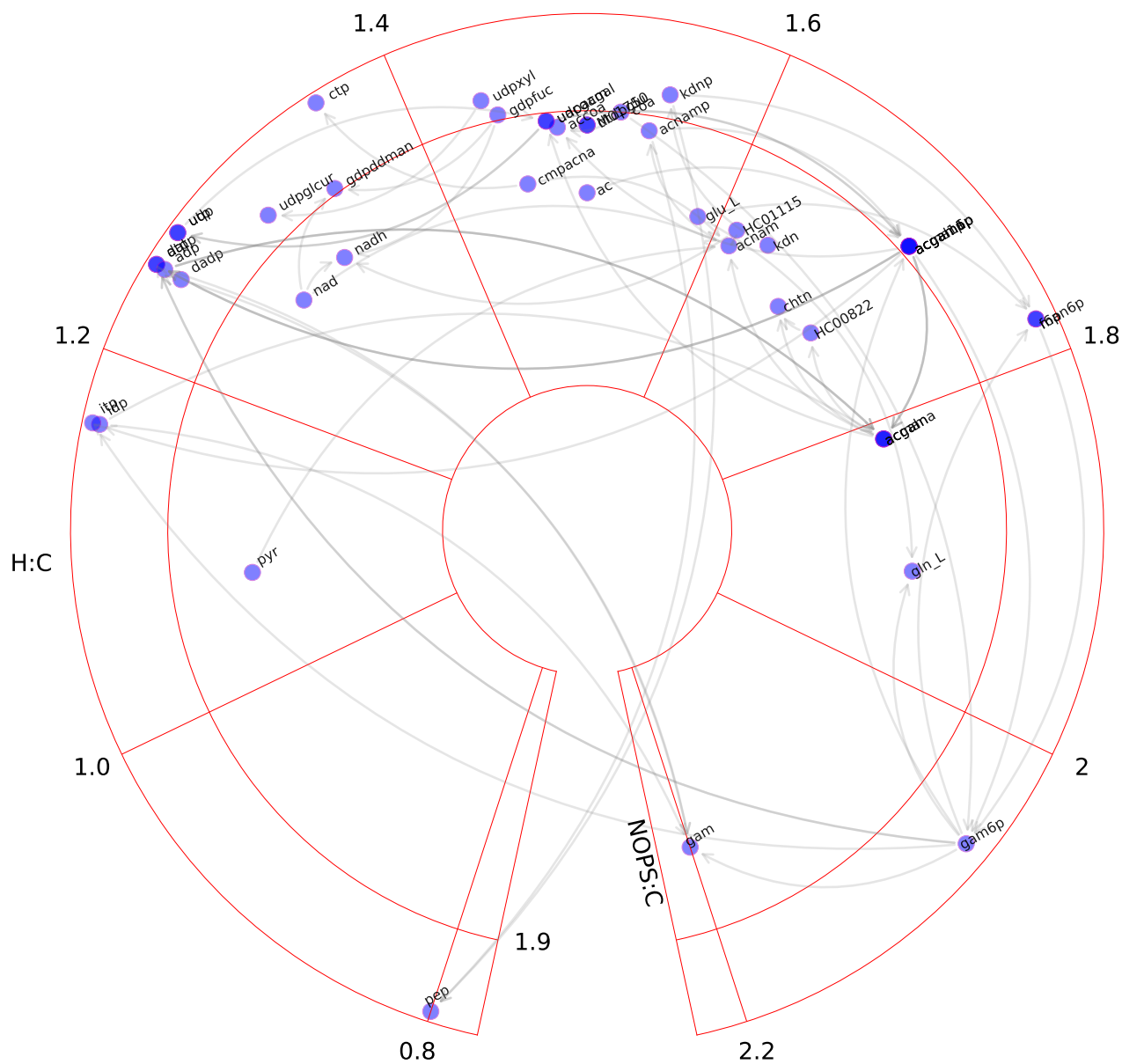

Supplement: Supplement 1 [file media-1.zip › Suppl_File_all_pathways/labeled/Aminosugar metabolism.pdf]

# ROS detoxification

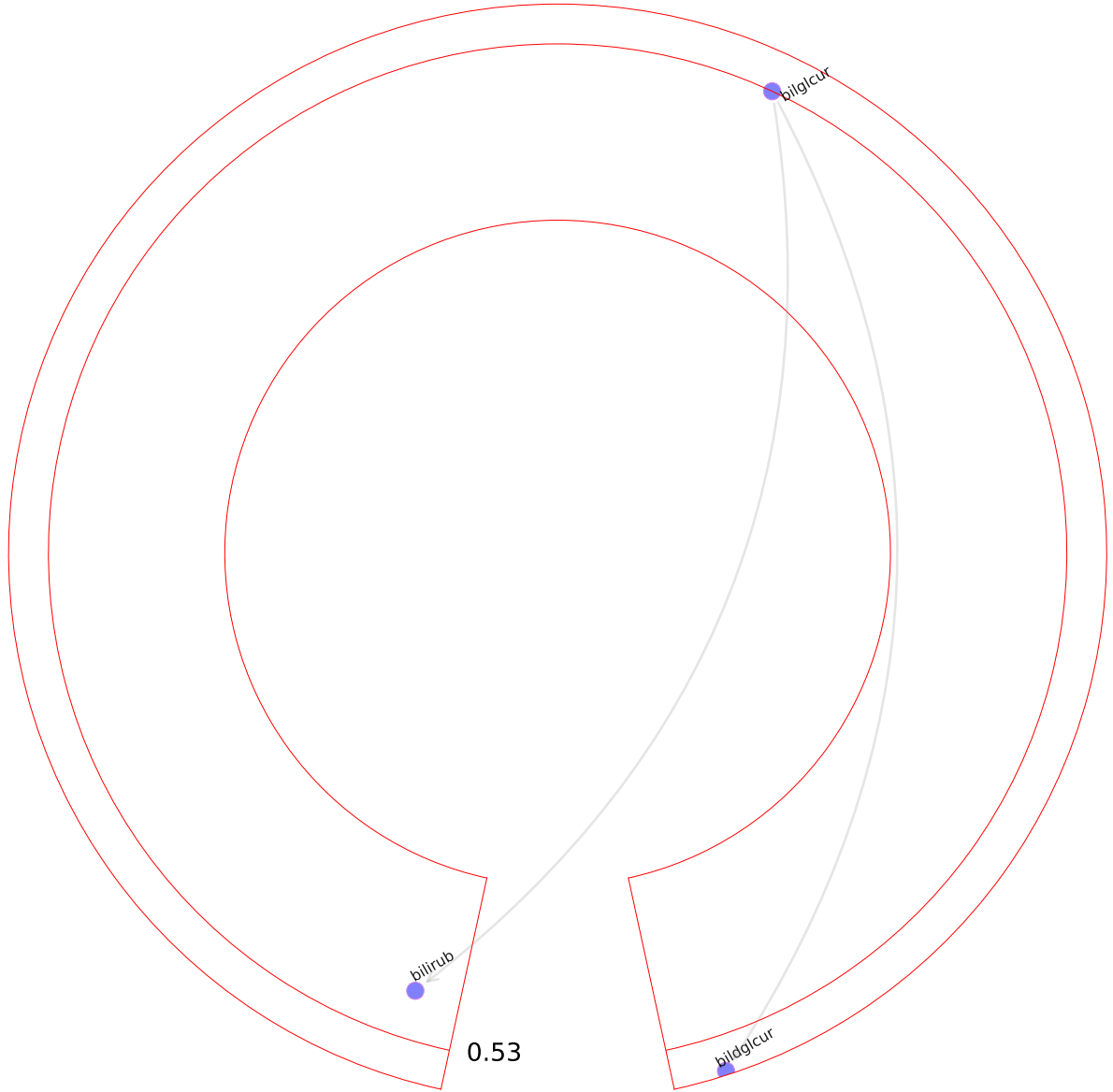

Supplement: Supplement 1 [file media-1.zip › Suppl_File_all_pathways/labeled/ROS detoxification.pdf]

## Heparan sulfate degradation

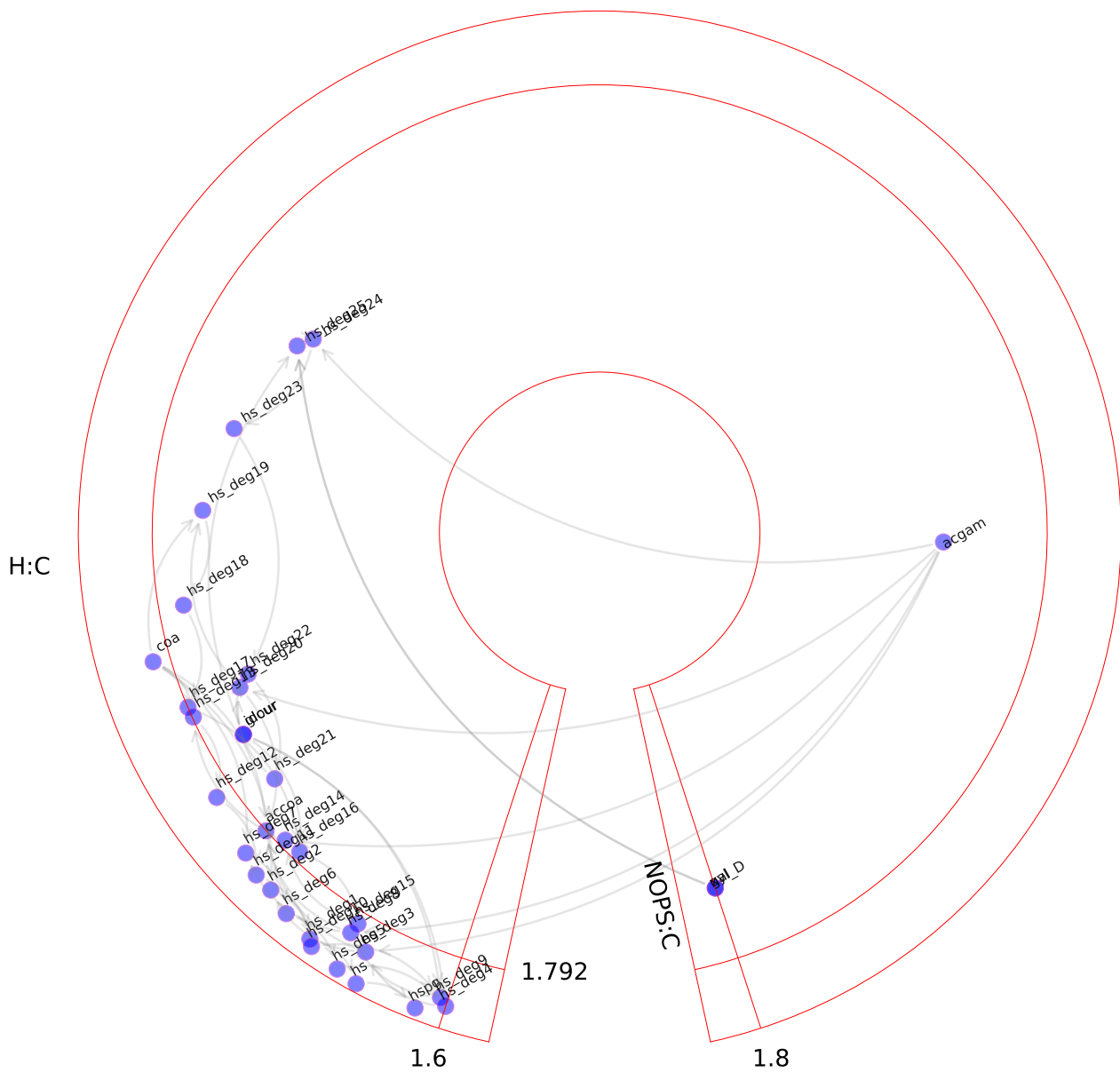

Supplement: Supplement 1 [file media-1.zip › Suppl_File_all_pathways/labeled/Heparan sulfate degradation.pdf]

Oxidative phosphorylation

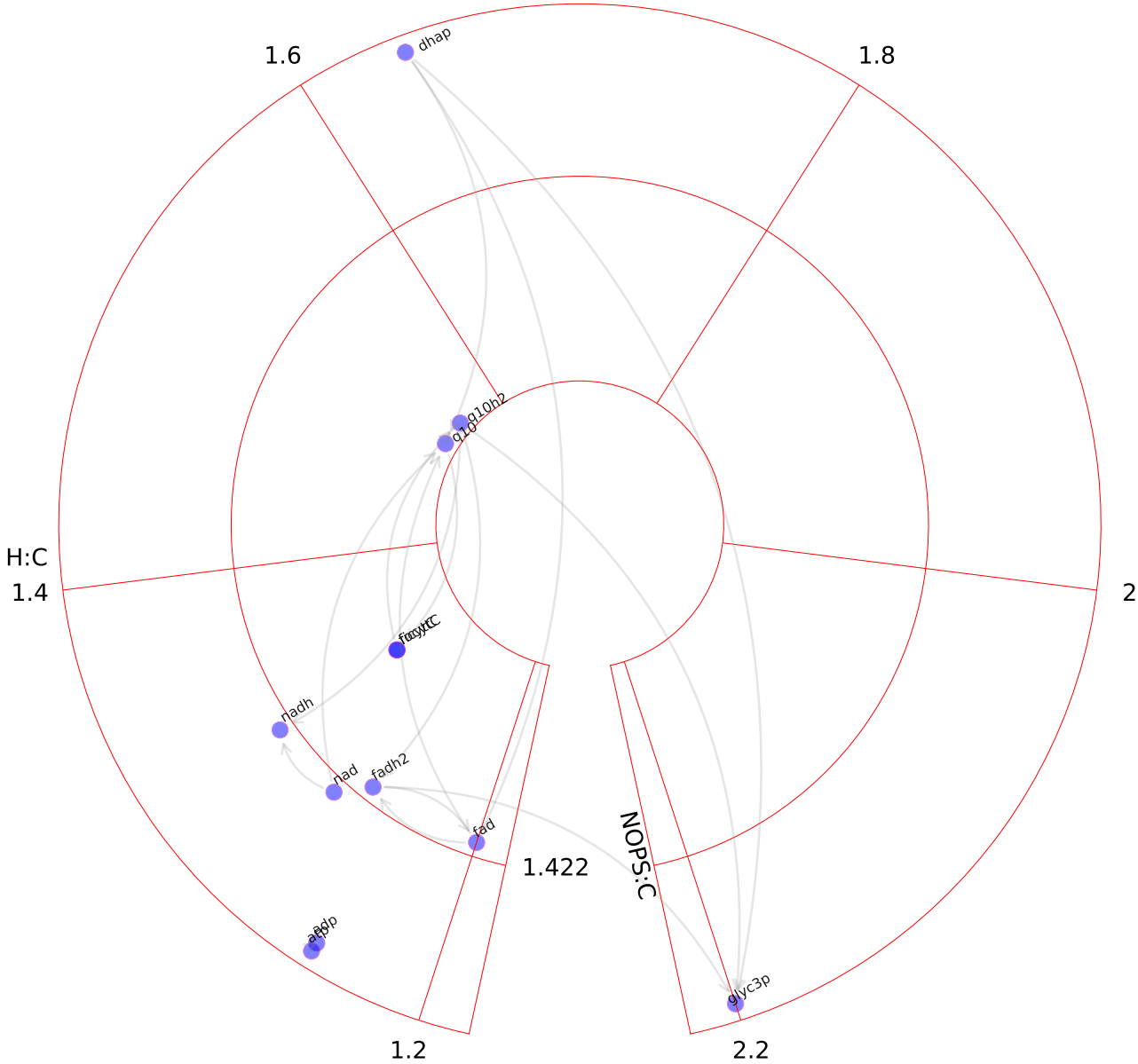

Supplement: Supplement 1 [file media-1.zip › Suppl_File_all_pathways/labeled/Oxidative phosphorylation.pdf]

## Phosphatidylinositol phosphate metabolism

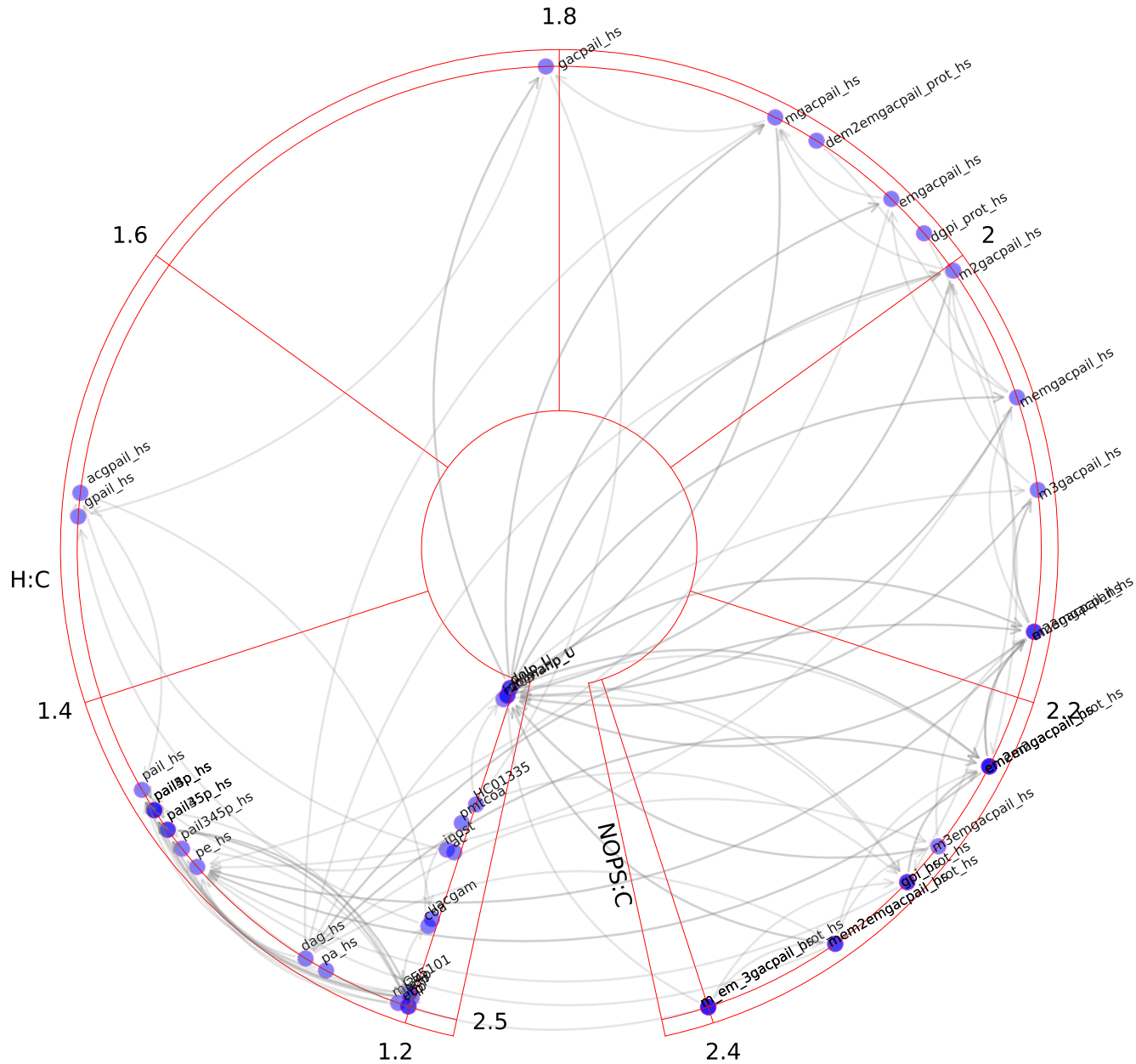

Supplement: Supplement 1 [file media-1.zip › Suppl_File_all_pathways/labeled/Phosphatidylinositol phosphate metabolism.pdf]

## 1.6

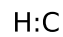

Supplement: Supplement 1 [file media-1.zip › Suppl_File_all_pathways/labeled/Glutamate metabolism.pdf]

## Beta-Alanine metabolism

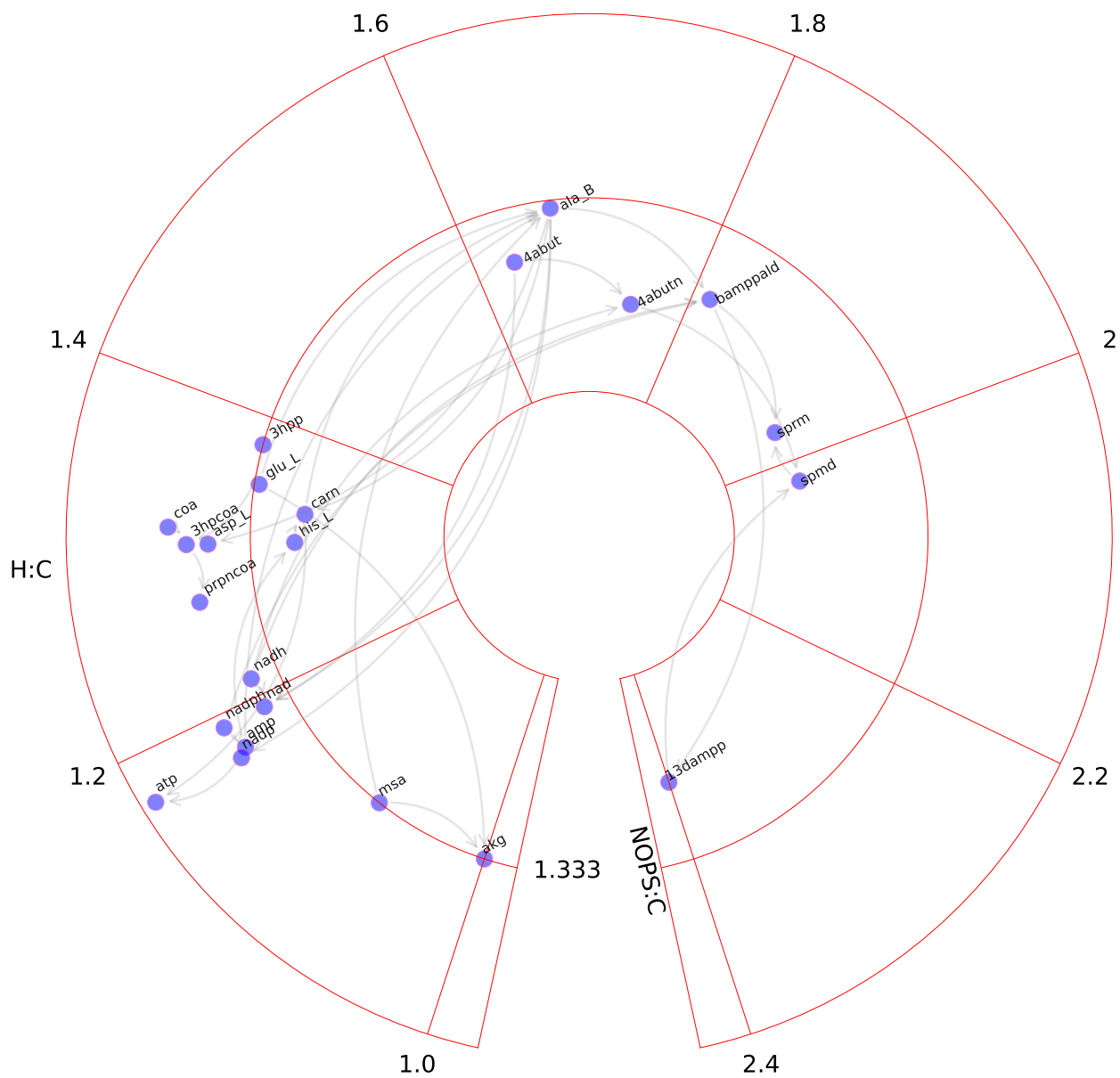

Supplement: Supplement 1 [file media-1.zip › Suppl_File_all_pathways/labeled/Beta-Alanine metabolism.pdf]

# Histidine metabolism

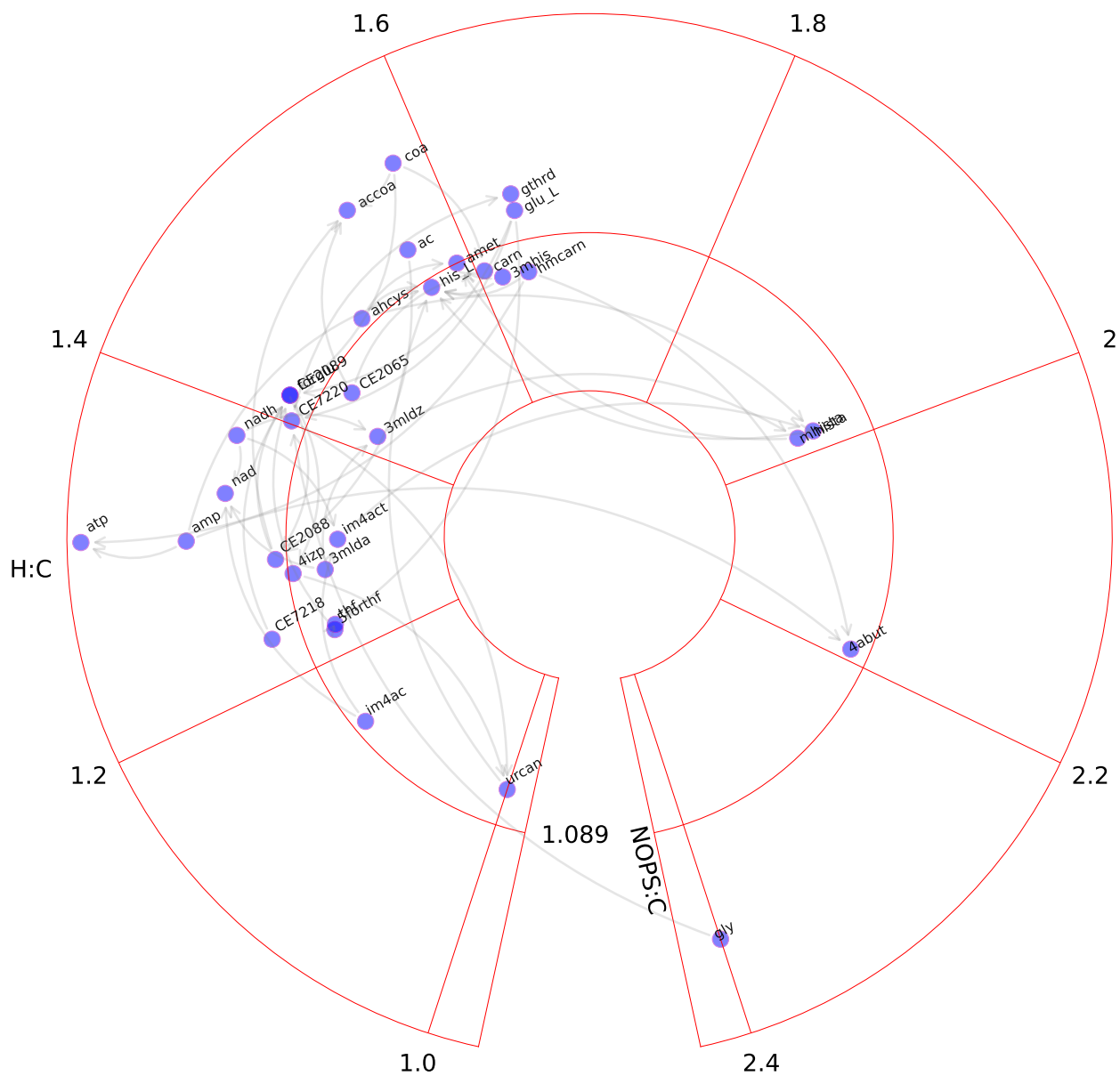

Supplement: Supplement 1 [file media-1.zip › Suppl_File_all_pathways/labeled/Histidine metabolism.pdf]

## Aminoacyl-tRNA biosynthesis

1.8

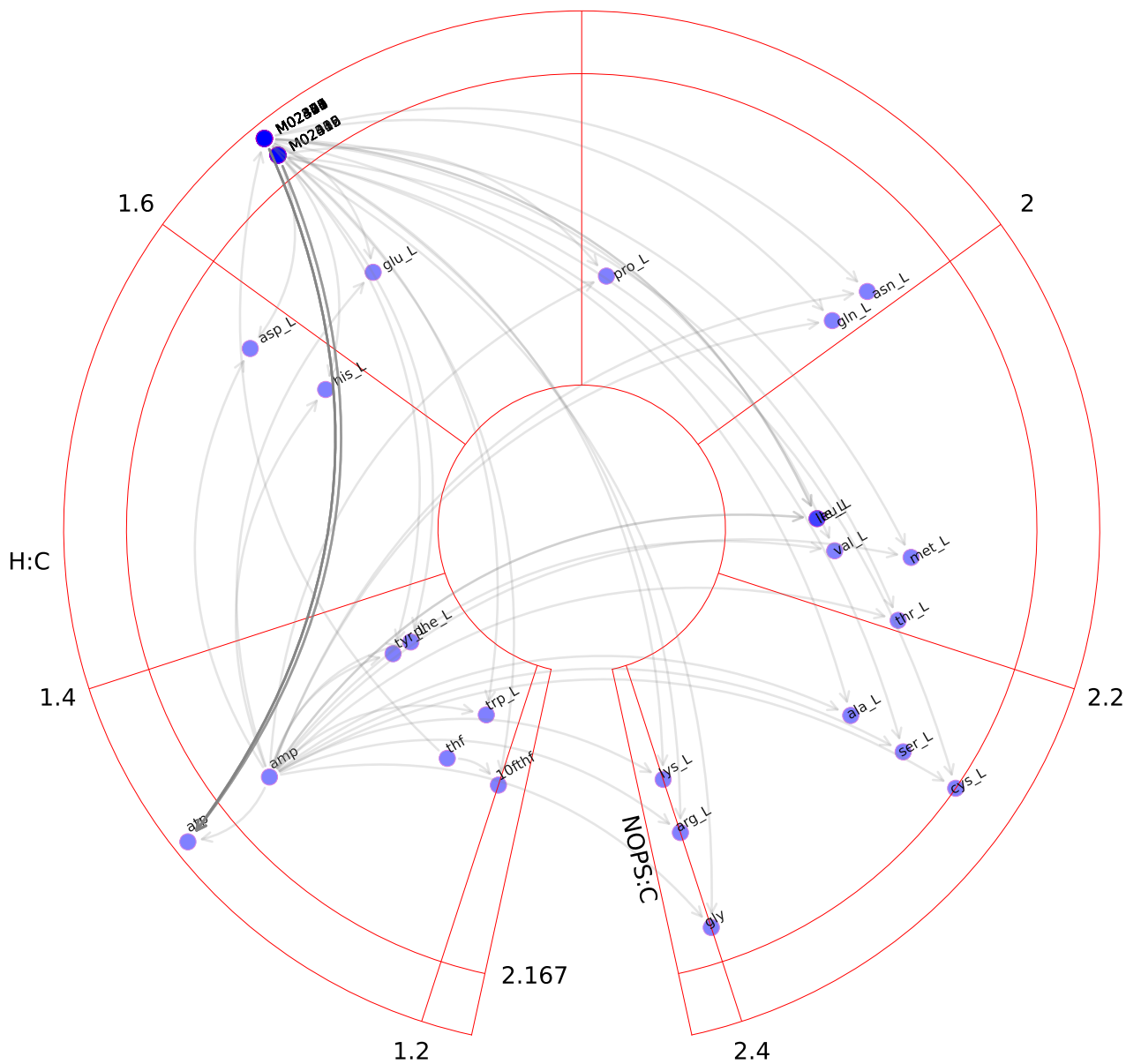

Supplement: Supplement 1 [file media-1.zip › Suppl_File_all_pathways/labeled/Aminoacyl-tRNA biosynthesis.pdf]

# D-alanine metabolism

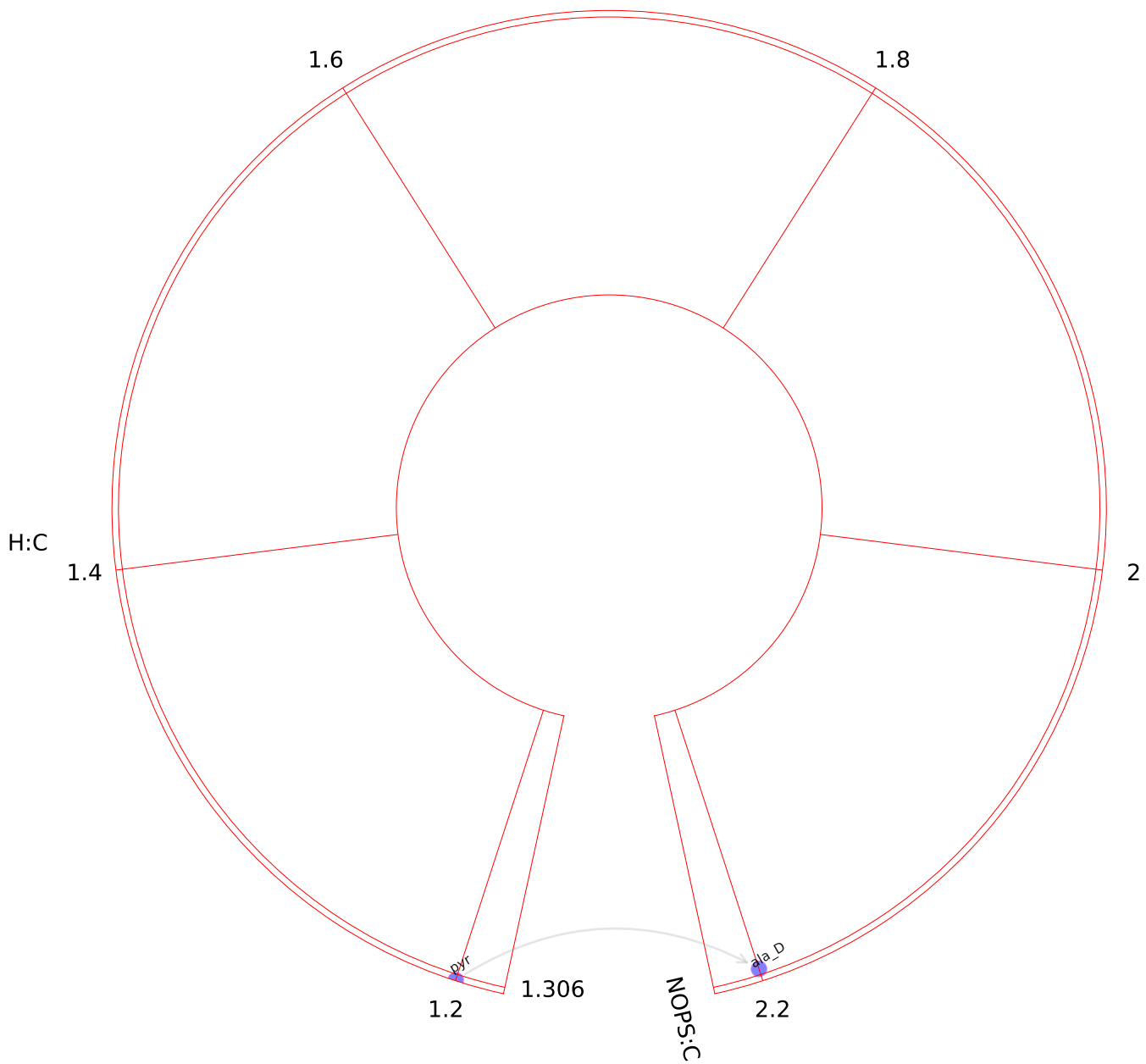

Supplement: Supplement 1 [file media-1.zip › Suppl_File_all_pathways/labeled/D-alanine metabolism.pdf]

## CoA synthesis

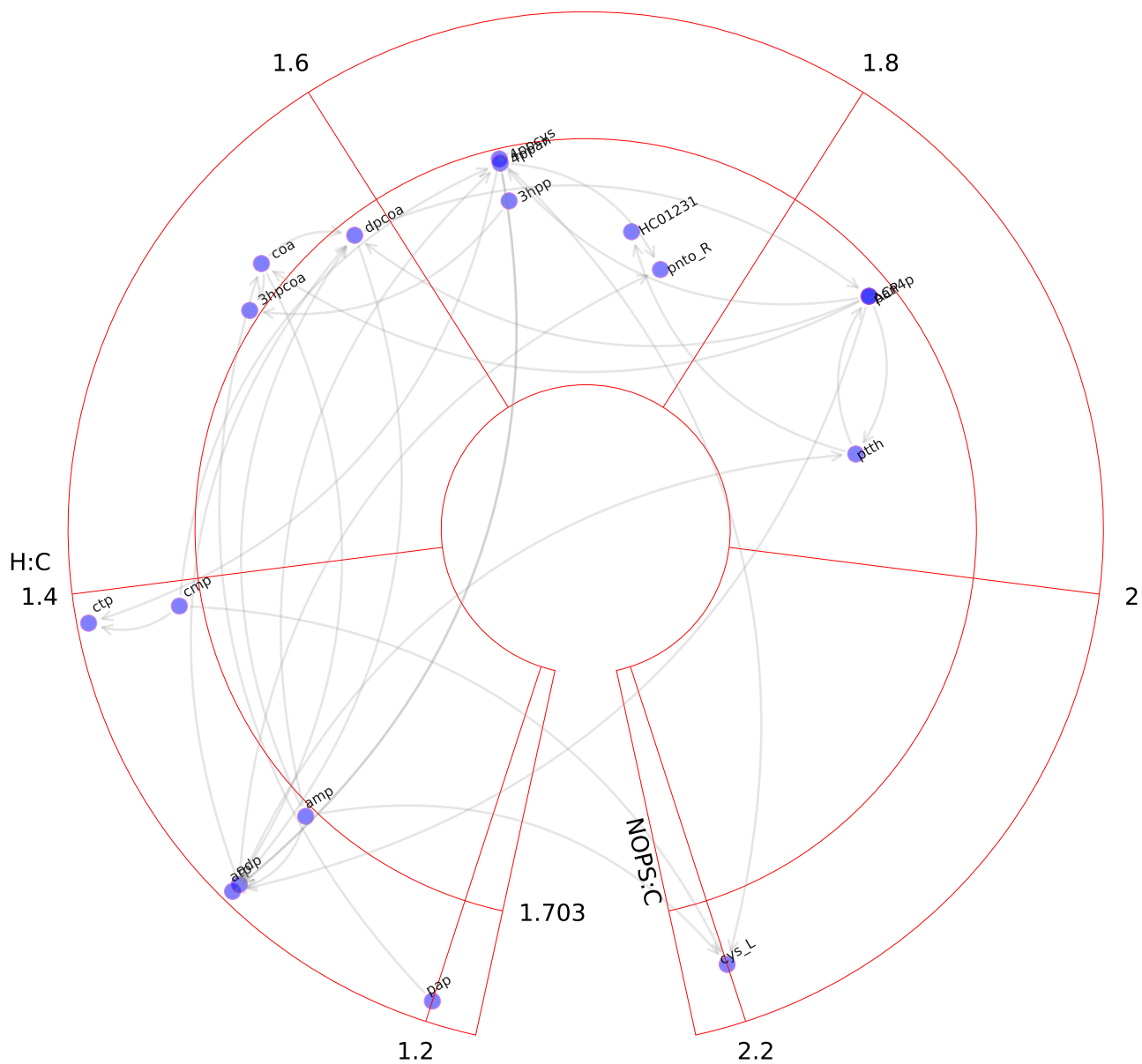

Supplement: Supplement 1 [file media-1.zip › Suppl_File_all_pathways/labeled/CoA synthesis.pdf]

# Linoleate metabolism

1.6

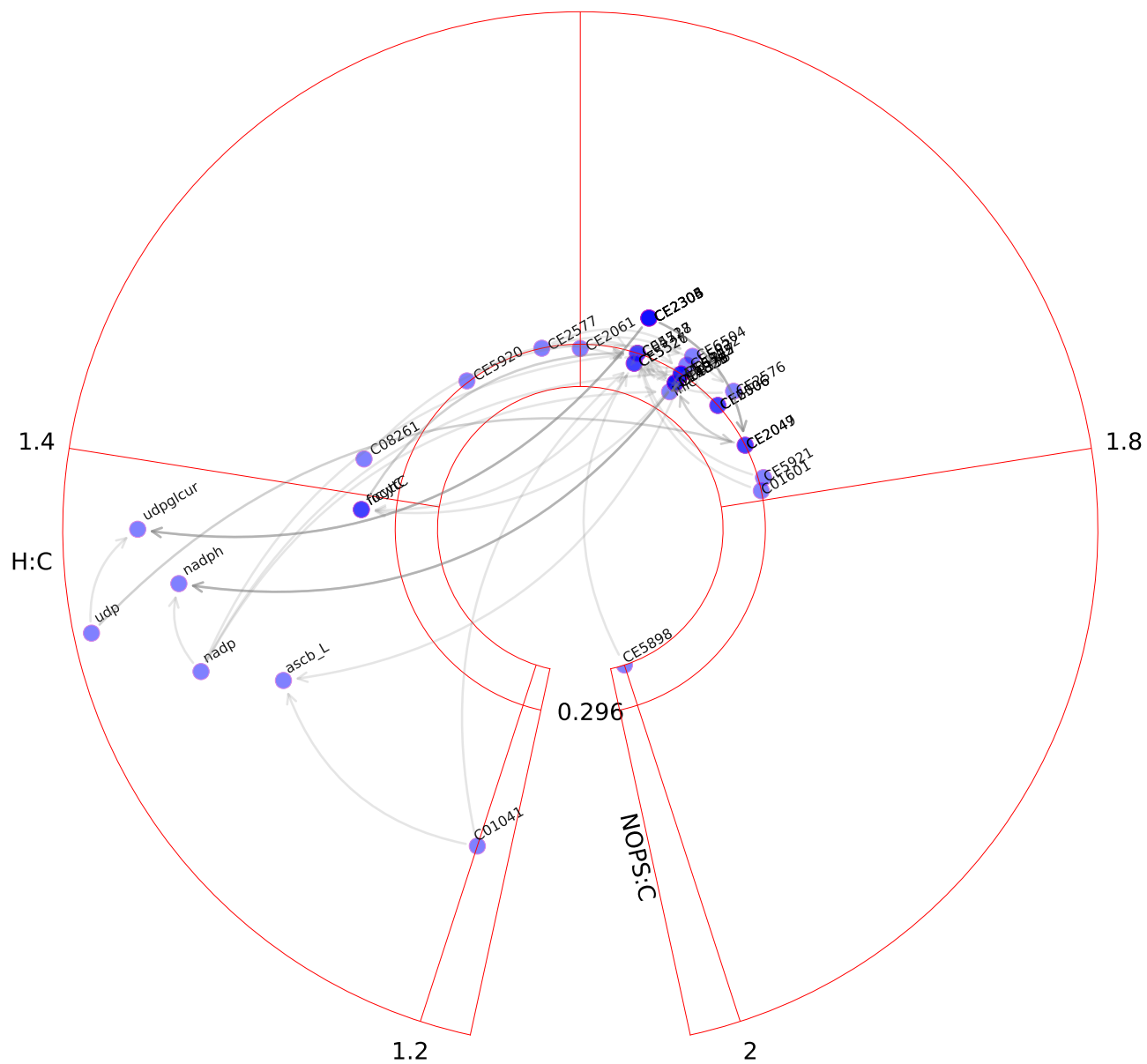

Supplement: Supplement 1 [file media-1.zip › Suppl_File_all_pathways/labeled/Linoleate metabolism.pdf]

# Keratan sulfate synthesis

1.4

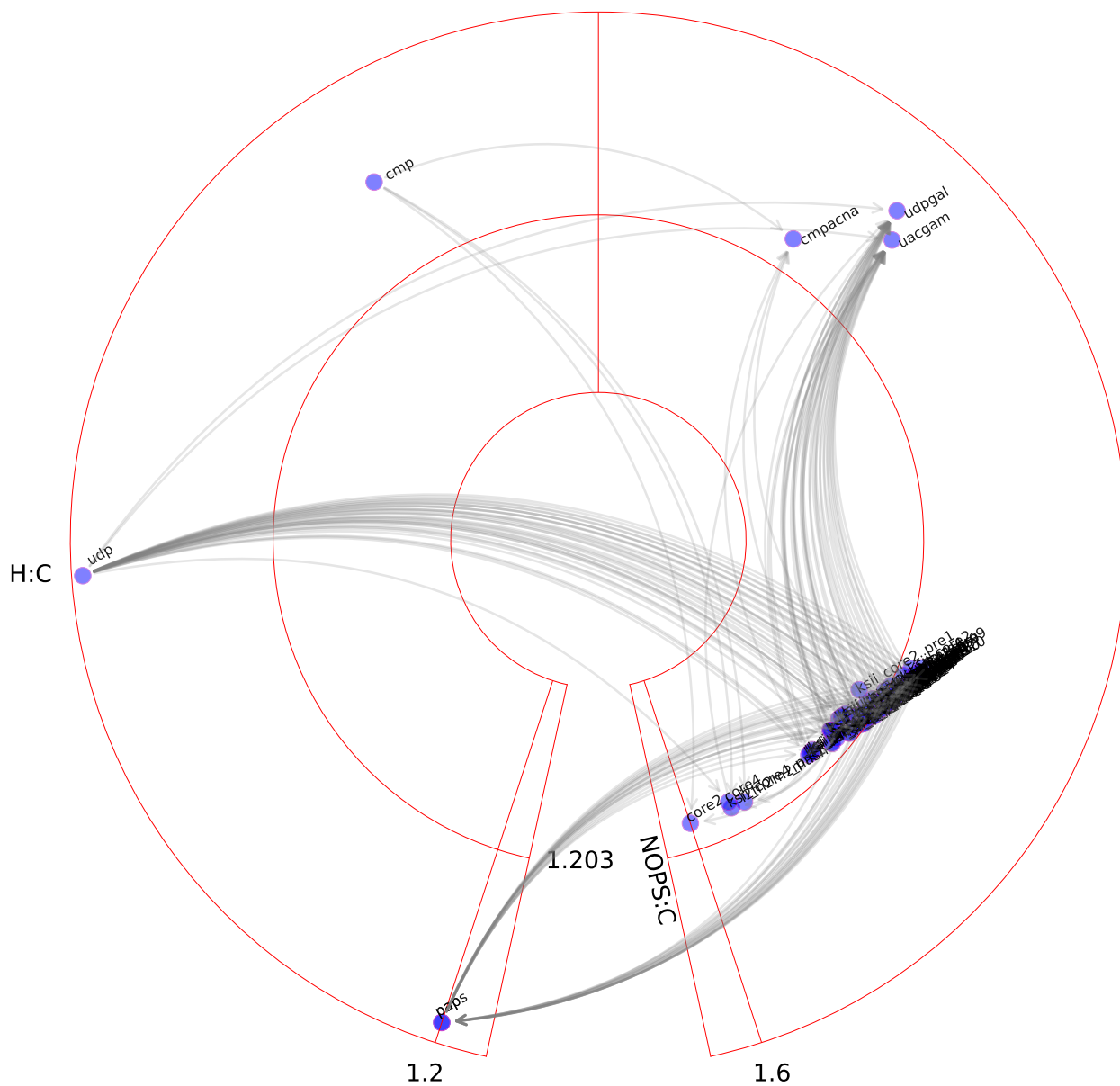

Supplement: Supplement 1 [file media-1.zip › Suppl_File_all_pathways/labeled/Keratan sulfate synthesis.pdf]

# Heme synthesis

1.2

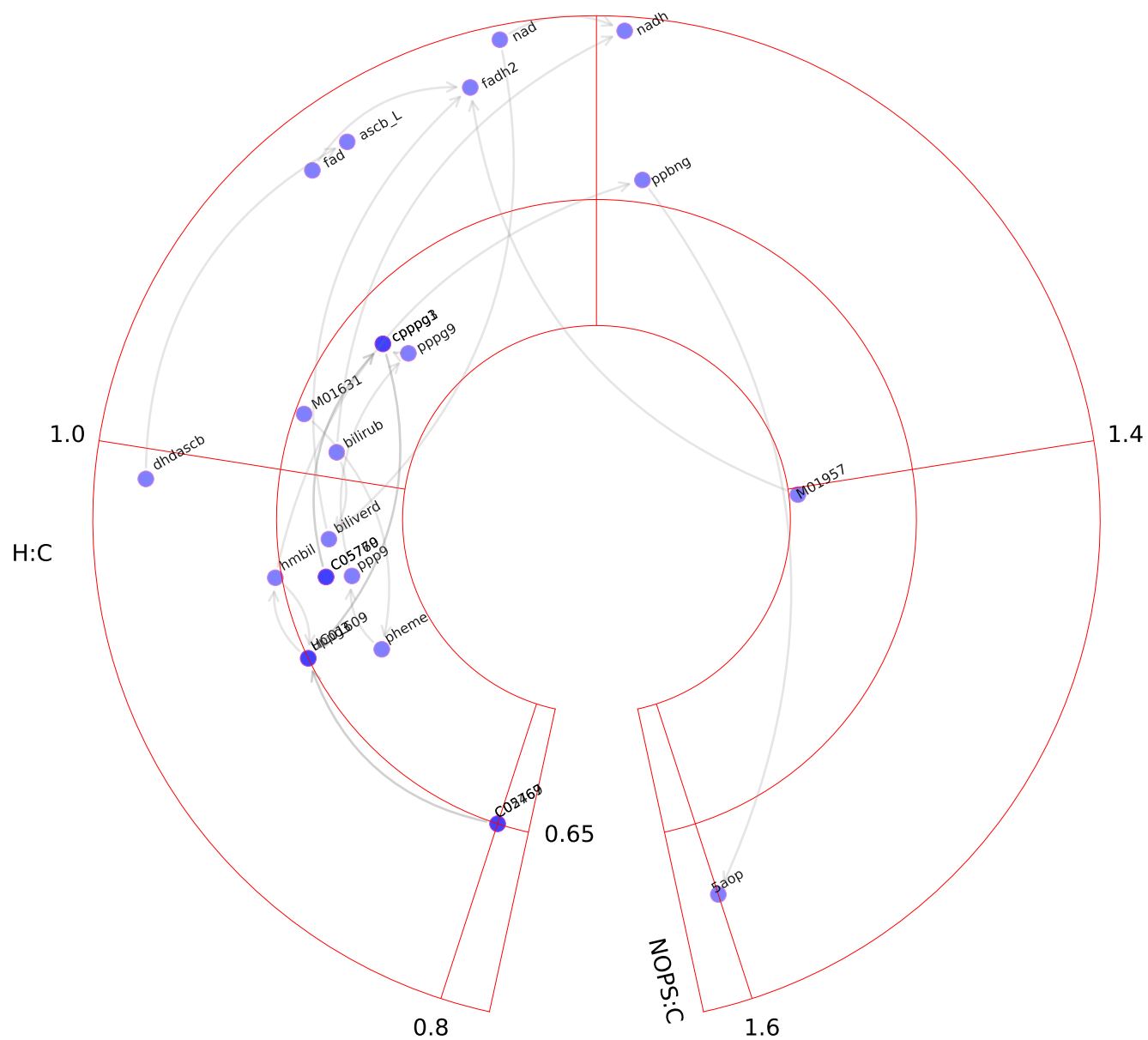

Supplement: Supplement 1 [file media-1.zip › Suppl_File_all_pathways/labeled/Heme synthesis.pdf]

## 1.6

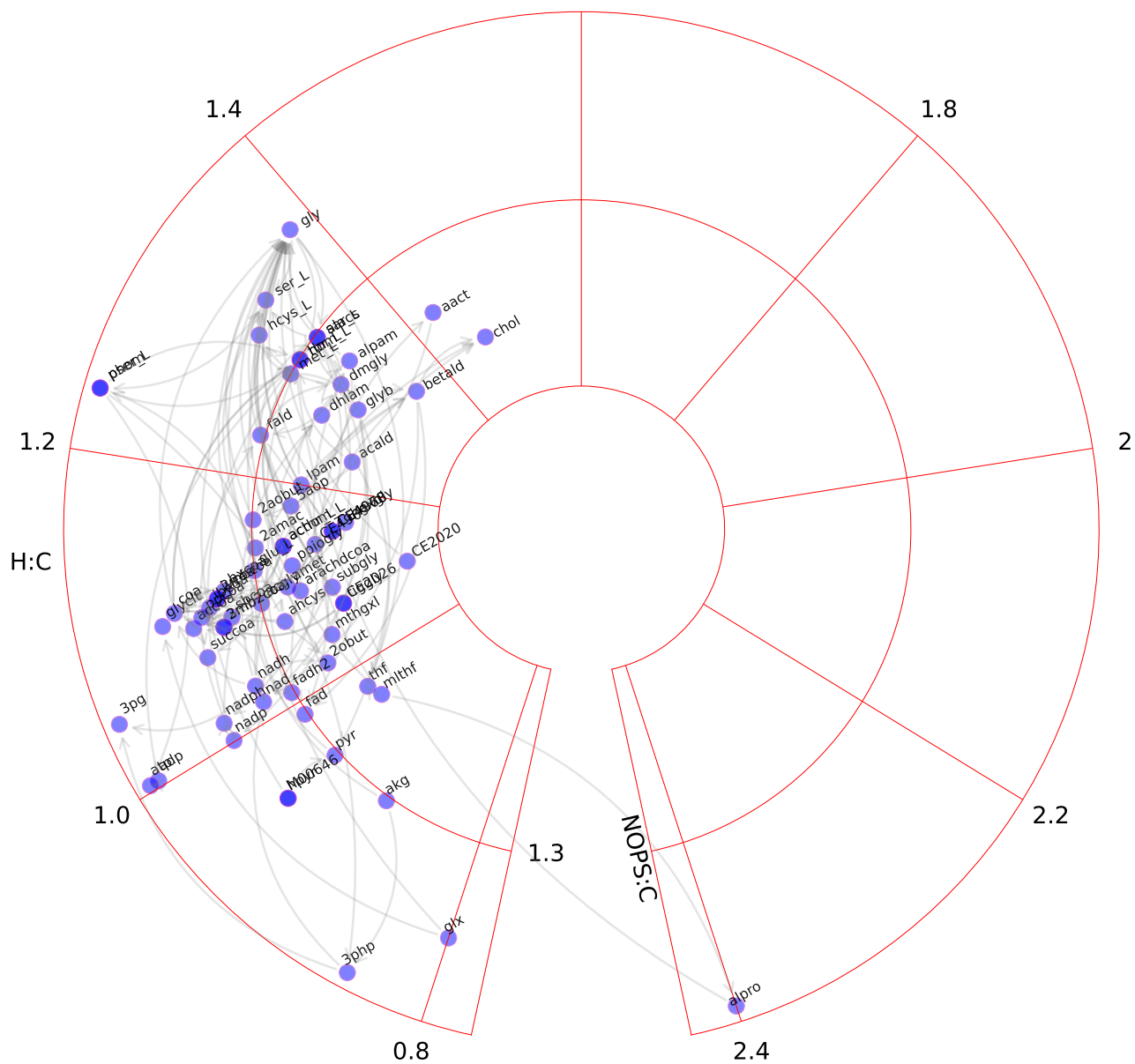

Supplement: Supplement 1 [file media-1.zip › Suppl_File_all_pathways/labeled/Glycine, serine, alanine, and threonine metabolism.pdf]

## Ubiquinone synthesis

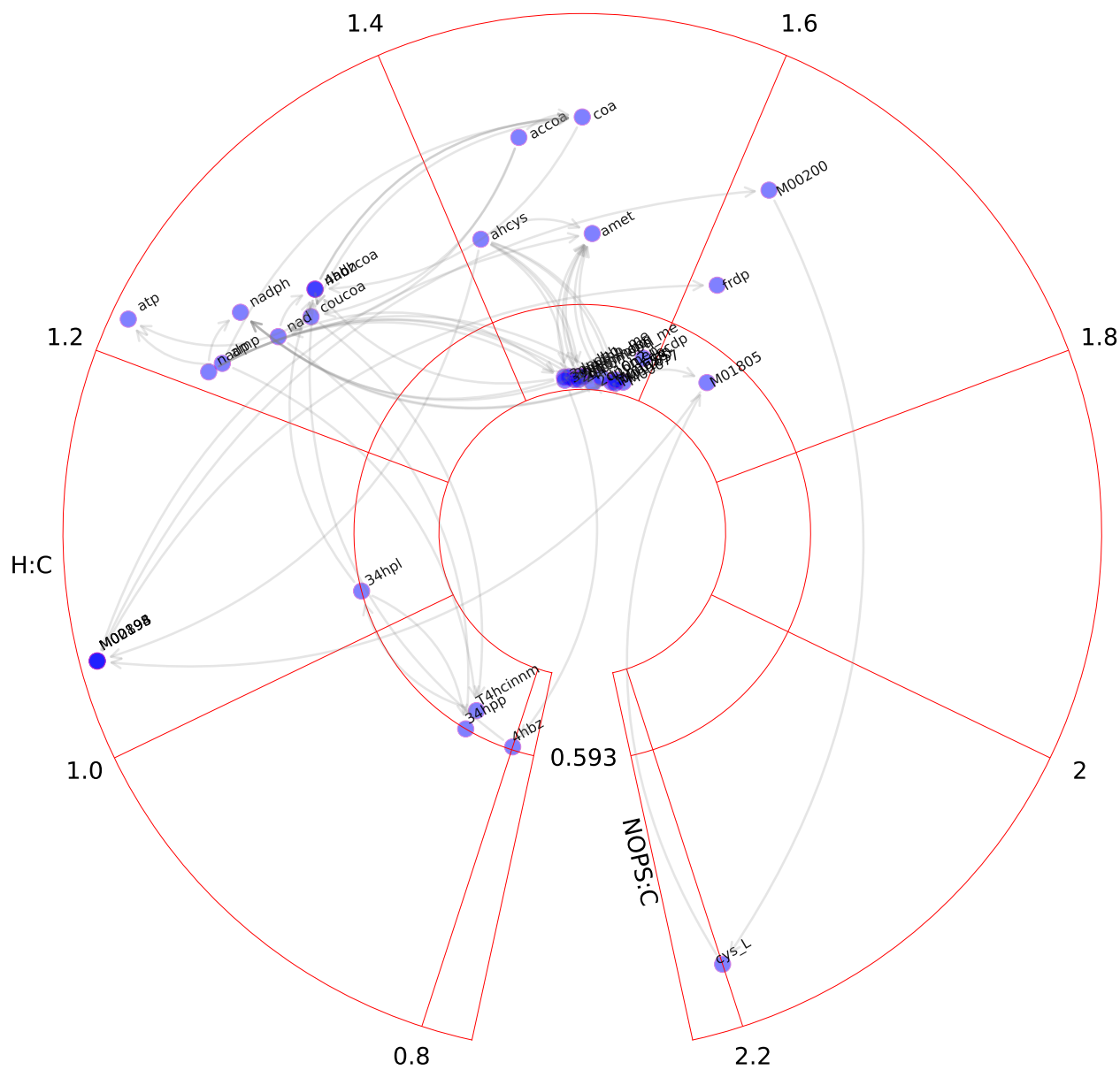

Supplement: Supplement 1 [file media-1.zip › Suppl_File_all_pathways/labeled/Ubiquinone synthesis.pdf]

# NAD metabolism

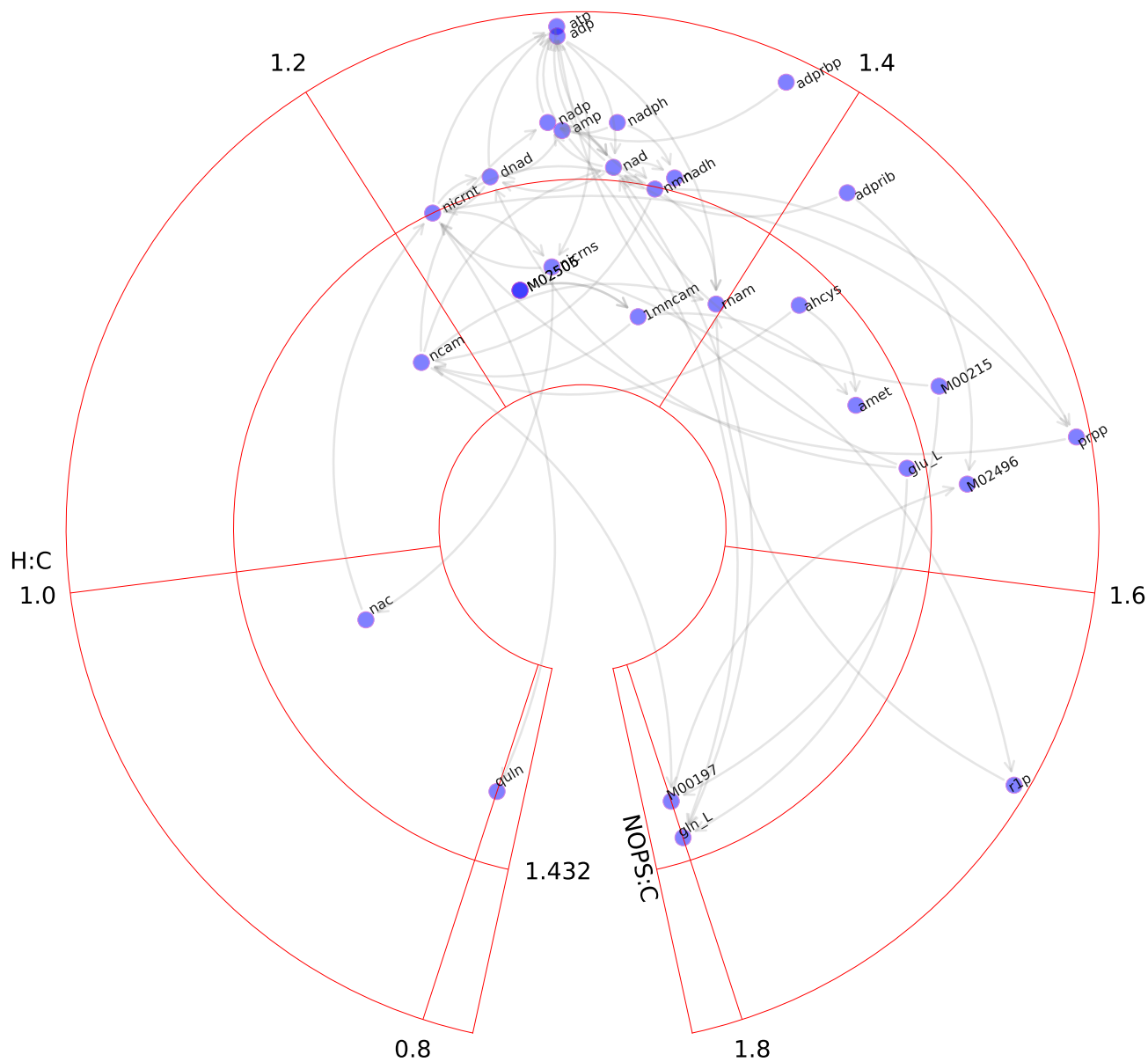

Supplement: Supplement 1 [file media-1.zip › Suppl_File_all_pathways/labeled/NAD metabolism.pdf]

# Stilbene, coumarine and lignin synthesis

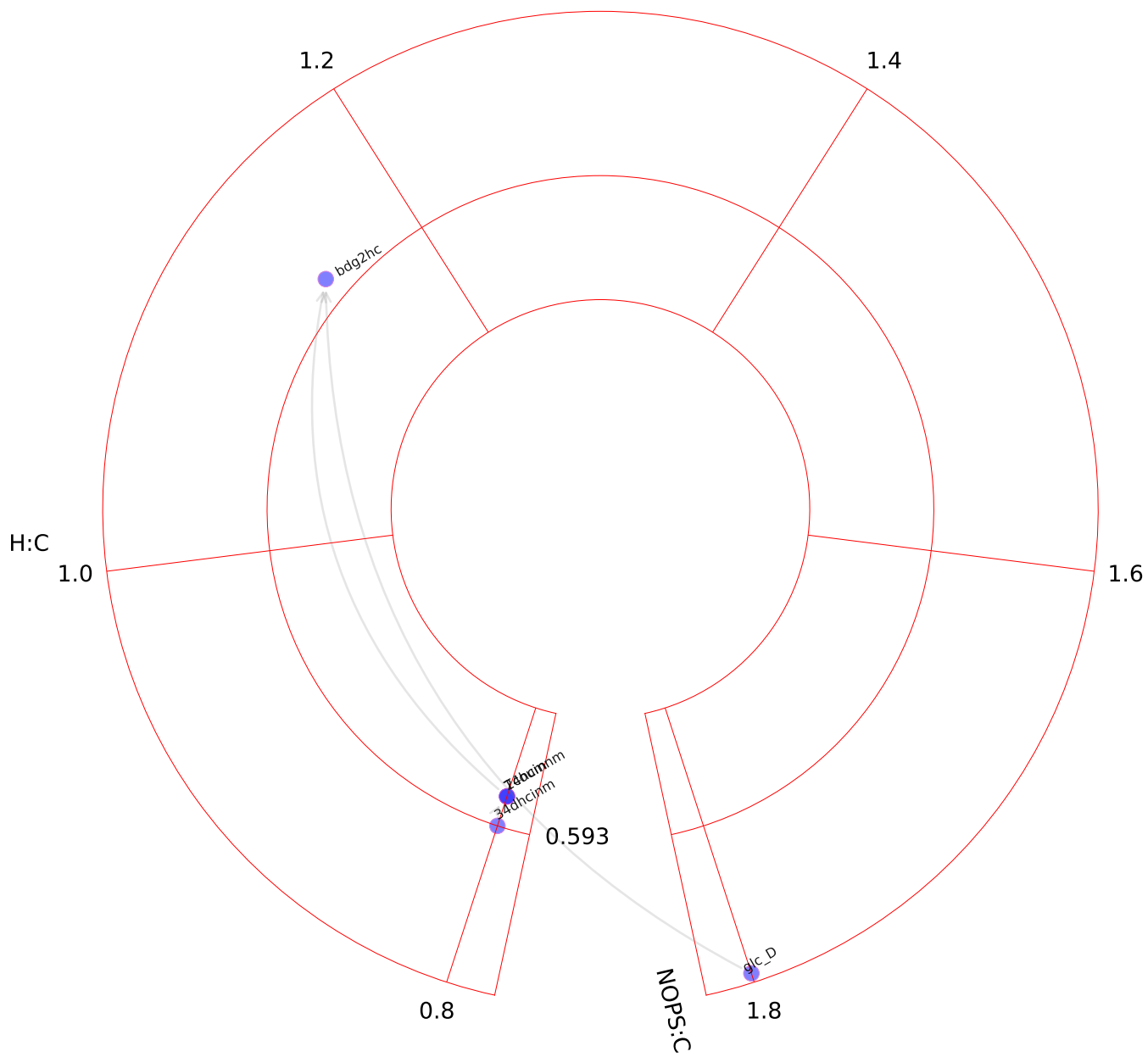

Supplement: Supplement 1 [file media-1.zip › Suppl_File_all_pathways/labeled/Stilbene, coumarine and lignin synthesis.pdf]

# Triglycerides formation

2

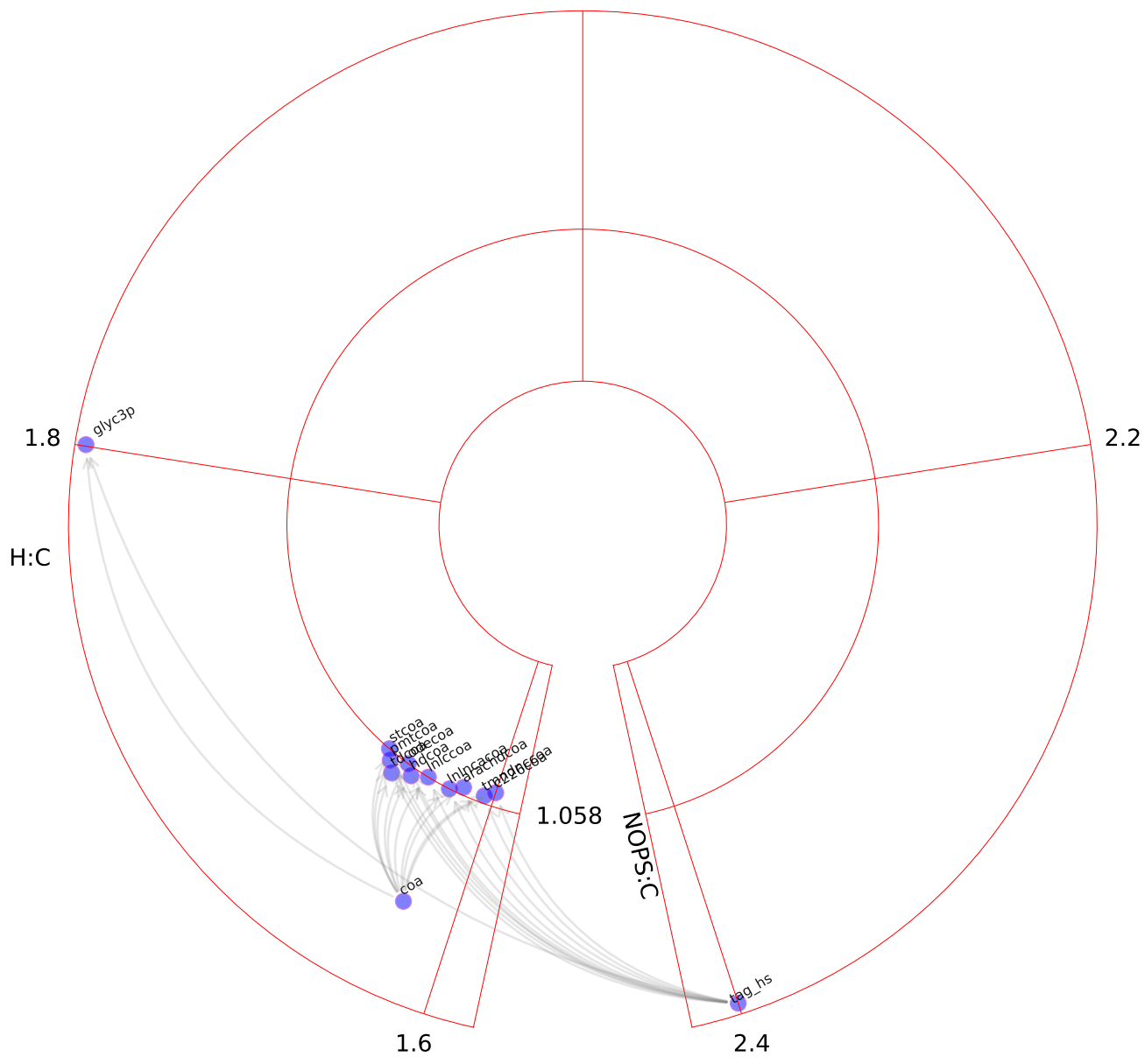

Supplement: Supplement 1 [file media-1.zip › Suppl_File_all_pathways/labeled/Triglycerides formation.pdf]

# Tyrosine metabolism

1.6

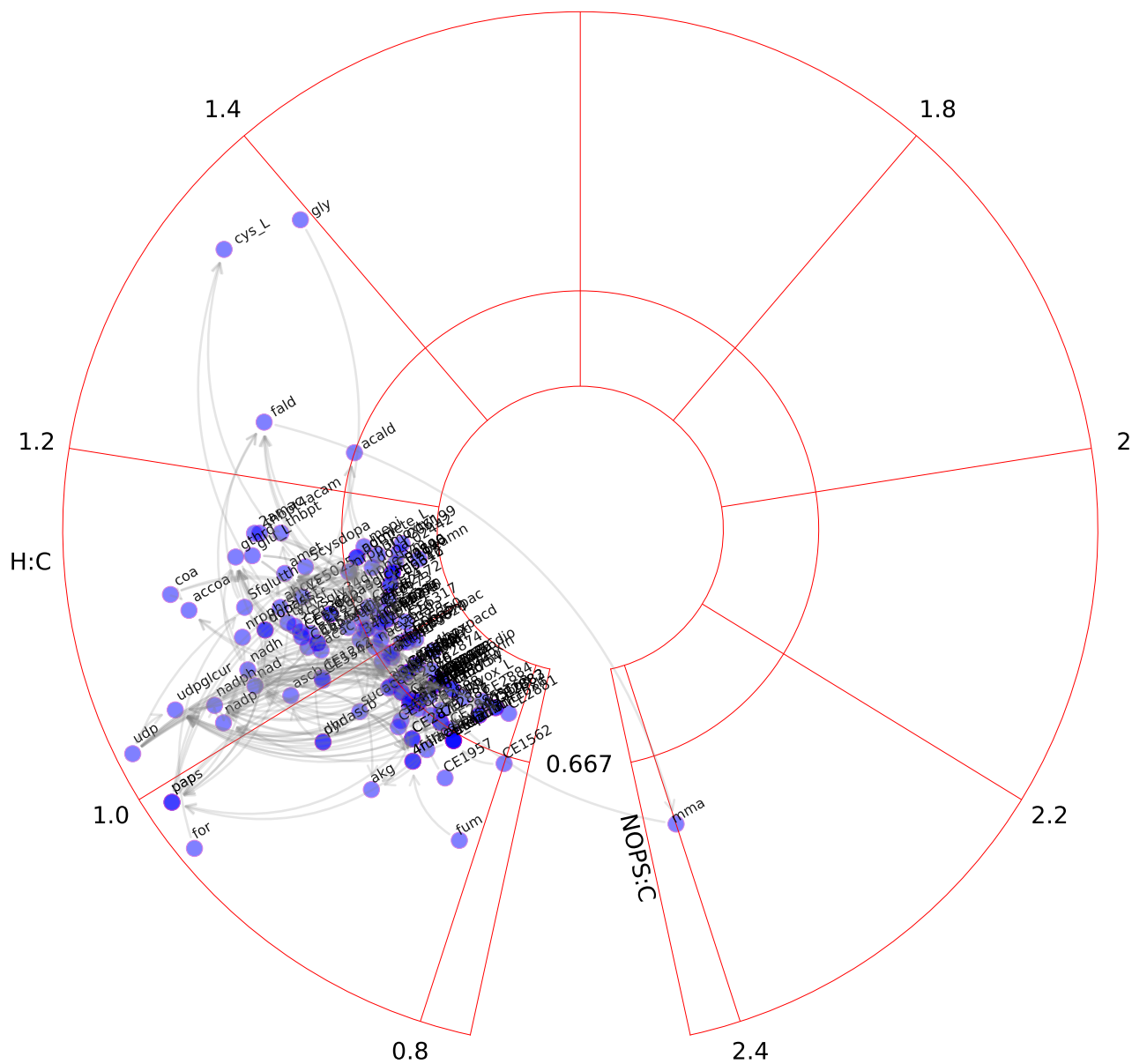

Supplement: Supplement 1 [file media-1.zip › Suppl_File_all_pathways/labeled/Tyrosine metabolism.pdf]

## 1.6

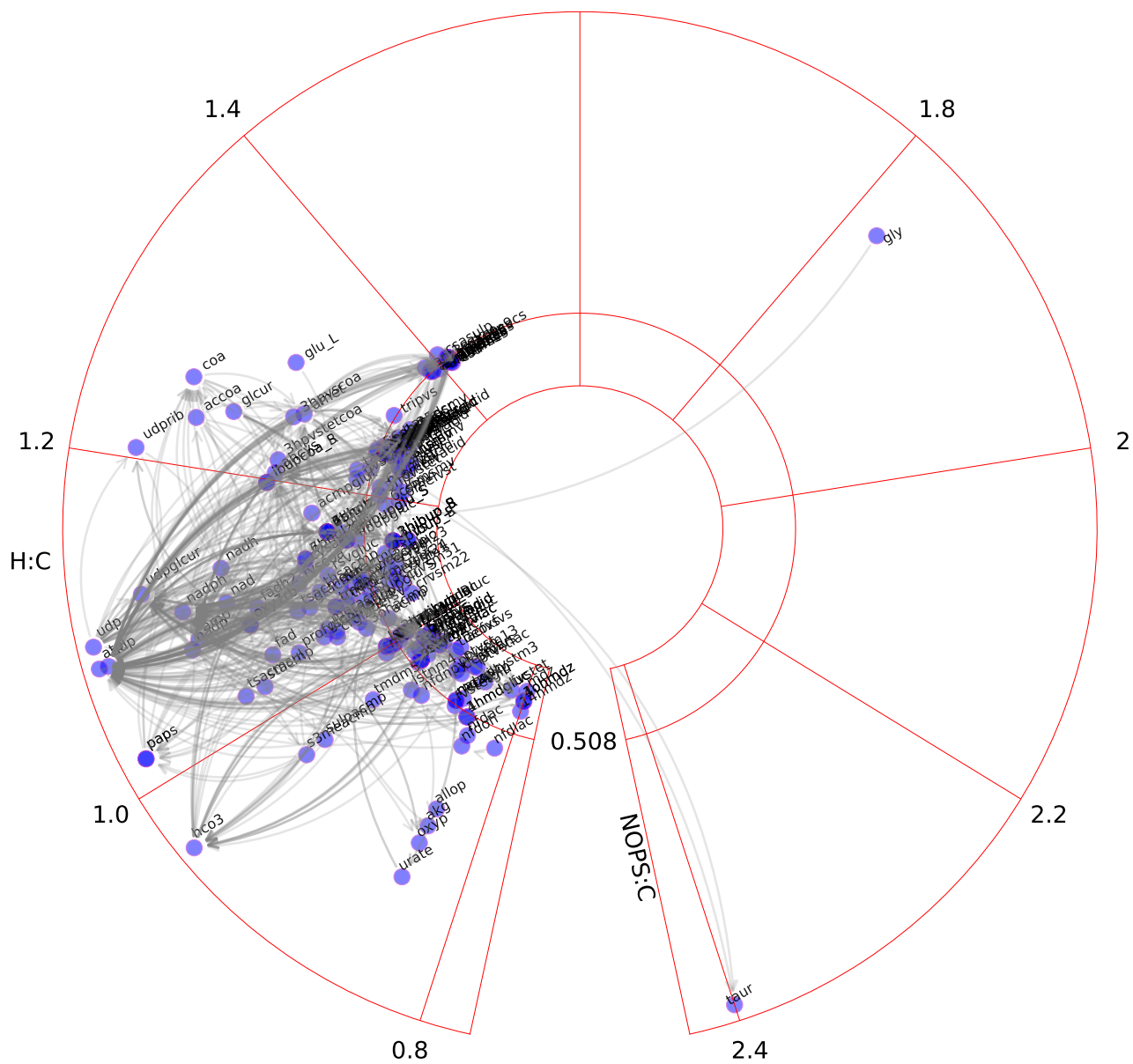

Supplement: Supplement 1 [file media-1.zip › Suppl_File_all_pathways/labeled/Drug metabolism.pdf]

# Urea cycle

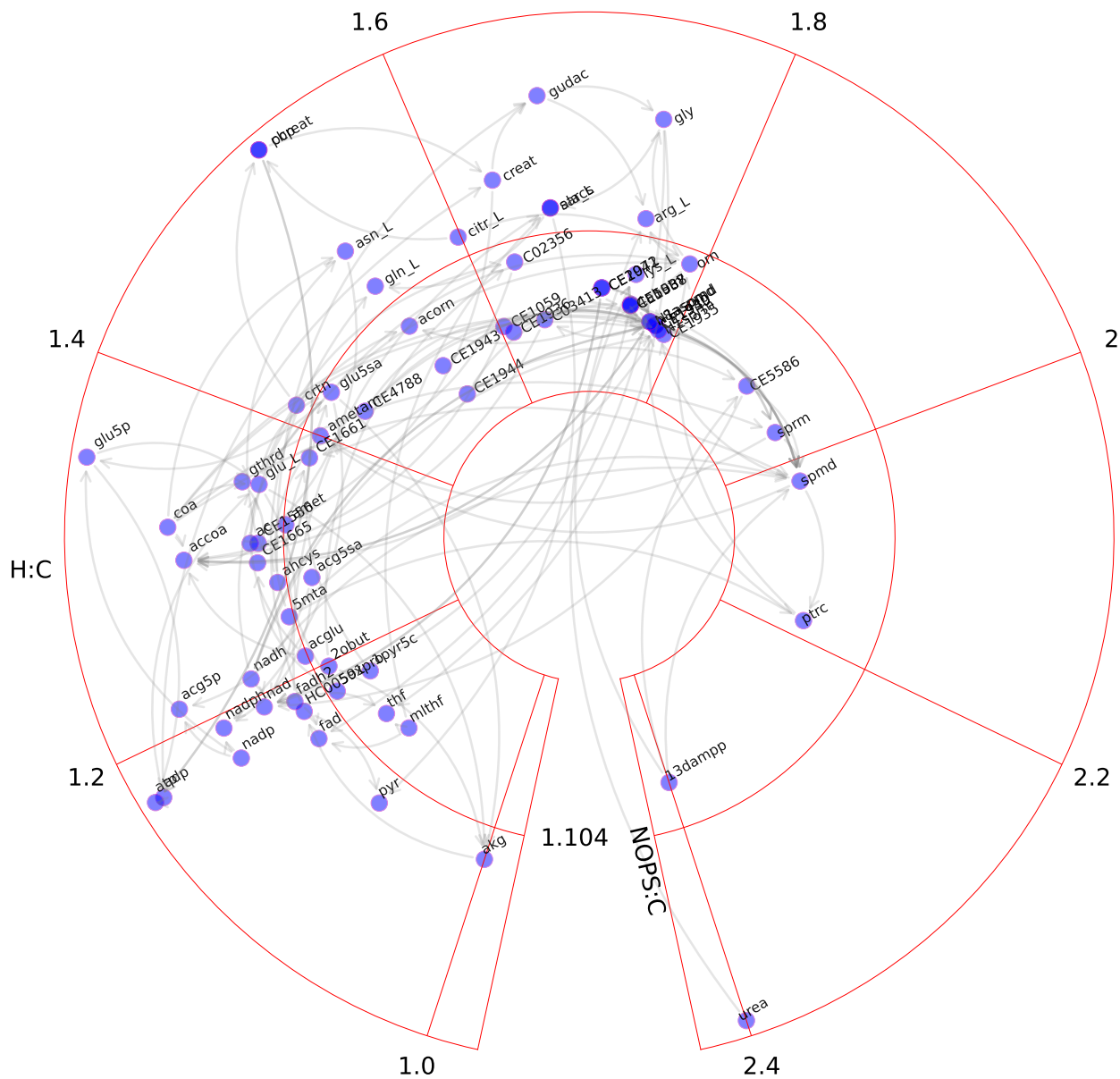

Supplement: Supplement 1 [file media-1.zip › Suppl_File_all_pathways/labeled/Urea cycle.pdf]

# Vitamin B12 metabolism

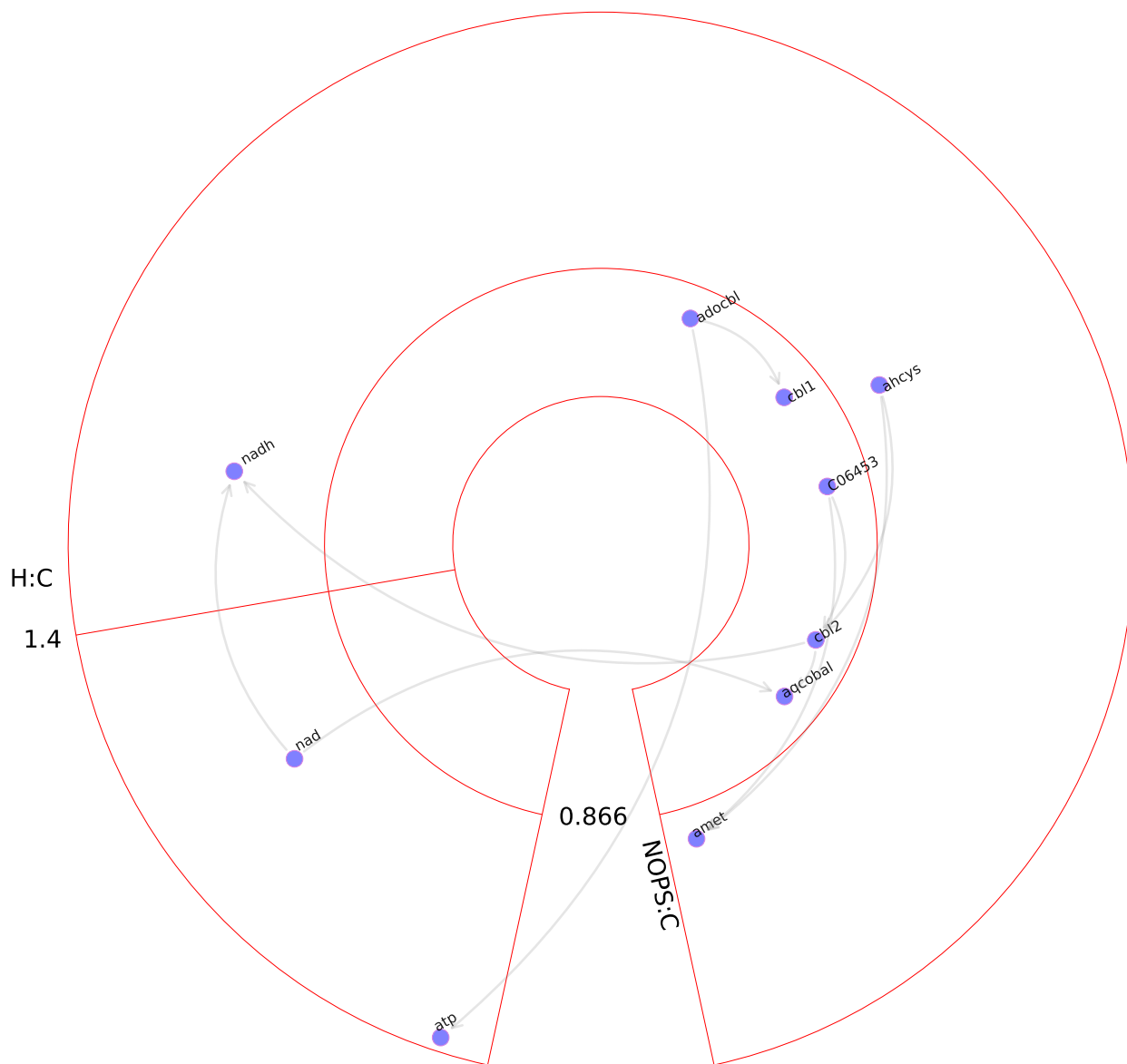

Supplement: Supplement 1 [file media-1.zip › Suppl_File_all_pathways/labeled/Vitamin B12 metabolism.pdf]

# C5-branched dibasic acid metabolism

1.2

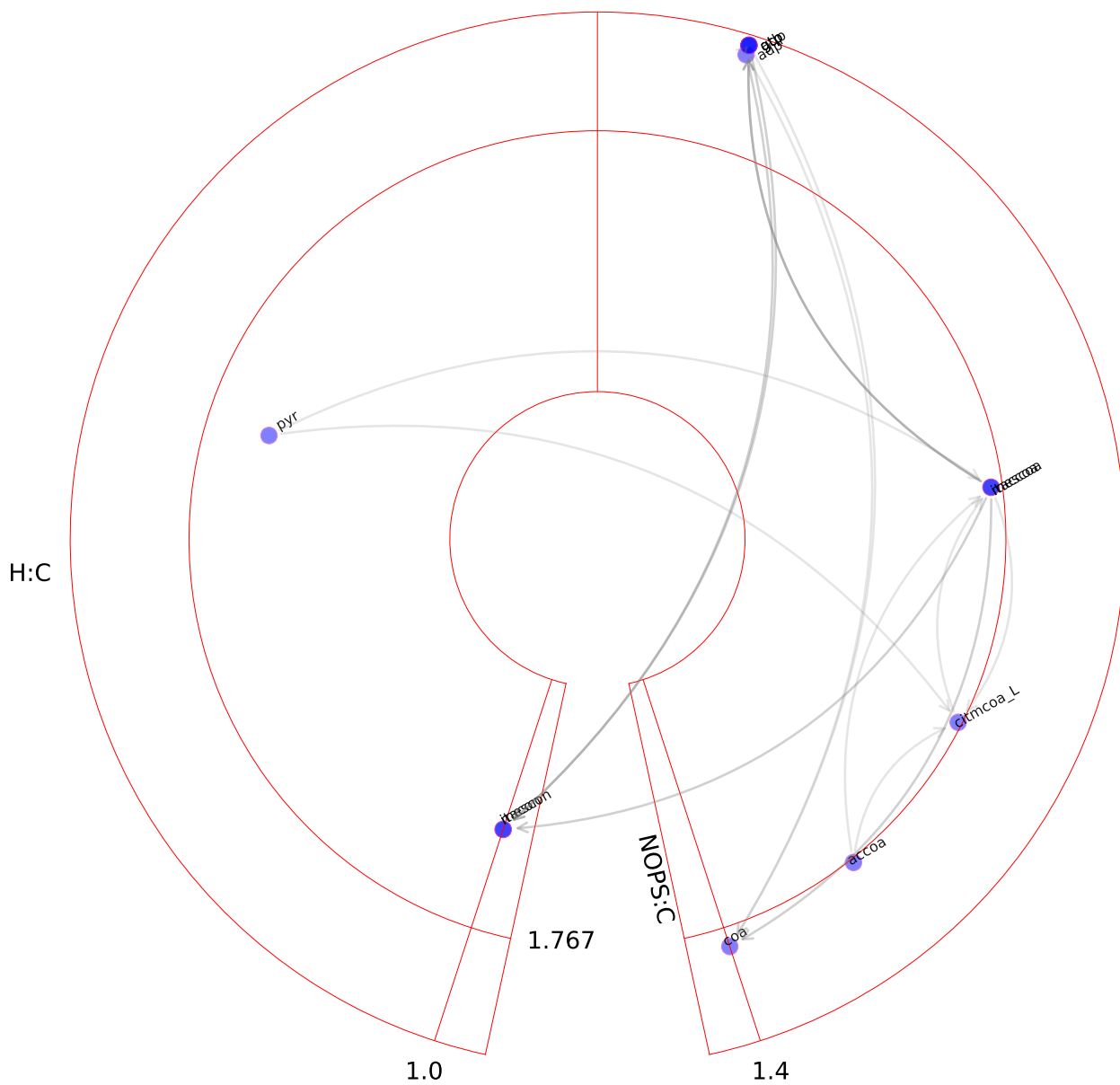

Supplement: Supplement 1 [file media-1.zip › Suppl_File_all_pathways/labeled/C5-branched dibasic acid metabolism.pdf]

Nucleotide salvage pathway

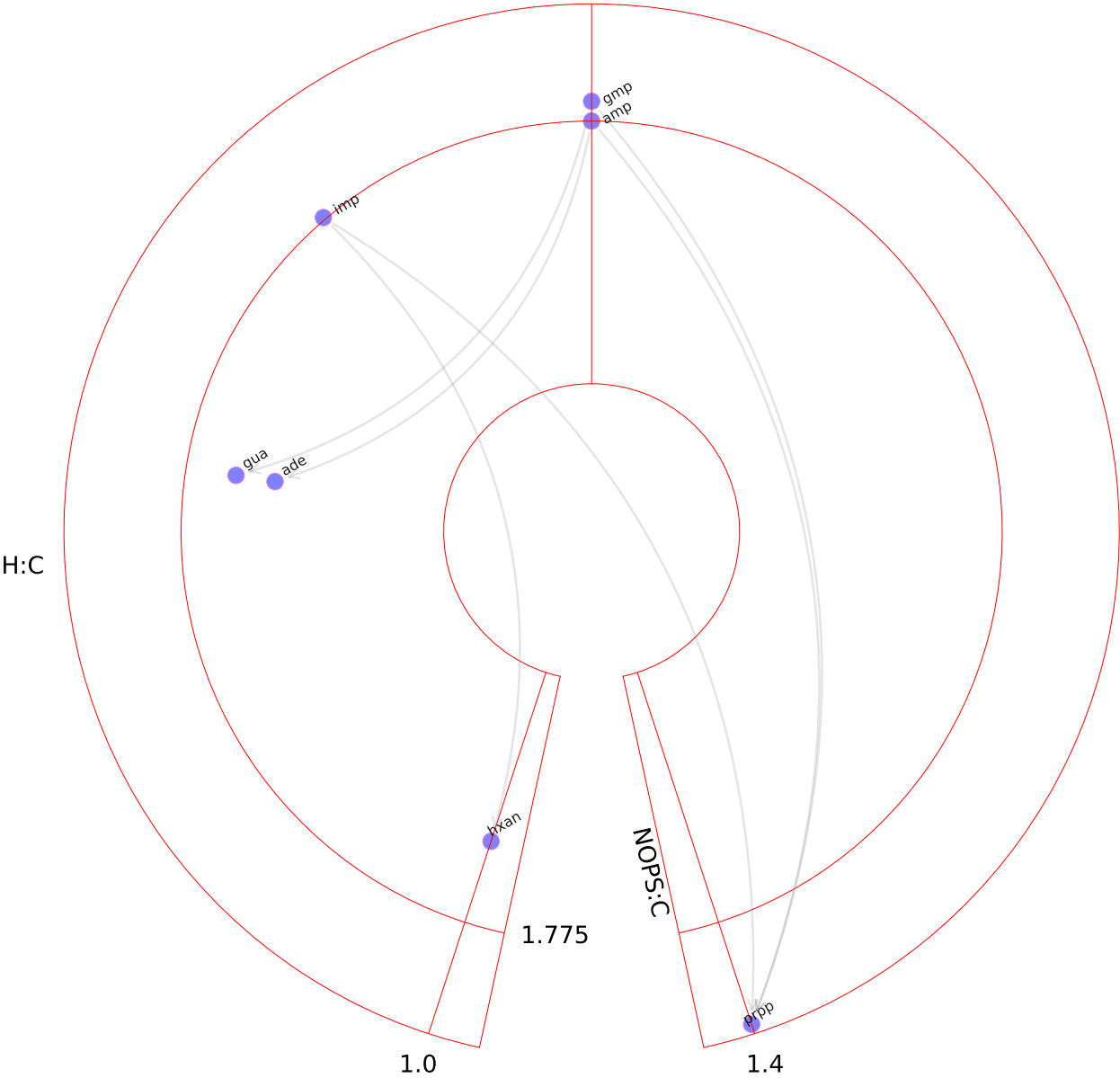

Supplement: Supplement 1 [file media-1.zip › Suppl_File_all_pathways/labeled/Nucleotide salvage pathway.pdf]

## Sphingolipid metabolism

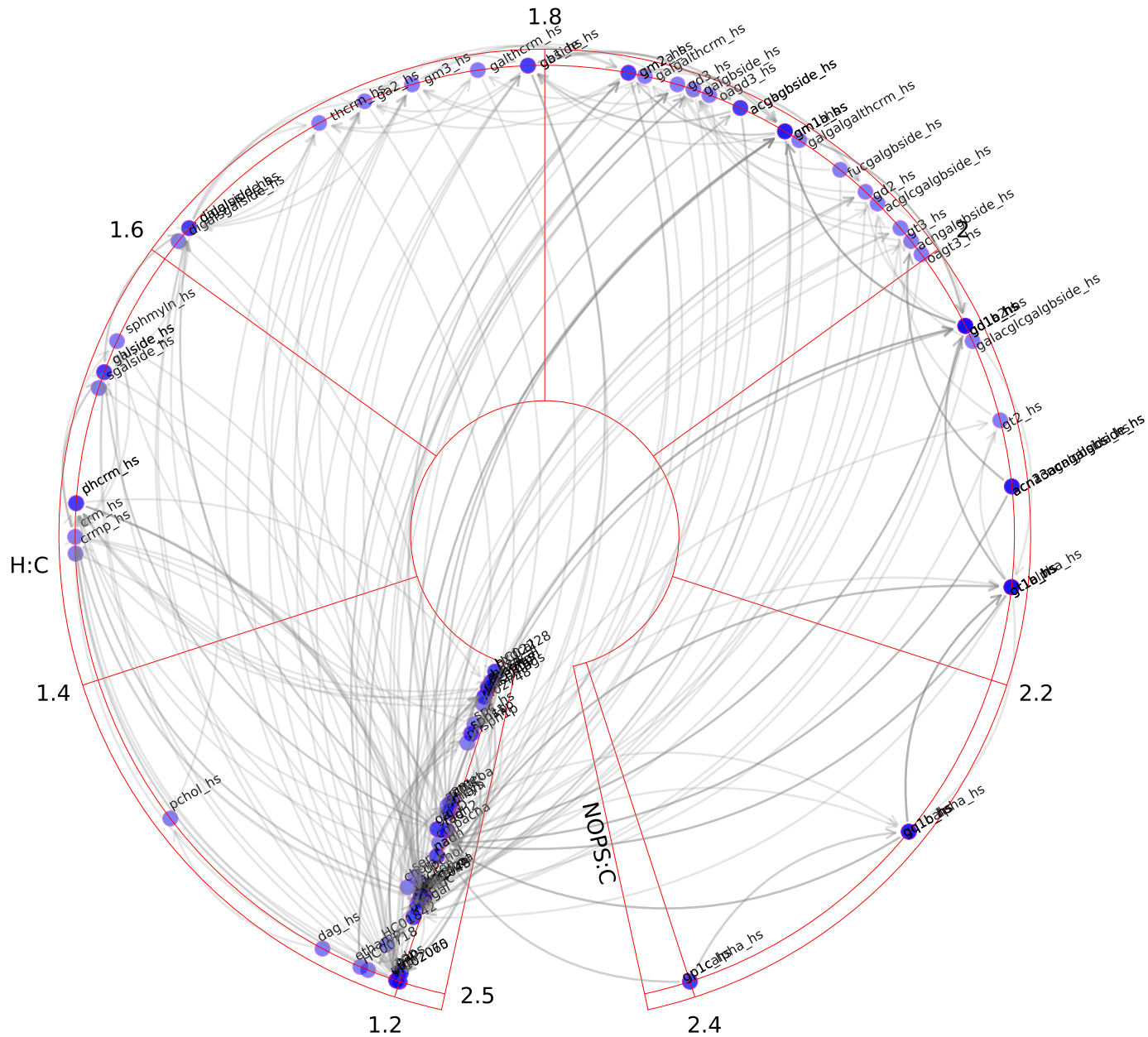

Supplement: Supplement 1 [file media-1.zip › Suppl_File_all_pathways/labeled/Sphingolipid metabolism.pdf]

## 1.6

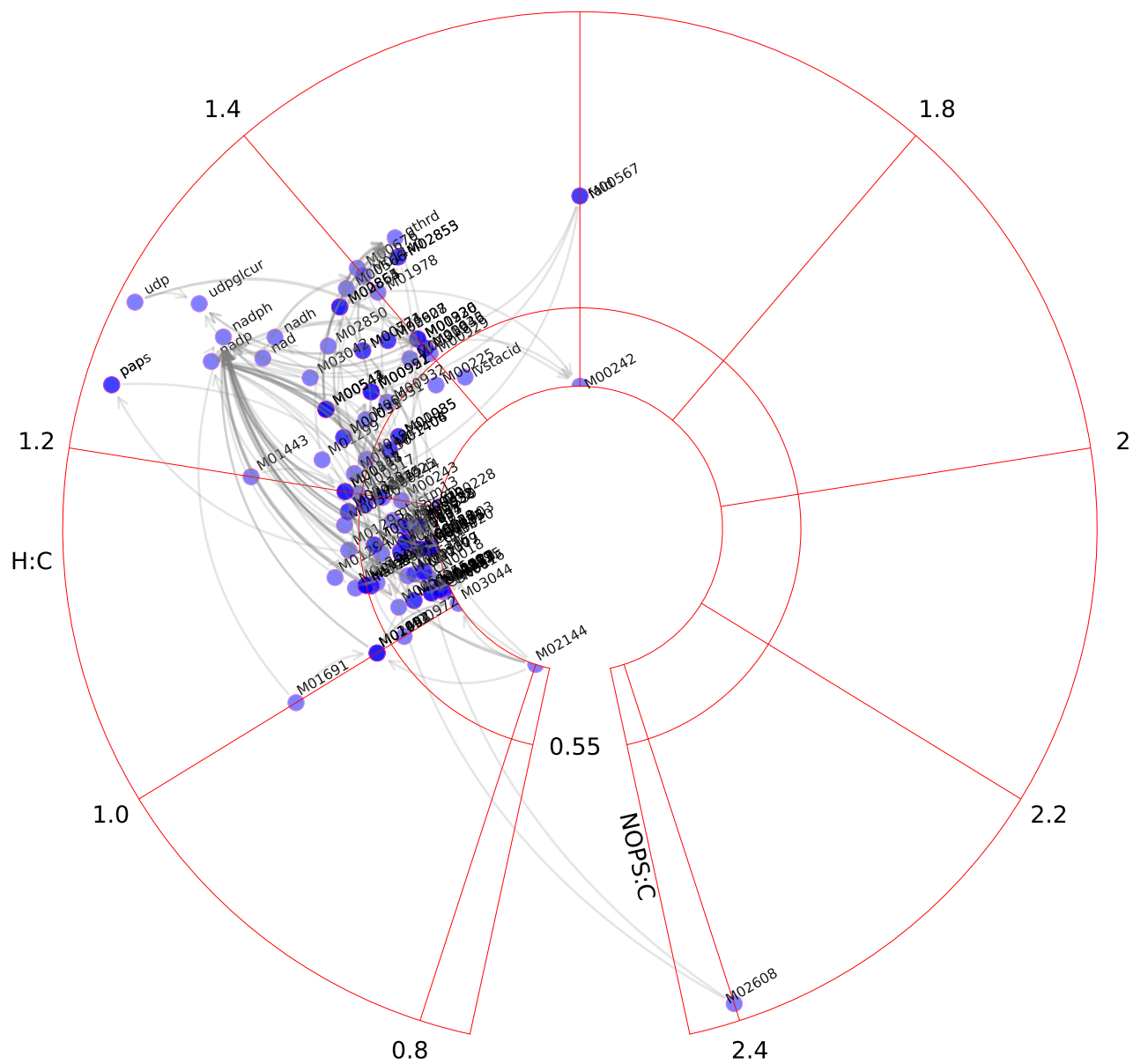

Supplement: Supplement 1 [file media-1.zip › Suppl_File_all_pathways/labeled/Xenobiotics metabolism.pdf]

# Leukotriene metabolism

1.8

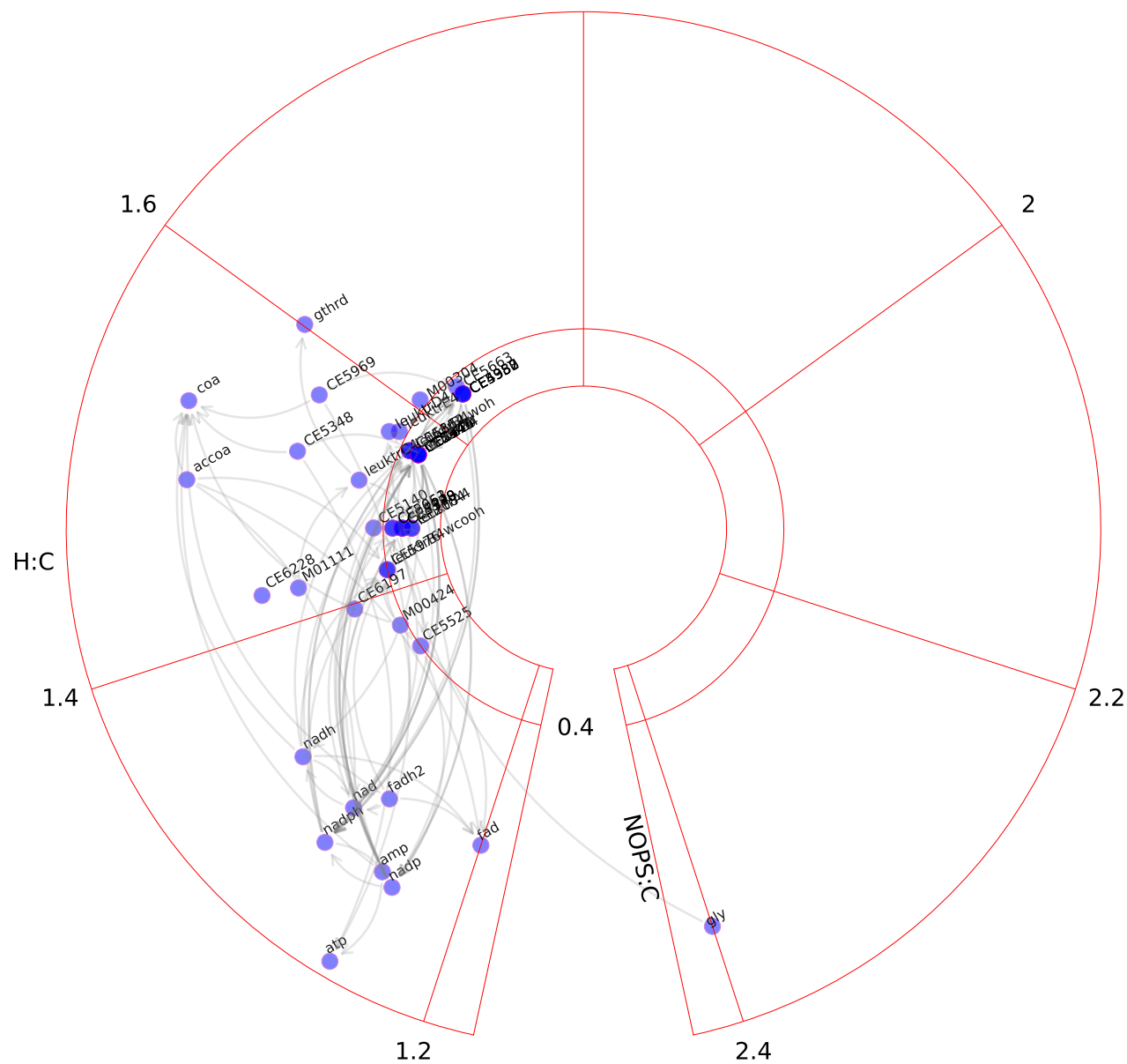

Supplement: Supplement 1 [file media-1.zip › Suppl_File_all_pathways/labeled/Leukotriene metabolism.pdf]

# Lipoate metabolism

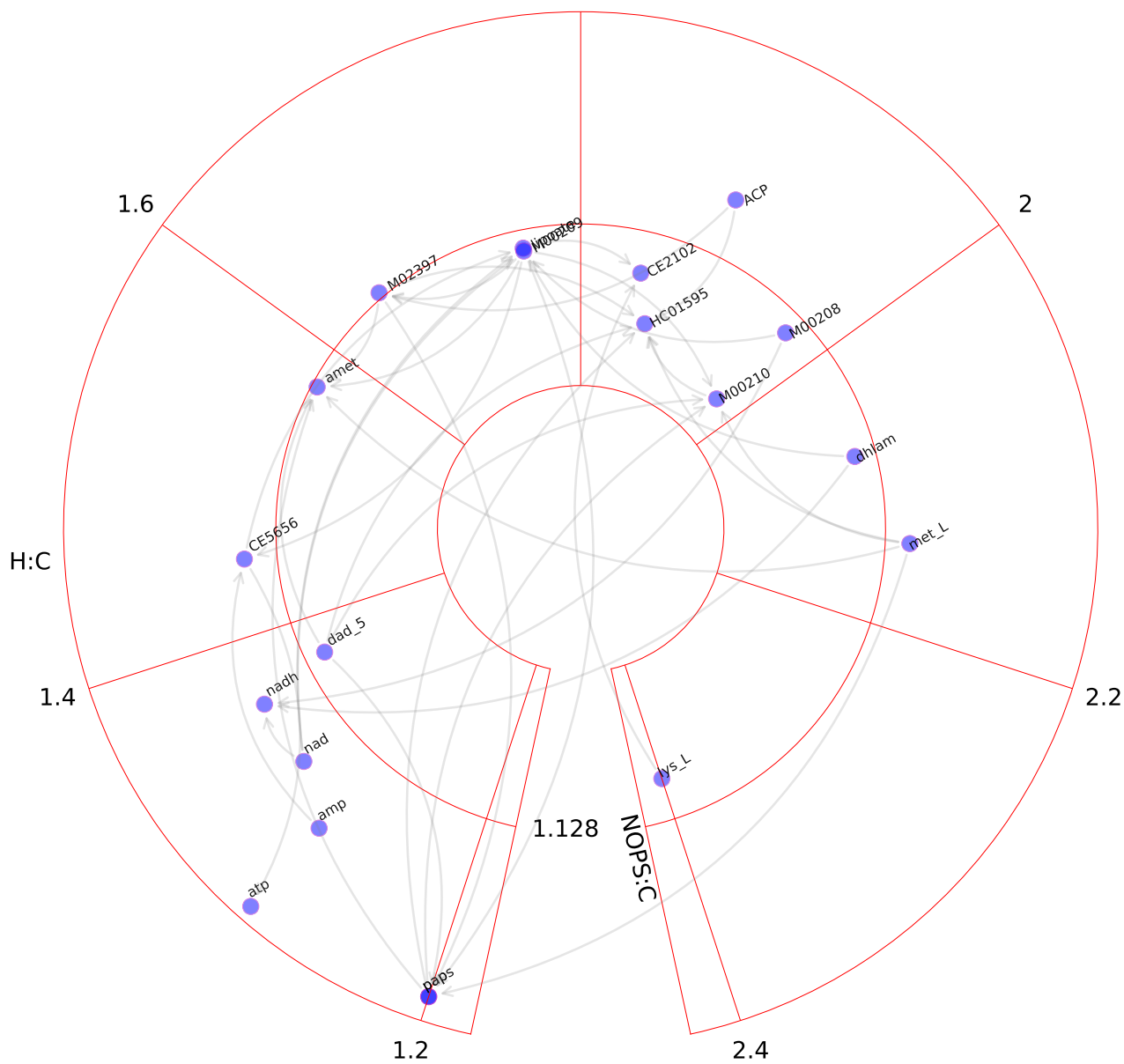

Supplement: Supplement 1 [file media-1.zip › Suppl_File_all_pathways/labeled/Lipoate metabolism.pdf]

## Androgen and estrogen synthesis and metabolism

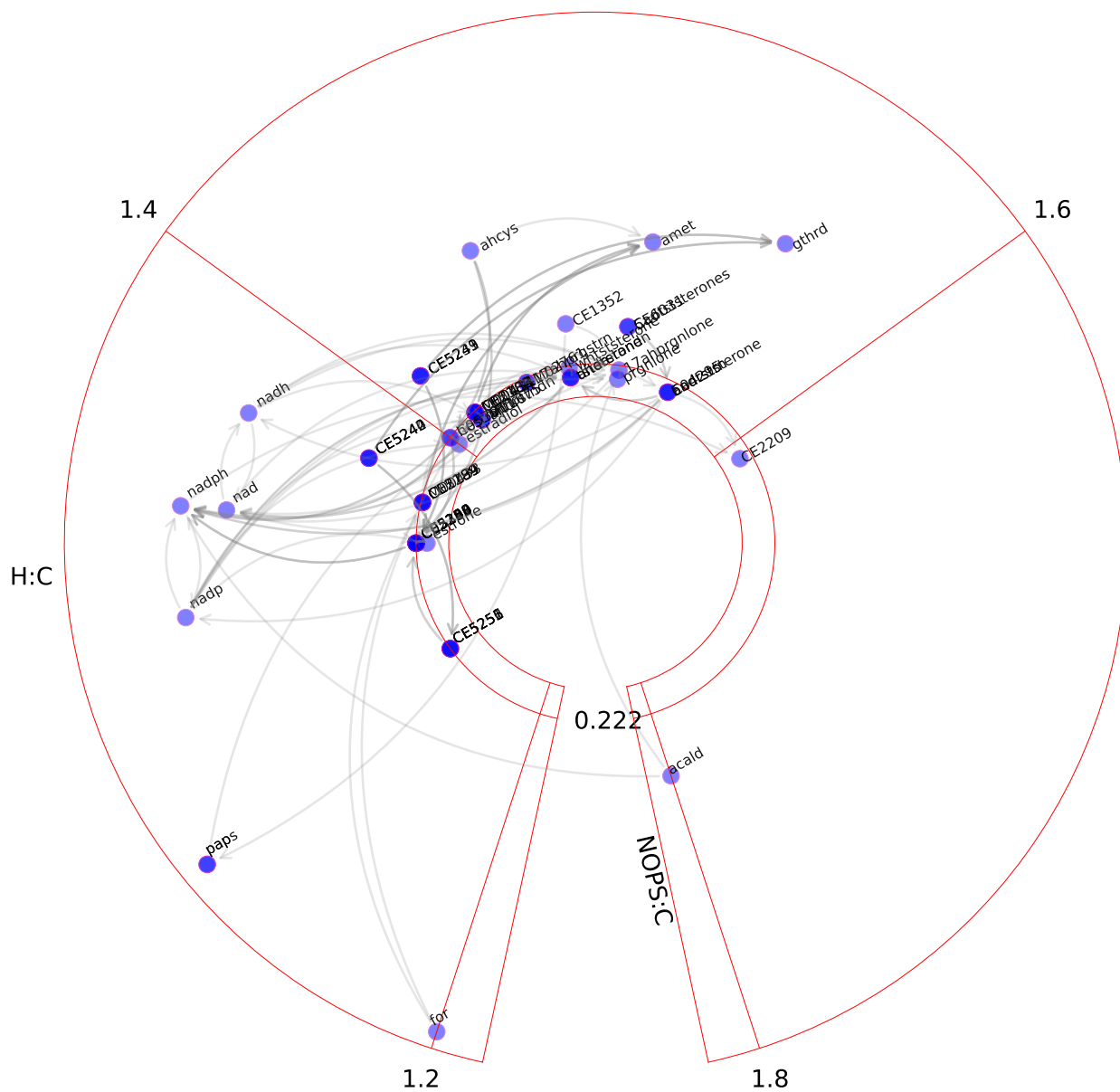

Supplement: Supplement 1 [file media-1.zip › Suppl_File_all_pathways/labeled/Androgen and estrogen synthesis and metabolism.pdf]

# Purine catabolism

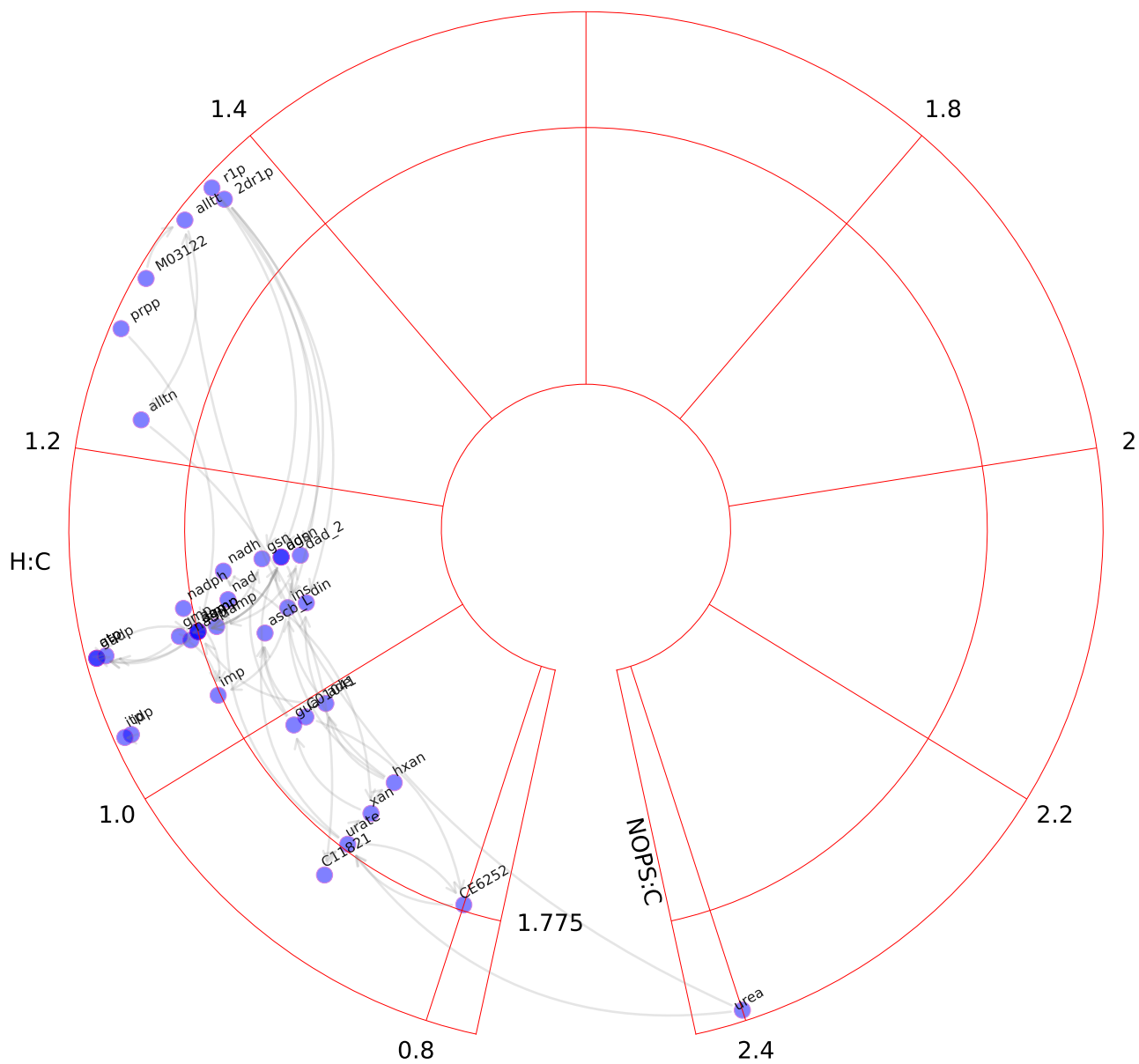

Supplement: Supplement 1 [file media-1.zip › Suppl_File_all_pathways/labeled/Purine catabolism.pdf]

## Starch and sucrose metabolism

## 1.6

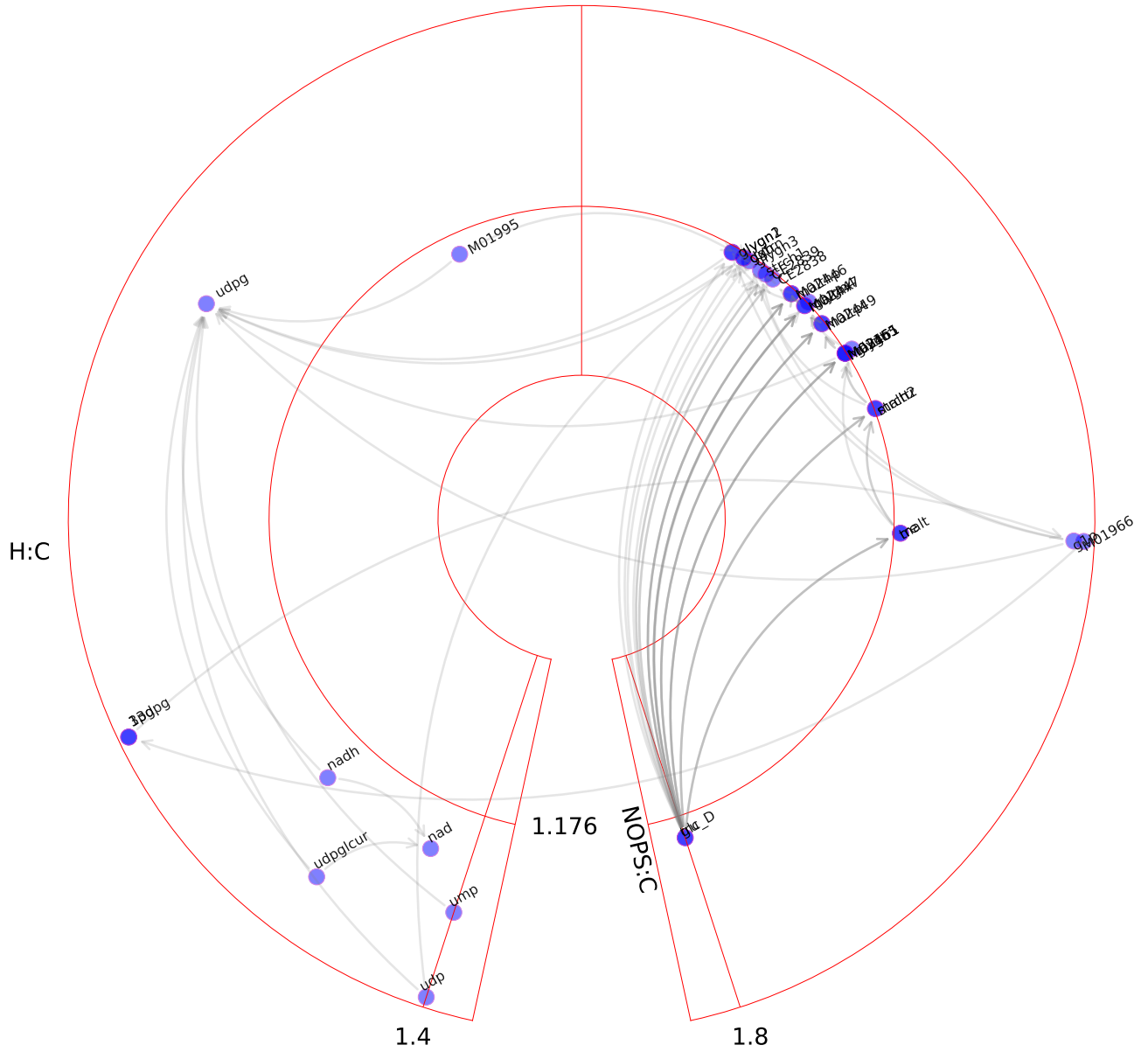

Supplement: Supplement 1 [file media-1.zip › Suppl_File_all_pathways/labeled/Starch and sucrose metabolism.pdf]

## Valine, leucine, and isoleucine metabolism

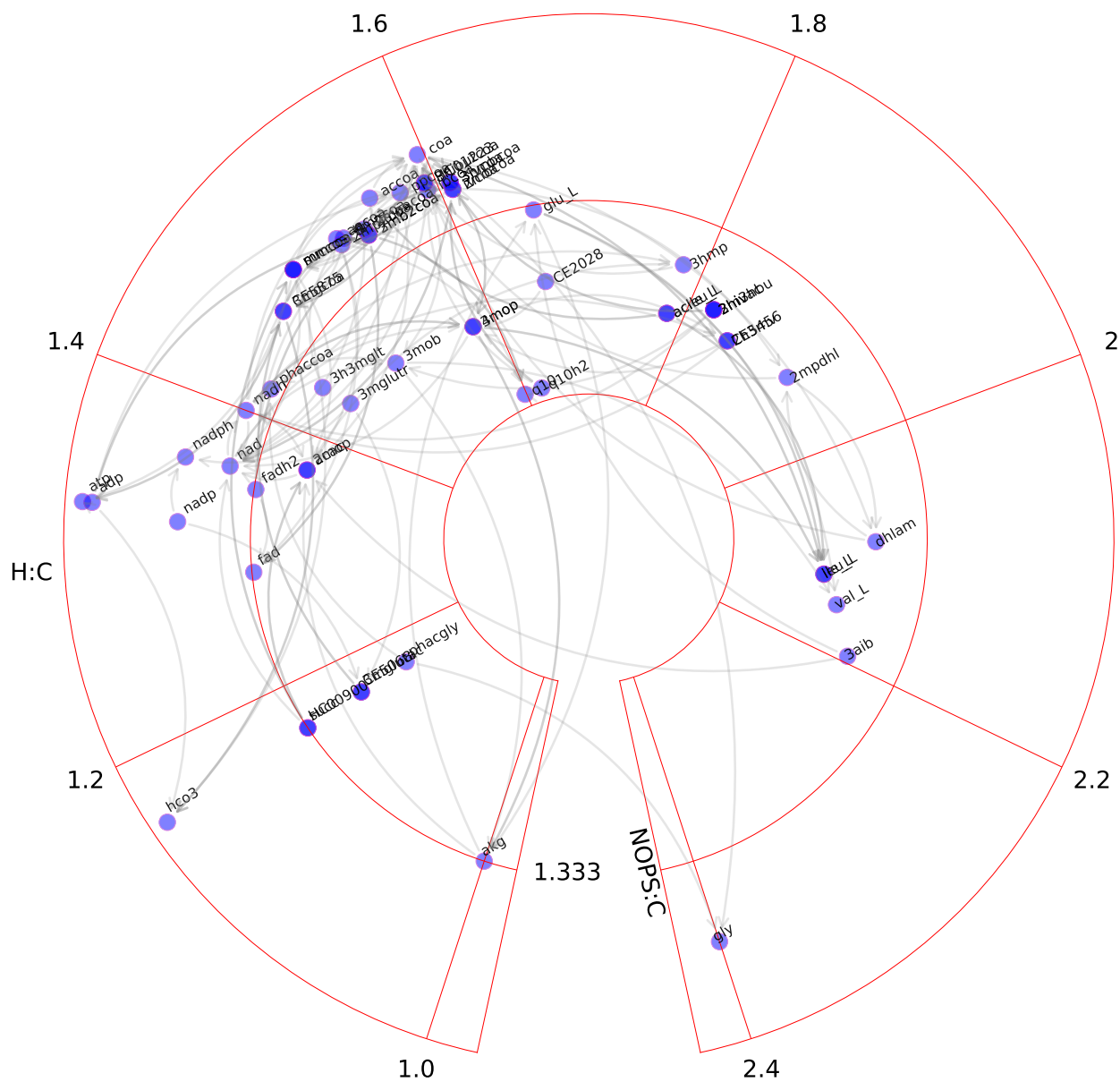

Supplement: Supplement 1 [file media-1.zip › Suppl_File_all_pathways/labeled/Valine, leucine, and isoleucine metabolism.pdf]

## Tryptophan metabolism

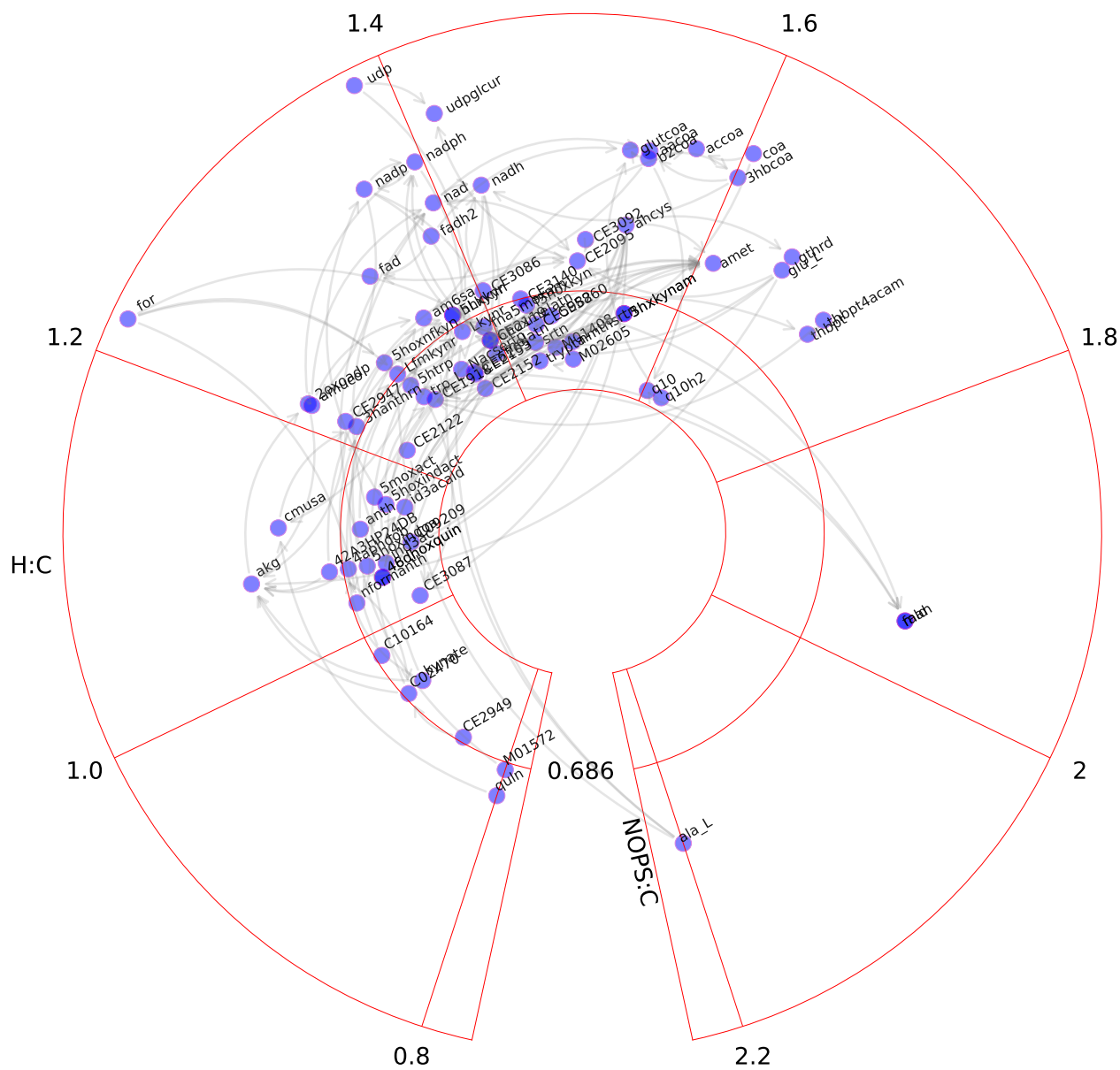

Supplement: Supplement 1 [file media-1.zip › Suppl_File_all_pathways/labeled/Tryptophan metabolism.pdf]

## Glycosphingolipid metabolism

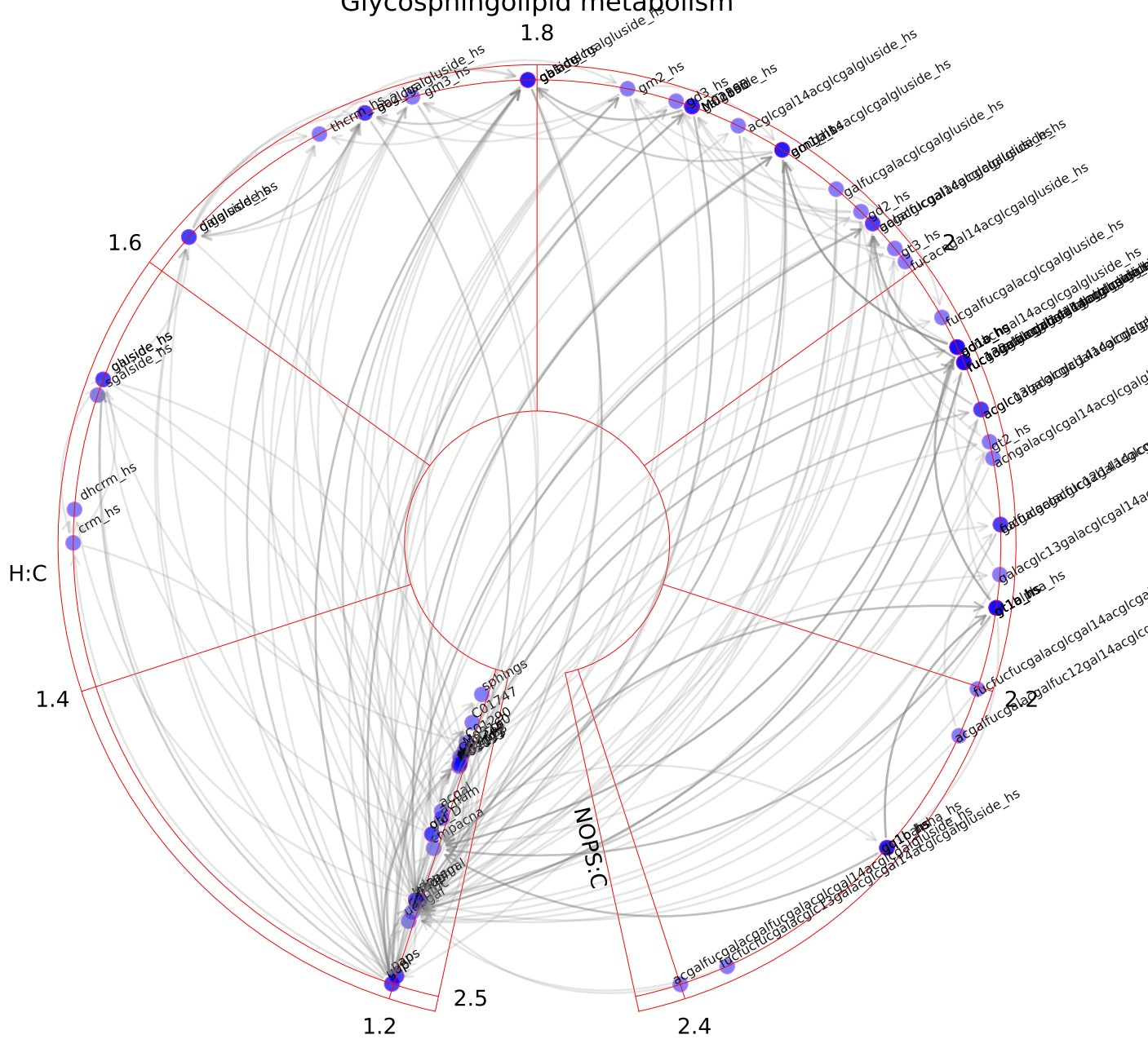

Supplement: Supplement 1 [file media-1.zip › Suppl_File_all_pathways/labeled/Glycosphingolipid metabolism.pdf]

## Methionine and cysteine metabolism

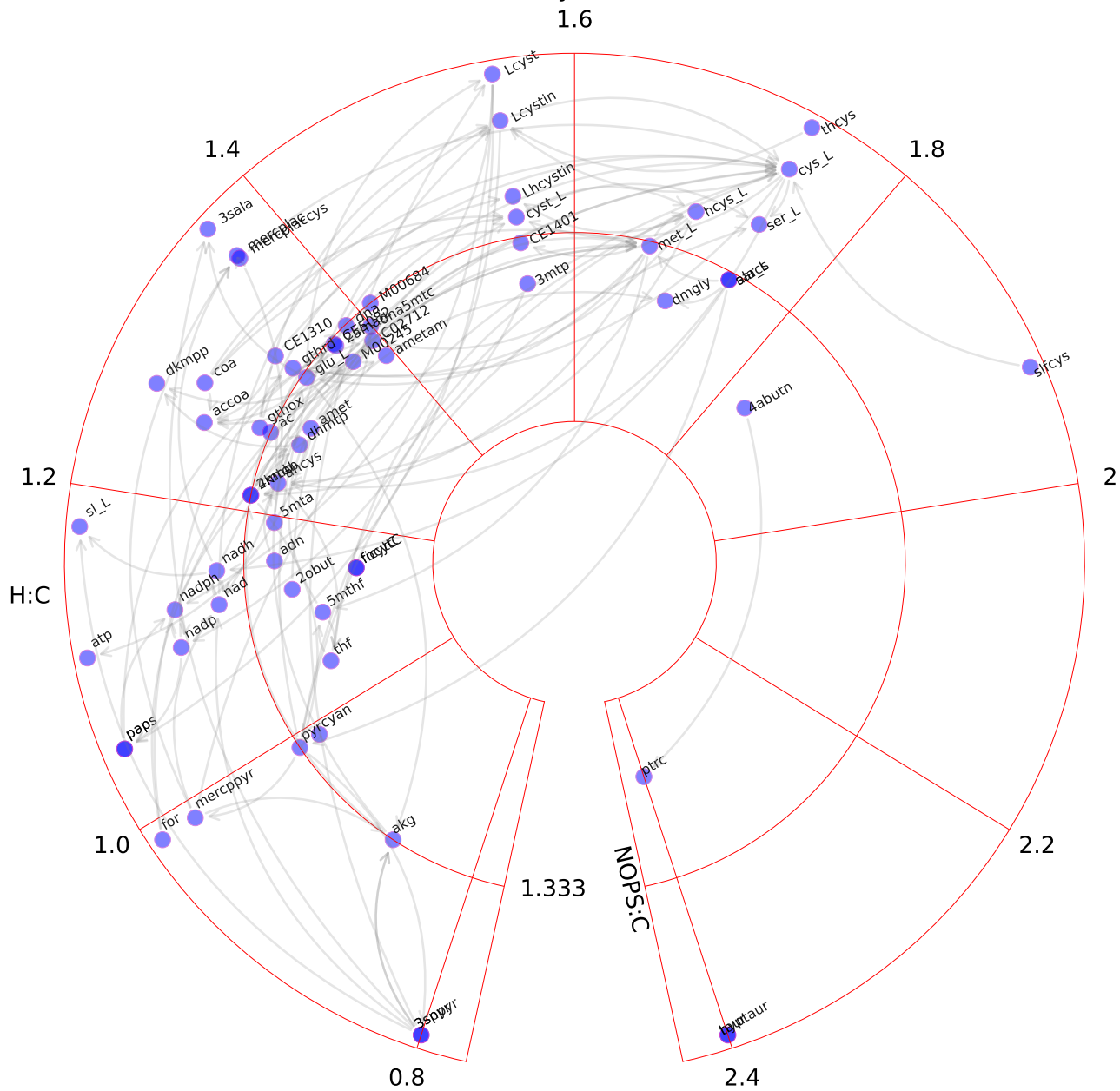

Supplement: Supplement 1 [file media-1.zip › Suppl_File_all_pathways/labeled/Methionine and cysteine metabolism.pdf]

## Glycolysis/gluconeogenesis

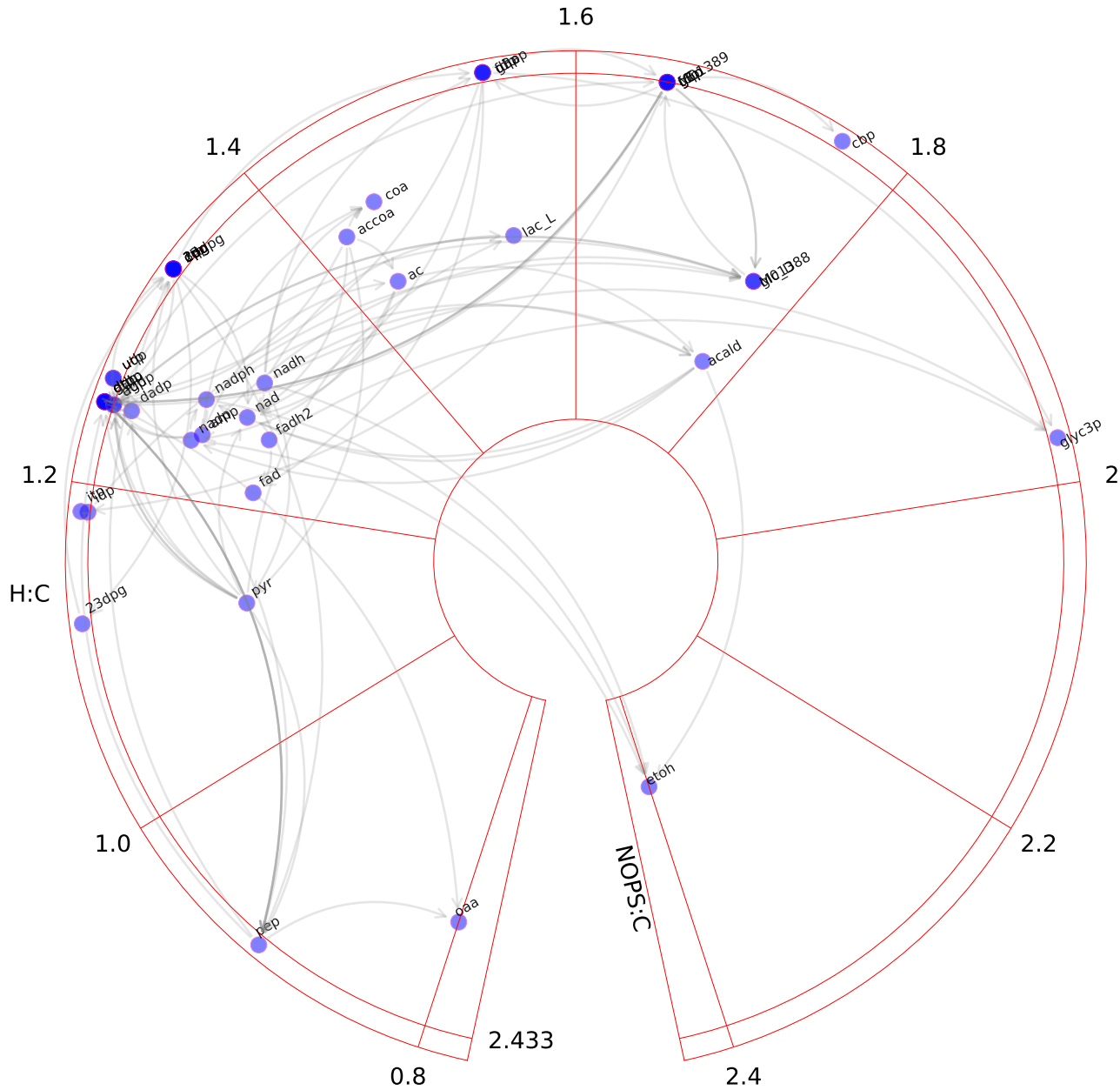

Supplement: Supplement 1 [file media-1.zip › Suppl_File_all_pathways/labeled/Glycolysis_gluconeogenesis.pdf]

## 1.8

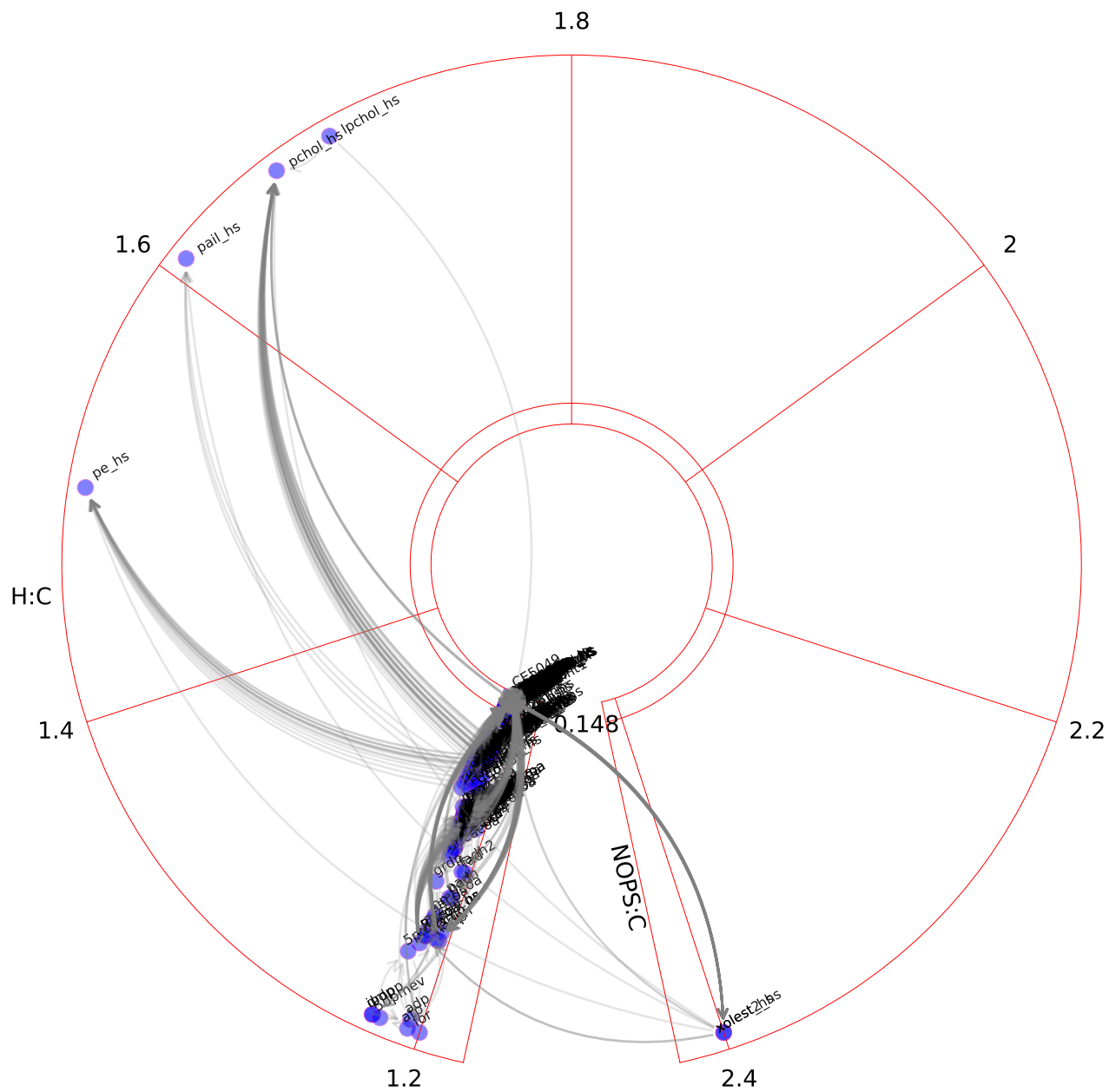

Supplement: Supplement 1 [file media-1.zip › Suppl_File_all_pathways/labeled/Cholesterol metabolism.pdf]

## 1.6

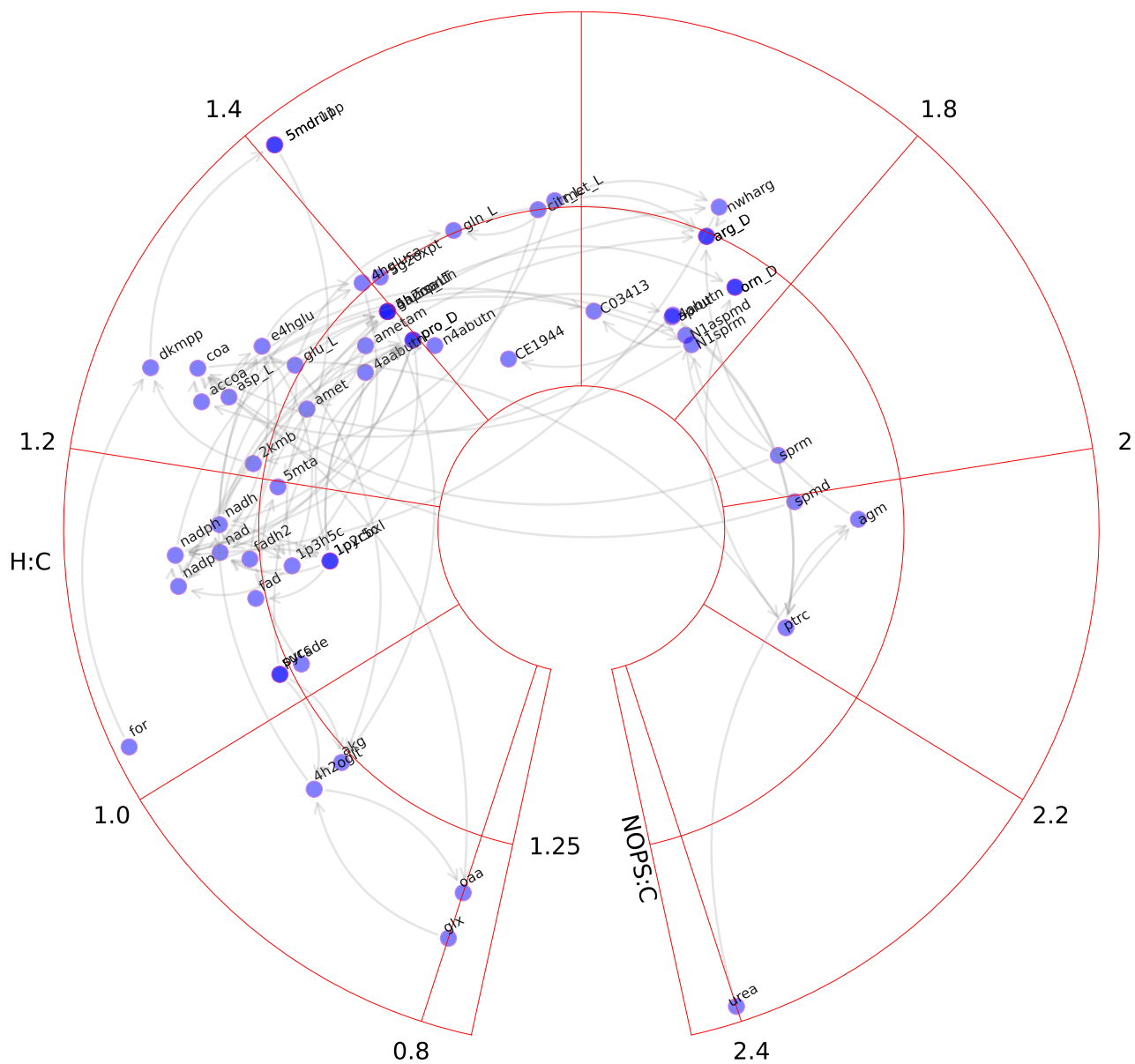

Supplement: Supplement 1 [file media-1.zip › Suppl_File_all_pathways/labeled/Arginine and proline metabolism.pdf]

# Triacylglycerol synthesis

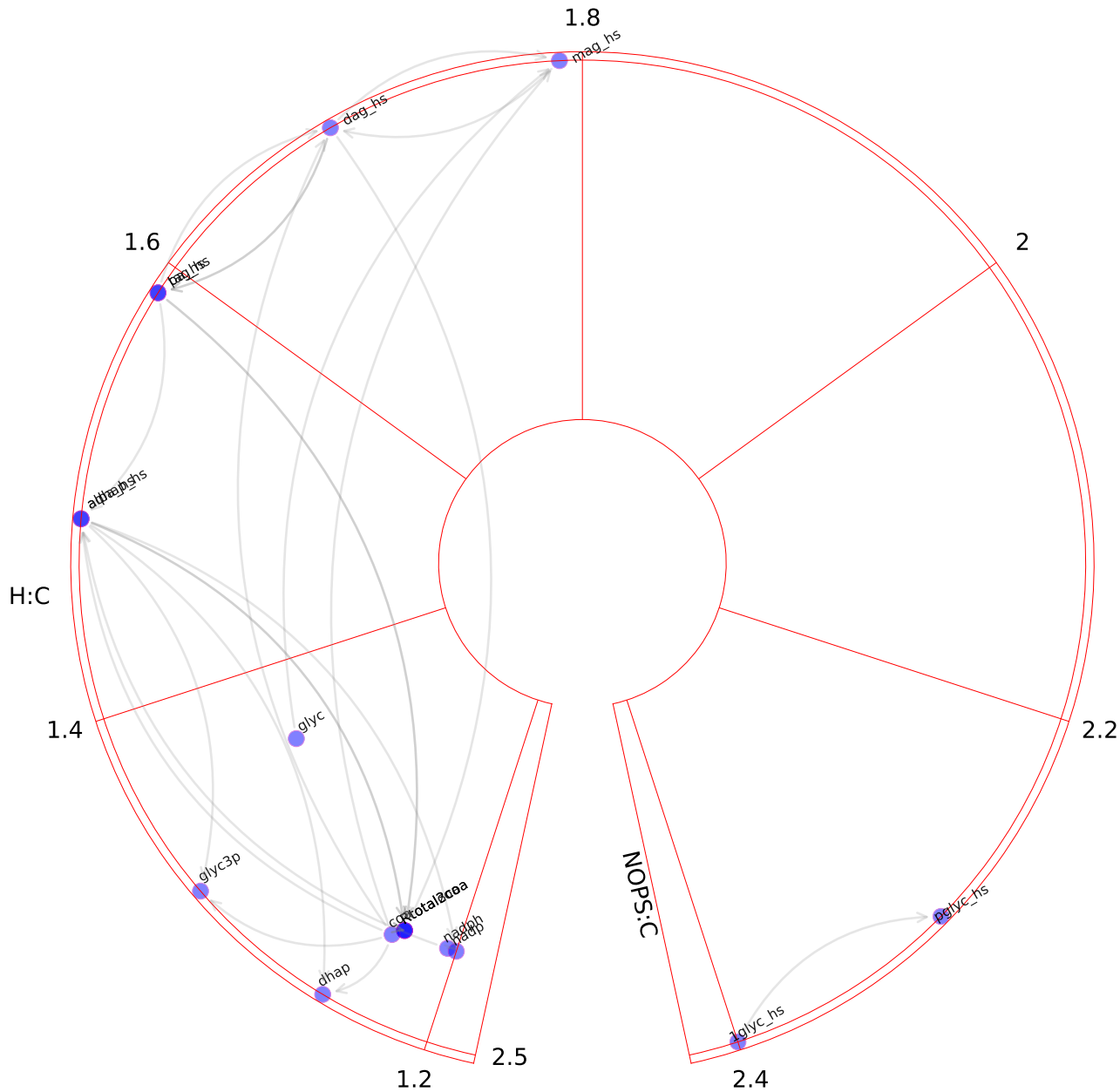

Supplement: Supplement 1 [file media-1.zip › Suppl_File_all_pathways/labeled/Triacylglycerol synthesis.pdf]

# Glutathione metabolism

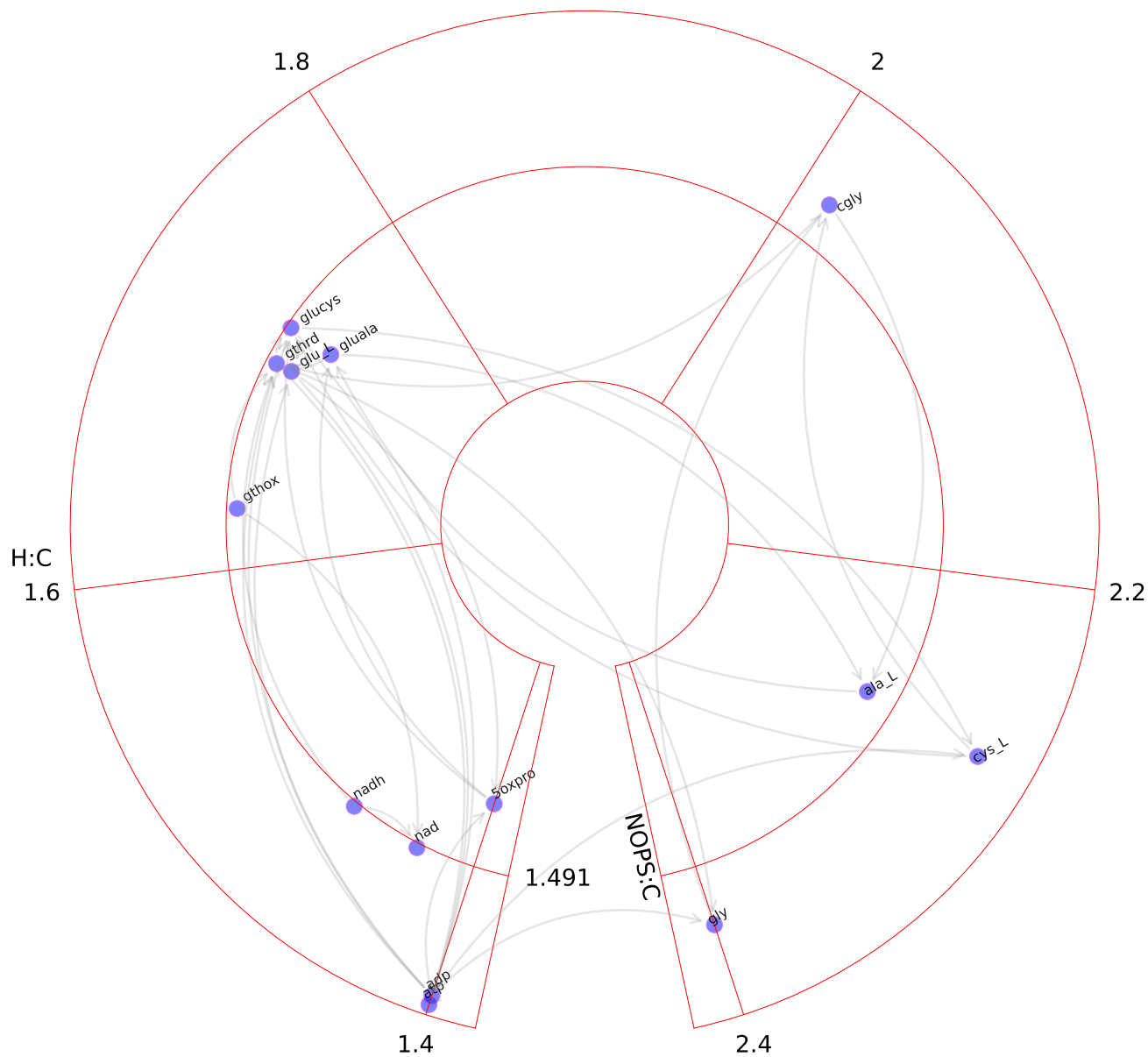

Supplement: Supplement 1 [file media-1.zip › Suppl_File_all_pathways/labeled/Glutathione metabolism.pdf]

## Bile acid synthesis

1.8

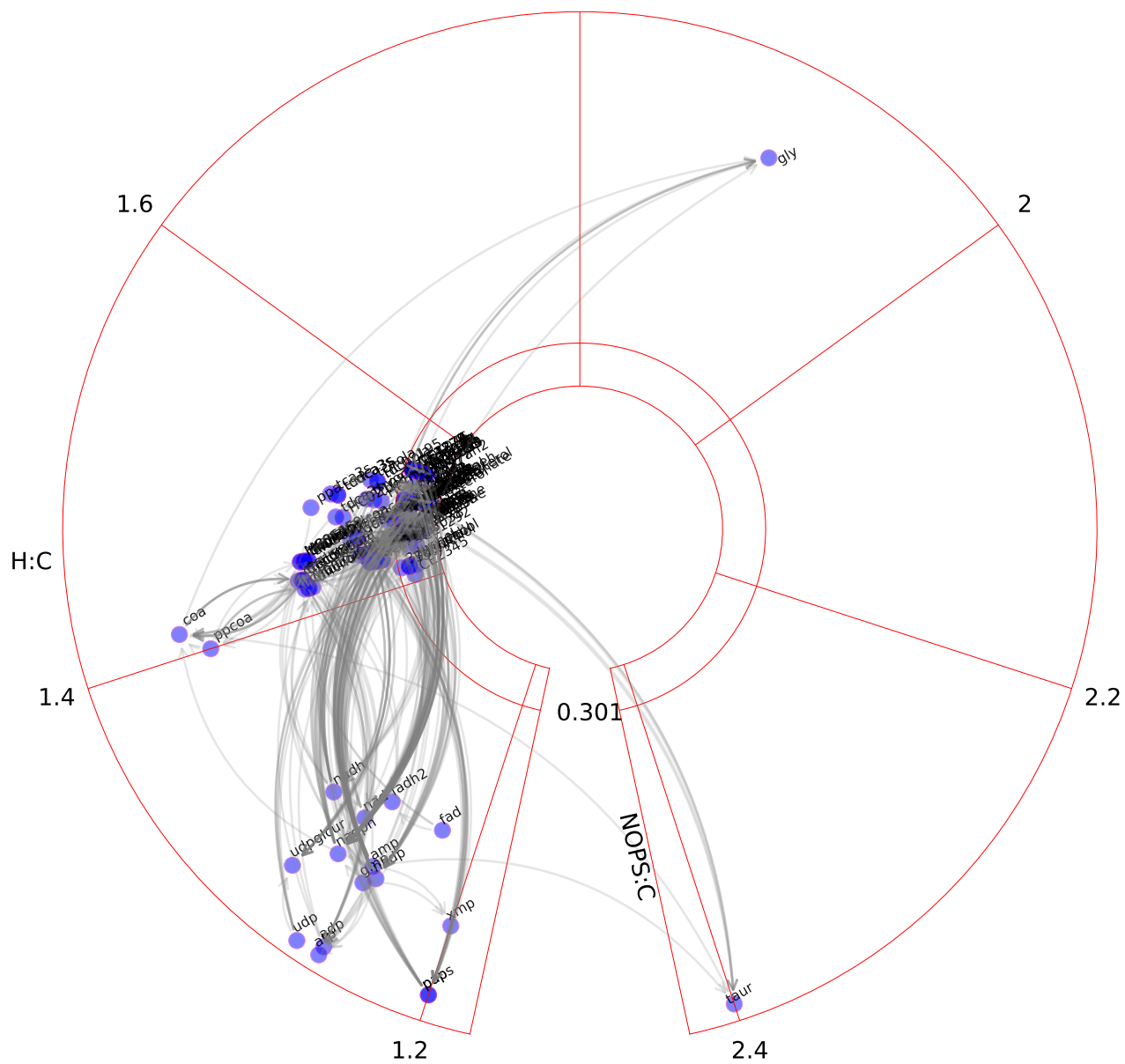

Supplement: Supplement 1 [file media-1.zip › Suppl_File_all_pathways/labeled/Bile acid synthesis.pdf]
