# Supplementary figures and images for: Circular van Krevelen diagram for visualizing metabolic pathways (part 1 of 2)
Source: bioRxiv. 2025 Jun 3:2025.05.31.657198. Preprint. [Version 1] doi: 10.1101/2025.05.31.657198 (PMC12157561; doi:10.1101/2025.05.31.657198)

# Keratan sulfate degradation

1.6

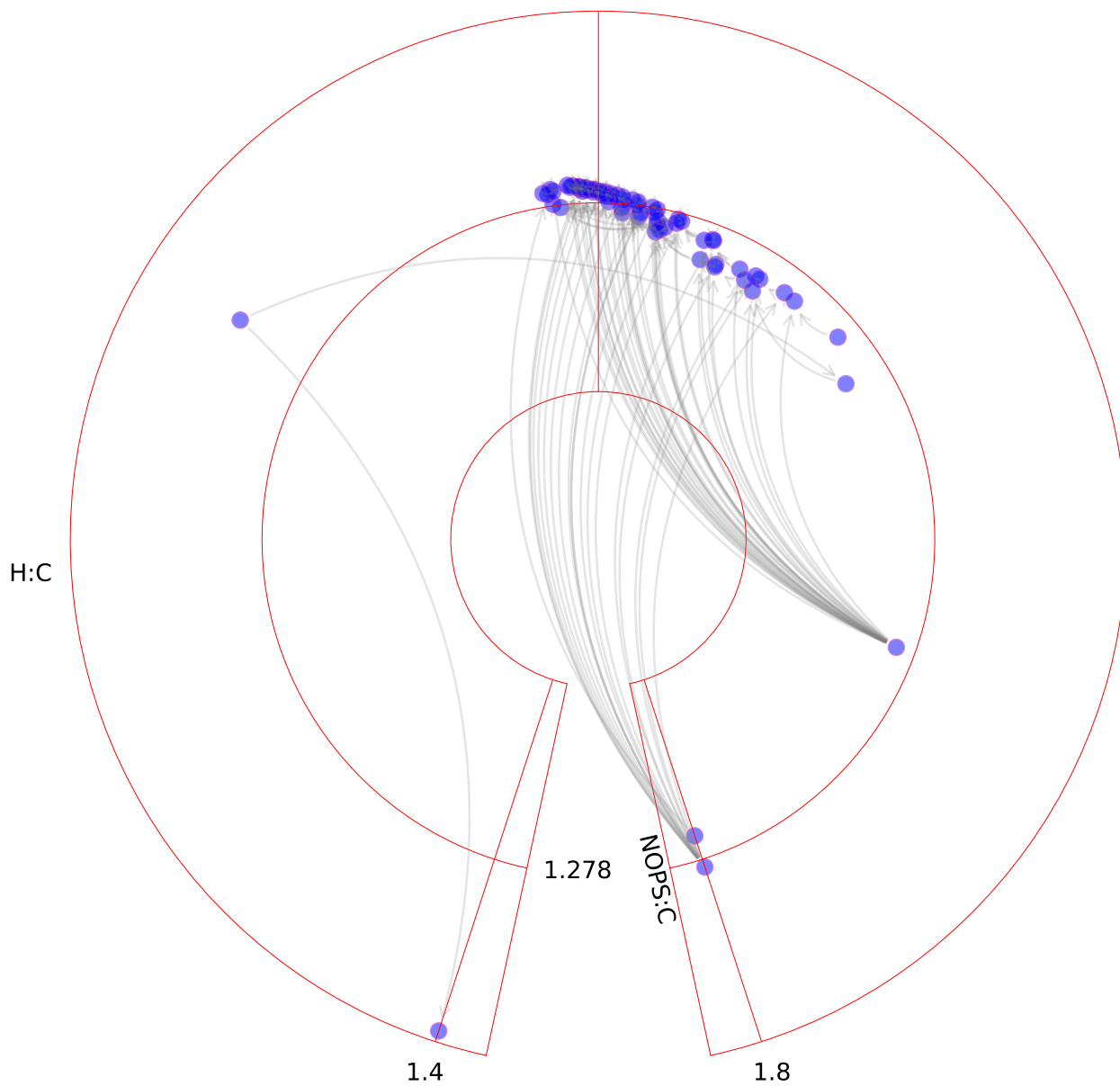

Supplement: Supplement 1 [file media-1.zip › Suppl_File_all_pathways/nolabel/Keratan sulfate degradation.pdf]

# Nucleotide metabolism

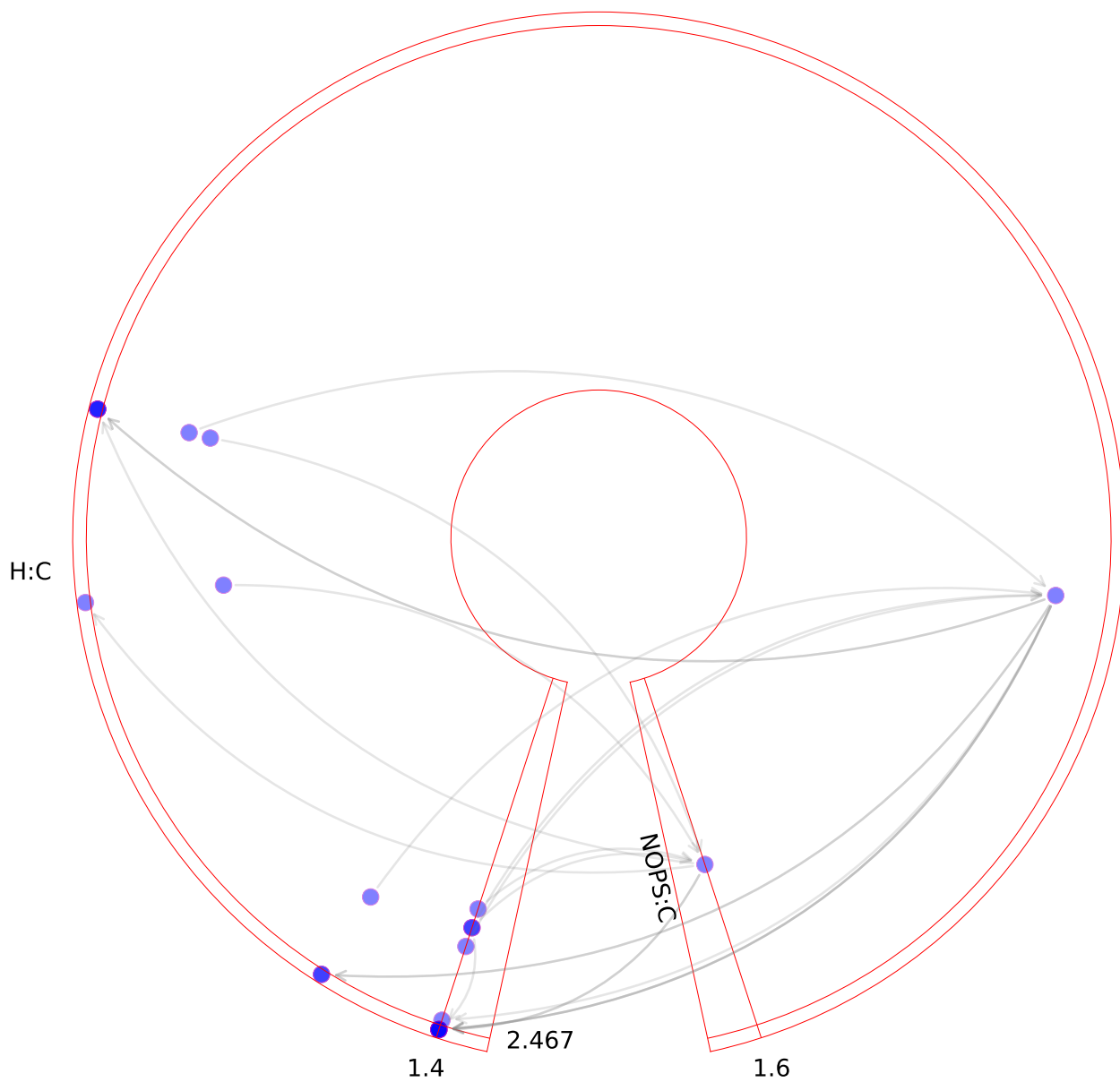

Supplement: Supplement 1 [file media-1.zip › Suppl_File_all_pathways/nolabel/Nucleotide metabolism.pdf]

# Folate metabolism

1.2

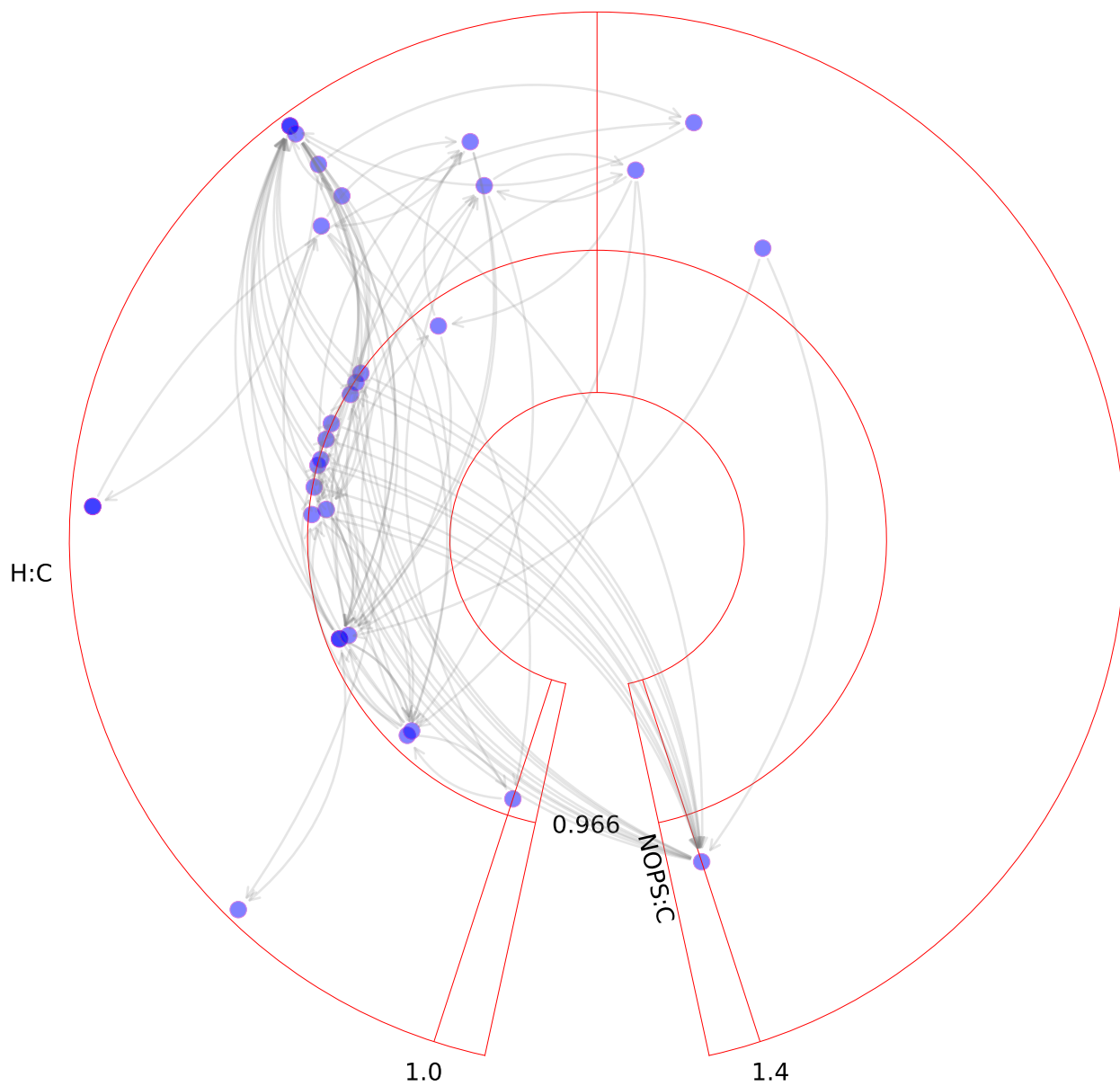

Supplement: Supplement 1 [file media-1.zip › Suppl_File_all_pathways/nolabel/Folate metabolism.pdf]

# Glycerophospholipid metabolism

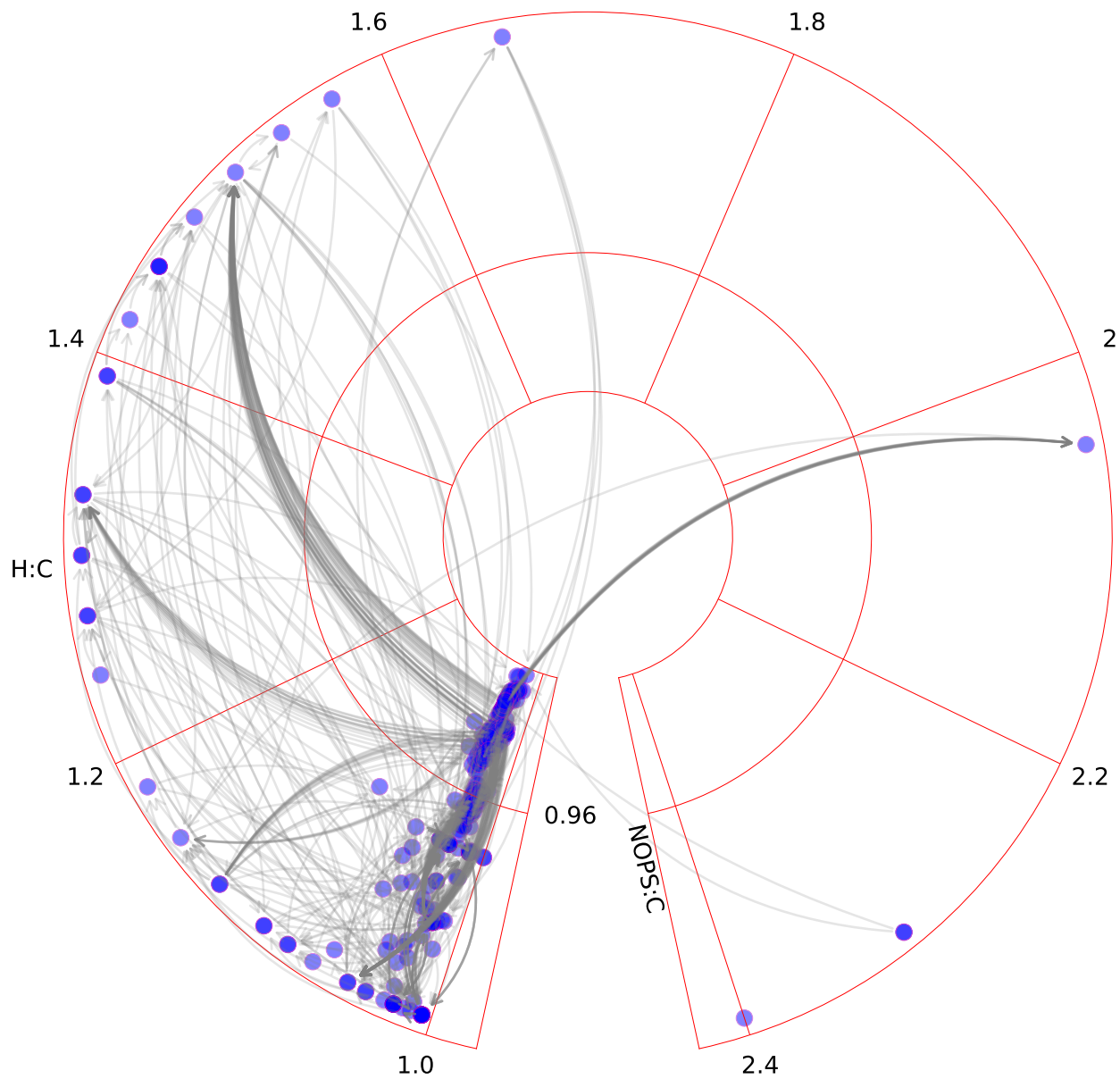

Supplement: Supplement 1 [file media-1.zip › Suppl_File_all_pathways/nolabel/Glycerophospholipid metabolism.pdf]

# Peptide metabolism

1.8

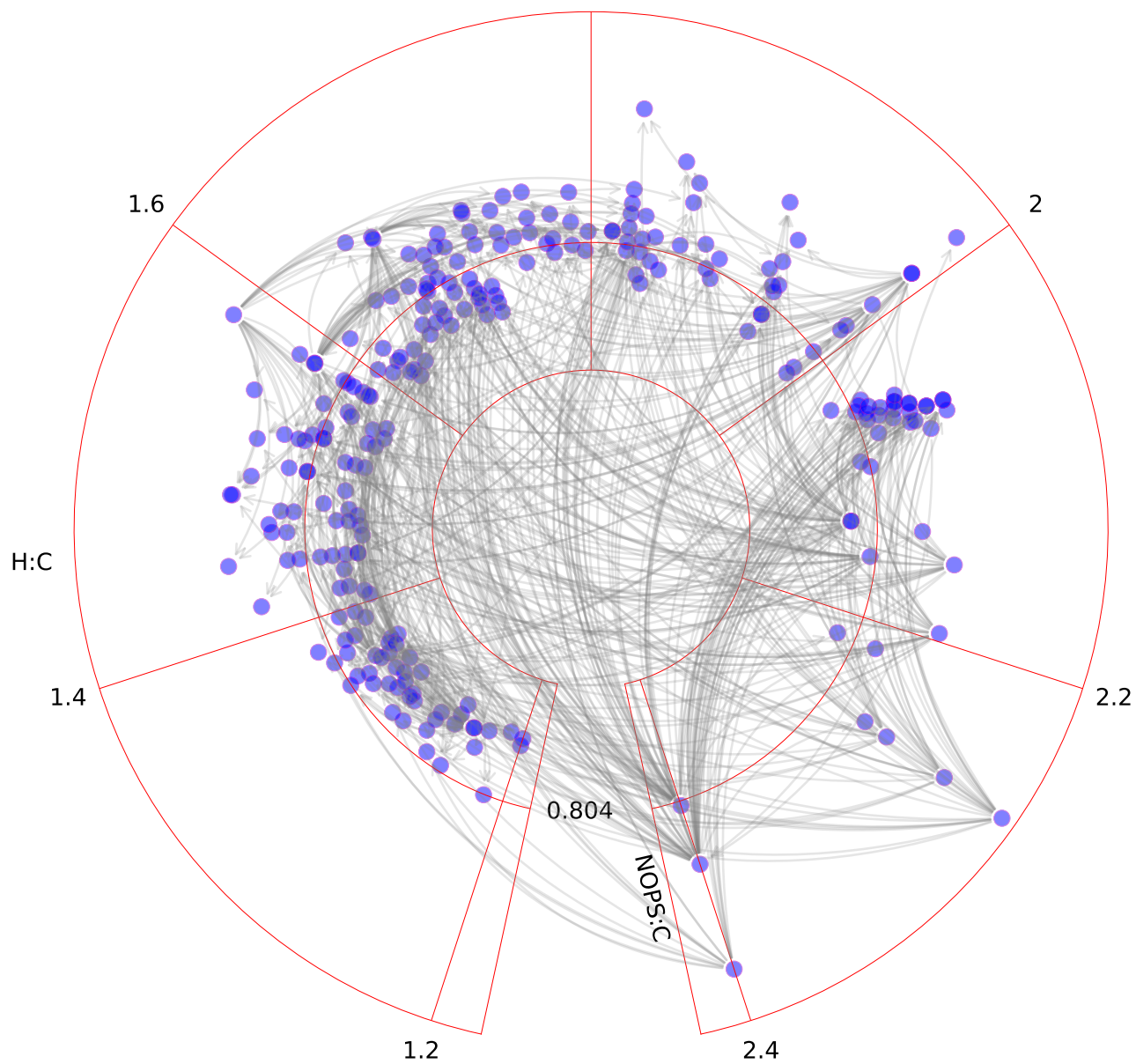

Supplement: Supplement 1 [file media-1.zip › Suppl_File_all_pathways/nolabel/Peptide metabolism.pdf]

# Biotin metabolism

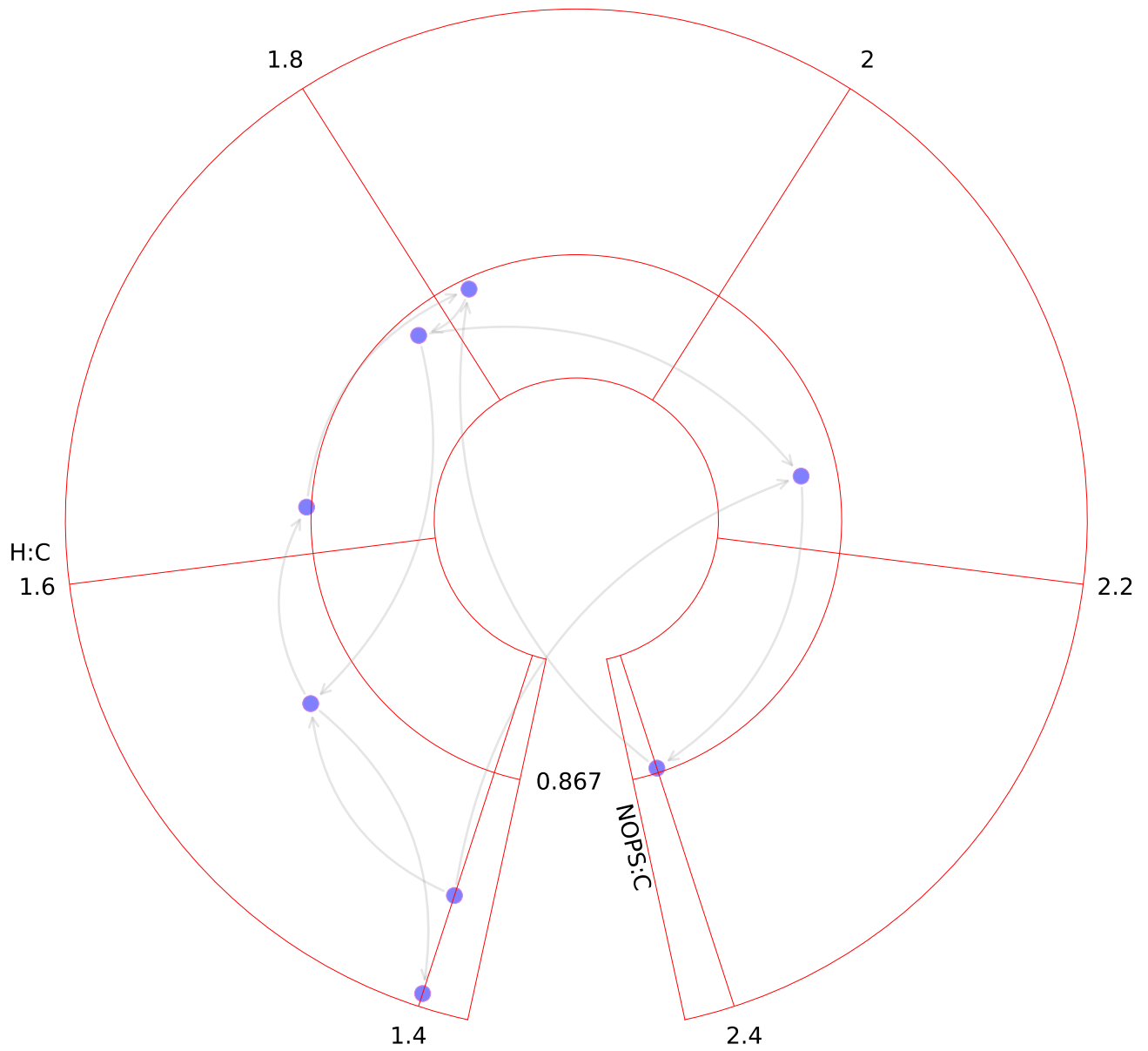

Supplement: Supplement 1 [file media-1.zip › Suppl_File_all_pathways/nolabel/Biotin metabolism.pdf]

# Steroid metabolism

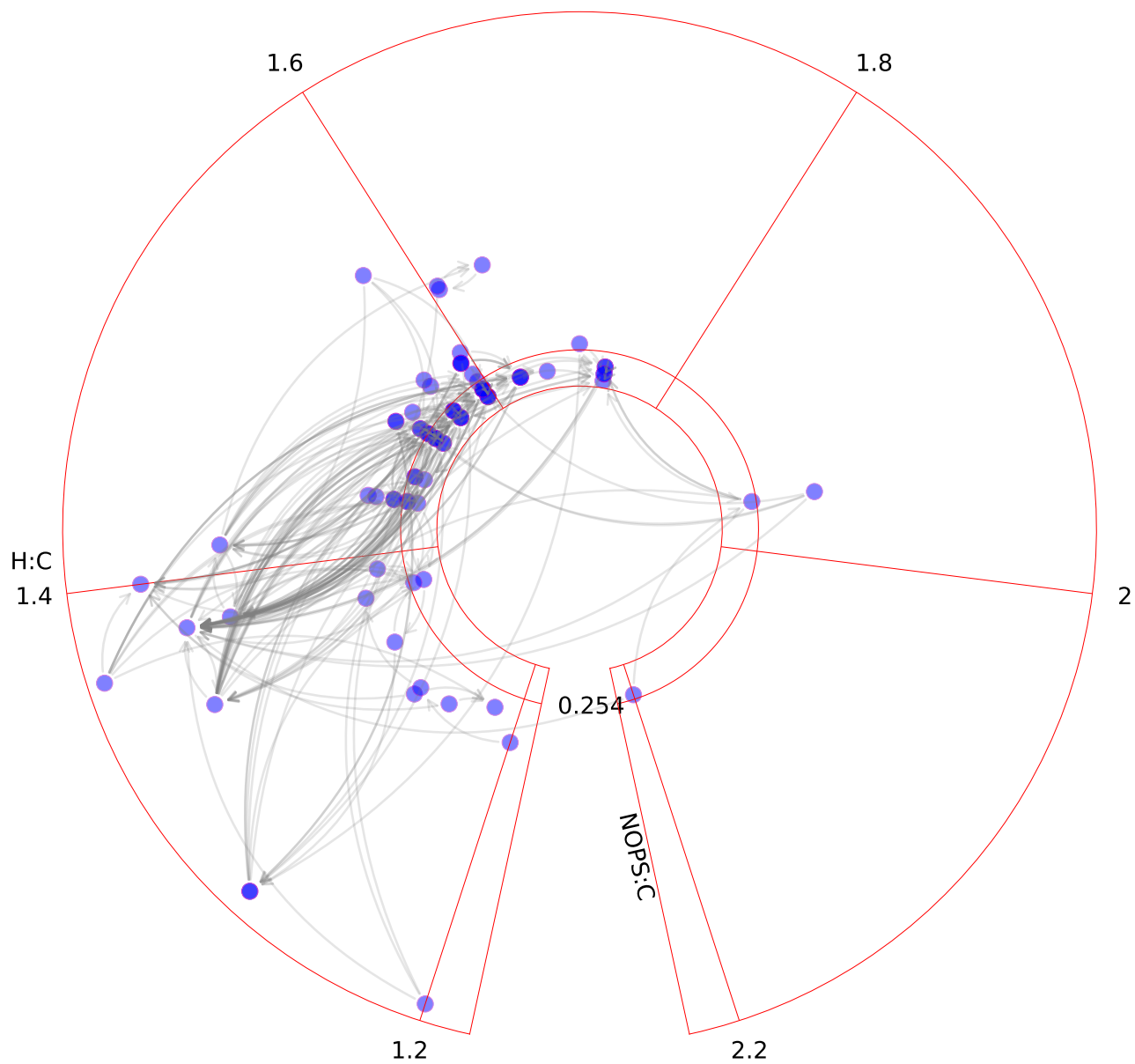

Supplement: Supplement 1 [file media-1.zip › Suppl_File_all_pathways/nolabel/Steroid metabolism.pdf]

# Inositol phosphate metabolism

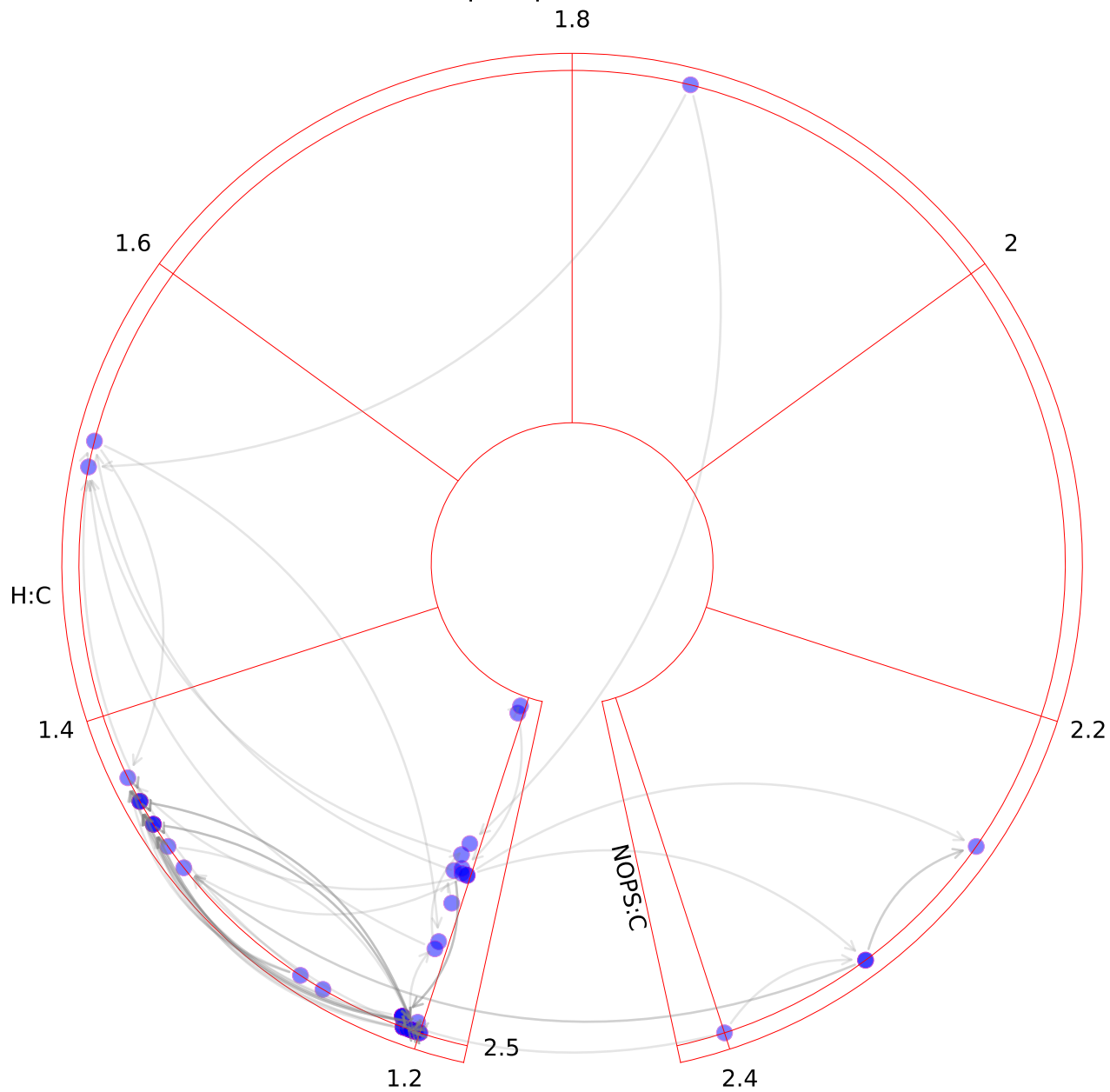

Supplement: Supplement 1 [file media-1.zip › Suppl_File_all_pathways/nolabel/Inositol phosphate metabolism.pdf]

# Arachidonic acid metabolism

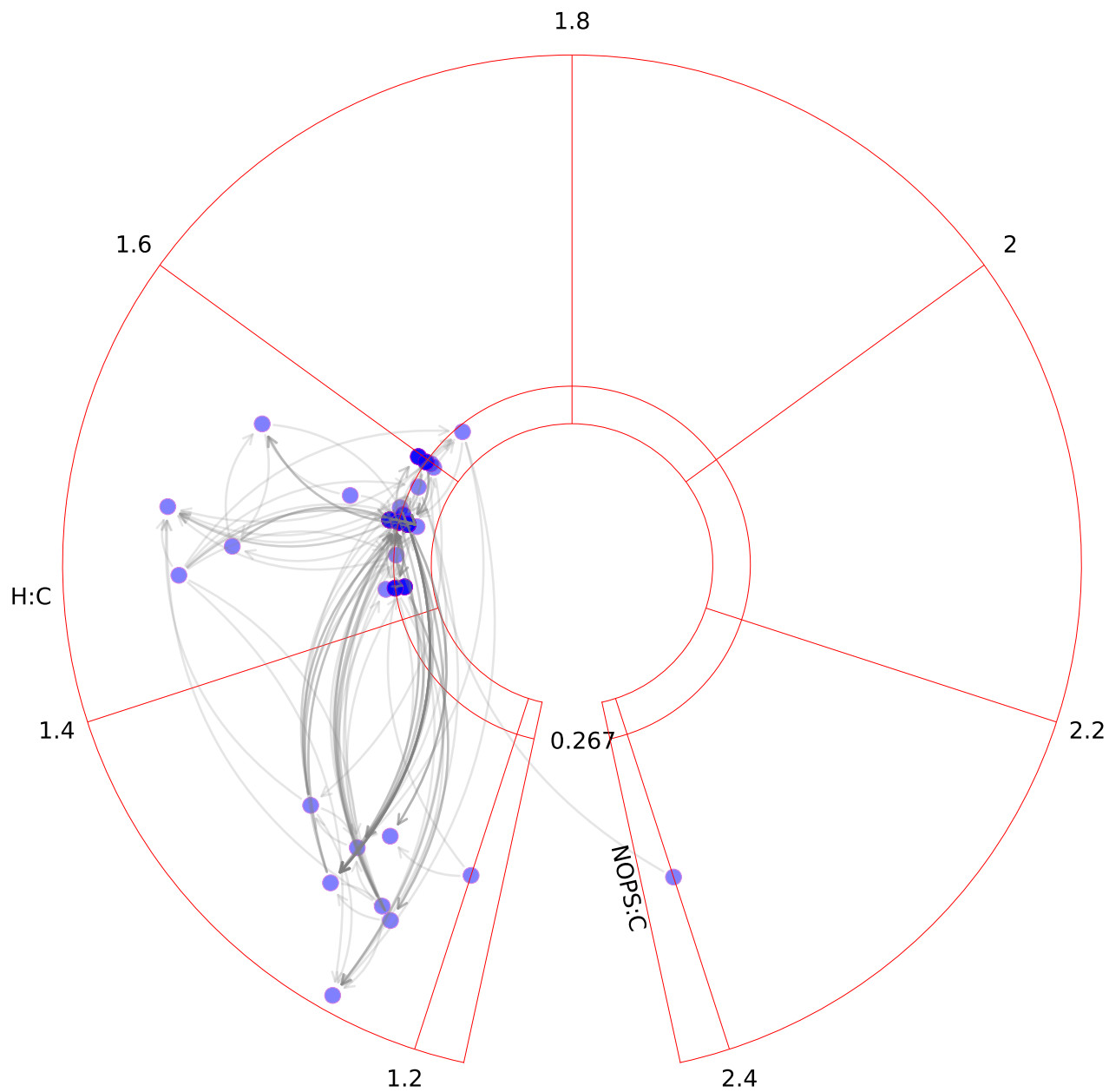

Supplement: Supplement 1 [file media-1.zip › Suppl_File_all_pathways/nolabel/Arachidonic acid metabolism.pdf]

# Cytochrome metabolism

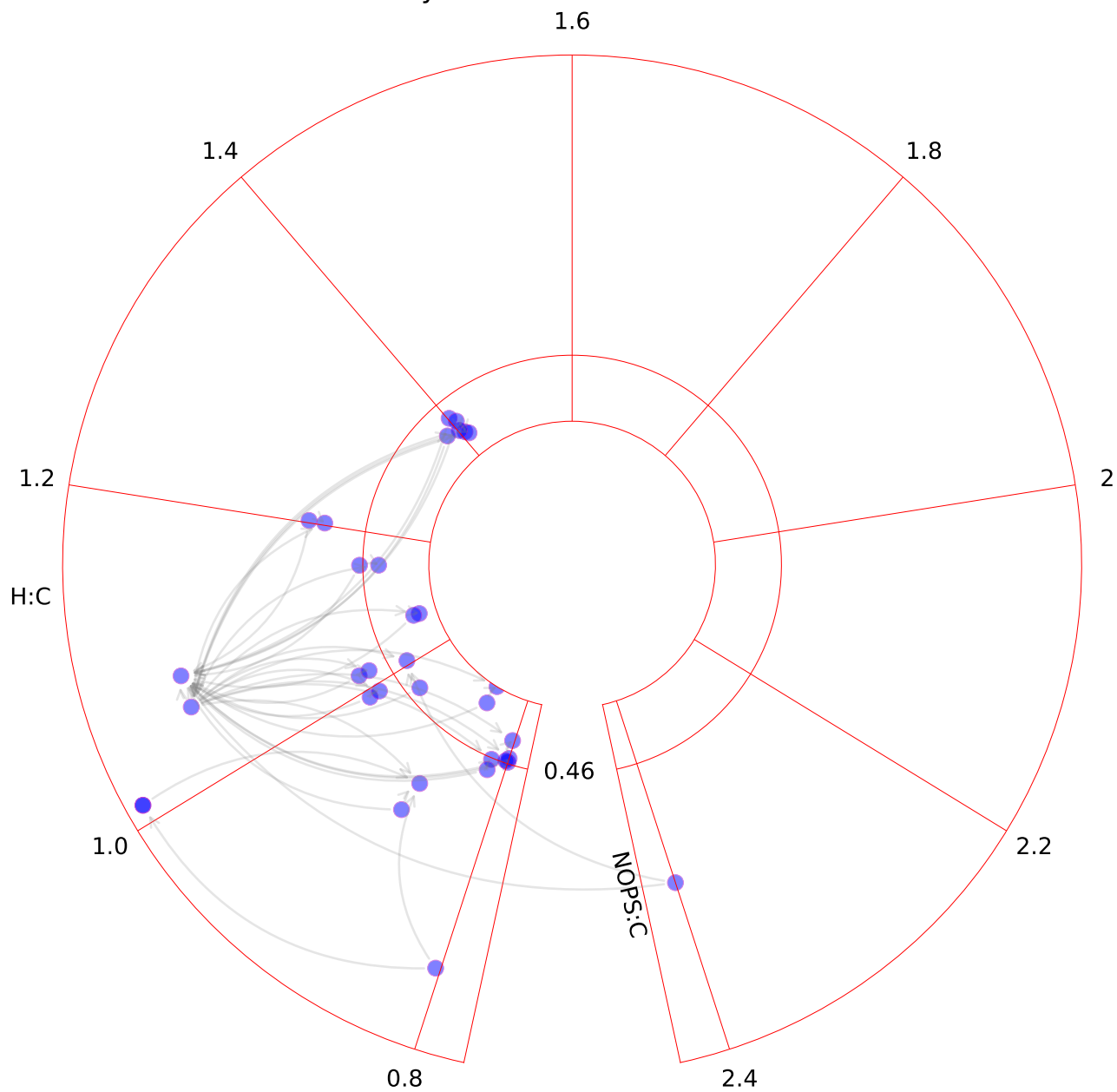

Supplement: Supplement 1 [file media-1.zip › Suppl_File_all_pathways/nolabel/Cytochrome metabolism.pdf]

# Selenoamino acid metabolism

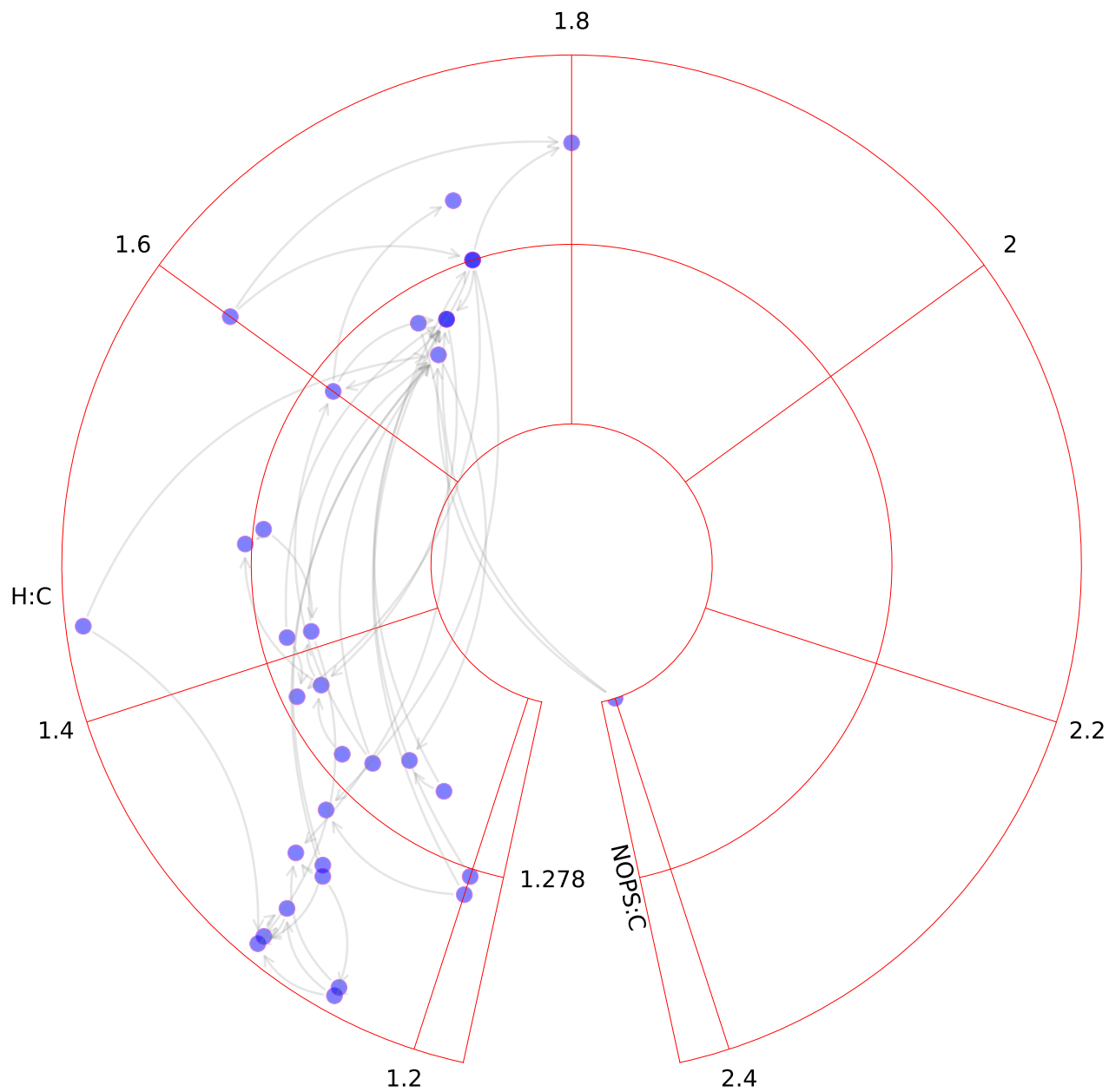

Supplement: Supplement 1 [file media-1.zip › Suppl_File_all_pathways/nolabel/Selenoamino acid metabolism.pdf]

# Phenylalanine metabolism

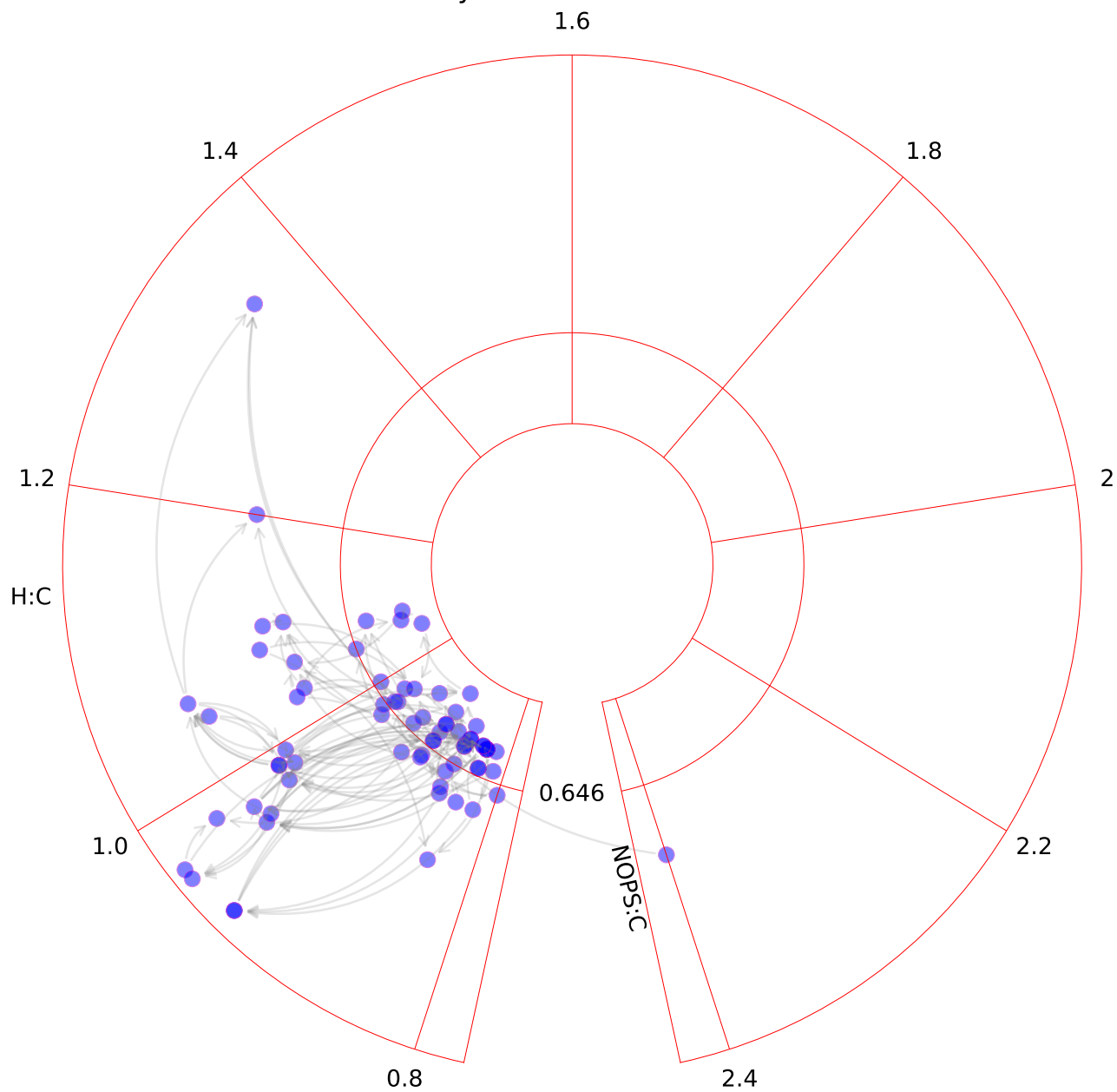

Supplement: Supplement 1 [file media-1.zip › Suppl_File_all_pathways/nolabel/Phenylalanine metabolism.pdf]

# Pyruvate metabolism

1.6

1.4

1.8

2

2.2

2.4

0.8

1.417

NOPS:C

1.2

H:C

1.0

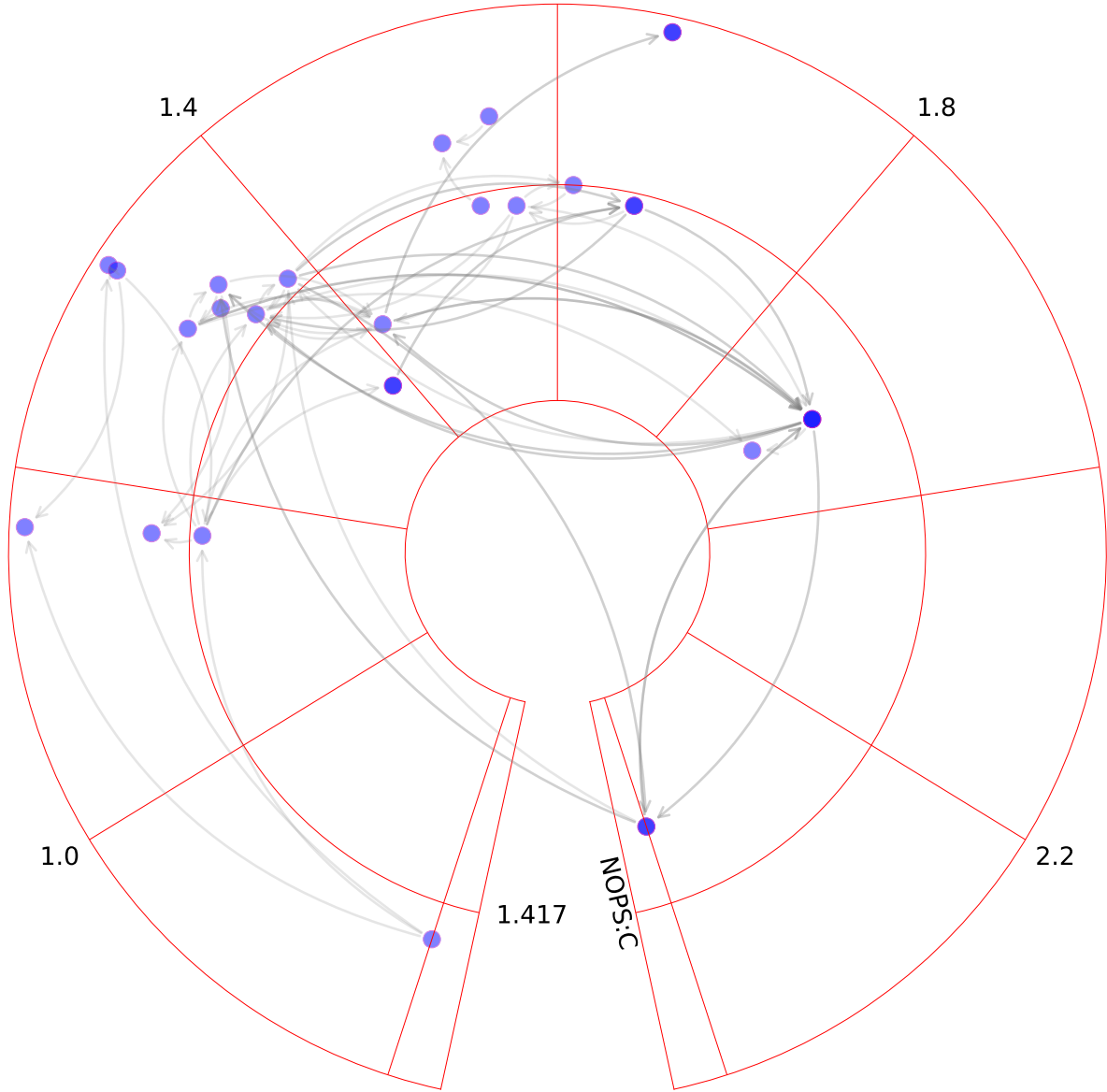

Supplement: Supplement 1 [file media-1.zip › Suppl_File_all_pathways/nolabel/Pyruvate metabolism.pdf]

# Glyoxylate and dicarboxylate metabolism

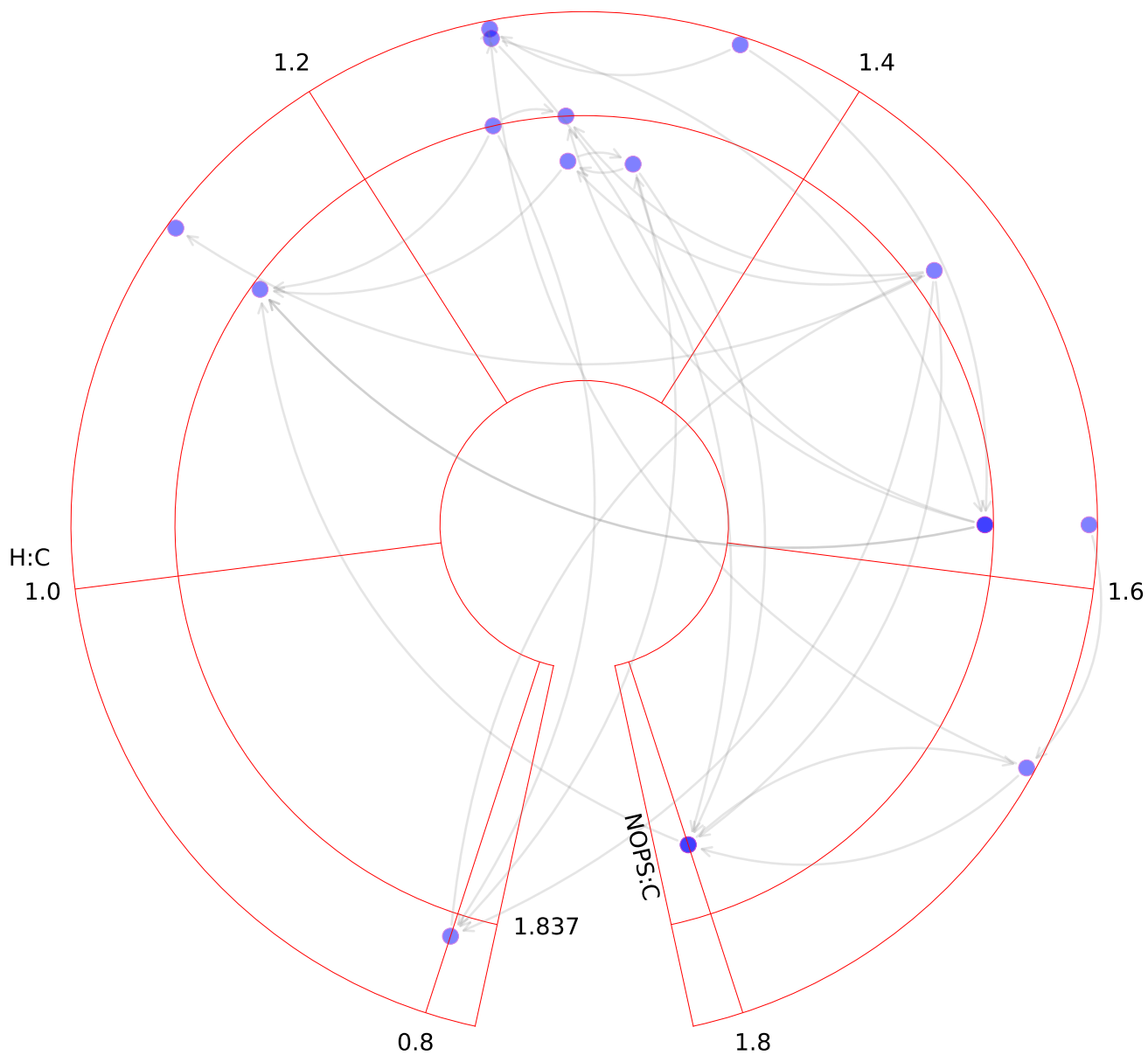

Supplement: Supplement 1 [file media-1.zip › Suppl_File_all_pathways/nolabel/Glyoxylate and dicarboxylate metabolism.pdf]

# Hippurate metabolism

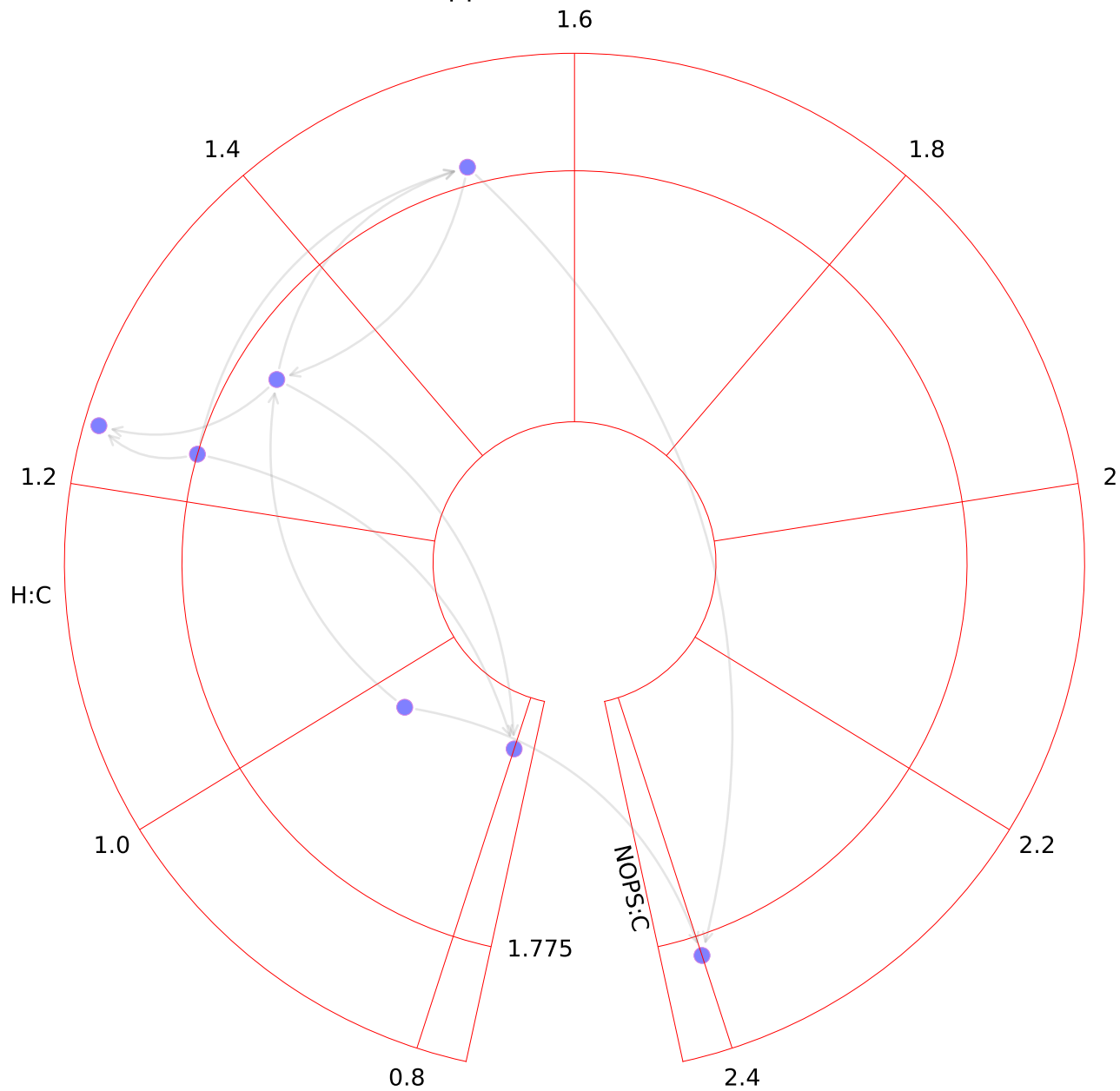

Supplement: Supplement 1 [file media-1.zip › Suppl_File_all_pathways/nolabel/Hippurate metabolism.pdf]

# N-glycan synthesis

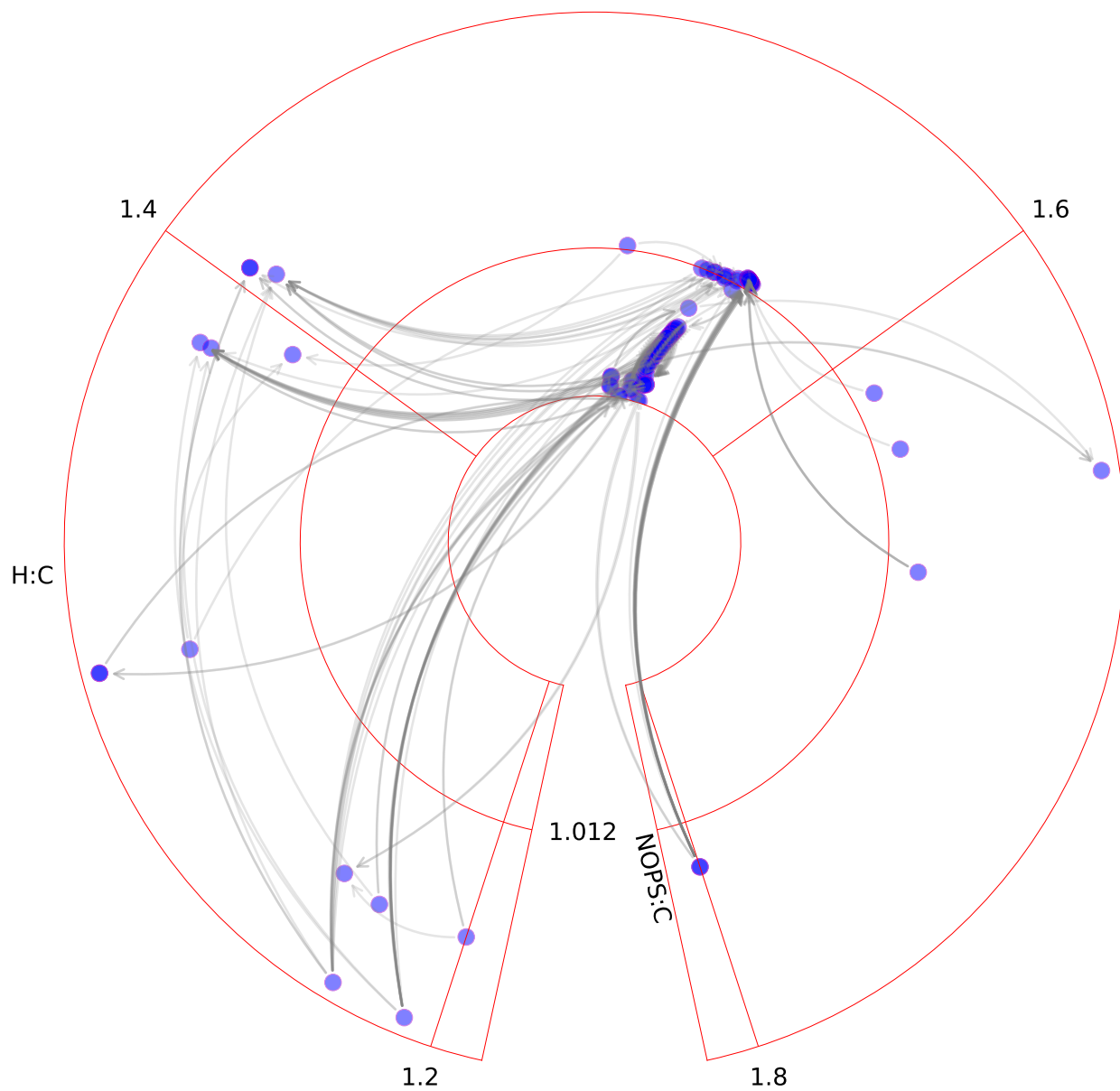

Supplement: Supplement 1 [file media-1.zip › Suppl_File_all_pathways/nolabel/N-glycan synthesis.pdf]

# Vitamin A metabolism

1.8

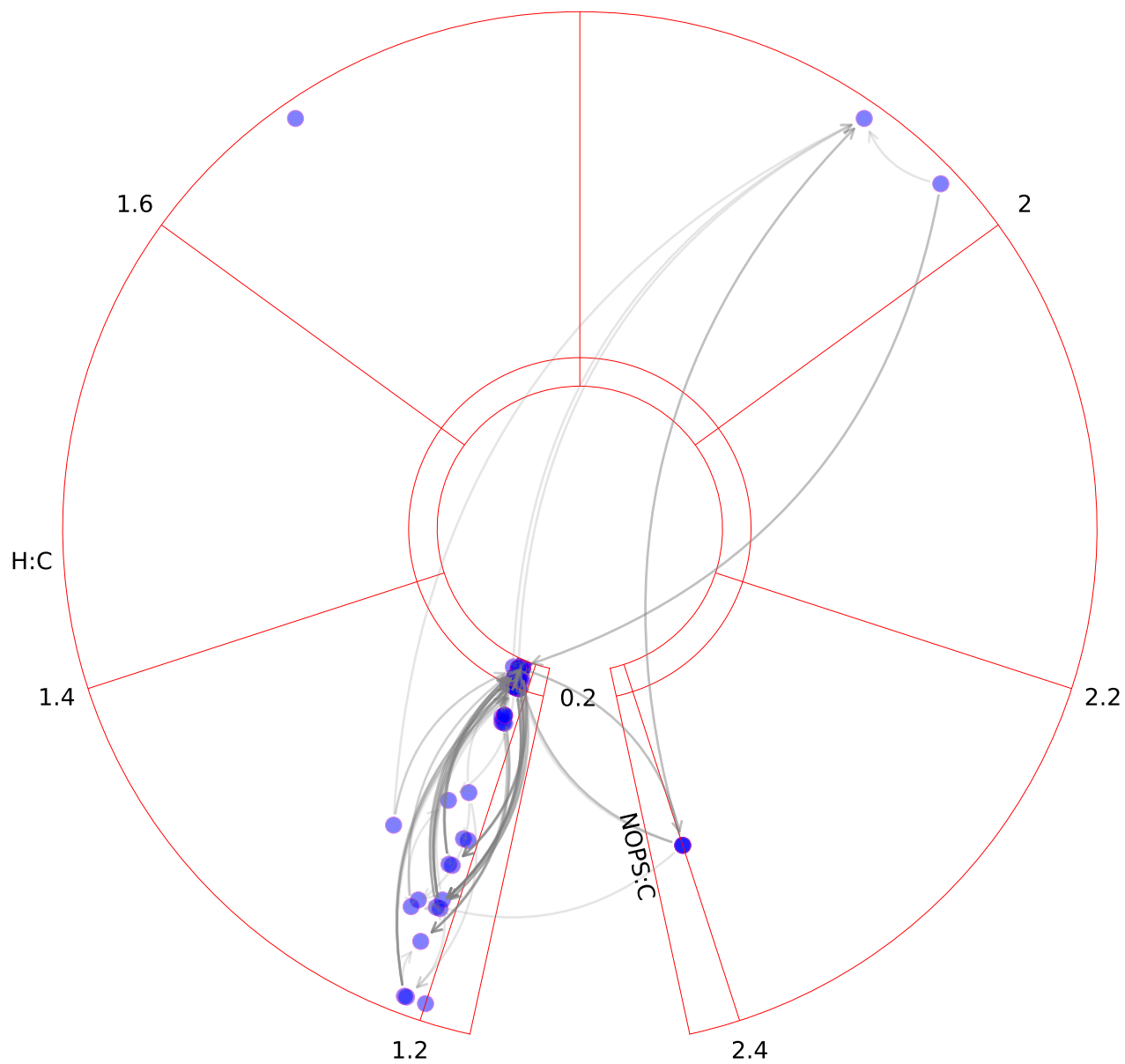

Supplement: Supplement 1 [file media-1.zip › Suppl_File_all_pathways/nolabel/Vitamin A metabolism.pdf]

# Butanoate metabolism

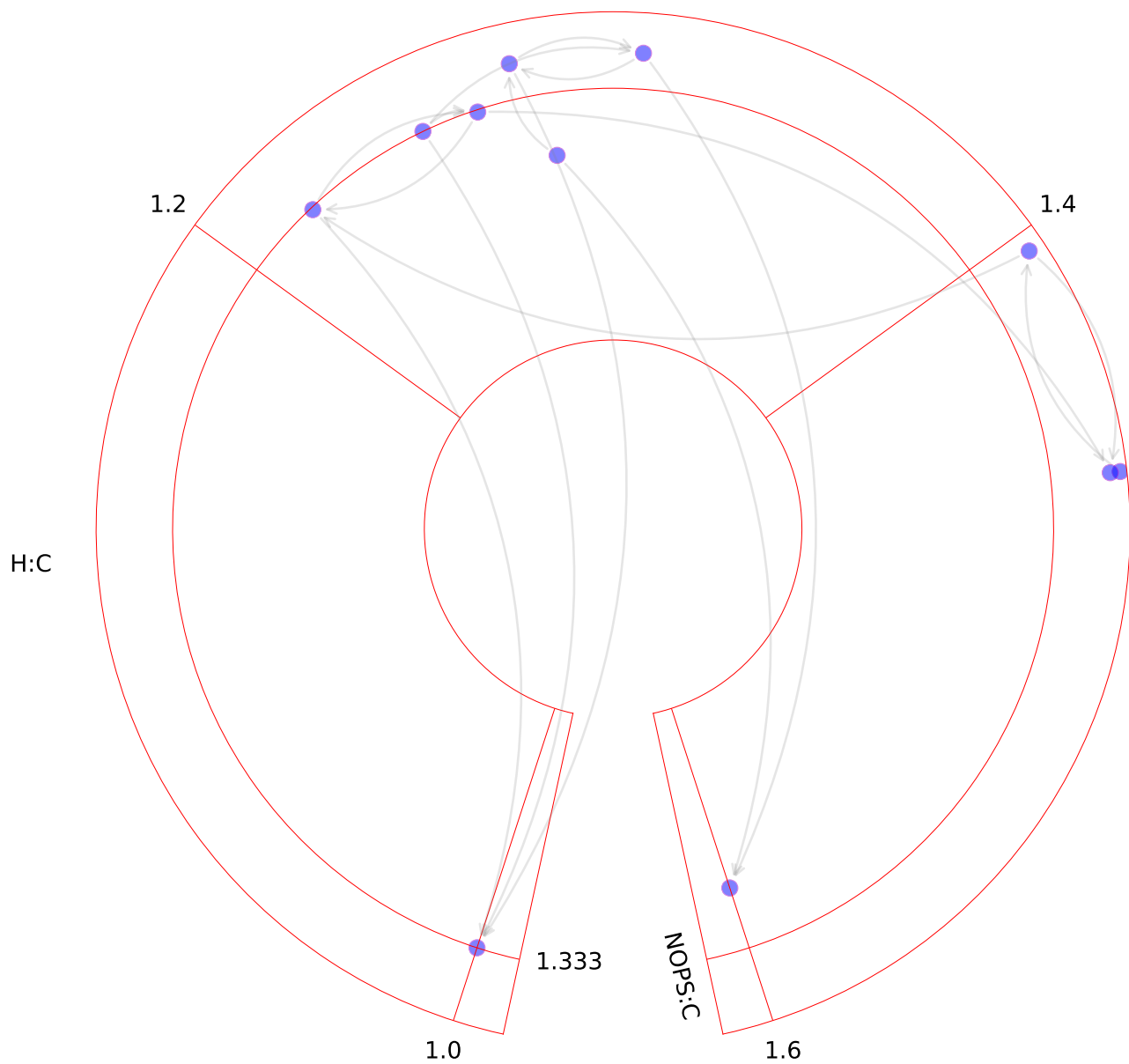

Supplement: Supplement 1 [file media-1.zip › Suppl_File_all_pathways/nolabel/Butanoate metabolism.pdf]

# Pyrimidine catabolism

1.6

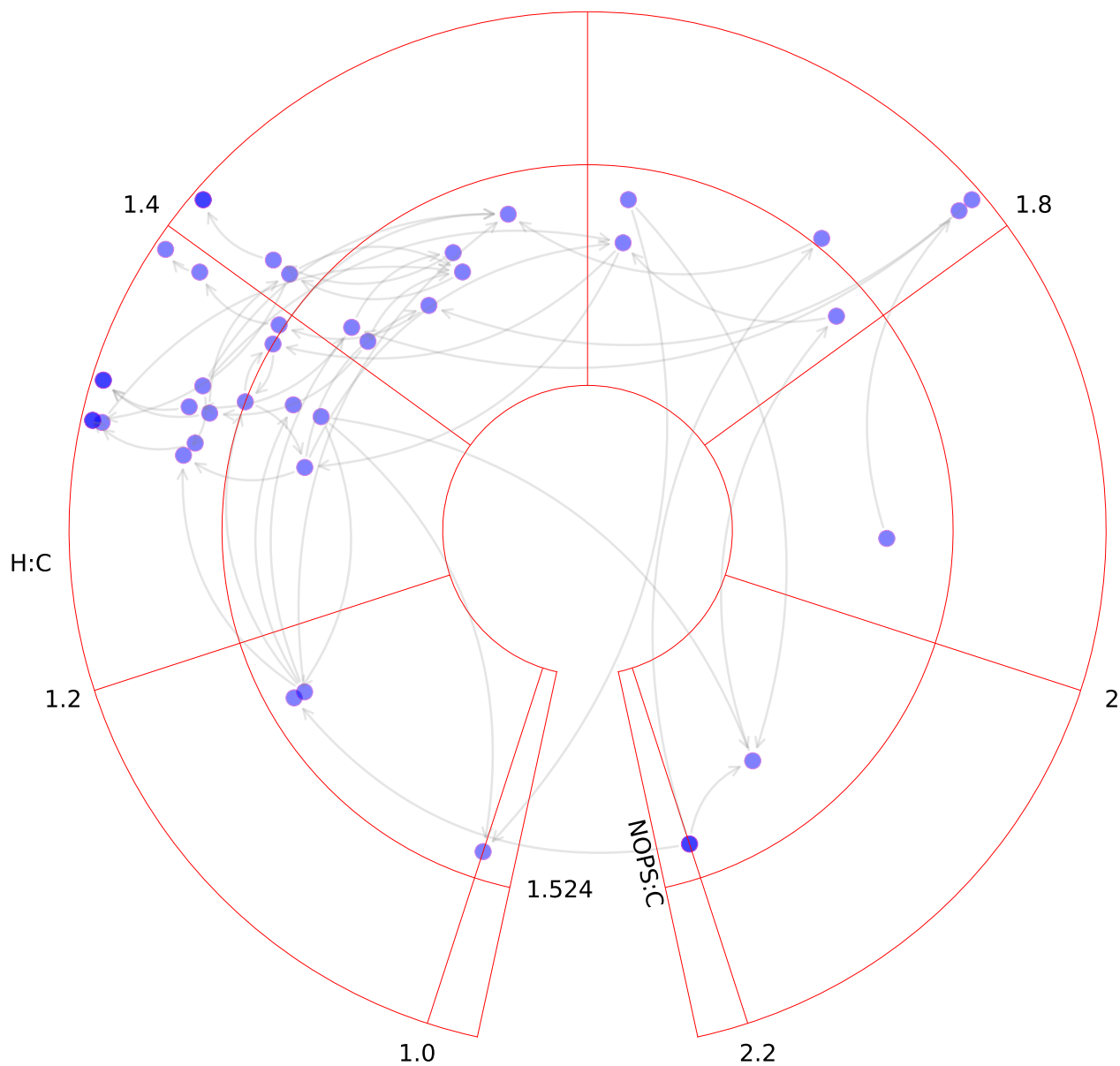

Supplement: Supplement 1 [file media-1.zip › Suppl_File_all_pathways/nolabel/Pyrimidine catabolism.pdf]

# Chondroitin synthesis

1.4

H:C

NOPS:C

1.563

1.2

1.6

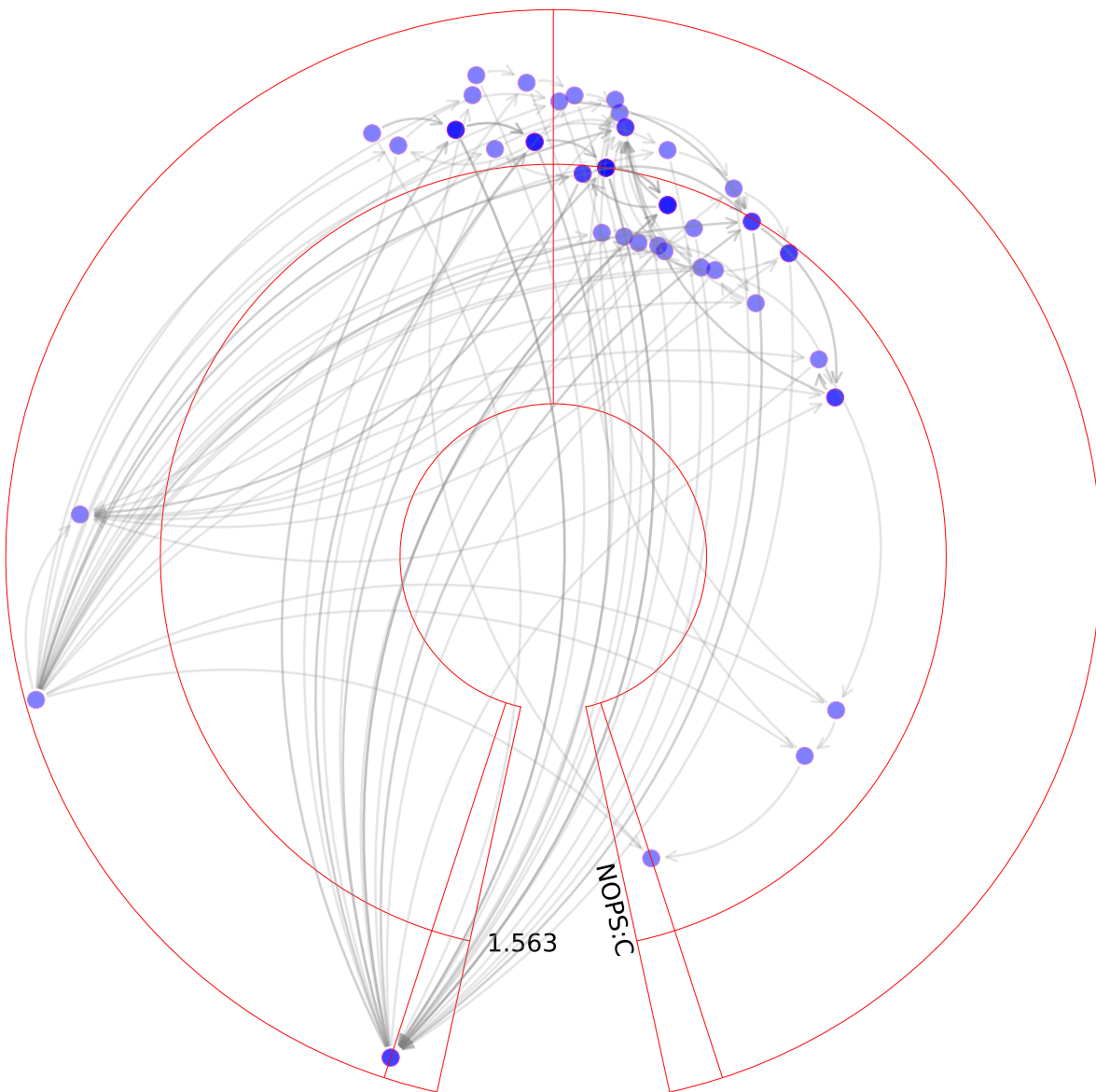

Supplement: Supplement 1 [file media-1.zip › Suppl_File_all_pathways/nolabel/Chondroitin synthesis.pdf]

# Fatty acid oxidation

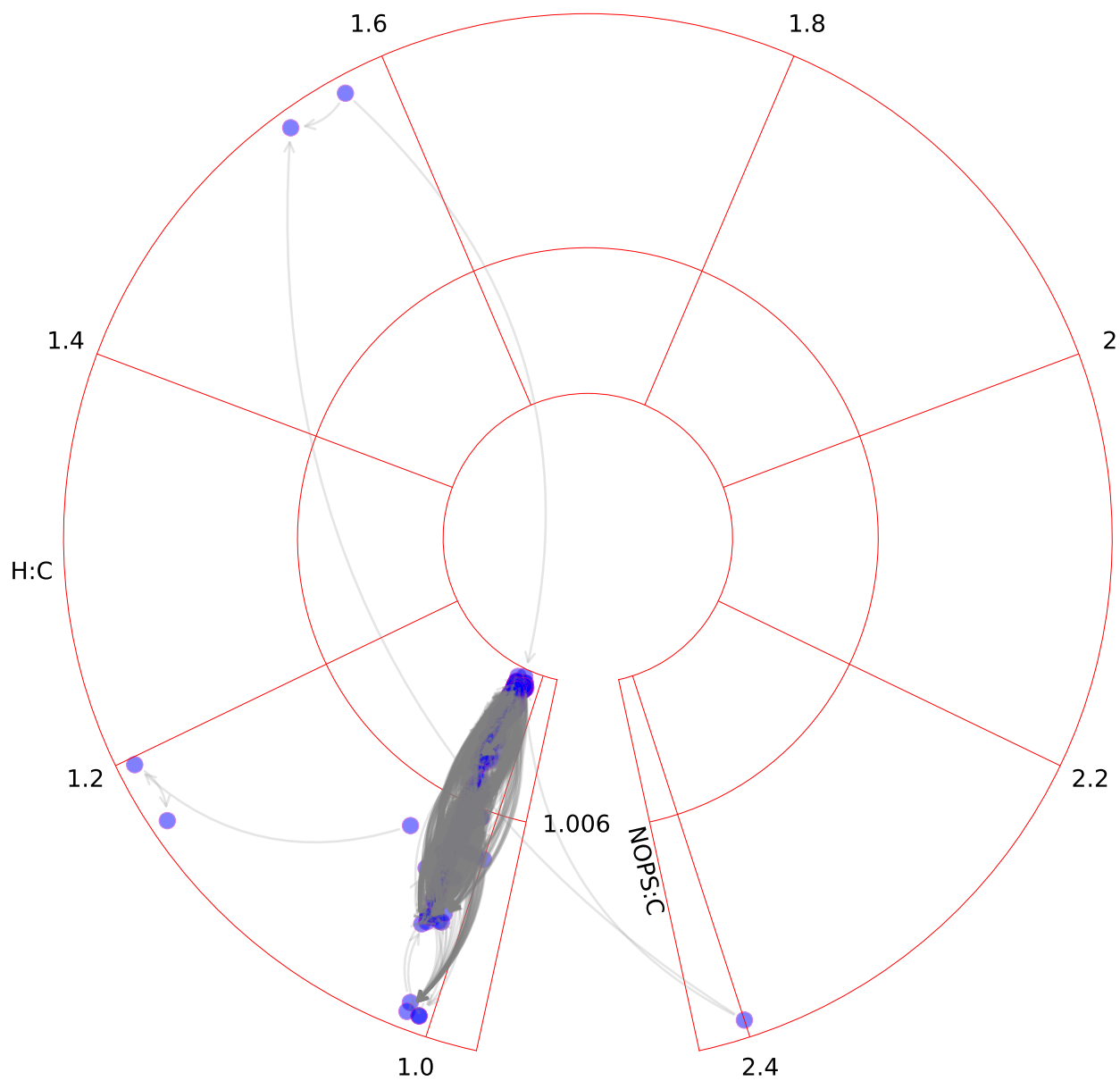

Supplement: Supplement 1 [file media-1.zip › Suppl_File_all_pathways/nolabel/Fatty acid oxidation.pdf]

# Vitamin B2 metabolism

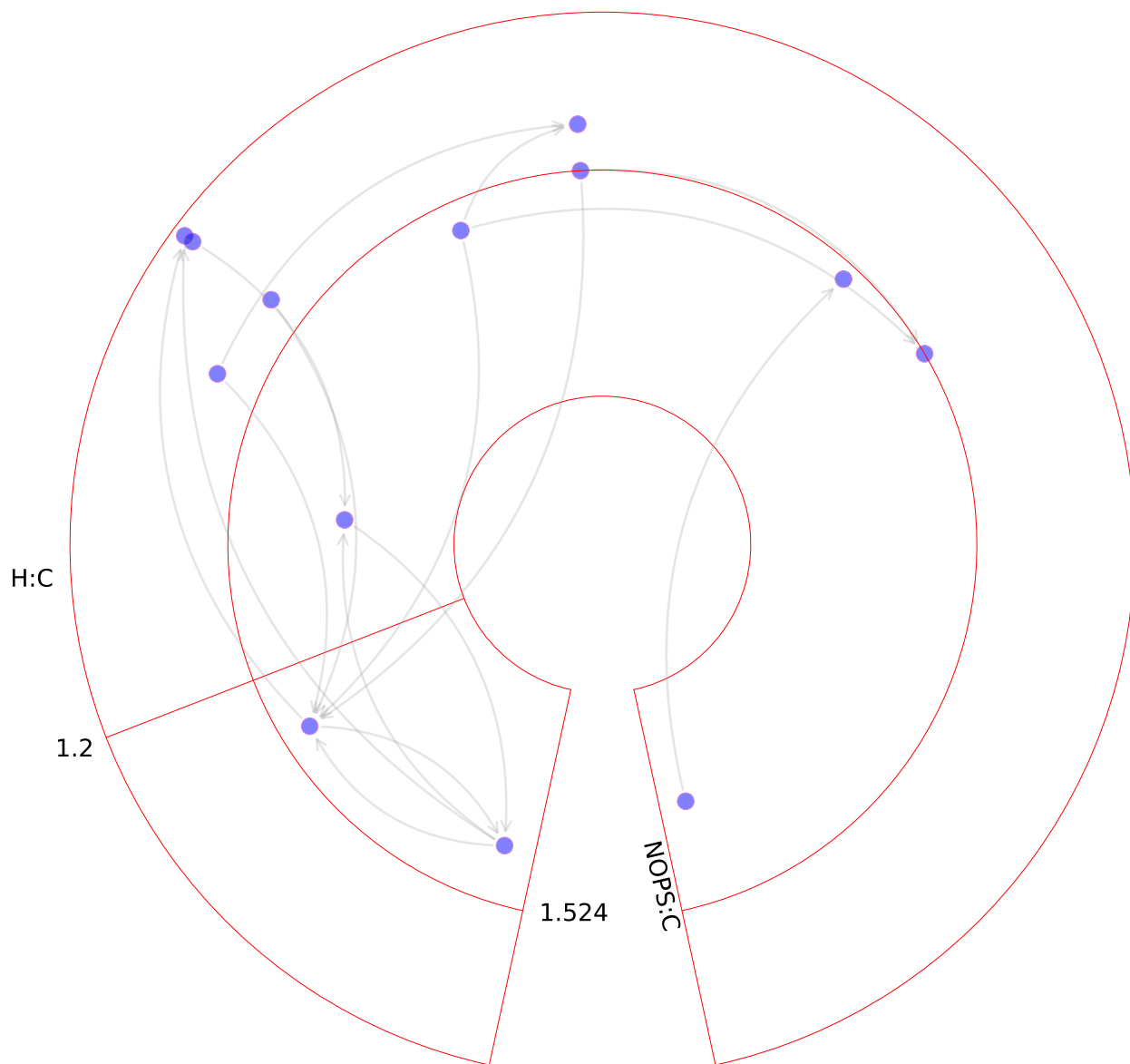

Supplement: Supplement 1 [file media-1.zip › Suppl_File_all_pathways/nolabel/Vitamin B2 metabolism.pdf]

# Chondroitin sulfate degradation

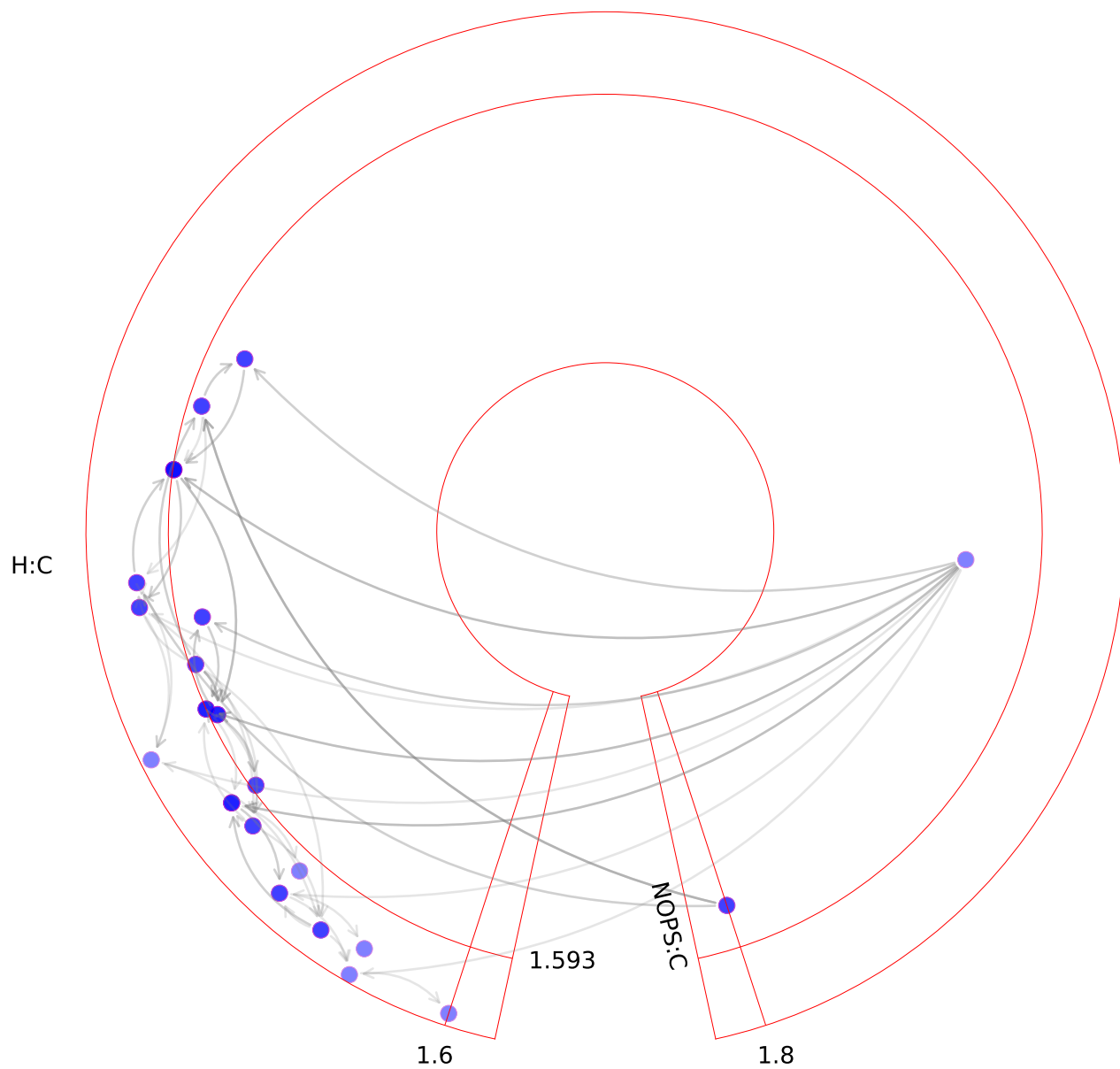

Supplement: Supplement 1 [file media-1.zip › Suppl_File_all_pathways/nolabel/Chondroitin sulfate degradation.pdf]

Heme degradation

H:C

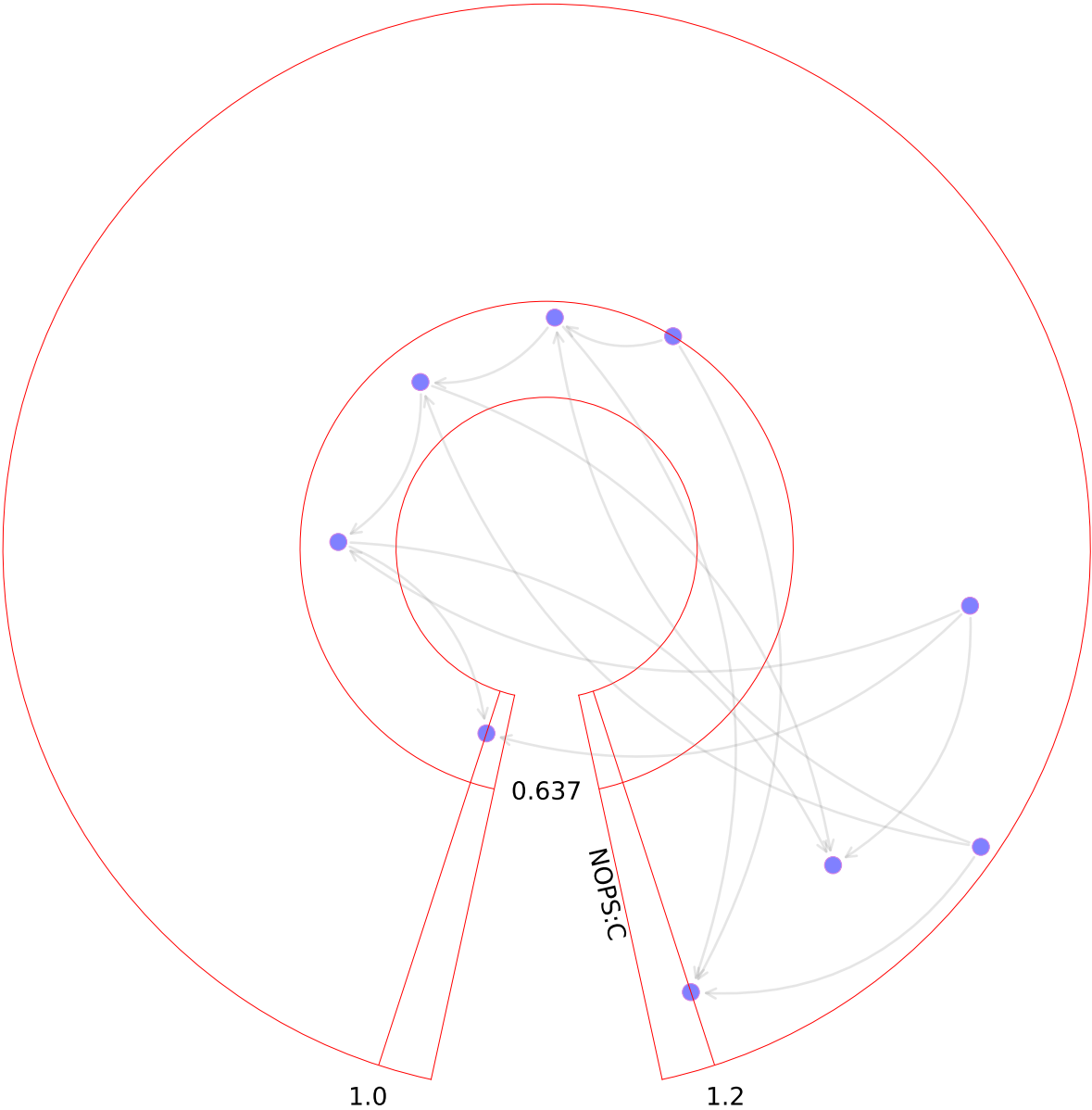

Supplement: Supplement 1 [file media-1.zip › Suppl_File_all_pathways/nolabel/Heme degradation.pdf]

# Propanoate metabolism

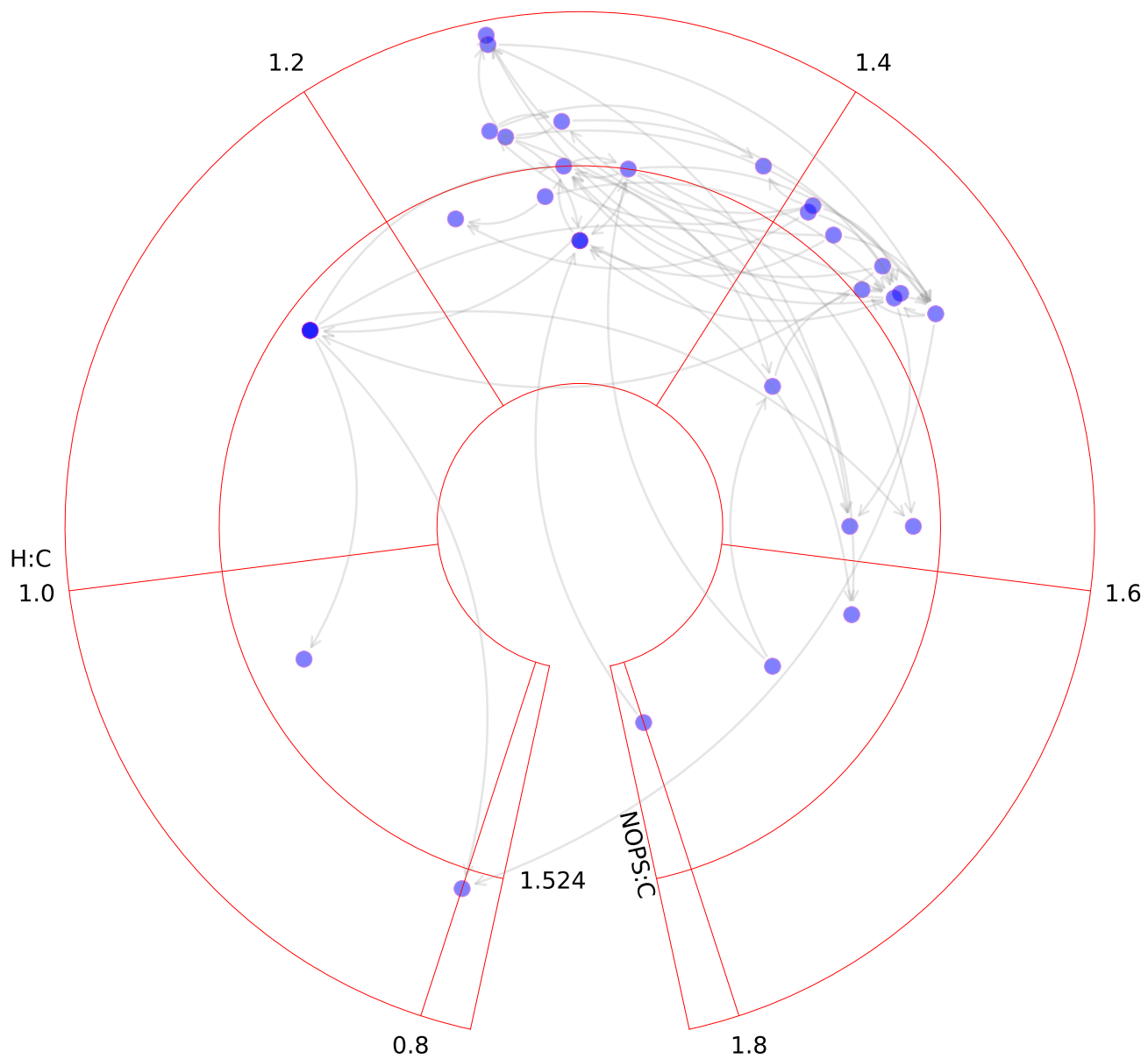

Supplement: Supplement 1 [file media-1.zip › Suppl_File_all_pathways/nolabel/Propanoate metabolism.pdf]

# Galactose metabolism

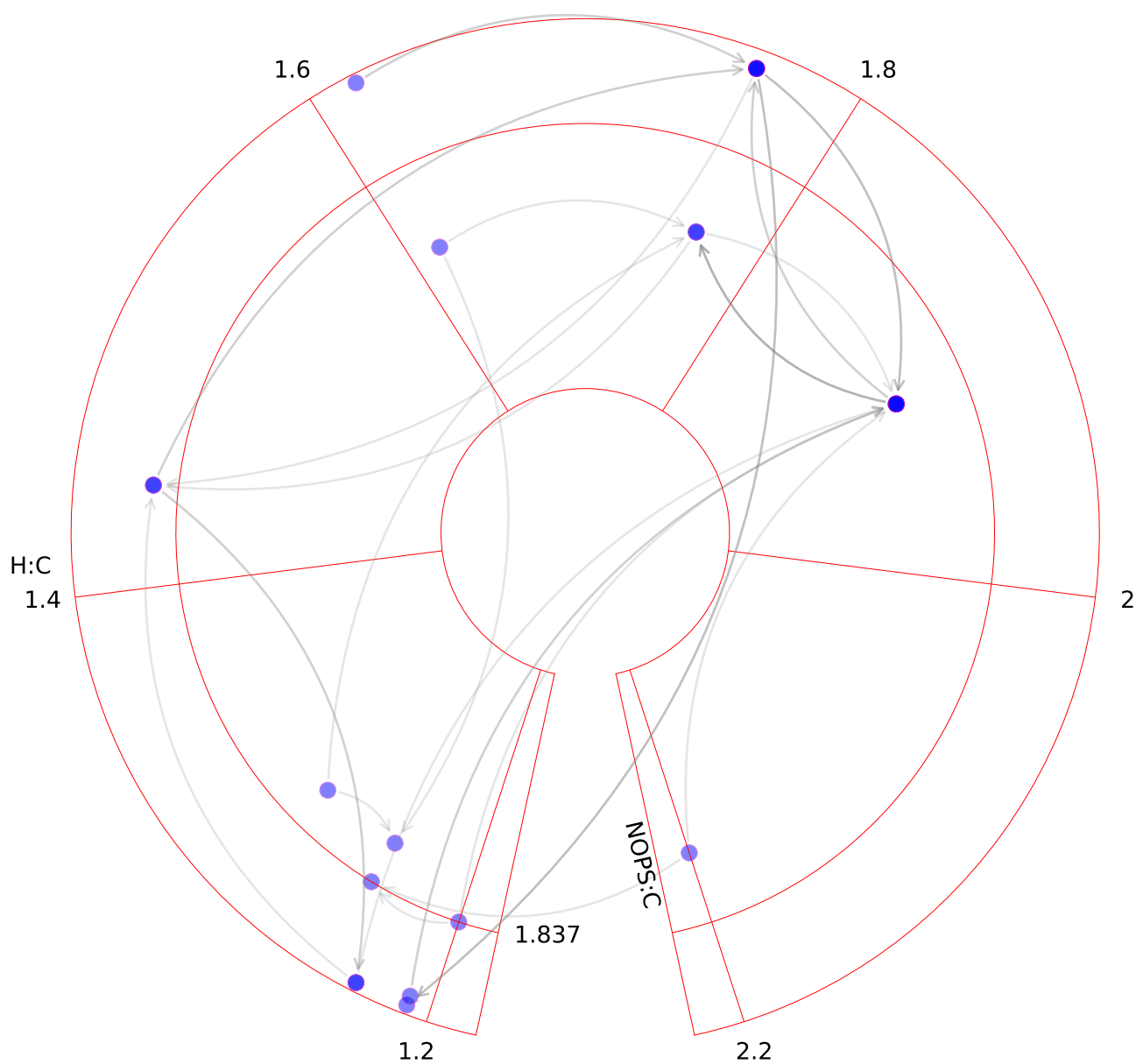

Supplement: Supplement 1 [file media-1.zip › Suppl_File_all_pathways/nolabel/Galactose metabolism.pdf]

# Pentose phosphate pathway

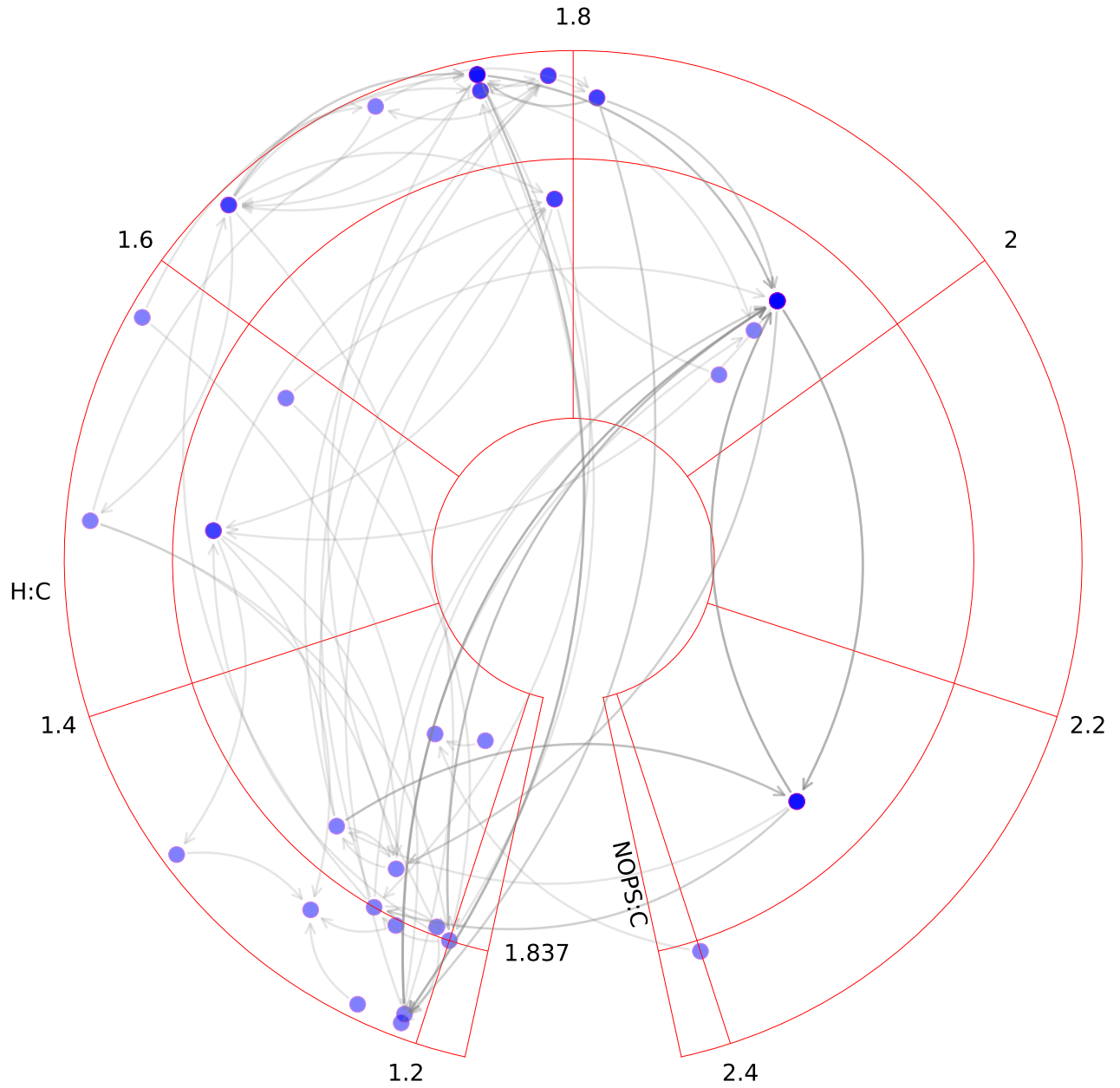

Supplement: Supplement 1 [file media-1.zip › Suppl_File_all_pathways/nolabel/Pentose phosphate pathway.pdf]

# Nucleotide interconversion

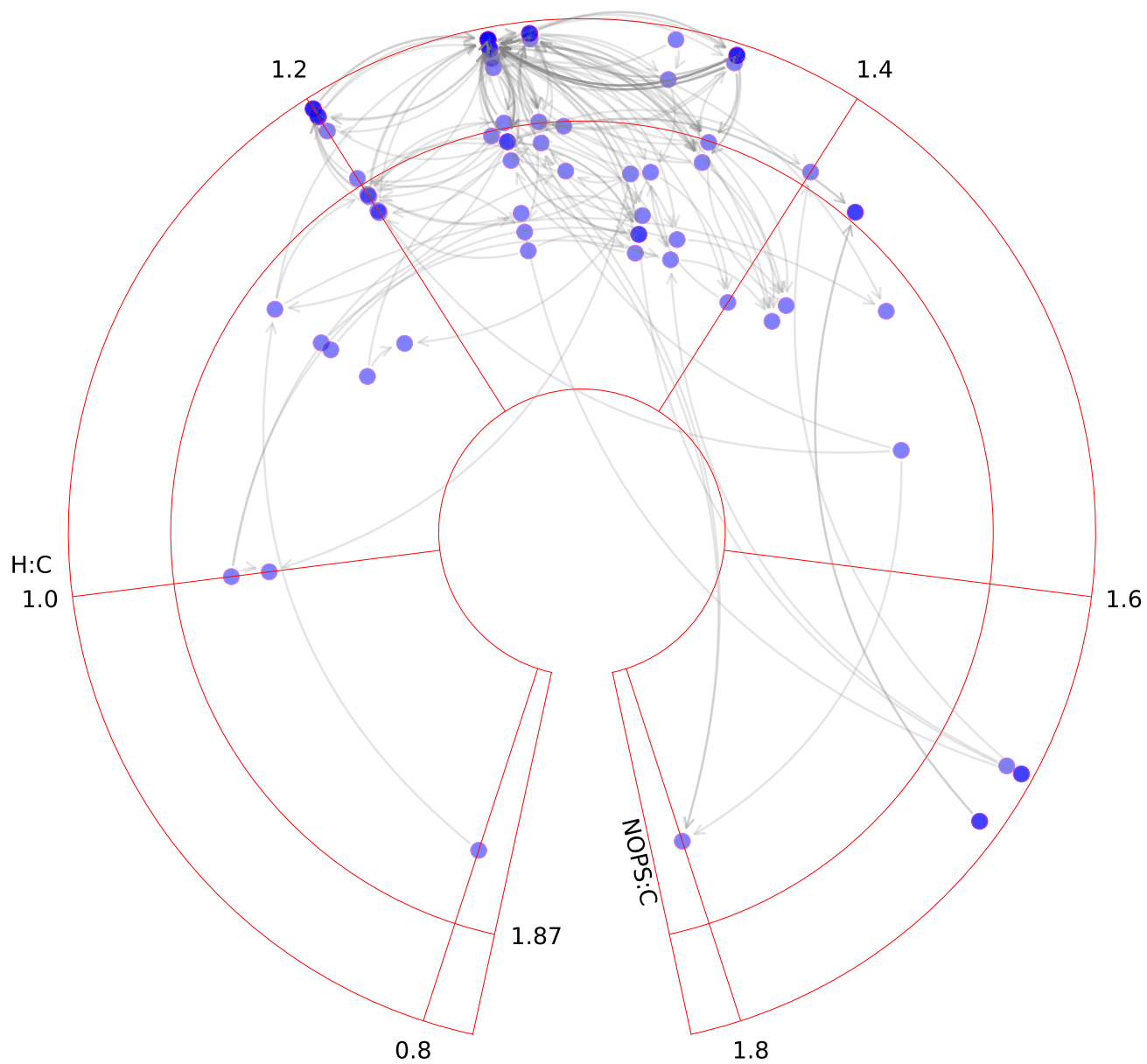

Supplement: Supplement 1 [file media-1.zip › Suppl_File_all_pathways/nolabel/Nucleotide interconversion.pdf]

# Alanine and aspartate metabolism

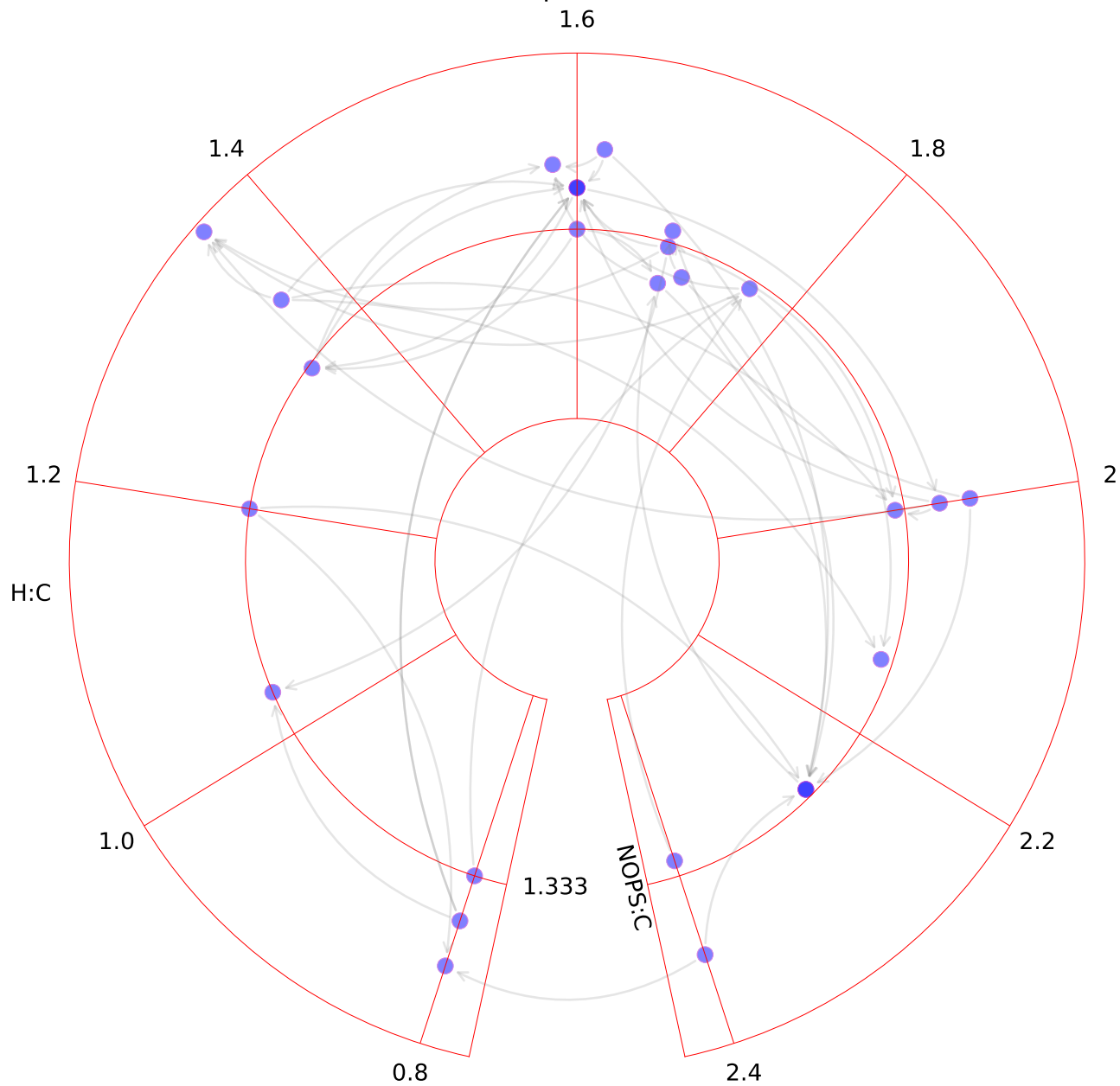

Supplement: Supplement 1 [file media-1.zip › Suppl_File_all_pathways/nolabel/Alanine and aspartate metabolism.pdf]

Dietary fiber binding  
1.6

H:C

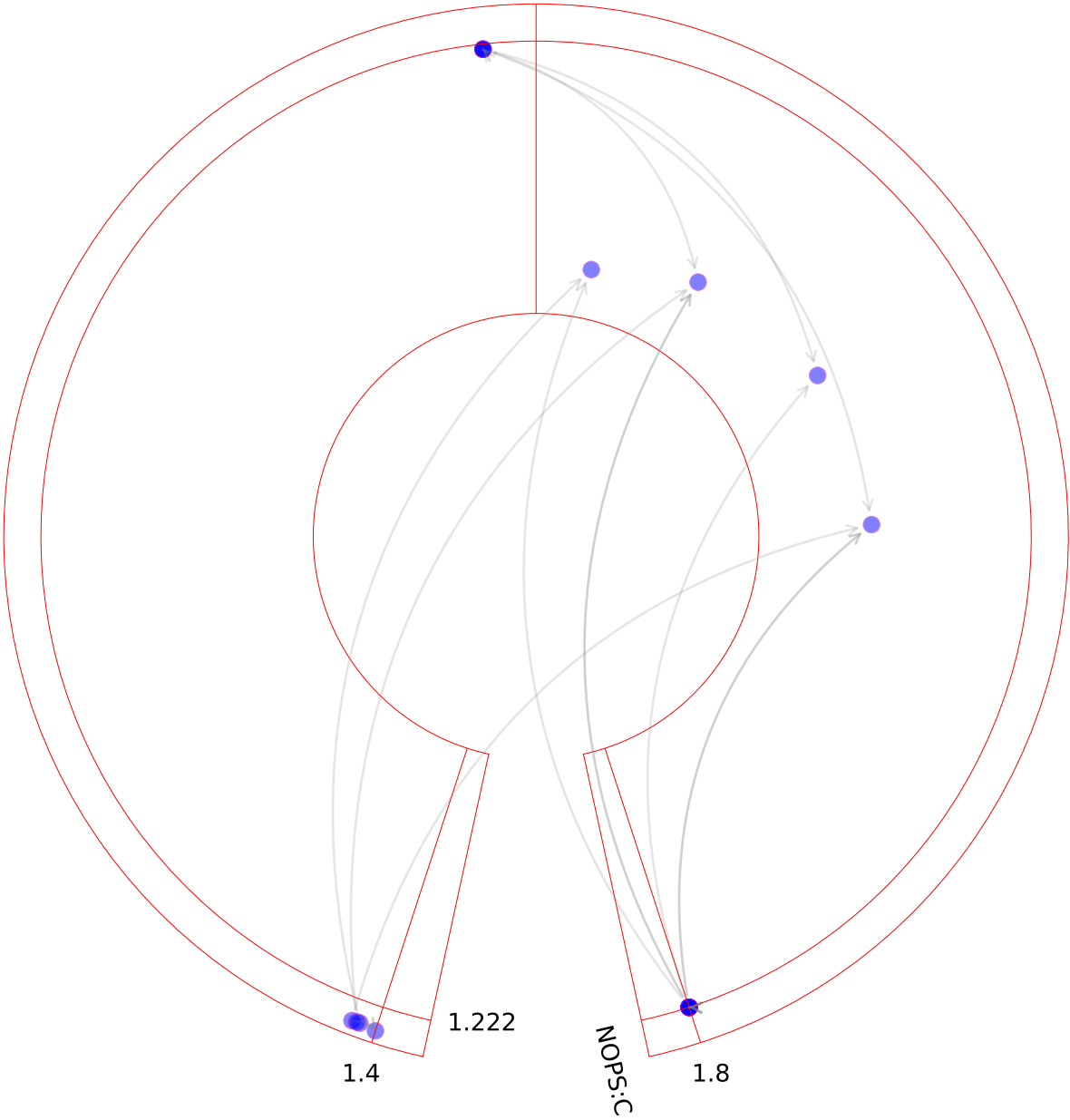

Supplement: Supplement 1 [file media-1.zip › Suppl_File_all_pathways/nolabel/Dietary fiber binding.pdf]

# CoA catabolism

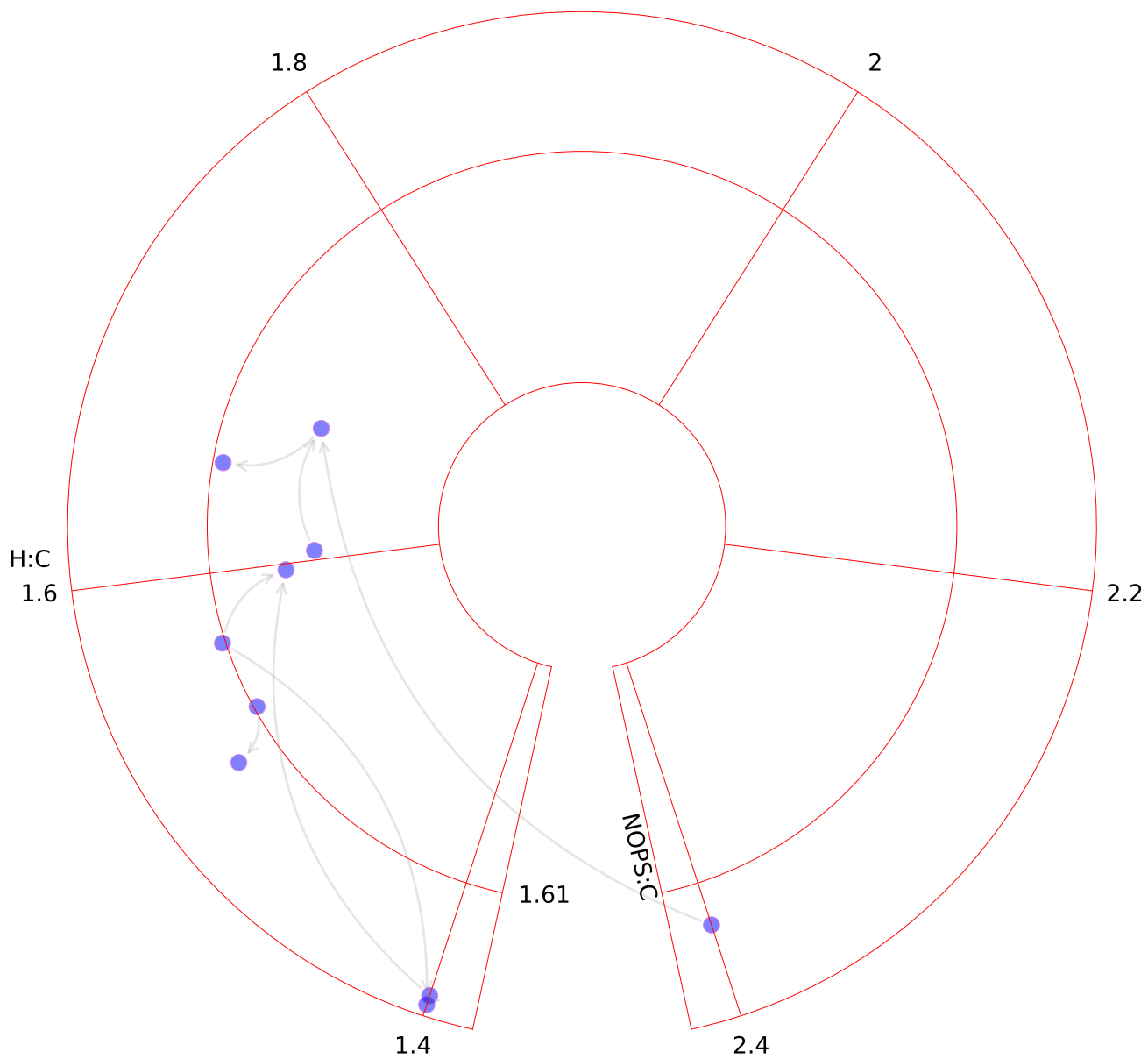

Supplement: Supplement 1 [file media-1.zip › Suppl_File_all_pathways/nolabel/CoA catabolism.pdf]

# Limonene and pinene degradation

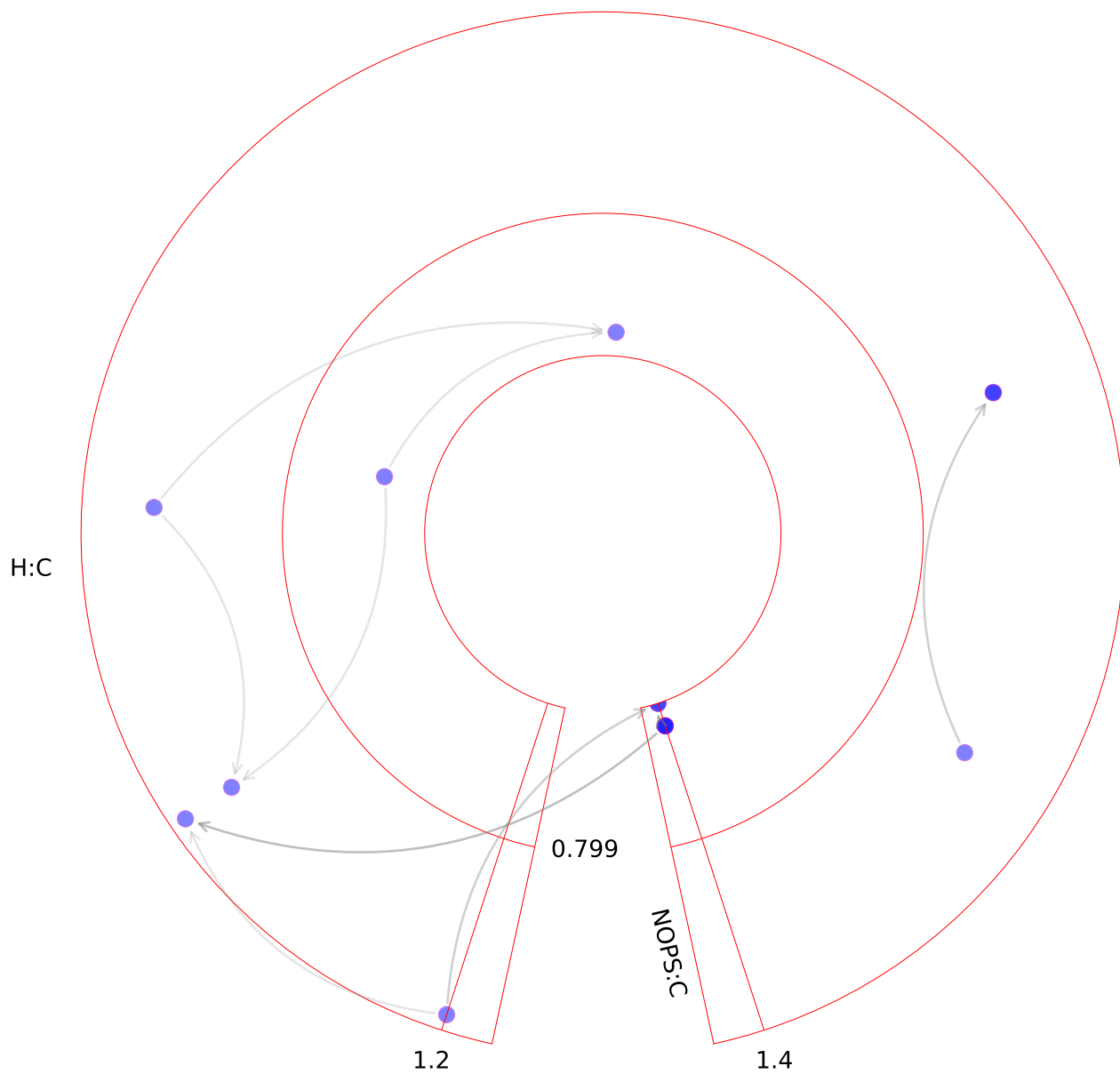

Supplement: Supplement 1 [file media-1.zip › Suppl_File_all_pathways/nolabel/Limonene and pinene degradation.pdf]

# Nucleotide sugar metabolism

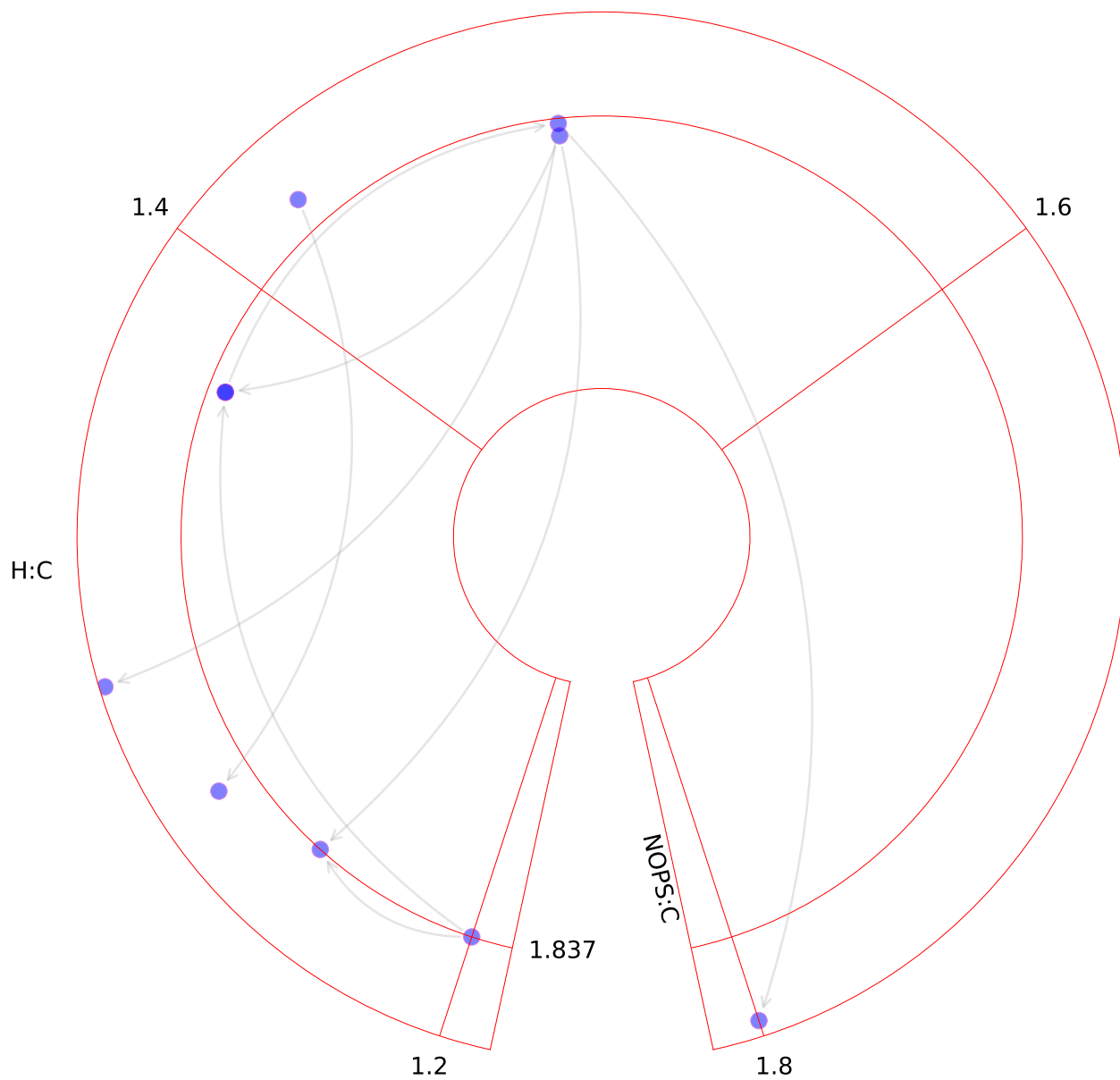

Supplement: Supplement 1 [file media-1.zip › Suppl_File_all_pathways/nolabel/Nucleotide sugar metabolism.pdf]

# Fructose and mannose metabolism

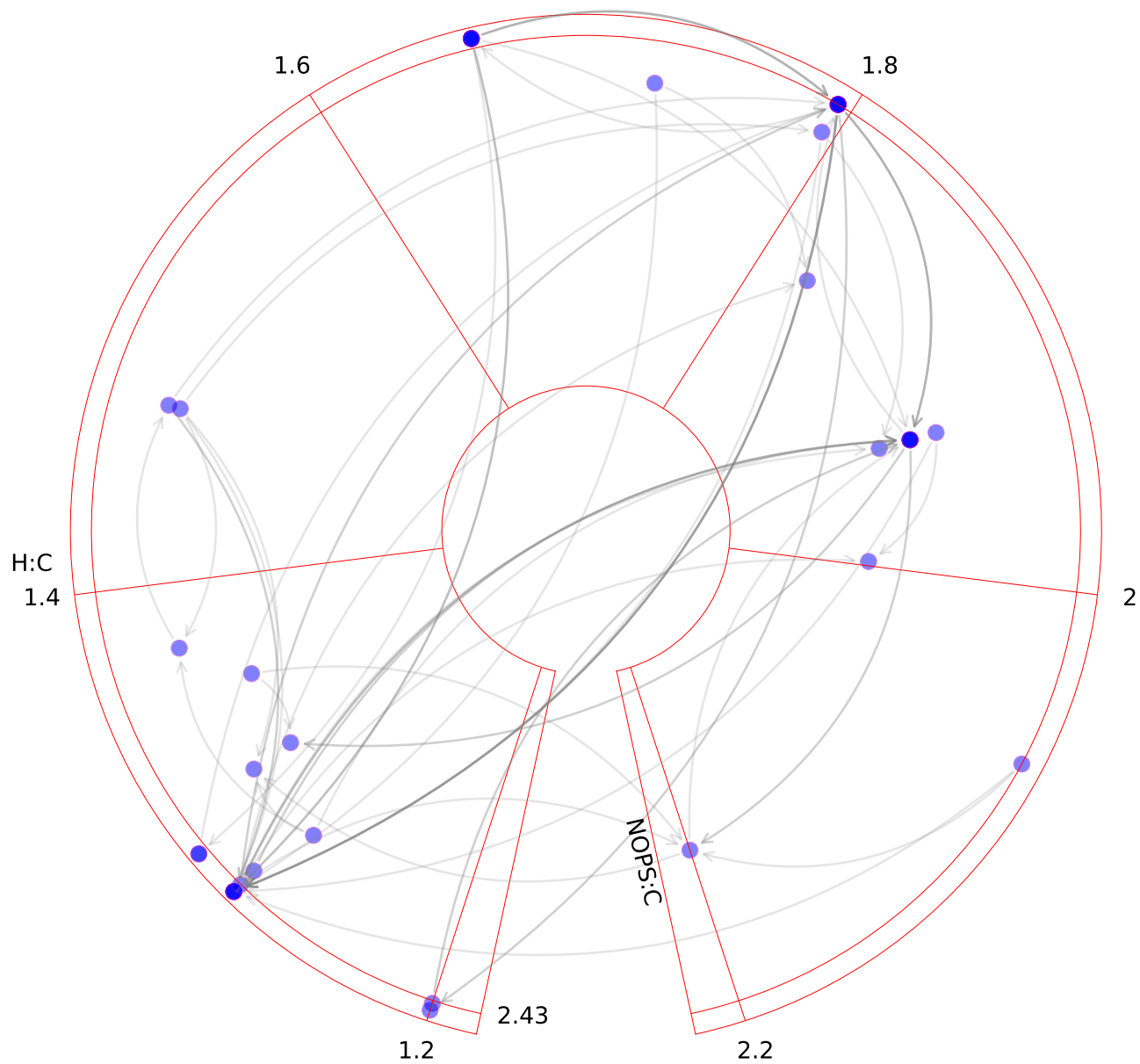

Supplement: Supplement 1 [file media-1.zip › Suppl_File_all_pathways/nolabel/Fructose and mannose metabolism.pdf]

# Vitamin D metabolism

1.4

H:C

0.161

NOPS:C

1.2

1.6

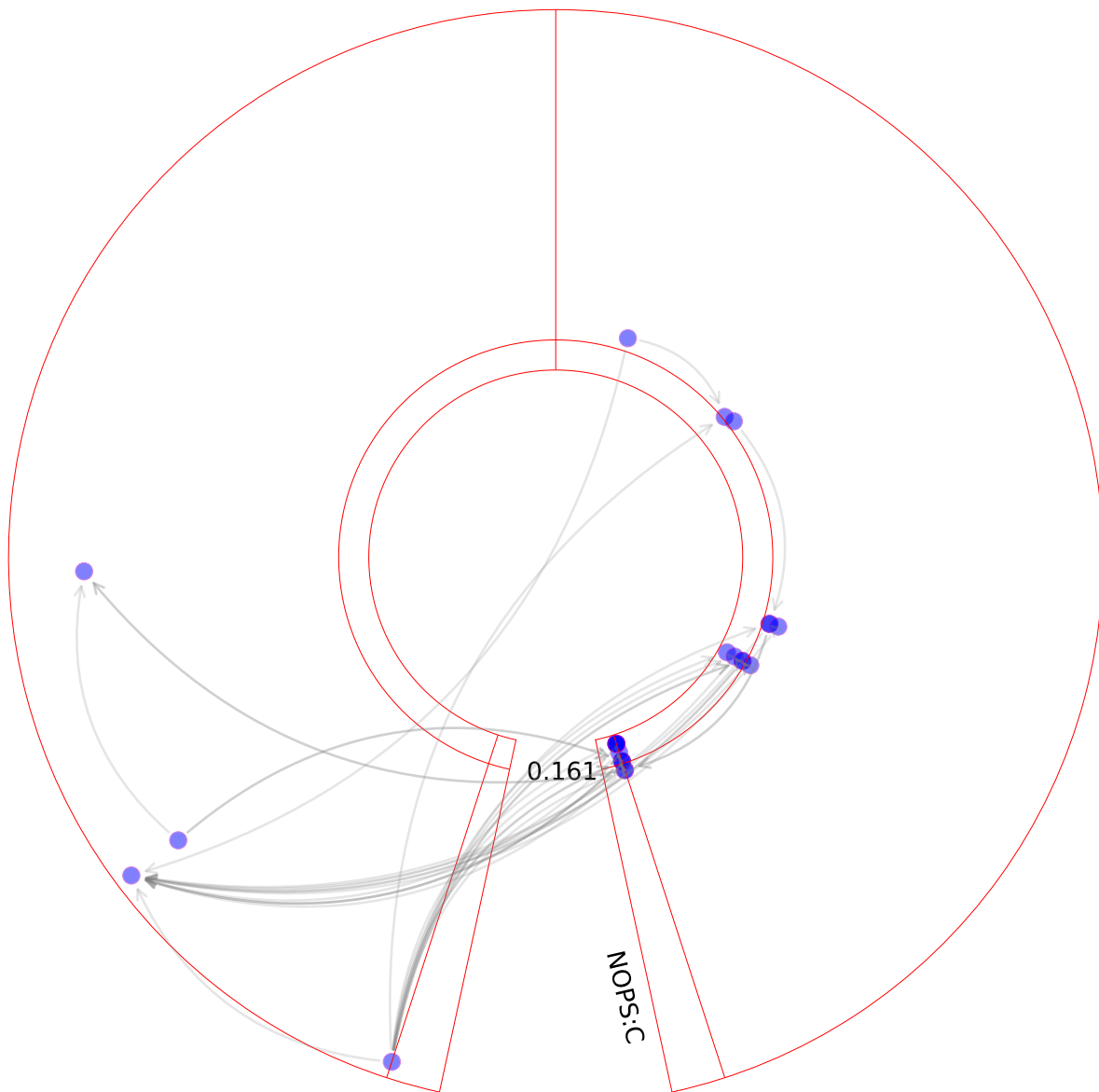

Supplement: Supplement 1 [file media-1.zip › Suppl_File_all_pathways/nolabel/Vitamin D metabolism.pdf]

# Eicosanoid metabolism

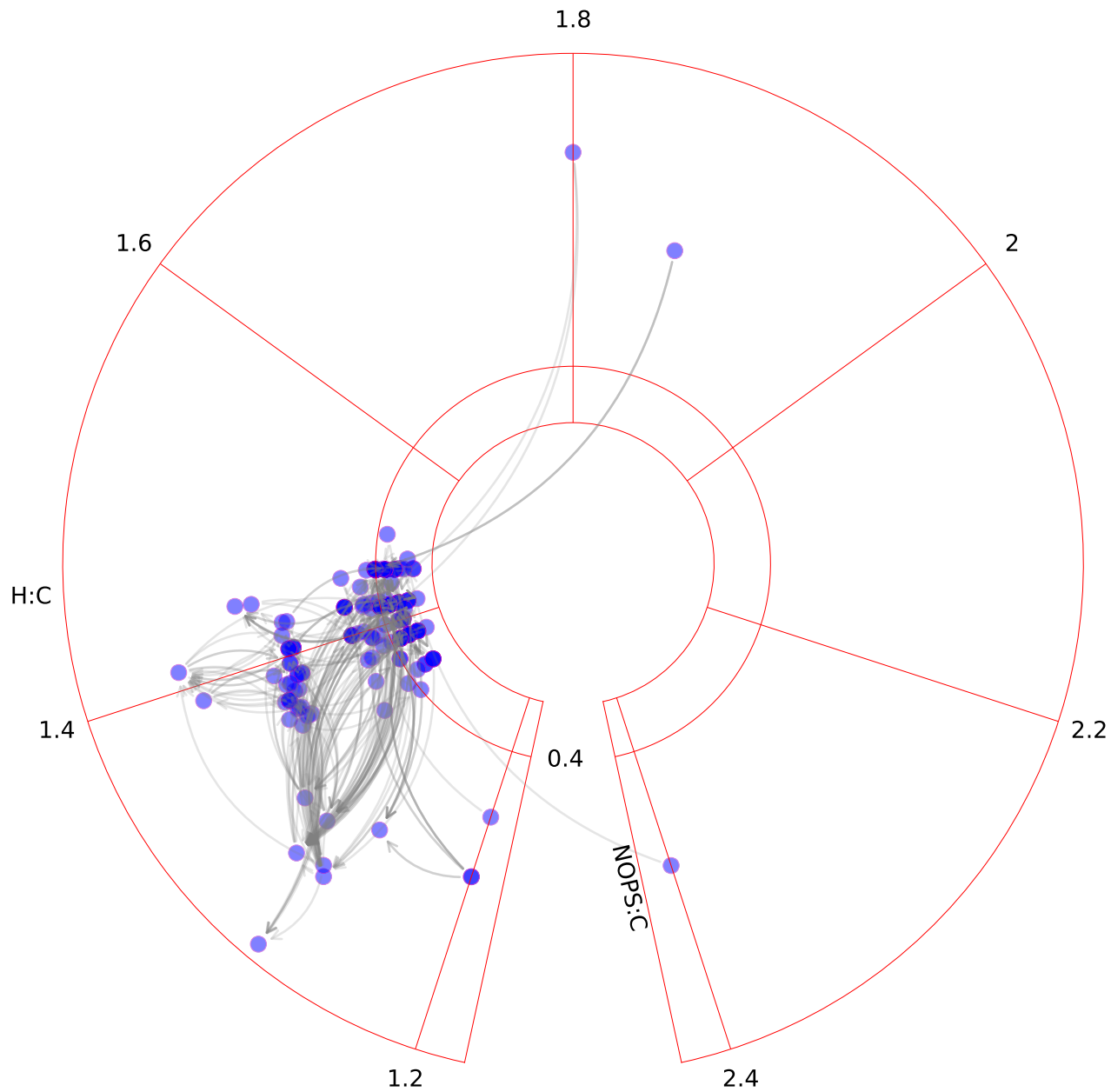

Supplement: Supplement 1 [file media-1.zip › Suppl_File_all_pathways/nolabel/Eicosanoid metabolism.pdf]

# Fatty acid synthesis

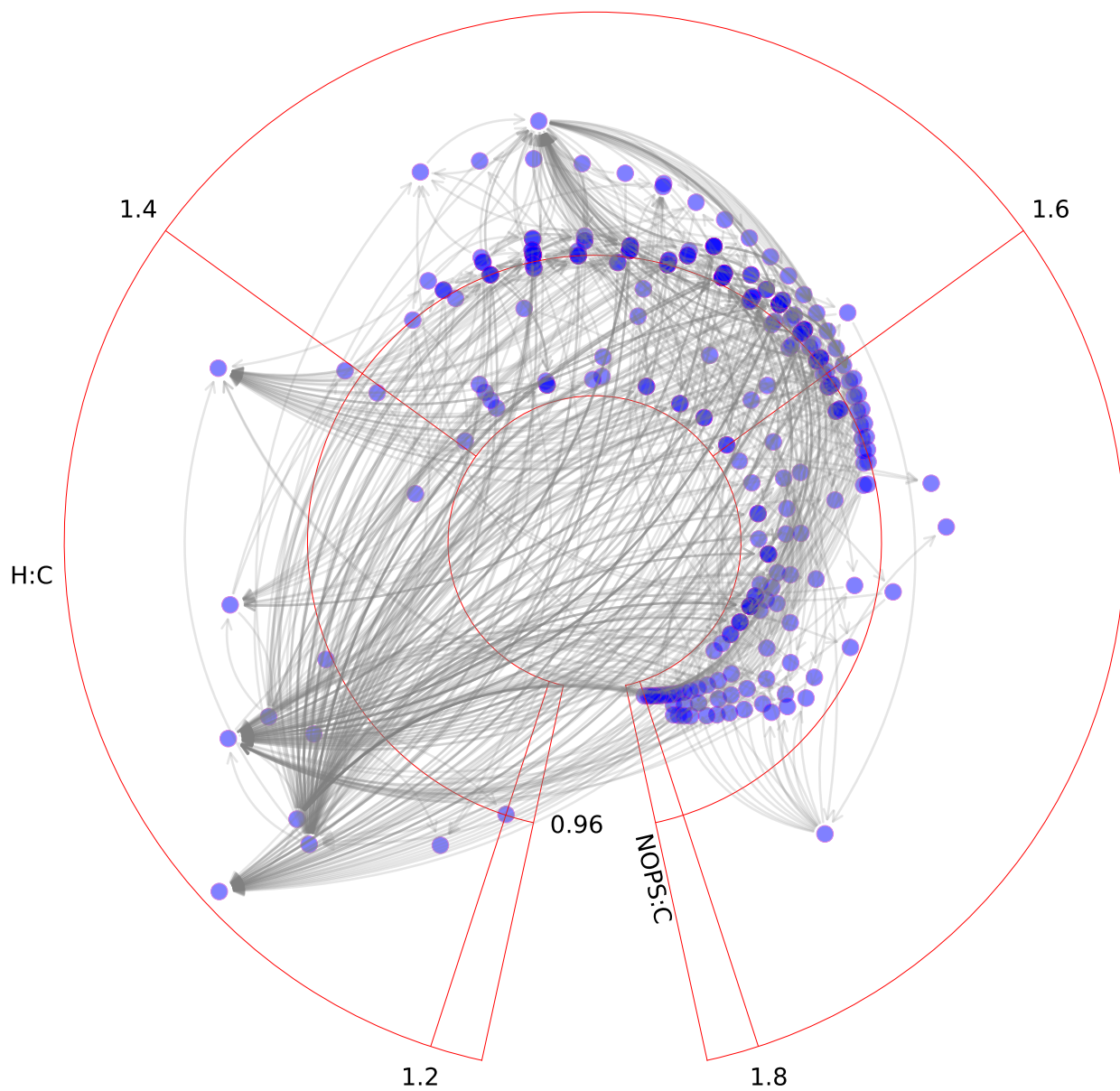

Supplement: Supplement 1 [file media-1.zip › Suppl_File_all_pathways/nolabel/Fatty acid synthesis.pdf]

# Vitamin K metabolism

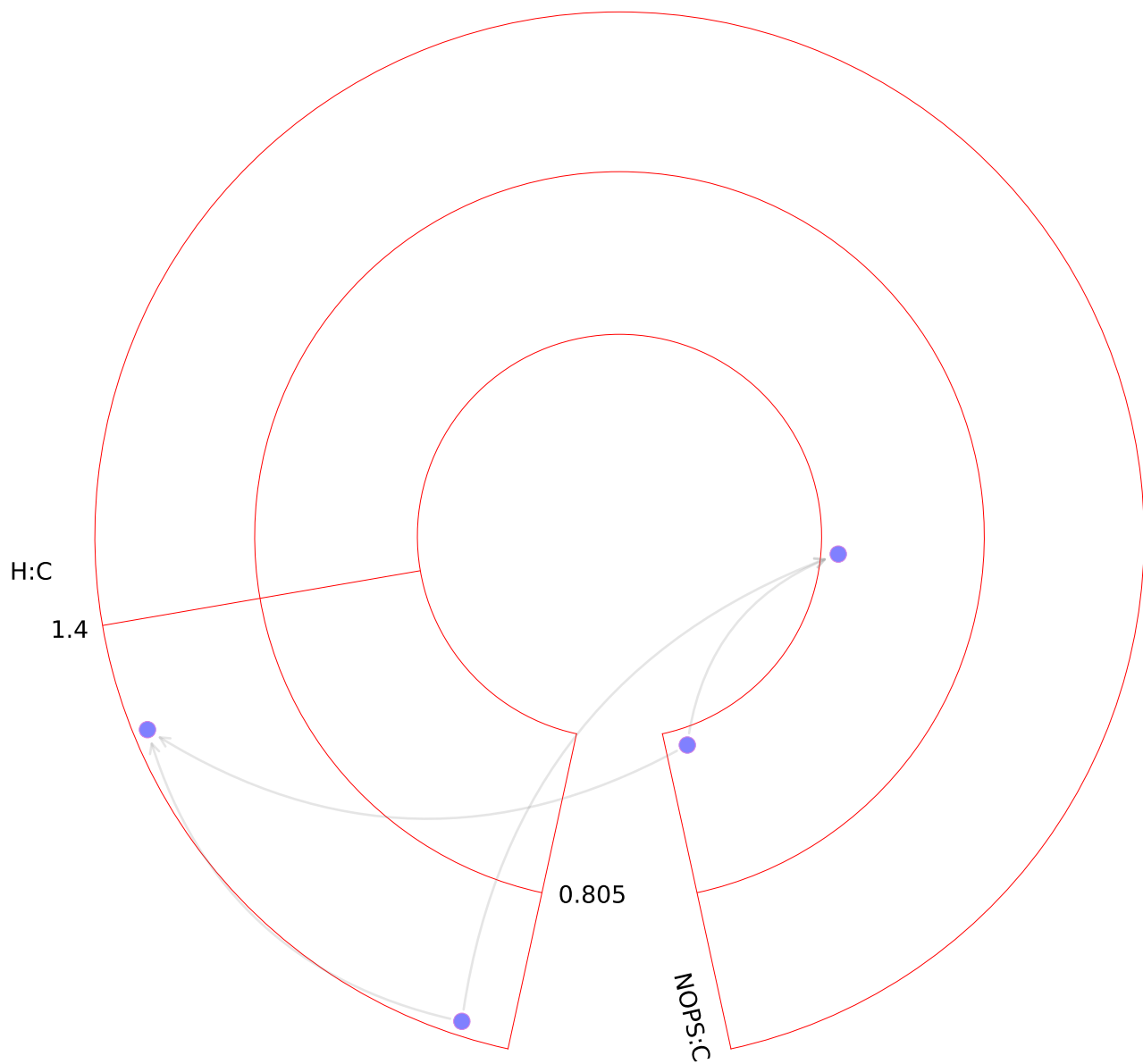

Supplement: Supplement 1 [file media-1.zip › Suppl_File_all_pathways/nolabel/Vitamin K metabolism.pdf]

# R group synthesis

1.4

H:C

1.058

NOPS:C

1.2

1.6

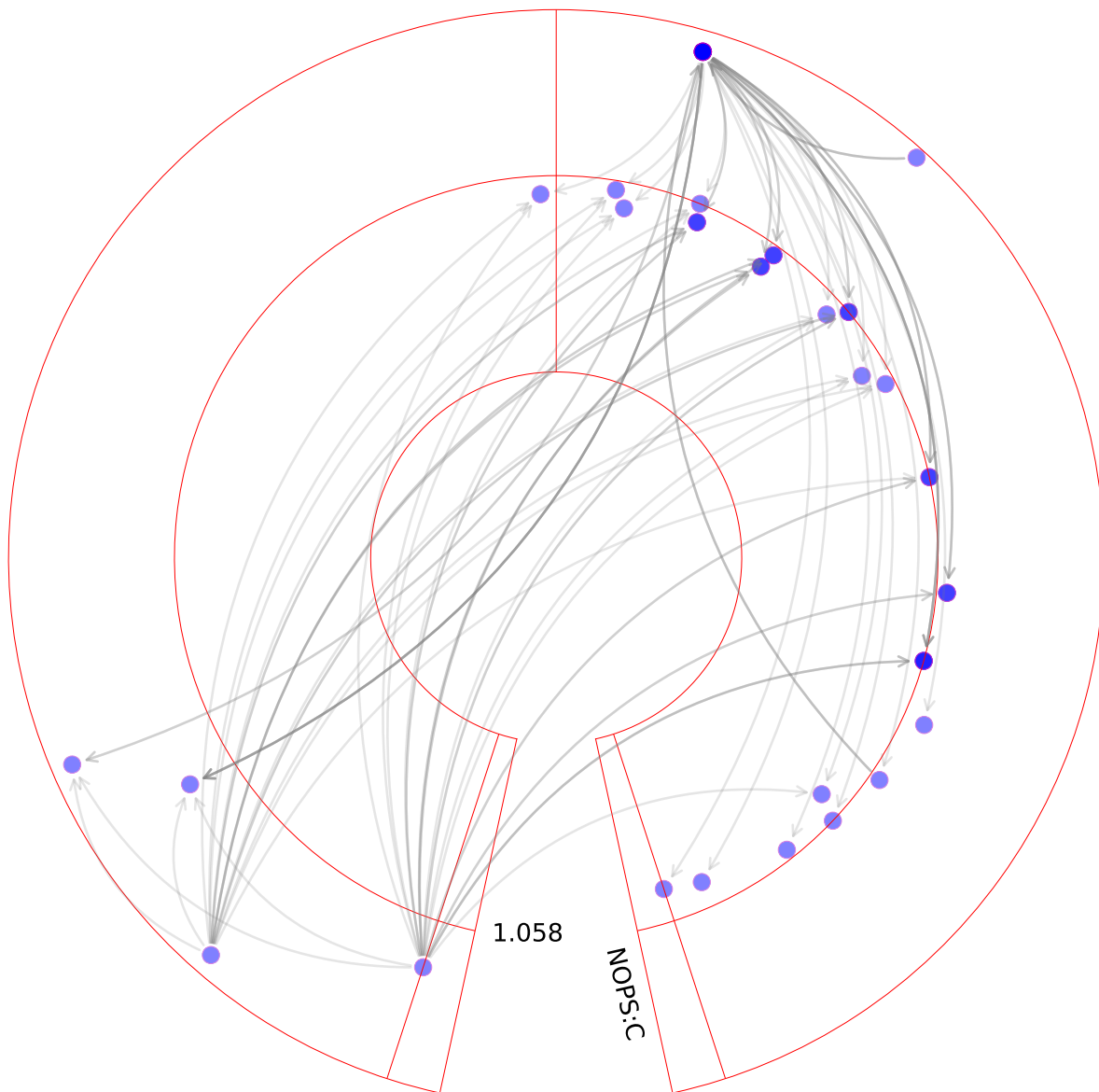

Supplement: Supplement 1 [file media-1.zip › Suppl_File_all_pathways/nolabel/R group synthesis.pdf]

# Tetrahydrobiopterin metabolism

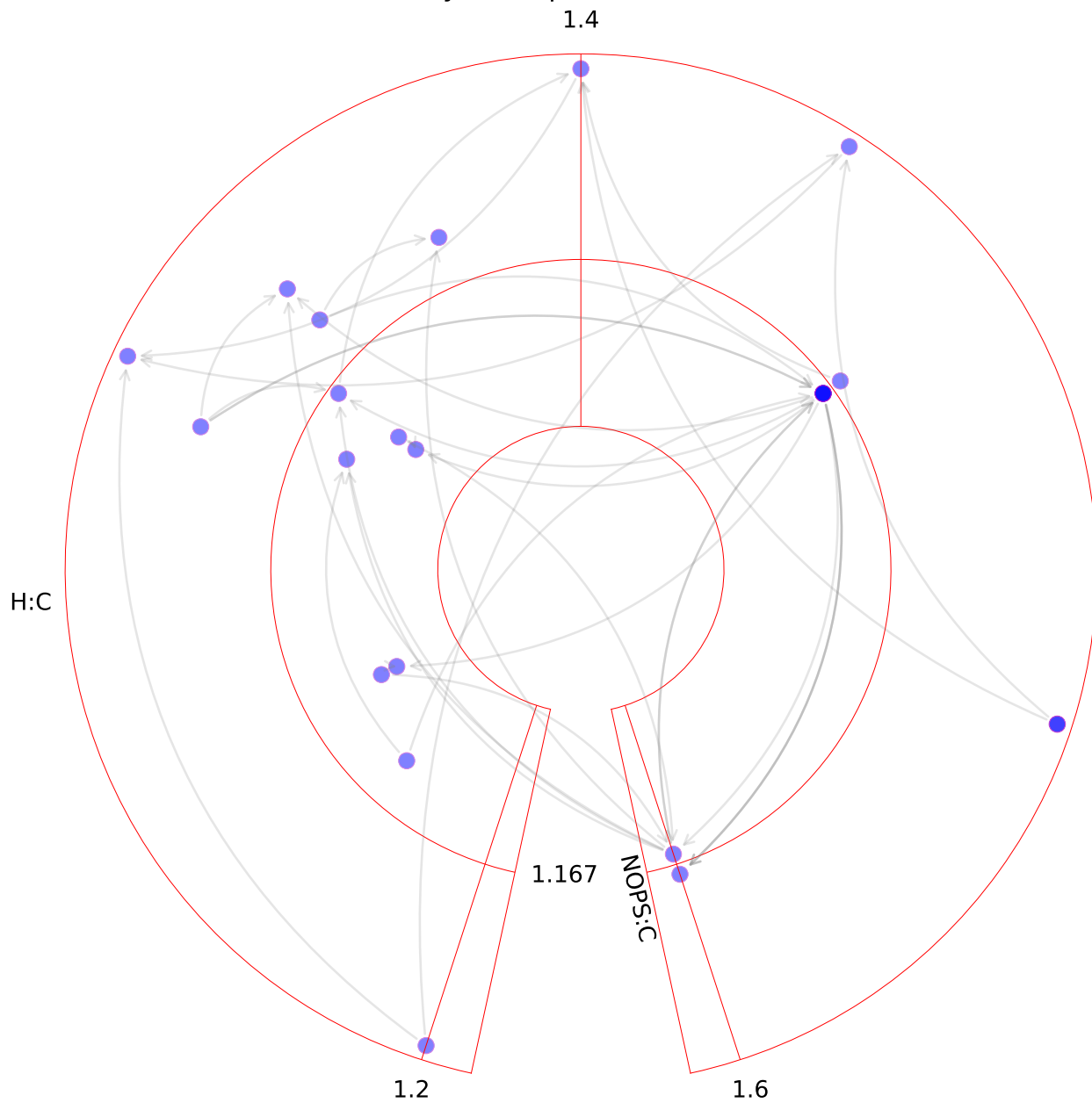

Supplement: Supplement 1 [file media-1.zip › Suppl_File_all_pathways/nolabel/Tetrahydrobiopterin metabolism.pdf]

# Lysine metabolism

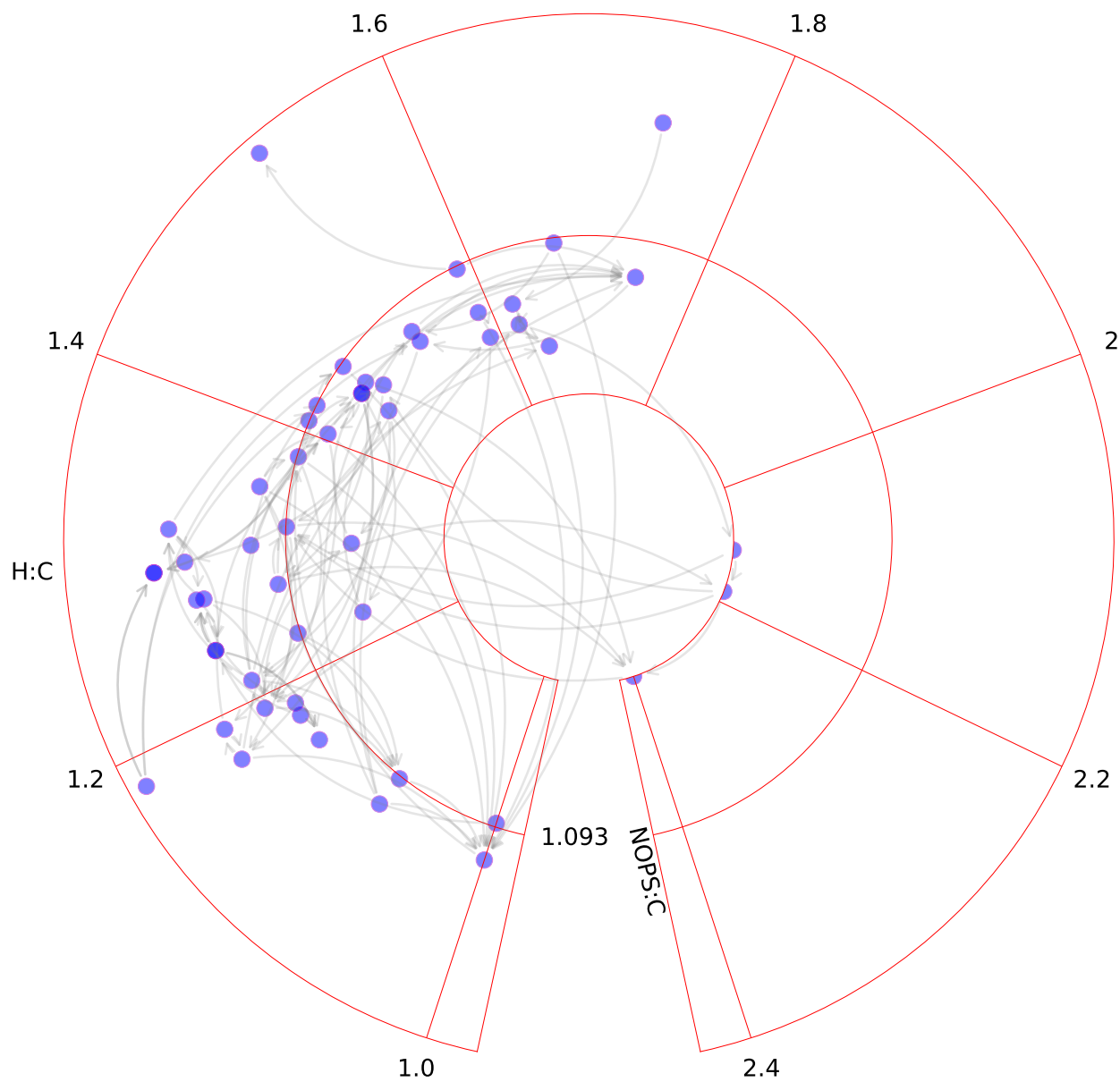

Supplement: Supplement 1 [file media-1.zip › Suppl_File_all_pathways/nolabel/Lysine metabolism.pdf]

# Blood group synthesis

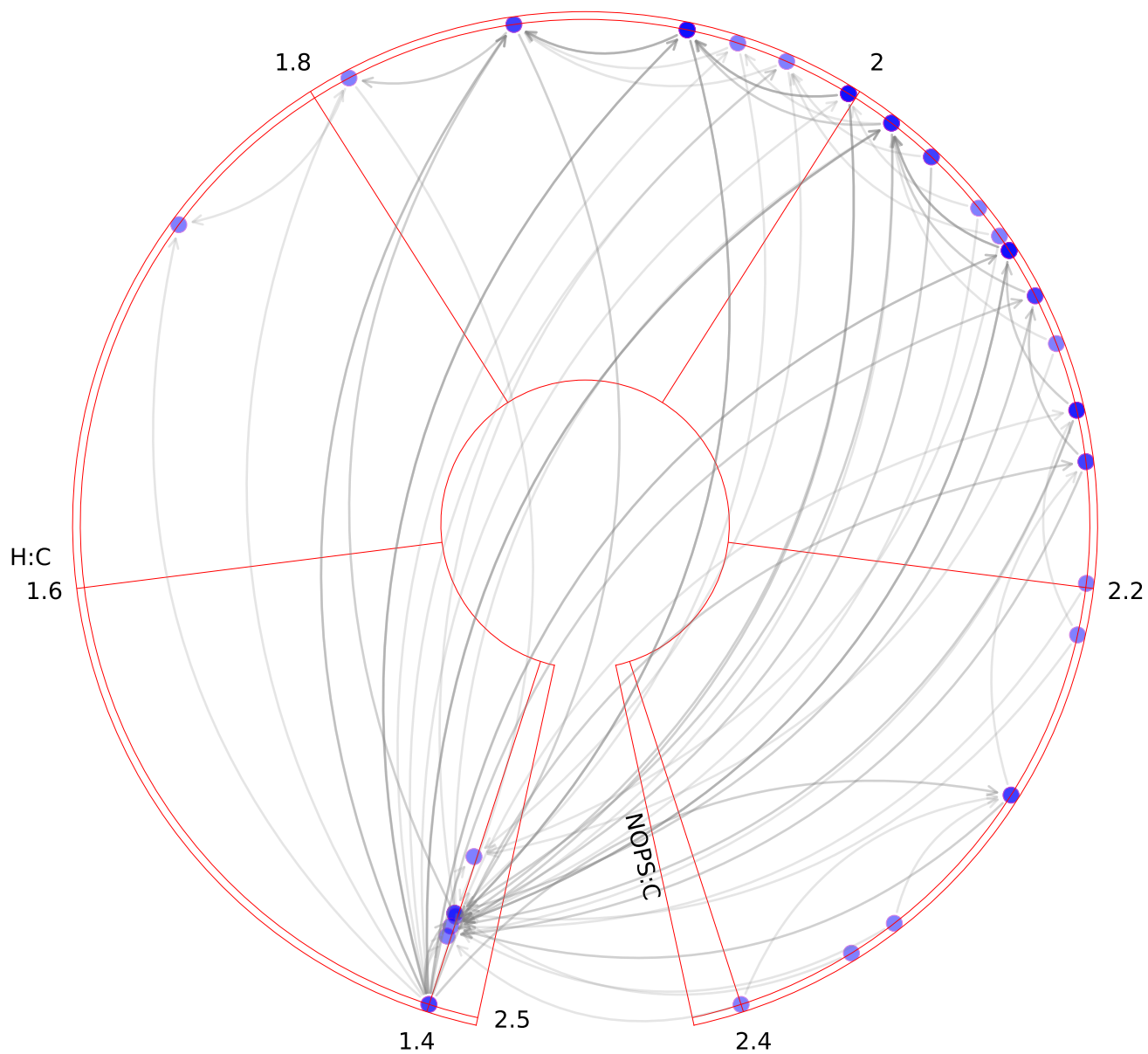

Supplement: Supplement 1 [file media-1.zip › Suppl_File_all_pathways/nolabel/Blood group synthesis.pdf]

# Alkaloid synthesis

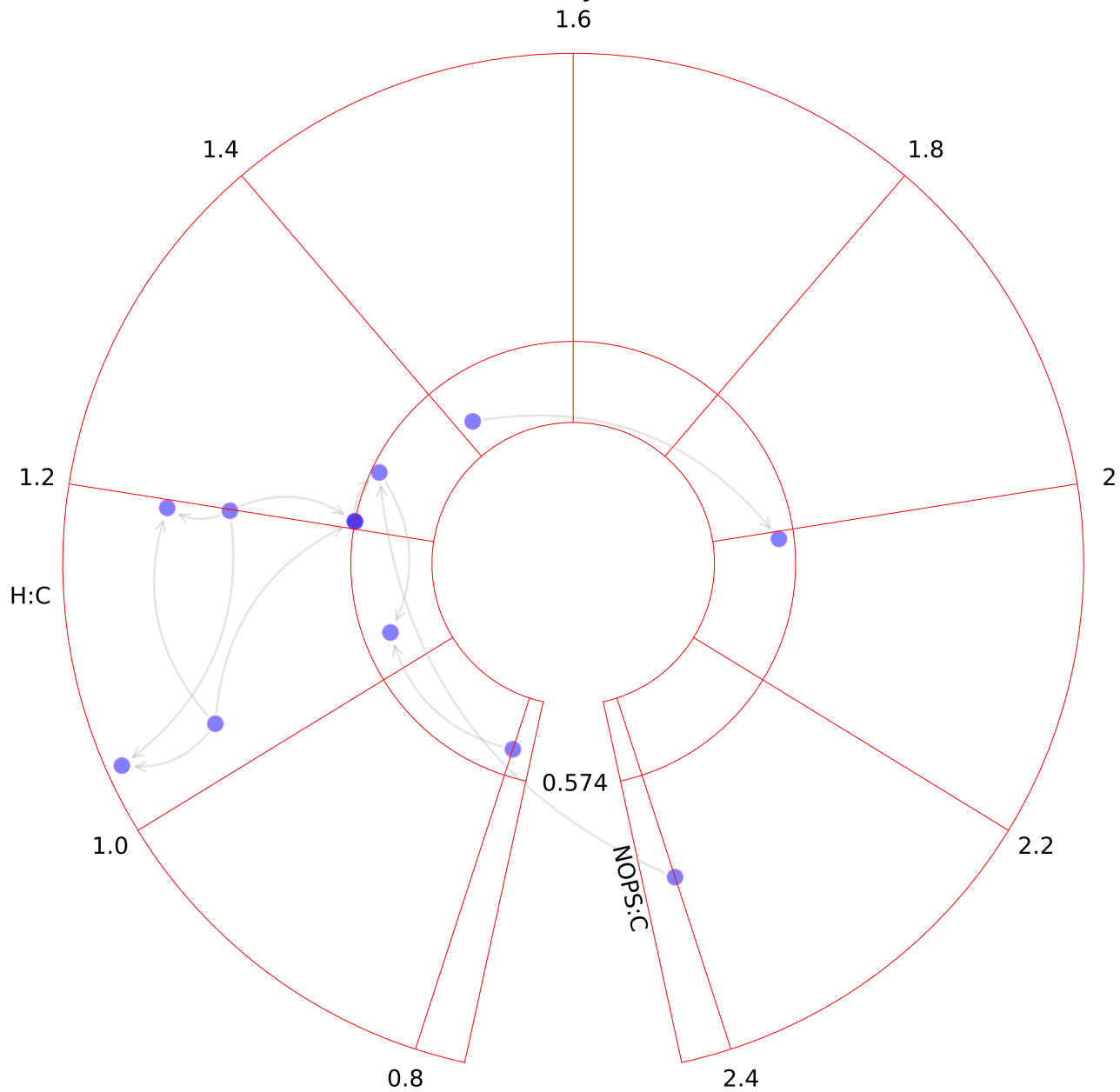

Supplement: Supplement 1 [file media-1.zip › Suppl_File_all_pathways/nolabel/Alkaloid synthesis.pdf]

# N-glycan degradation

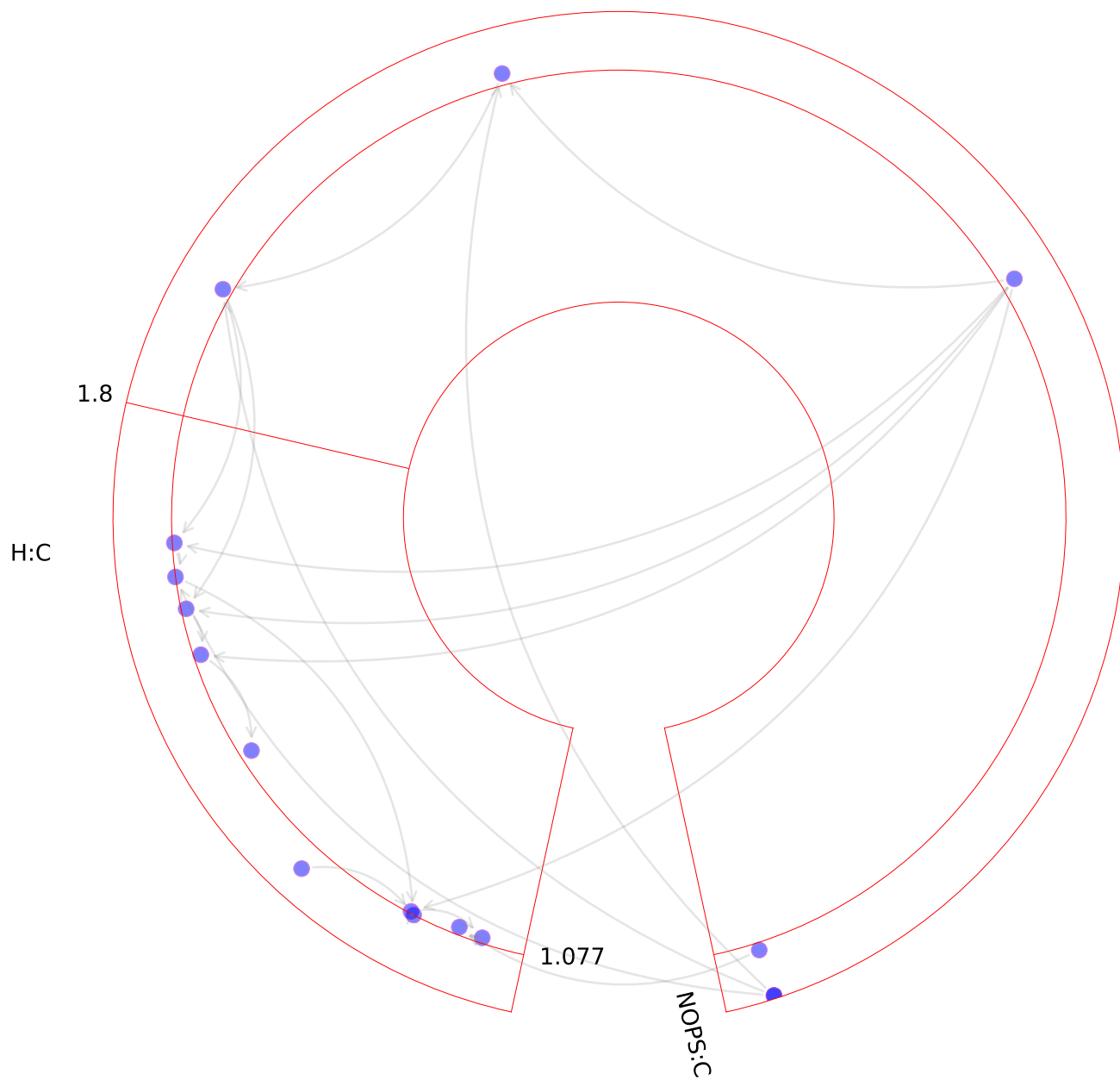

Supplement: Supplement 1 [file media-1.zip › Suppl_File_all_pathways/nolabel/N-glycan degradation.pdf]

# Squalene and cholesterol synthesis

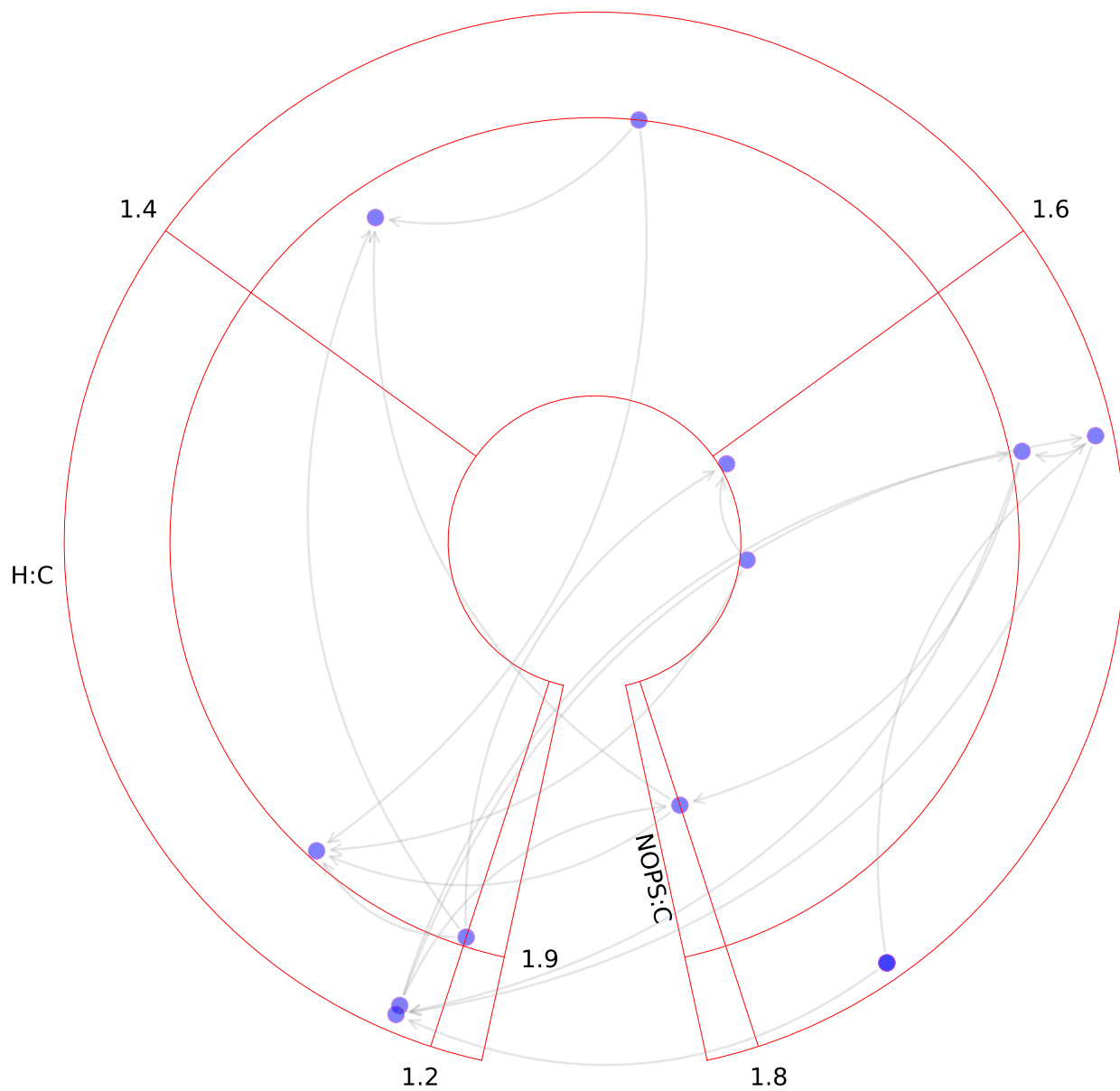

Supplement: Supplement 1 [file media-1.zip › Suppl_File_all_pathways/nolabel/Squalene and cholesterol synthesis.pdf]

# O-glycan metabolism

1.8

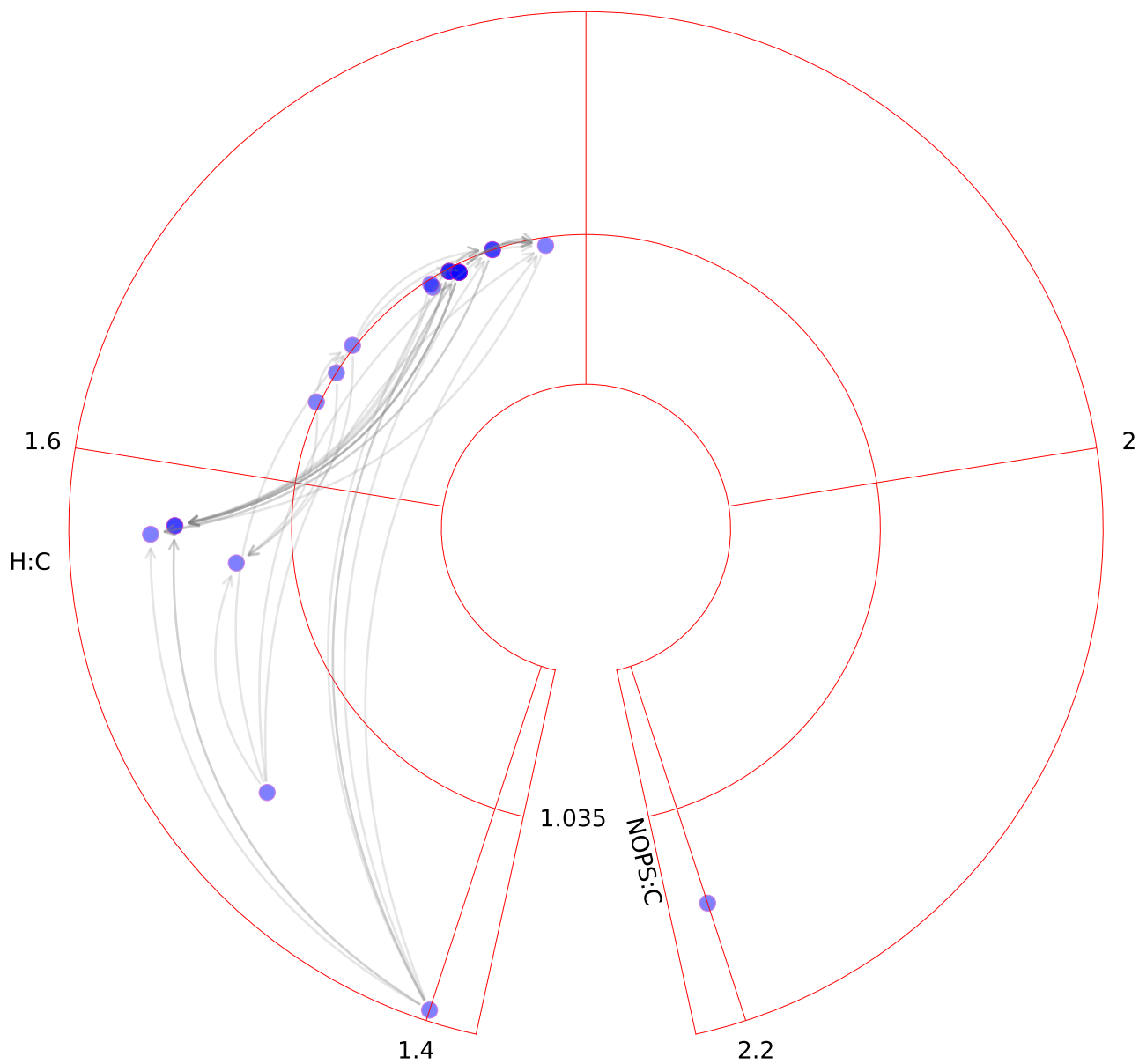

Supplement: Supplement 1 [file media-1.zip › Suppl_File_all_pathways/nolabel/O-glycan metabolism.pdf]

# Vitamin C metabolism

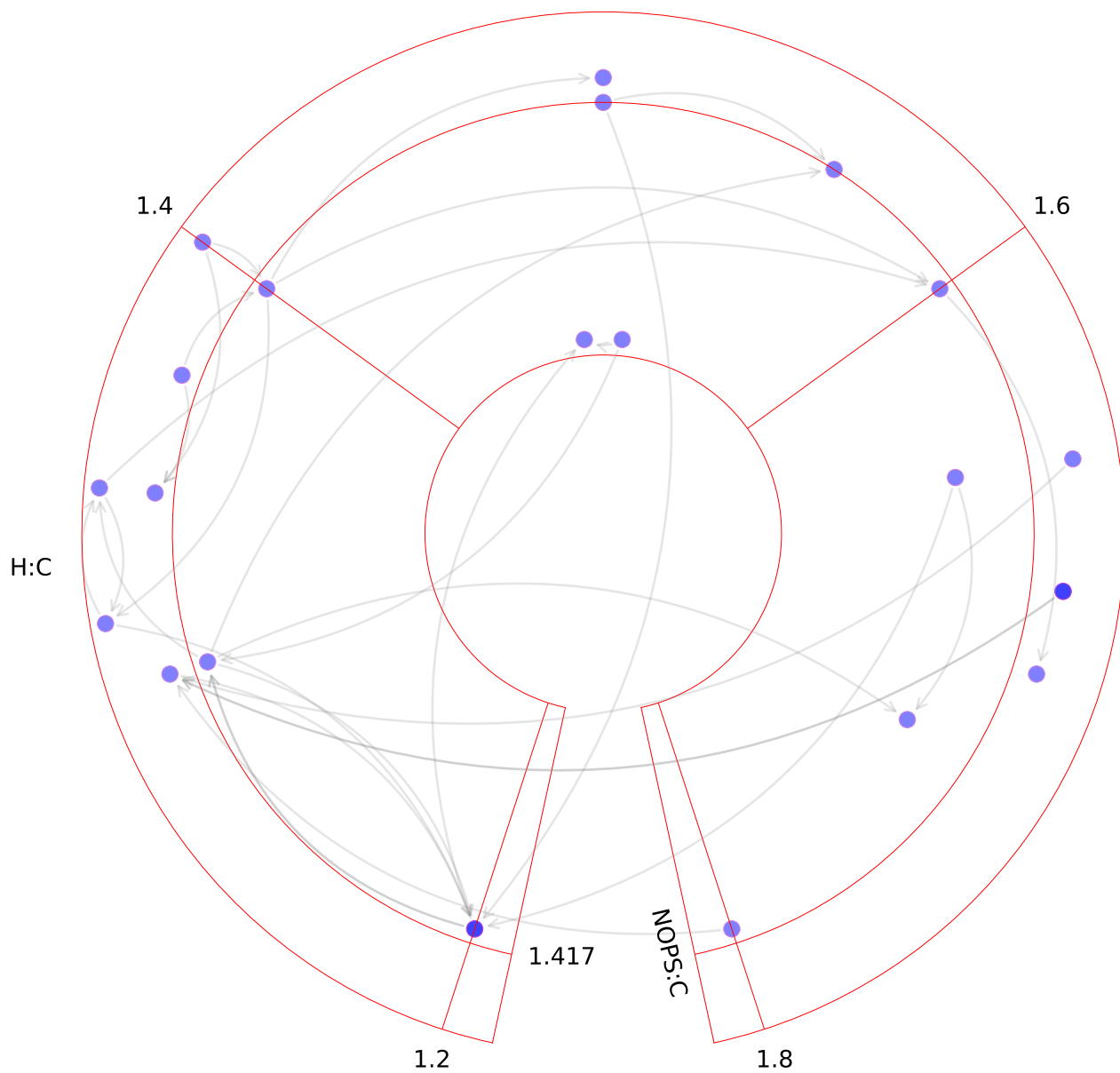

Supplement: Supplement 1 [file media-1.zip › Suppl_File_all_pathways/nolabel/Vitamin C metabolism.pdf]

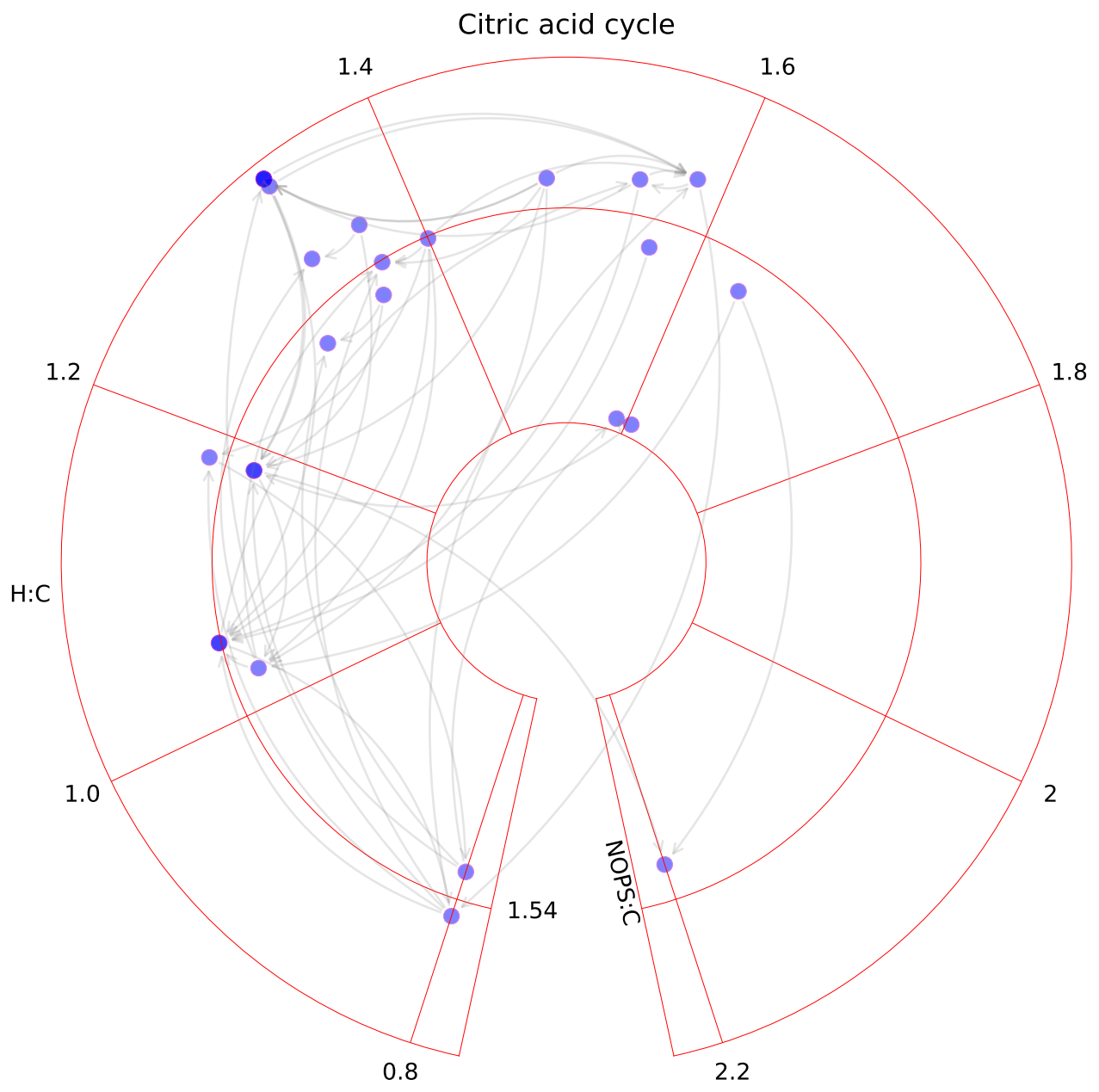

Supplement: Supplement 1 [file media-1.zip › Suppl_File_all_pathways/nolabel/Citric acid cycle.pdf]

# Taurine and hypotaurine metabolism

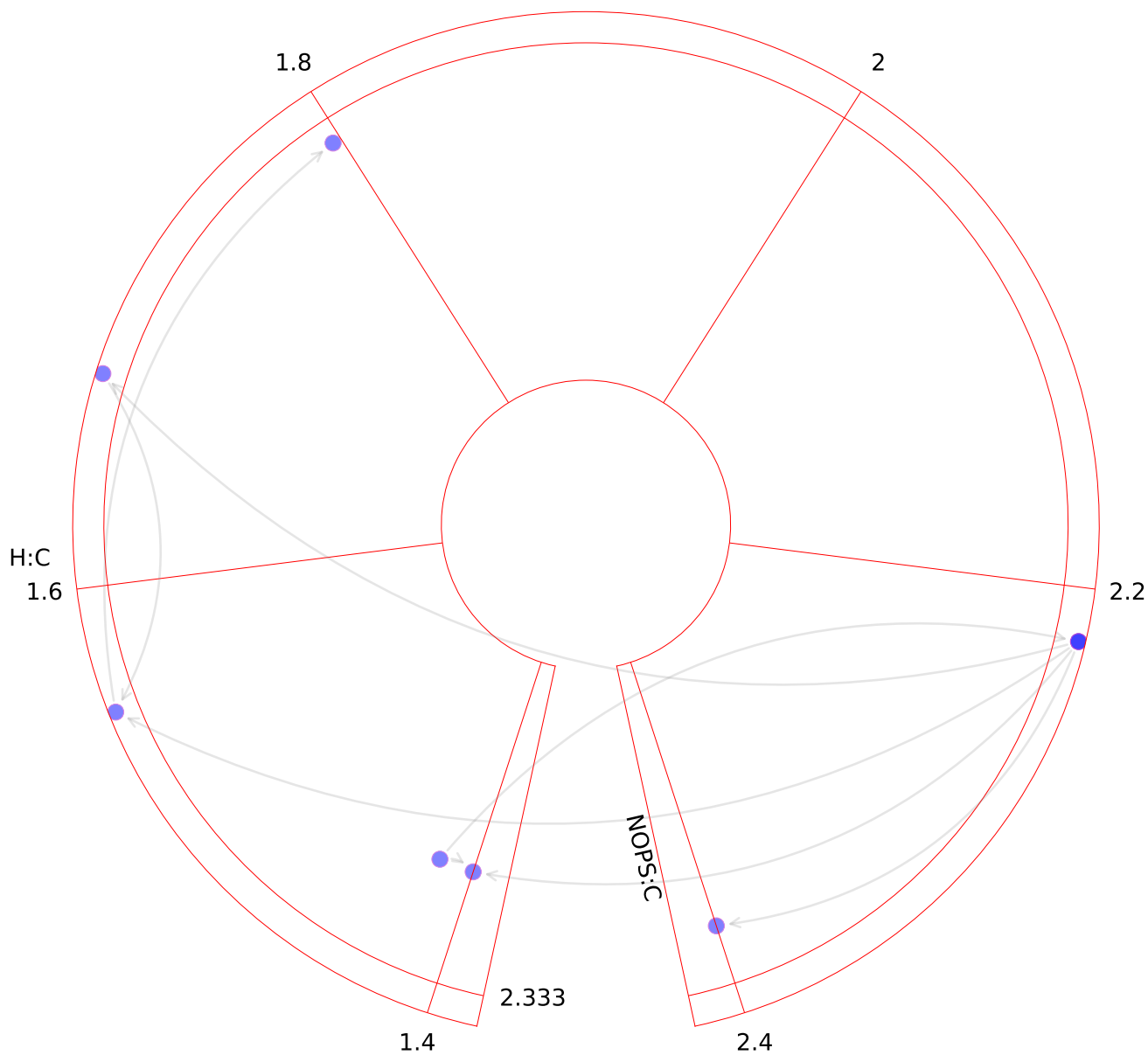

Supplement: Supplement 1 [file media-1.zip › Suppl_File_all_pathways/nolabel/Taurine and hypotaurine metabolism.pdf]

# Purine synthesis

1.6

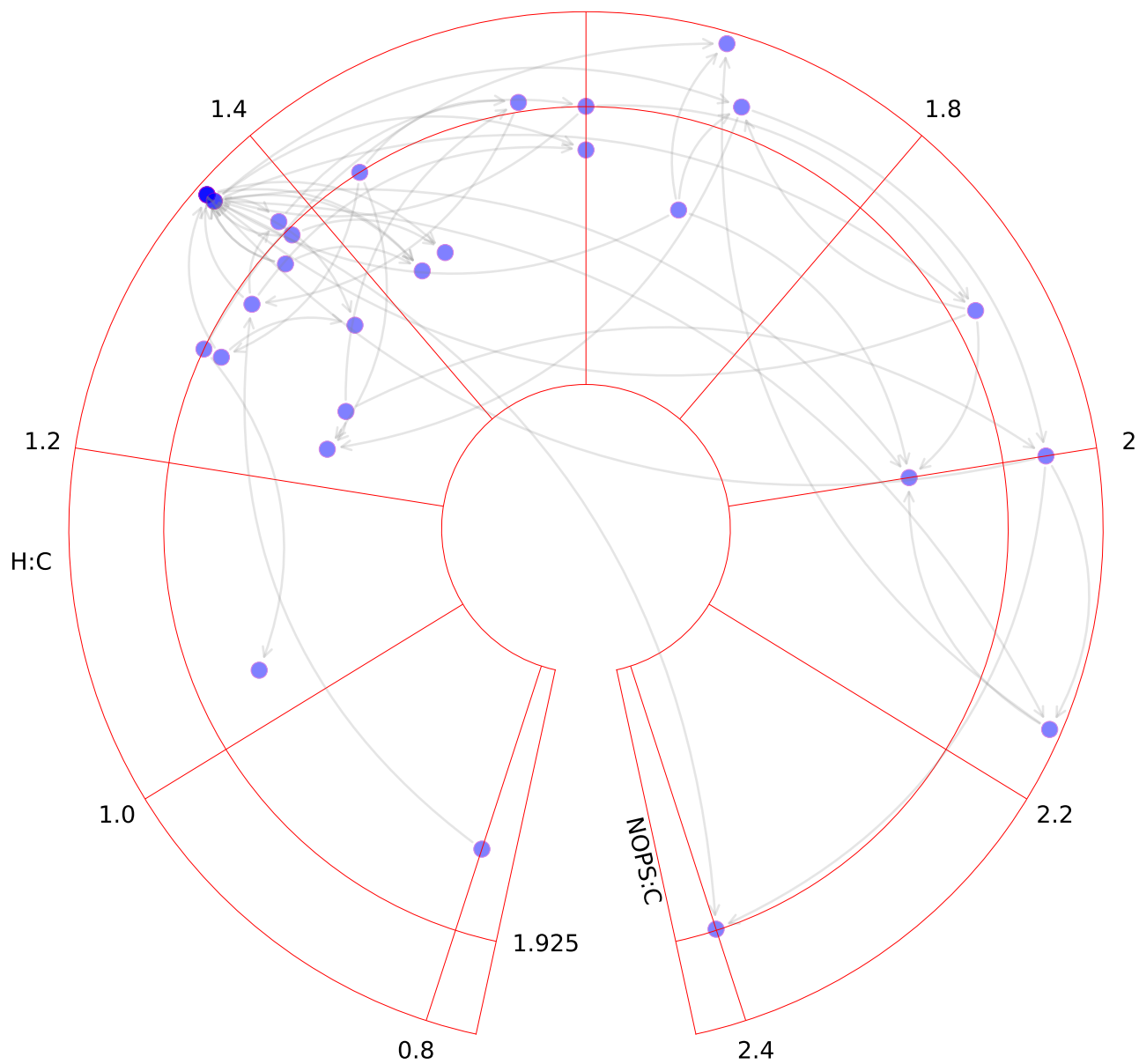

Supplement: Supplement 1 [file media-1.zip › Suppl_File_all_pathways/nolabel/Purine synthesis.pdf]

# Hyaluronan metabolism

1.6

H:C

1.35

NOPS:C

1.4

1.8

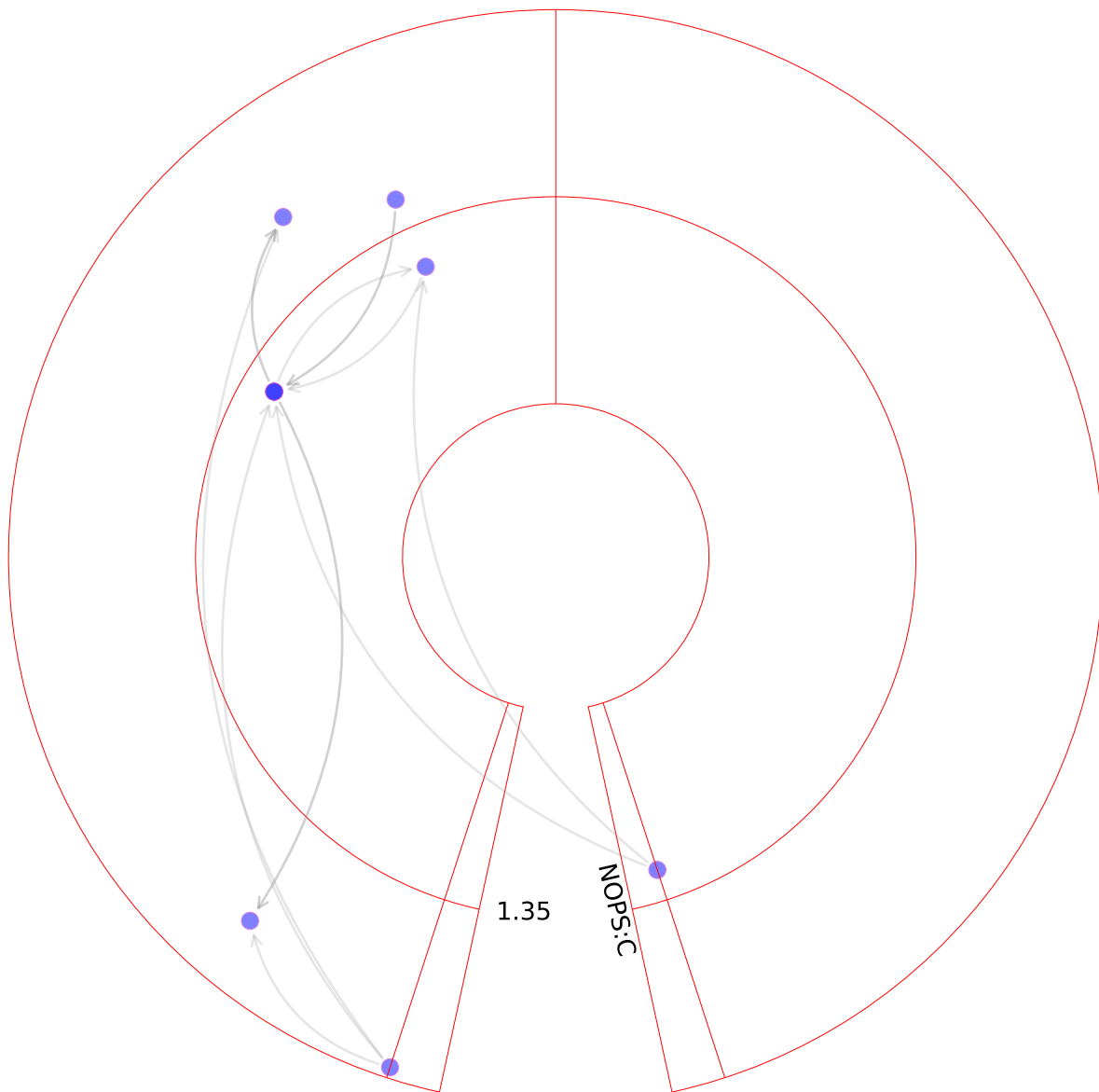

Supplement: Supplement 1 [file media-1.zip › Suppl_File_all_pathways/nolabel/Hyaluronan metabolism.pdf]

# Vitamin B6 metabolism

1.4

H:C

NOPS:C

1.448

1.2

1.6

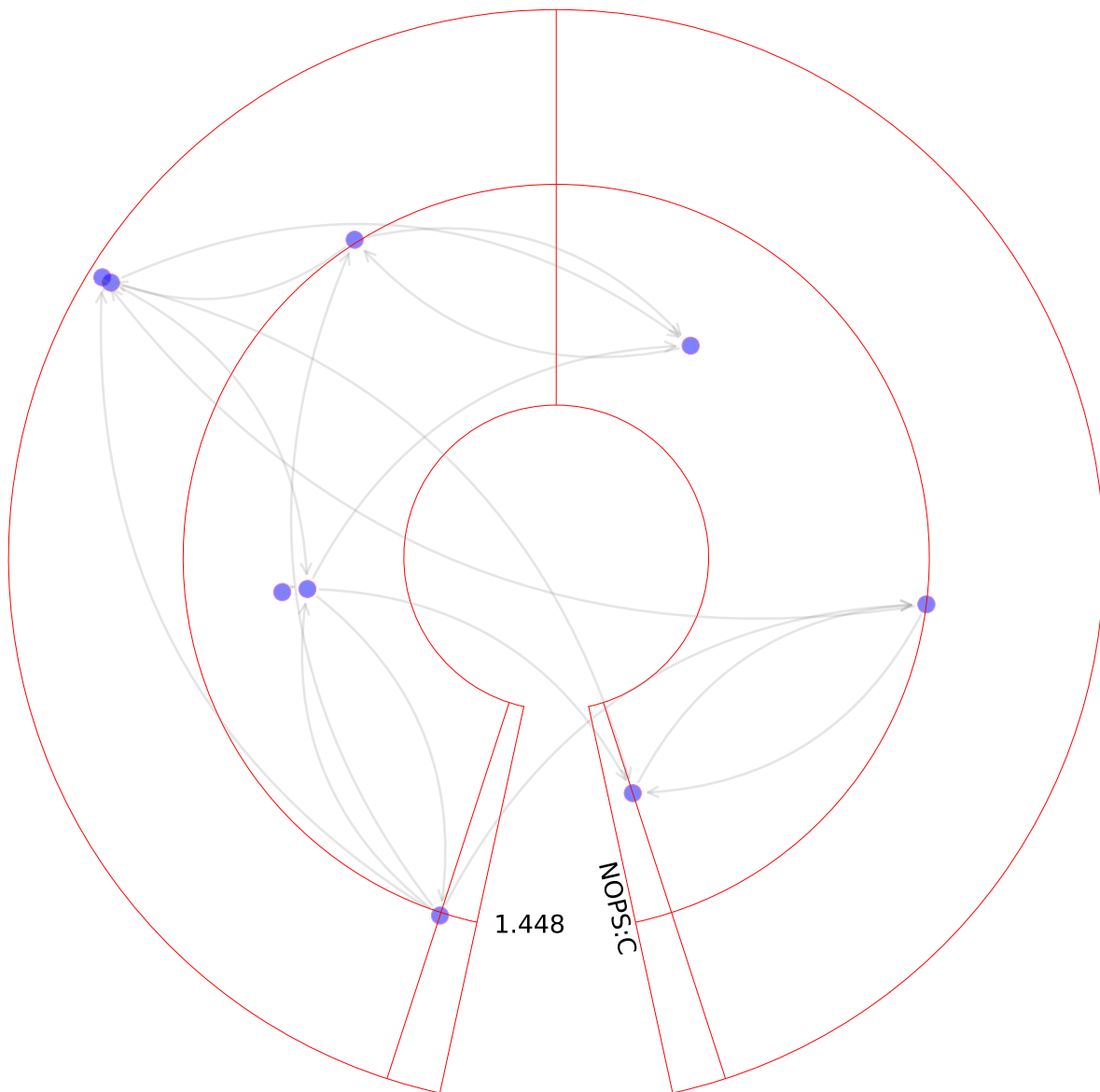

Supplement: Supplement 1 [file media-1.zip › Suppl_File_all_pathways/nolabel/Vitamin B6 metabolism.pdf]

# Pyrimidine synthesis

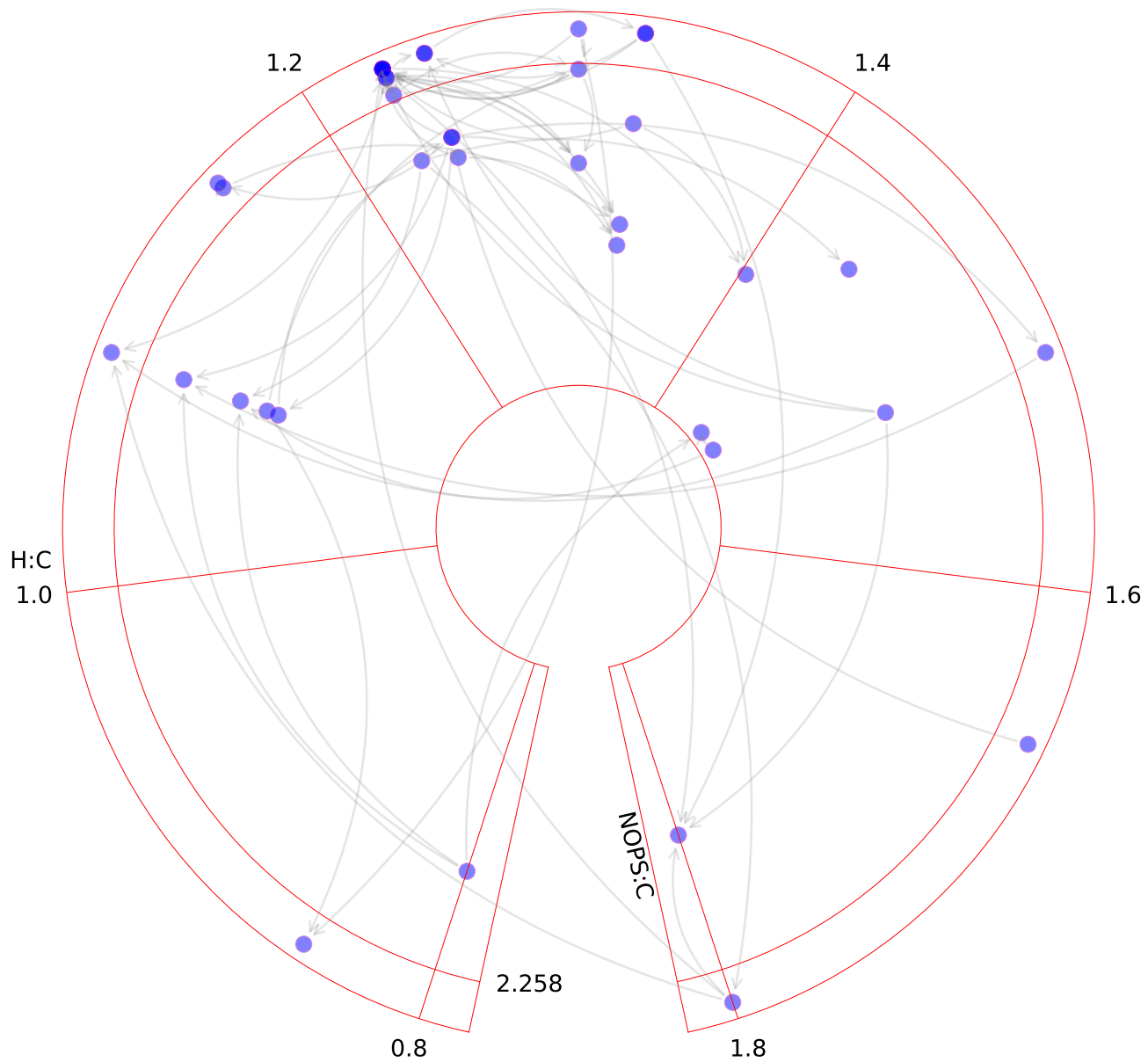

Supplement: Supplement 1 [file media-1.zip › Suppl_File_all_pathways/nolabel/Pyrimidine synthesis.pdf]

# Vitamin E metabolism

1.4

H:C

0.213

NO<sub>2</sub>:C

1.2

1.6

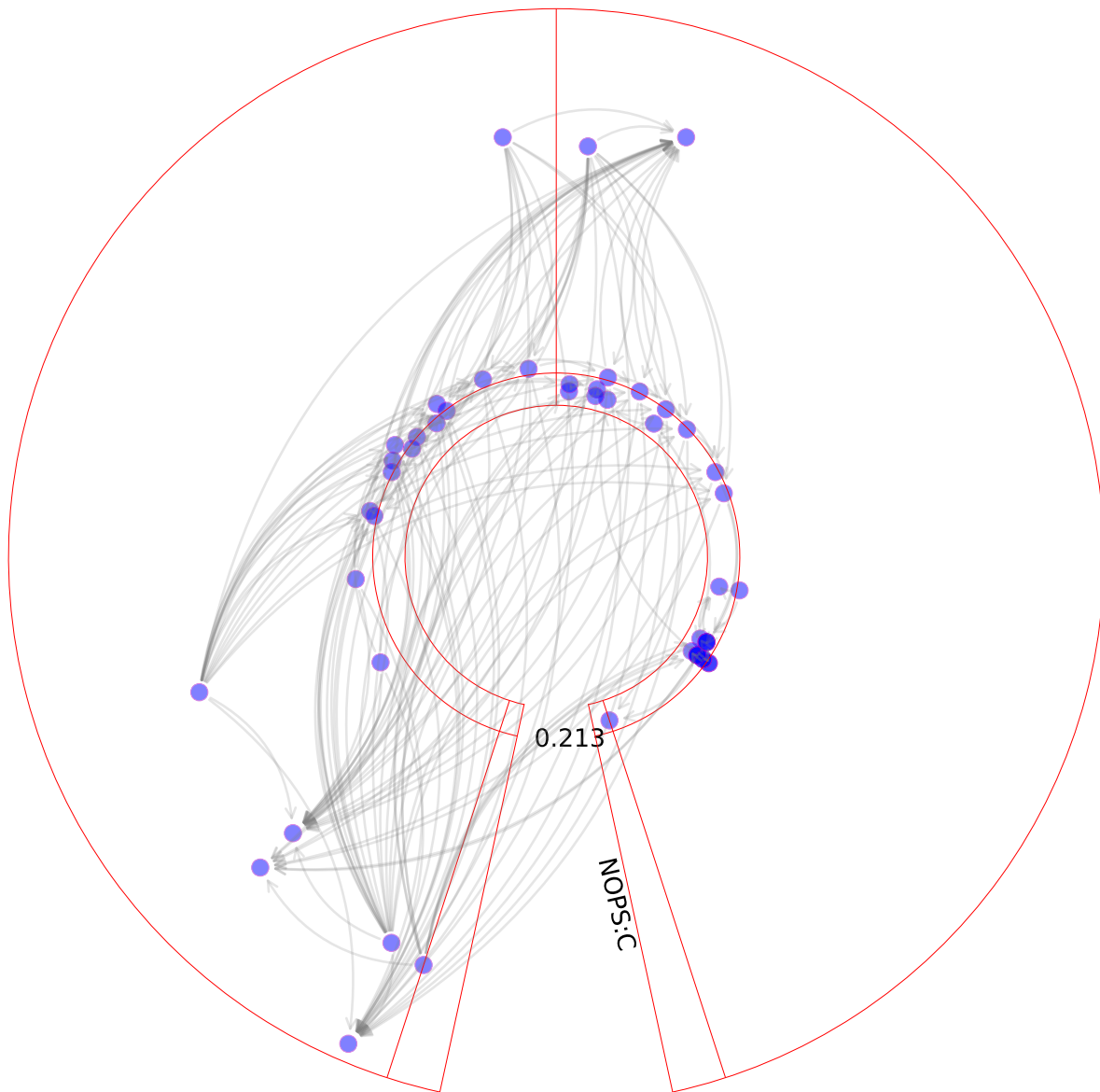

Supplement: Supplement 1 [file media-1.zip › Suppl_File_all_pathways/nolabel/Vitamin E metabolism.pdf]

# Aminosugar metabolism

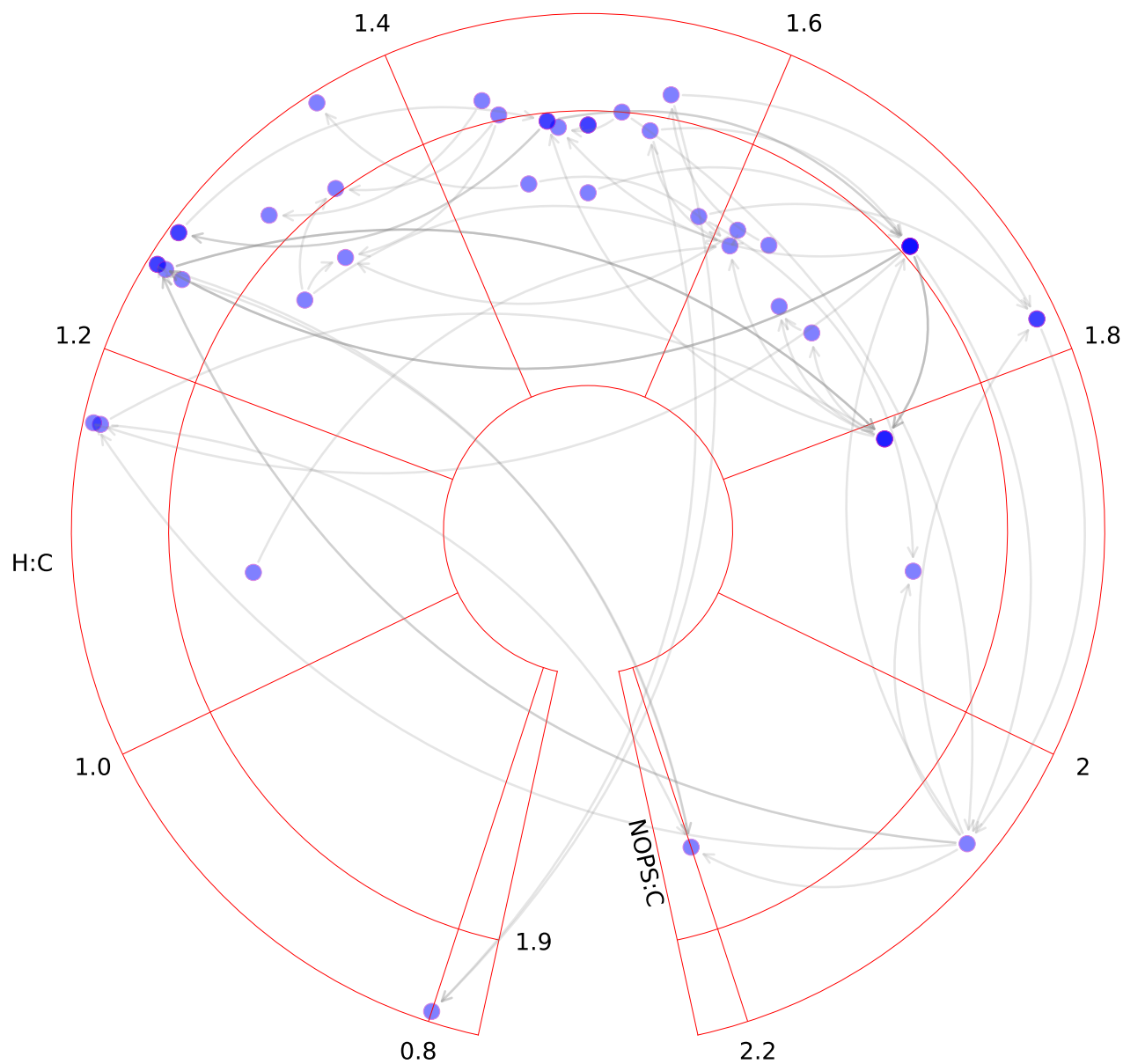

Supplement: Supplement 1 [file media-1.zip › Suppl_File_all_pathways/nolabel/Aminosugar metabolism.pdf]

ROS detoxification

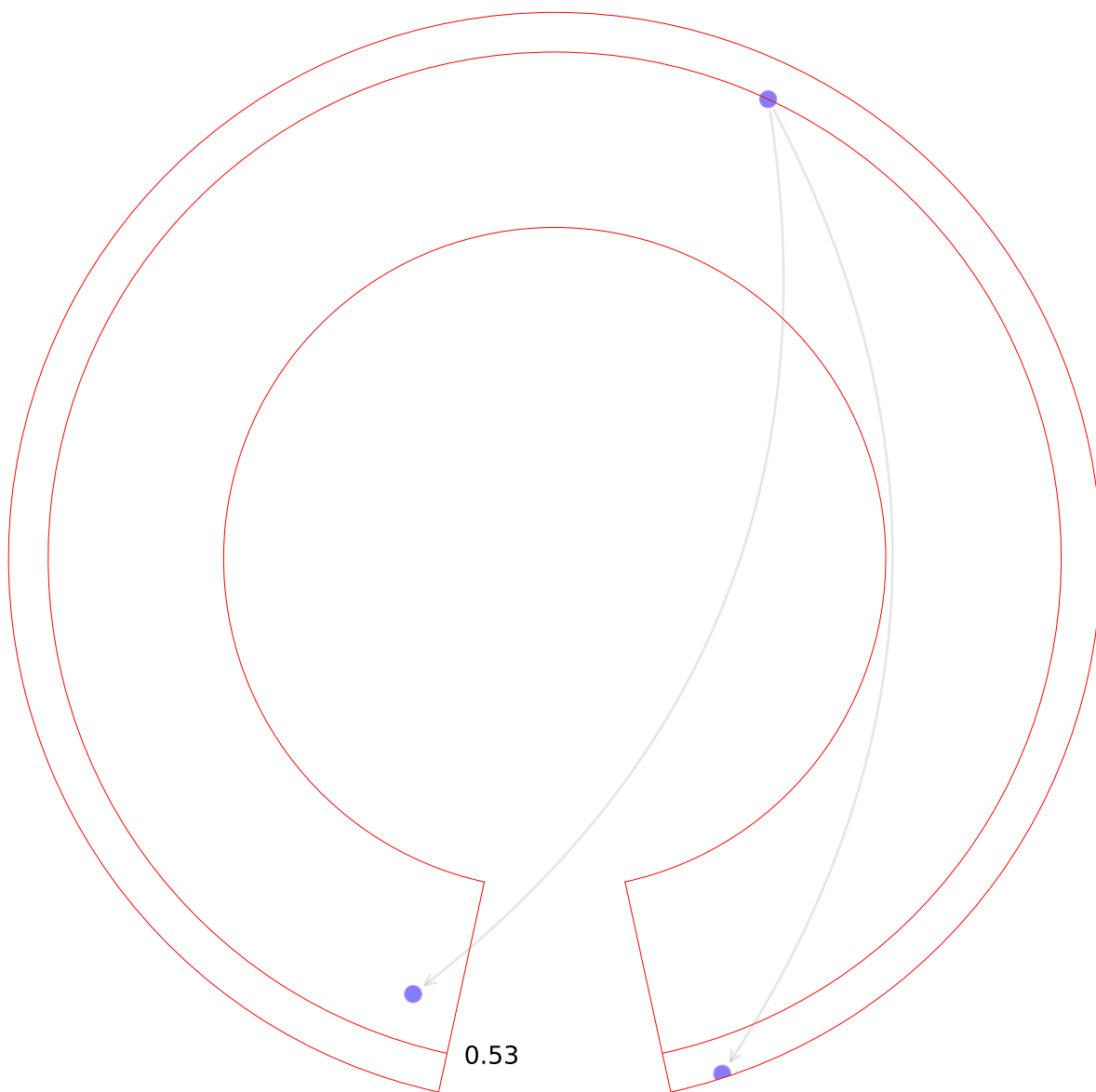

0.53

H:C

Supplement: Supplement 1 [file media-1.zip › Suppl_File_all_pathways/nolabel/ROS detoxification.pdf]

# Heparan sulfate degradation

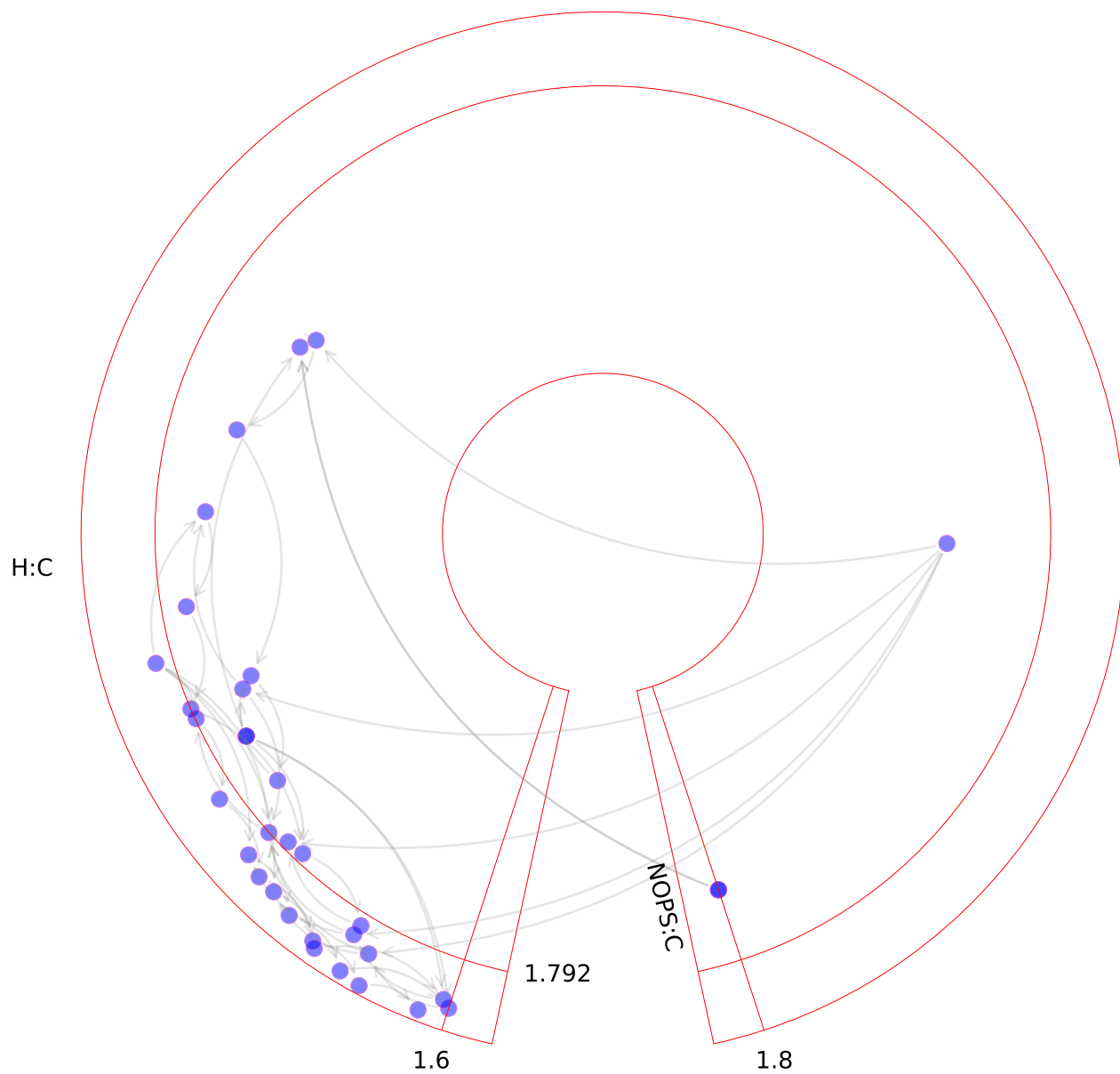

Supplement: Supplement 1 [file media-1.zip › Suppl_File_all_pathways/nolabel/Heparan sulfate degradation.pdf]

Oxidative phosphorylation

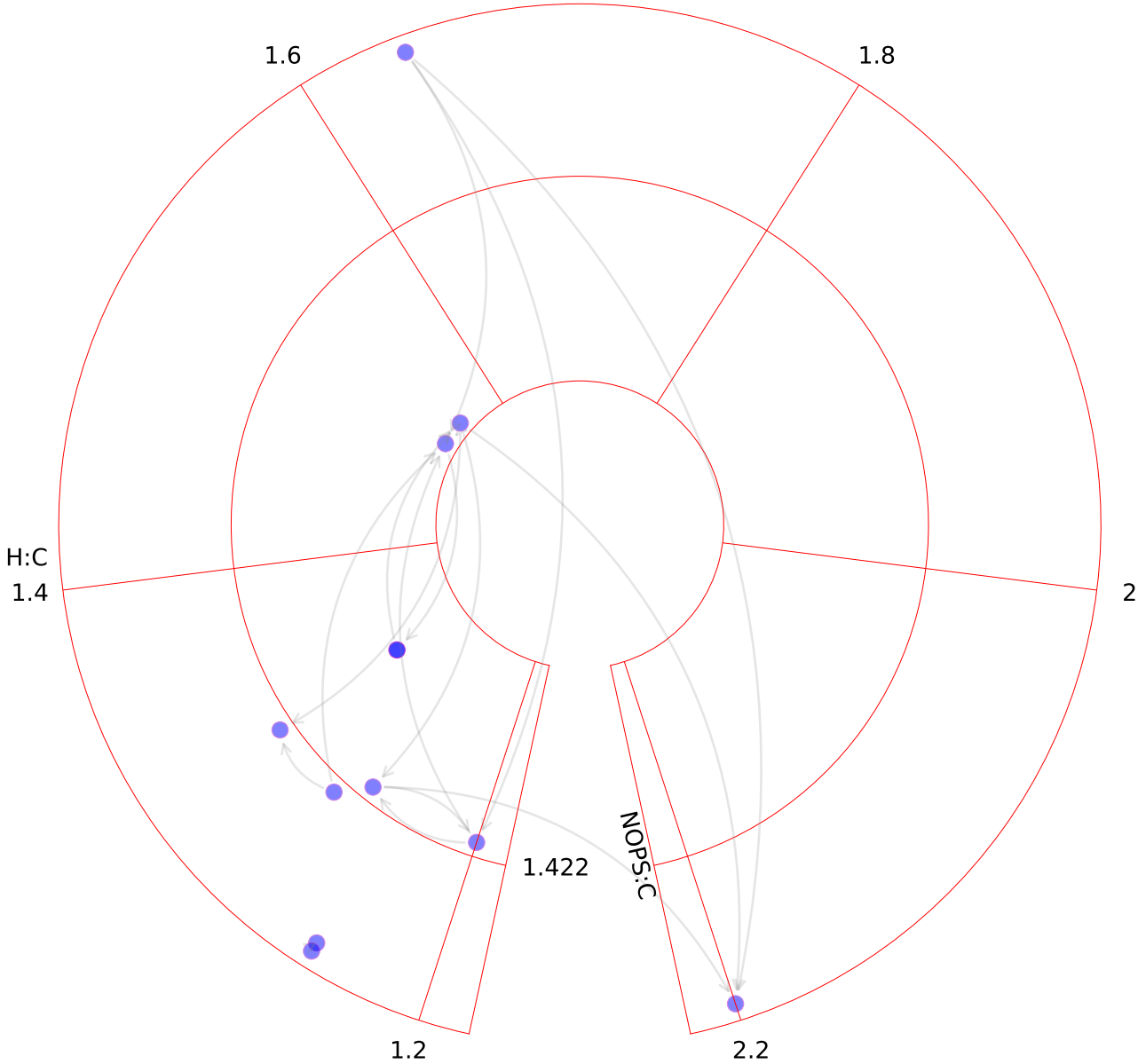

Supplement: Supplement 1 [file media-1.zip › Suppl_File_all_pathways/nolabel/Oxidative phosphorylation.pdf]

# Phosphatidylinositol phosphate metabolism

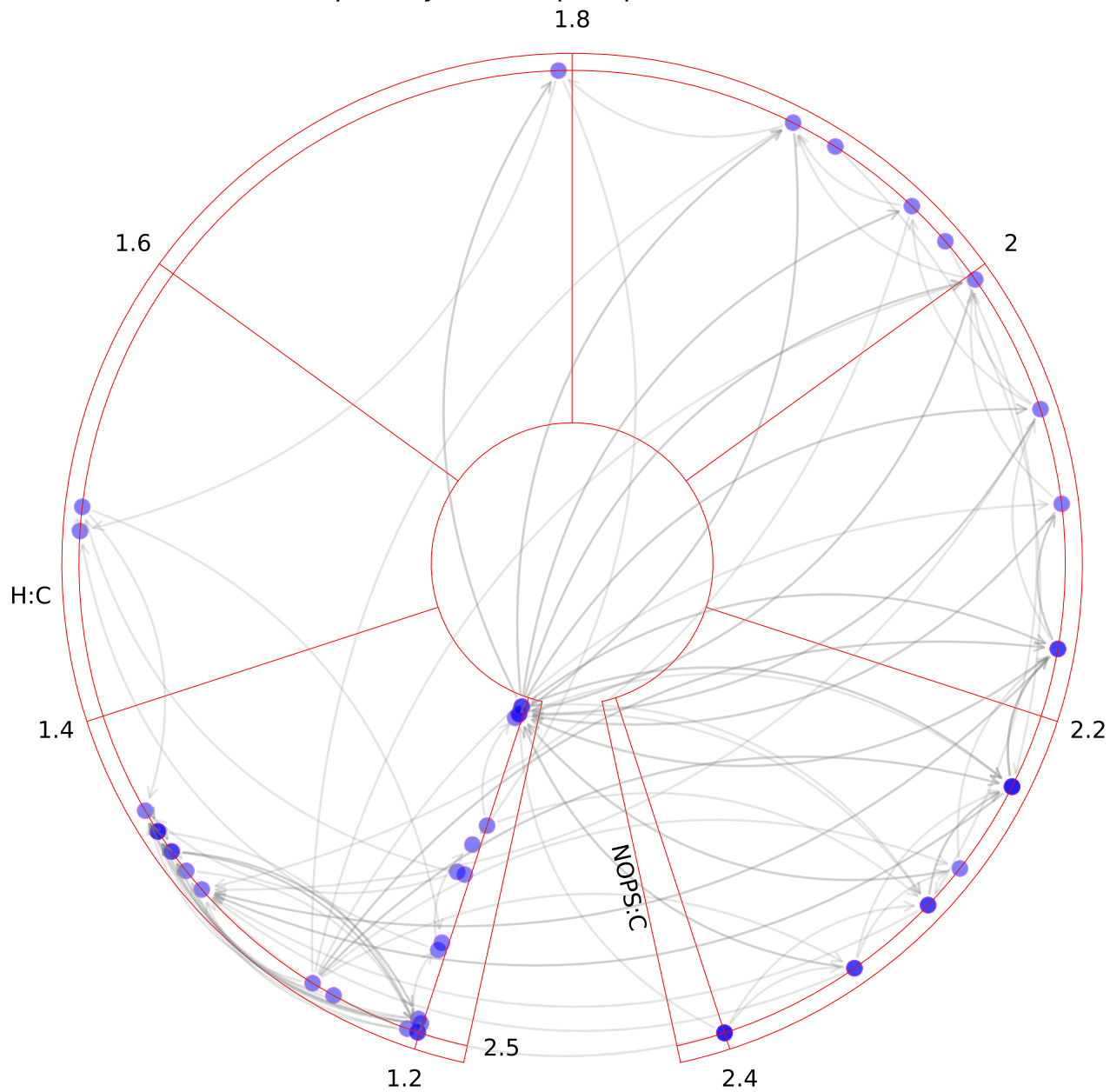

Supplement: Supplement 1 [file media-1.zip › Suppl_File_all_pathways/nolabel/Phosphatidylinositol phosphate metabolism.pdf]

# Glutamate metabolism

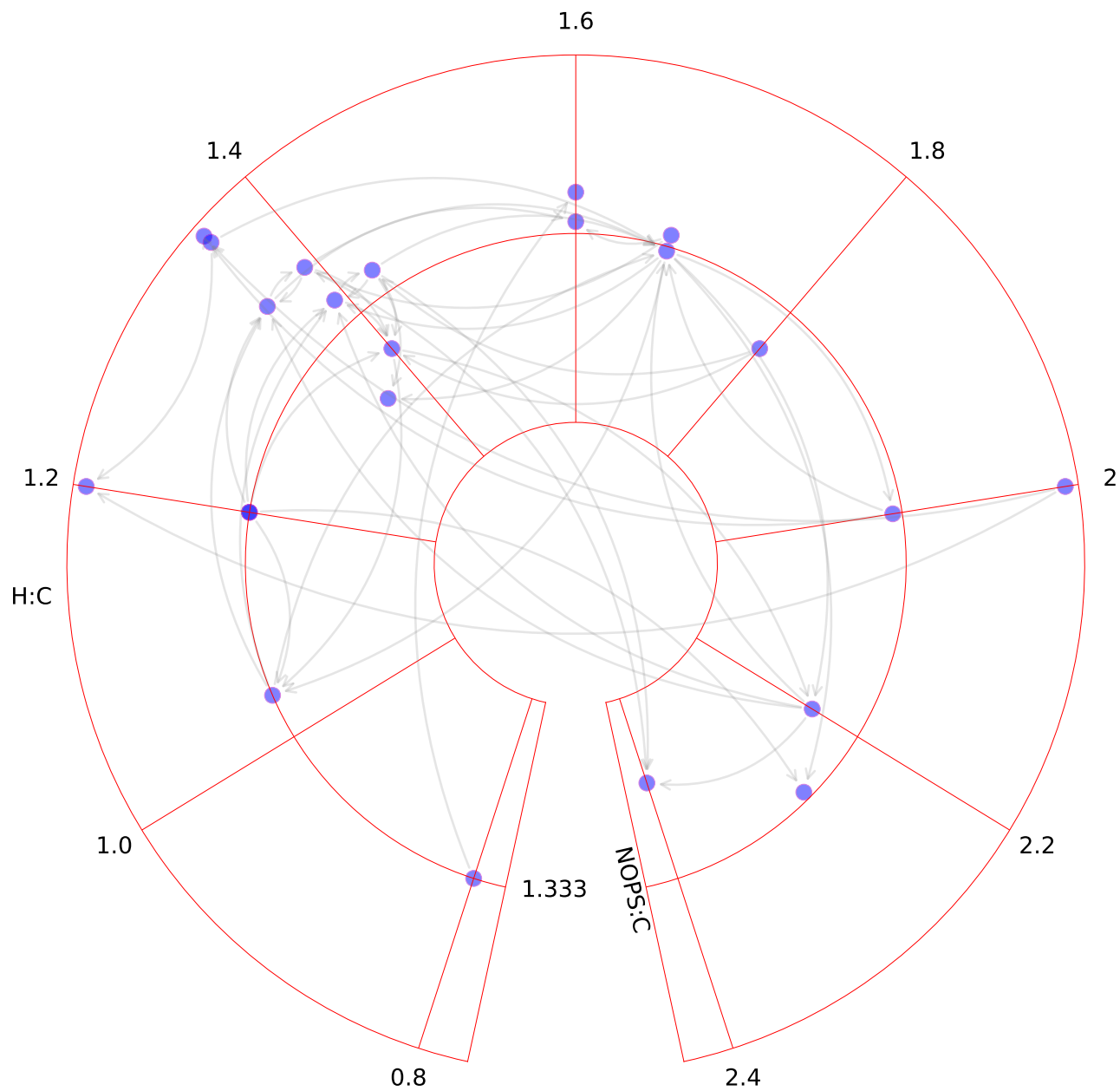

Supplement: Supplement 1 [file media-1.zip › Suppl_File_all_pathways/nolabel/Glutamate metabolism.pdf]

# Beta-Alanine metabolism

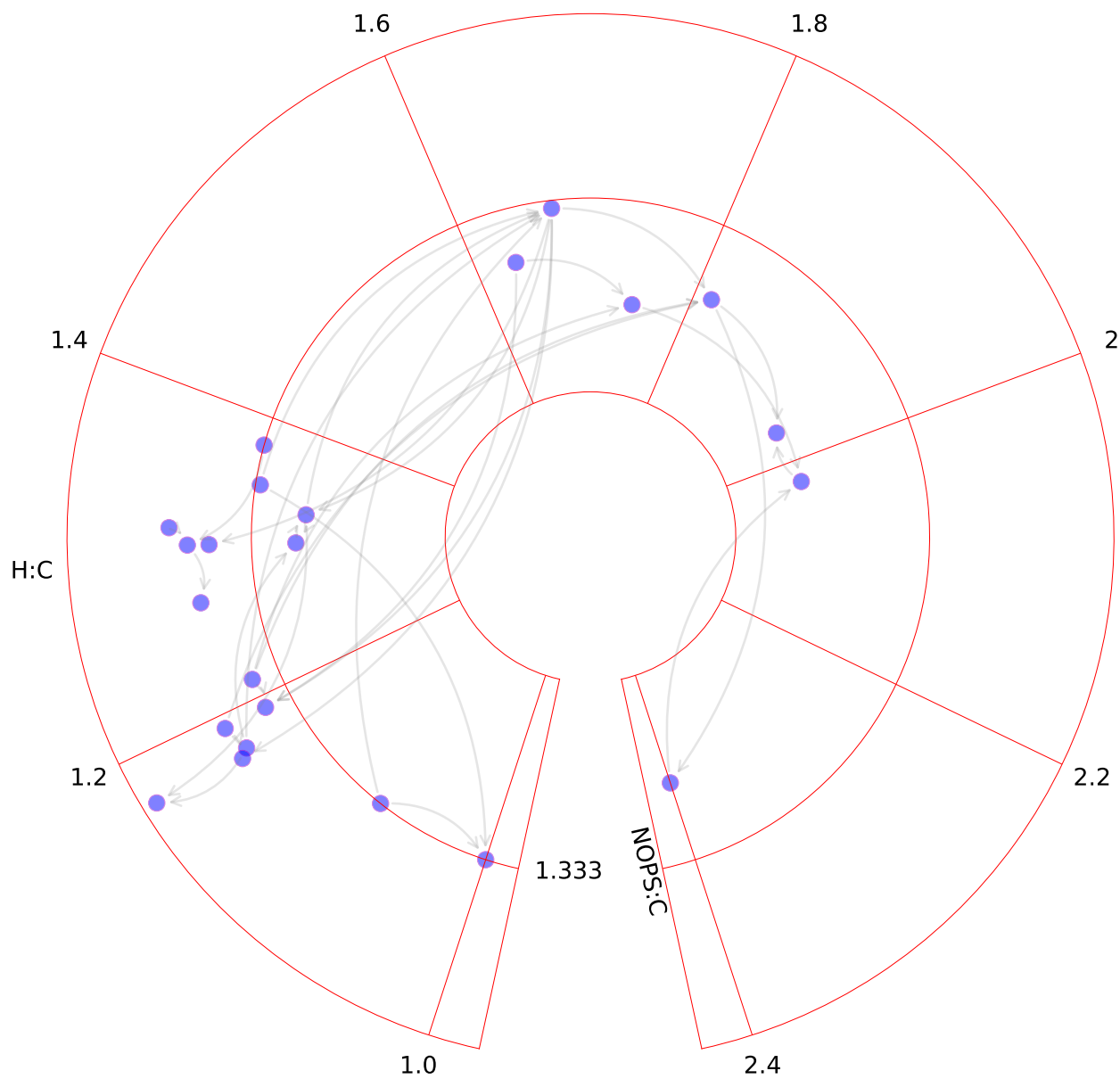

Supplement: Supplement 1 [file media-1.zip › Suppl_File_all_pathways/nolabel/Beta-Alanine metabolism.pdf]

# Histidine metabolism

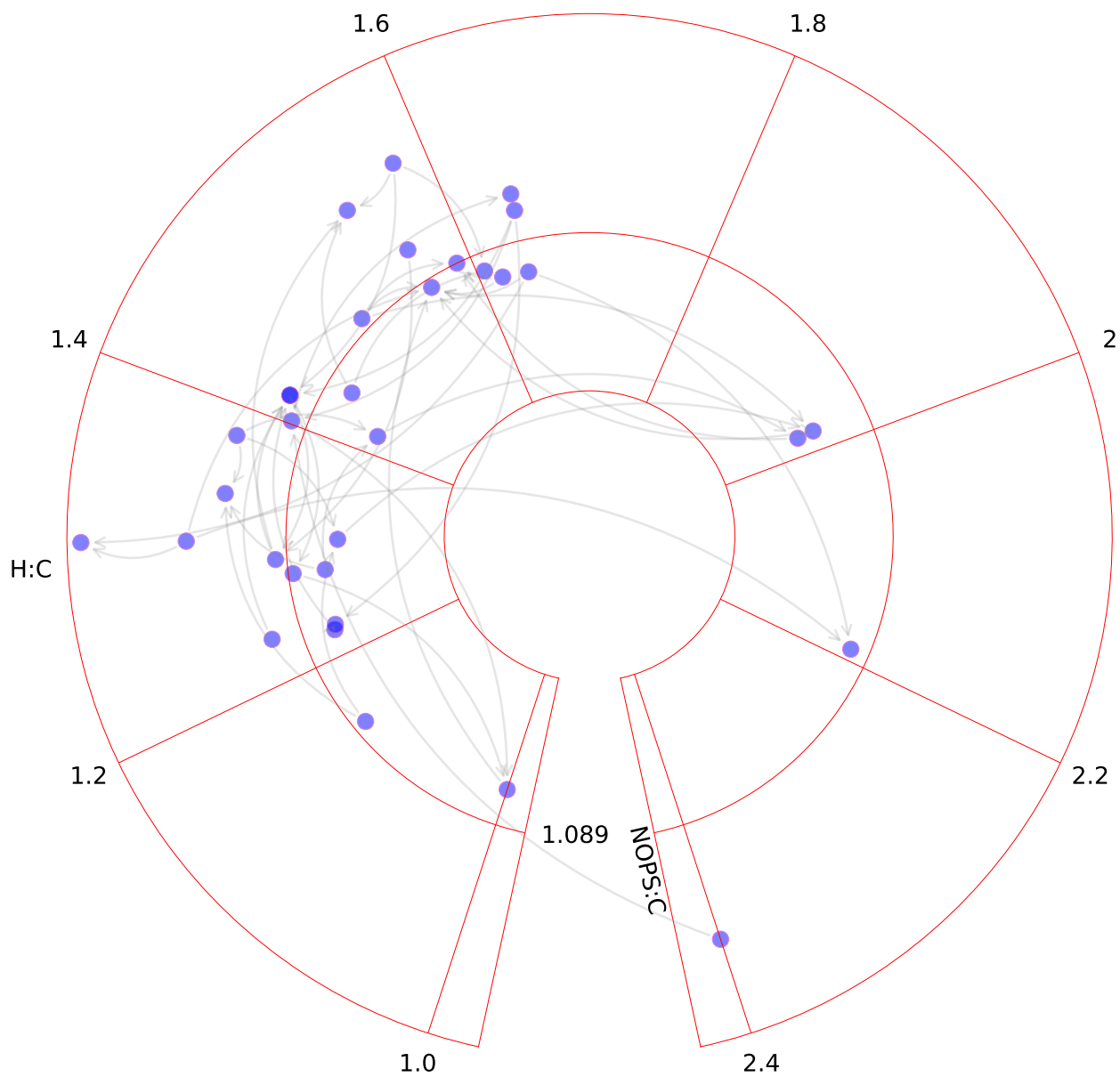

Supplement: Supplement 1 [file media-1.zip › Suppl_File_all_pathways/nolabel/Histidine metabolism.pdf]

# Aminoacyl-tRNA biosynthesis

1.8

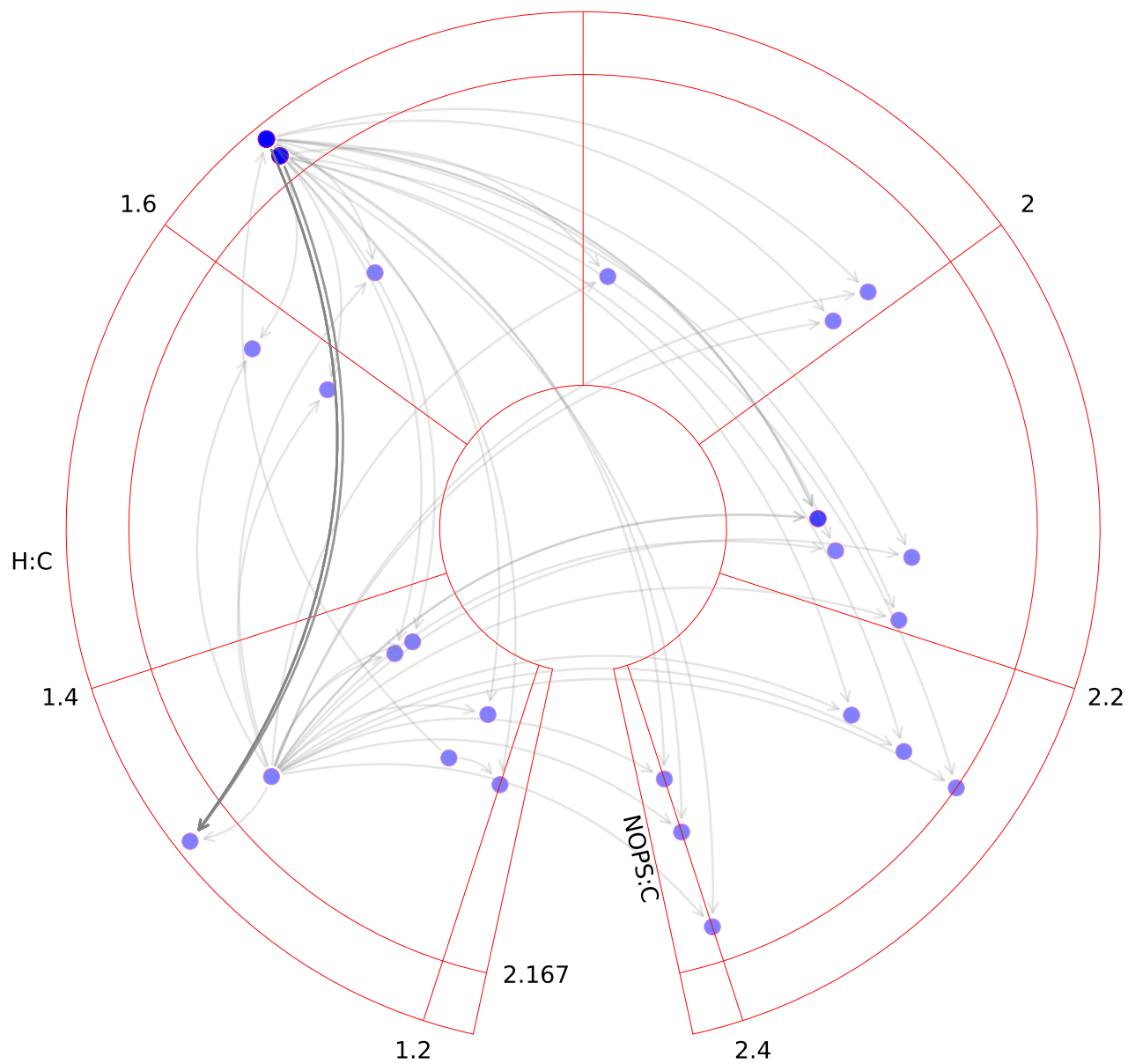

Supplement: Supplement 1 [file media-1.zip › Suppl_File_all_pathways/nolabel/Aminoacyl-tRNA biosynthesis.pdf]

# D-alanine metabolism

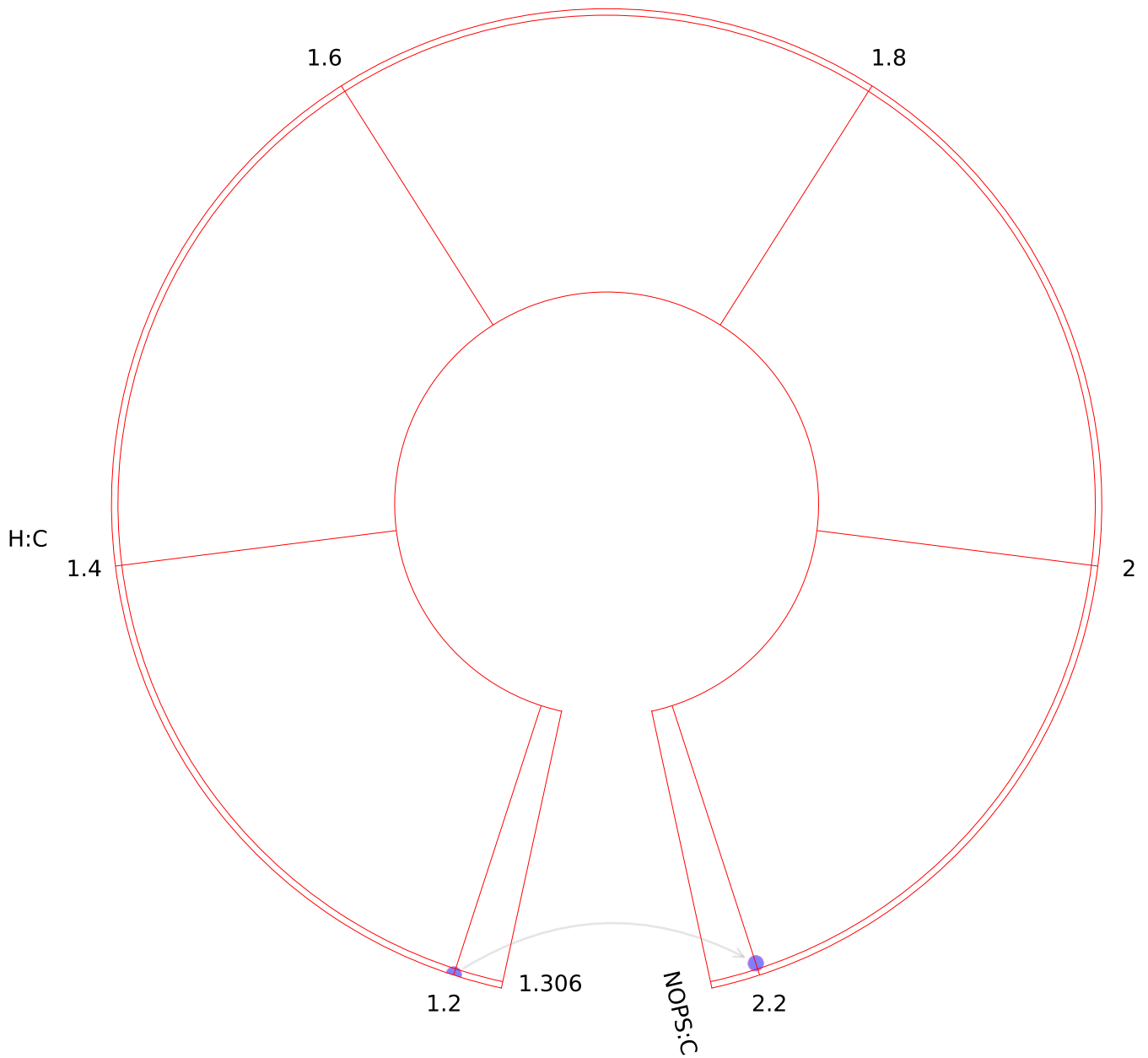

Supplement: Supplement 1 [file media-1.zip › Suppl_File_all_pathways/nolabel/D-alanine metabolism.pdf]

# CoA synthesis

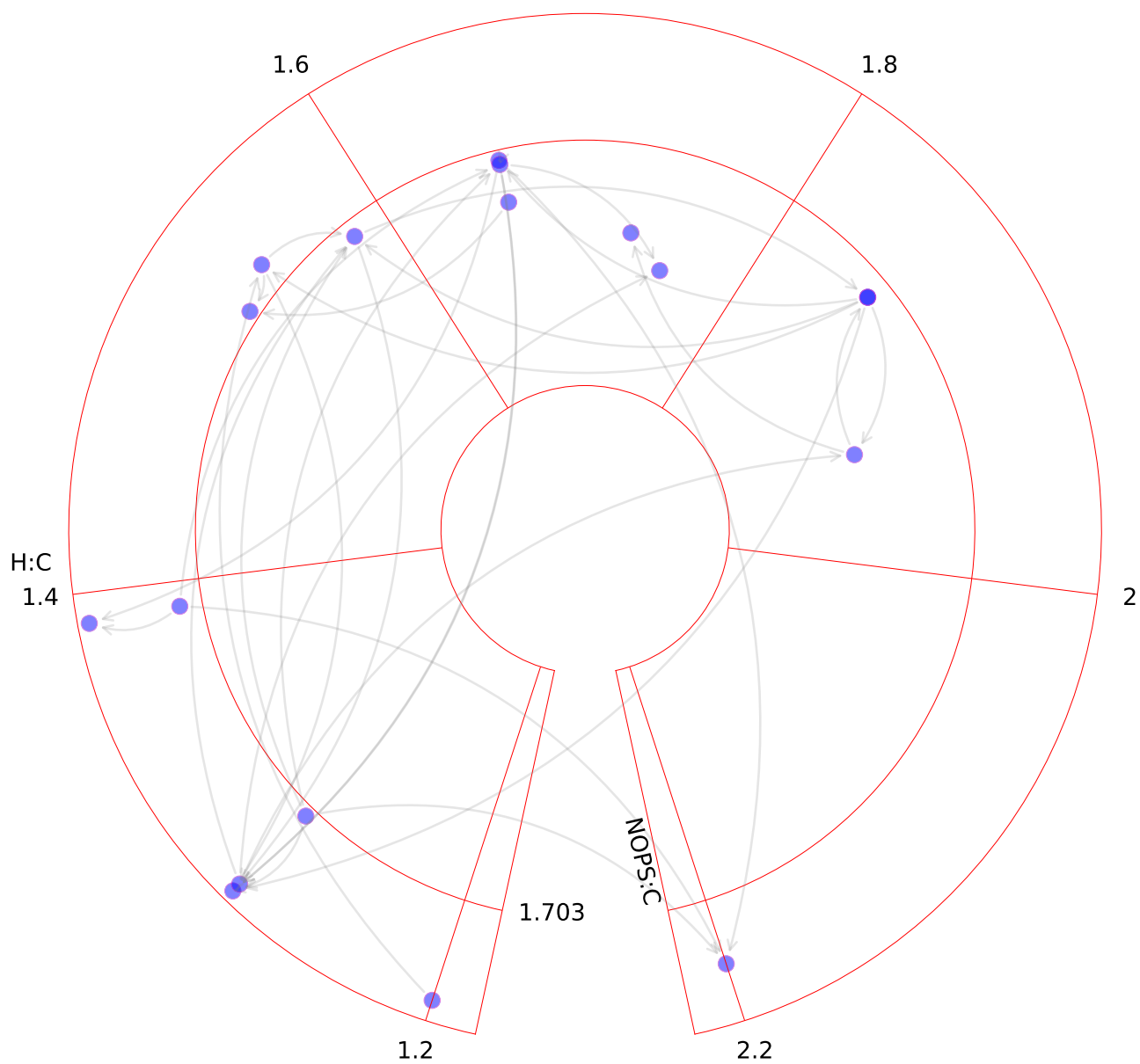

Supplement: Supplement 1 [file media-1.zip › Suppl_File_all_pathways/nolabel/CoA synthesis.pdf]

# Linoleate metabolism

1.6

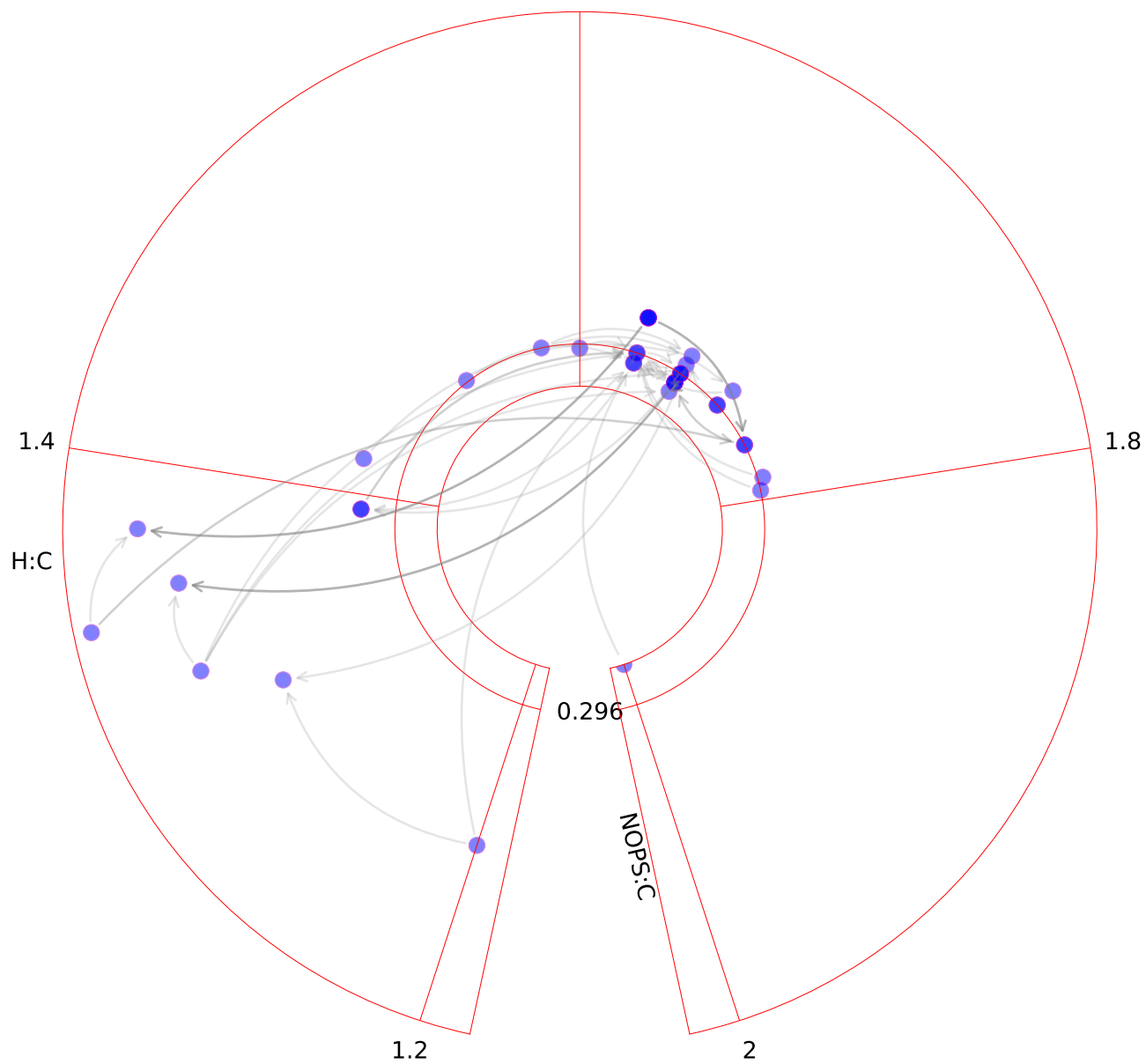

Supplement: Supplement 1 [file media-1.zip › Suppl_File_all_pathways/nolabel/Linoleate metabolism.pdf]

# Keratan sulfate synthesis

1.4

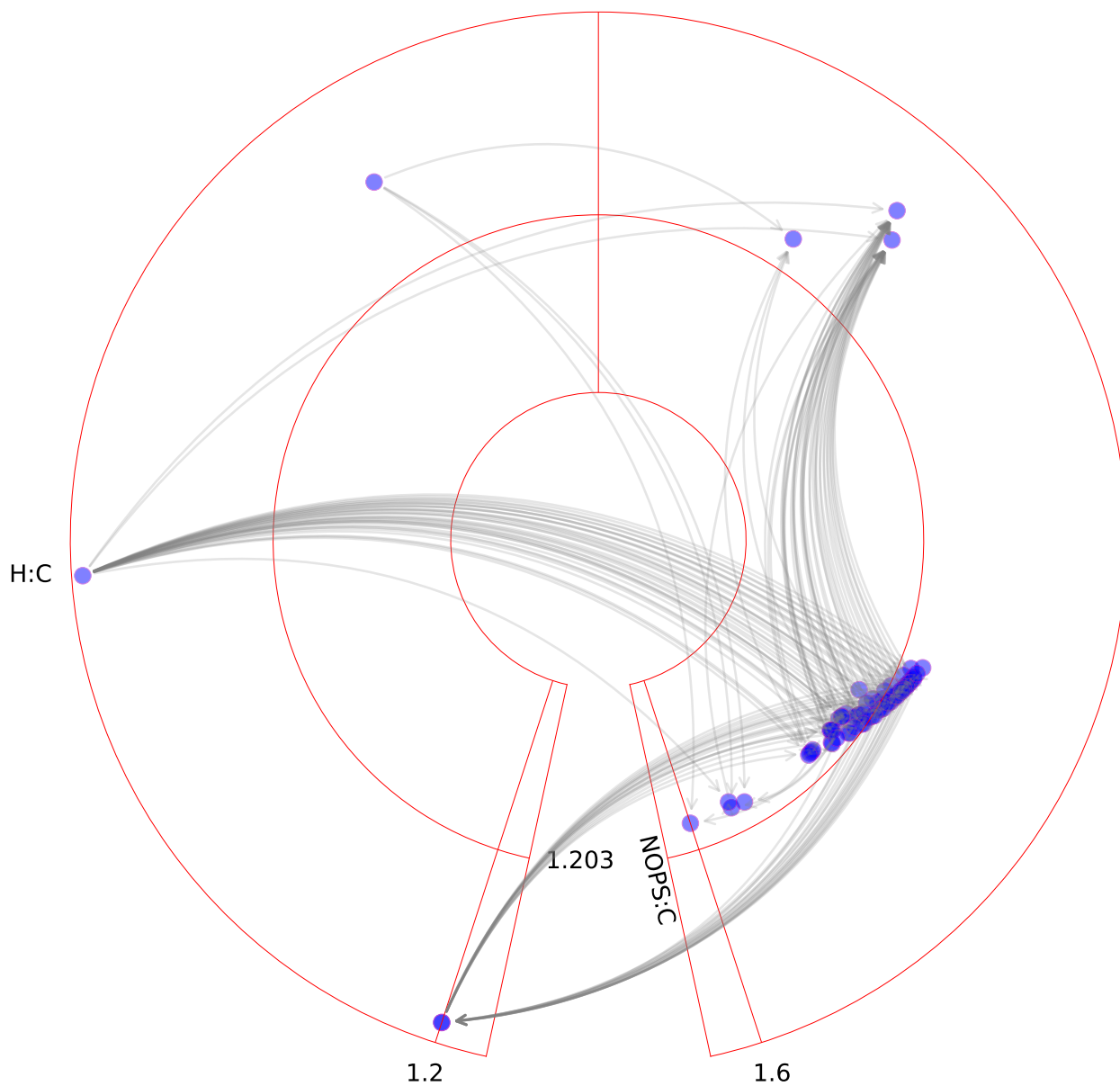

Supplement: Supplement 1 [file media-1.zip › Suppl_File_all_pathways/nolabel/Keratan sulfate synthesis.pdf]

# Heme synthesis

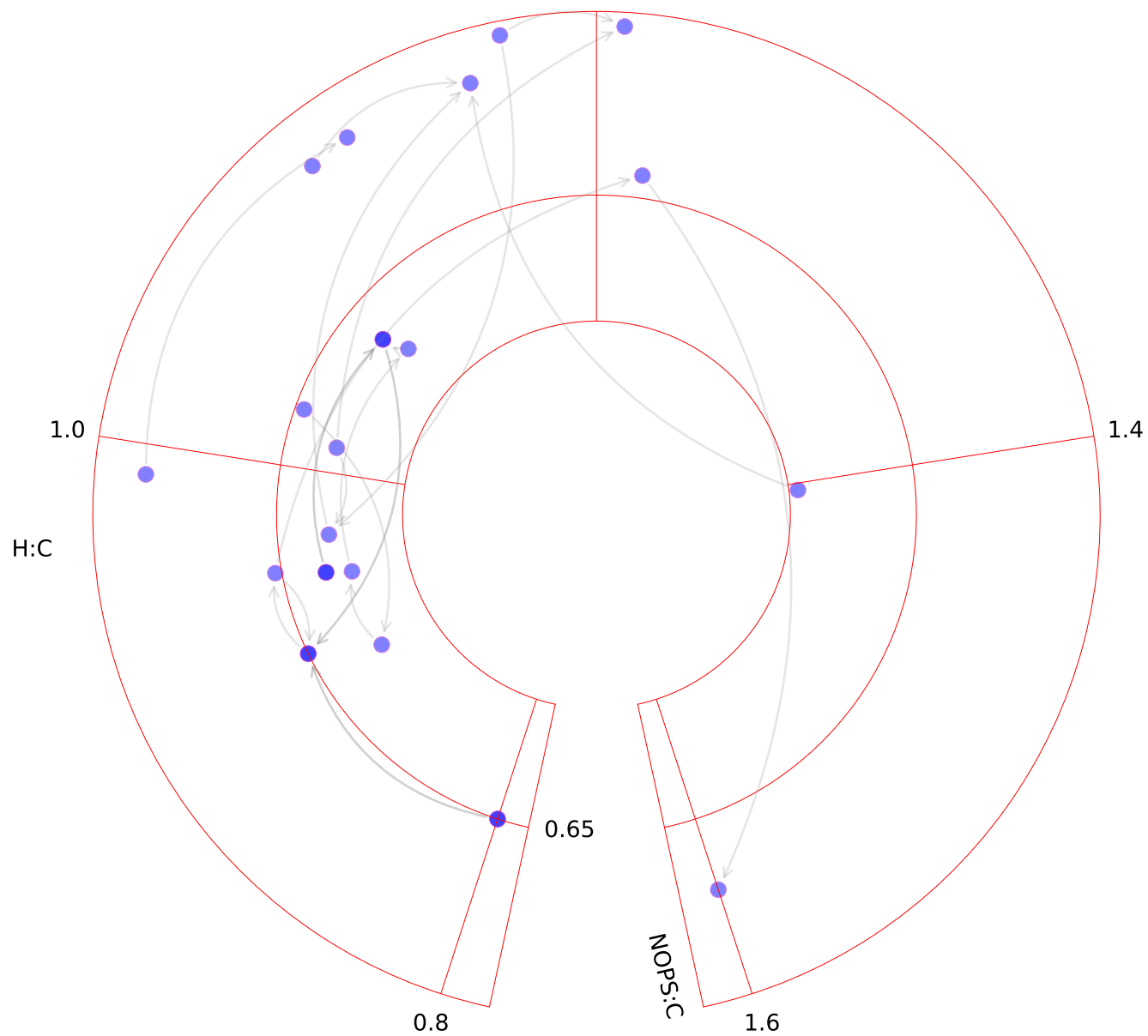

Supplement: Supplement 1 [file media-1.zip › Suppl_File_all_pathways/nolabel/Heme synthesis.pdf]

# Glycine, serine, alanine, and threonine metabolism

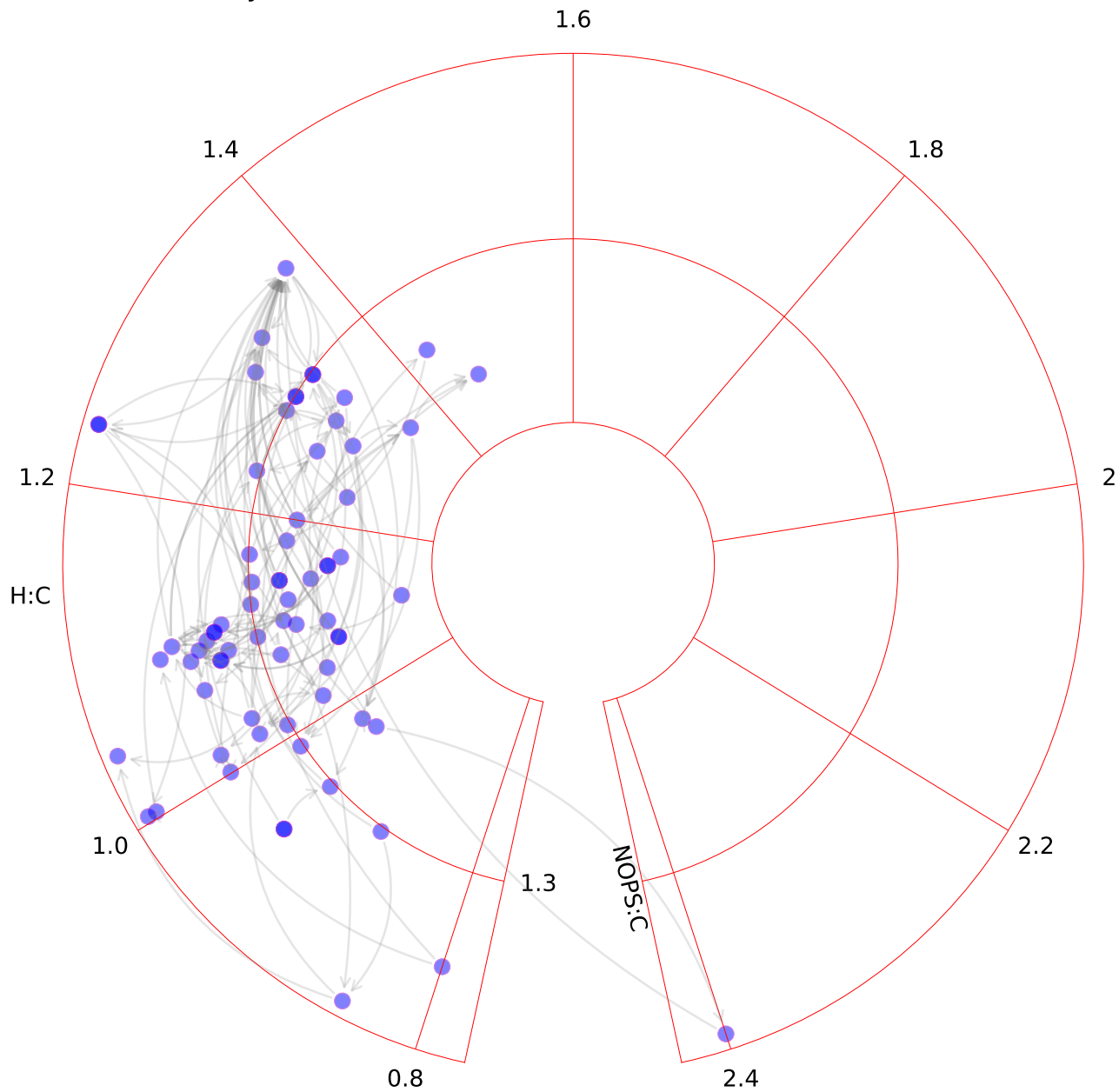

Supplement: Supplement 1 [file media-1.zip › Suppl_File_all_pathways/nolabel/Glycine, serine, alanine, and threonine metabolism.pdf]

# Ubiquinone synthesis

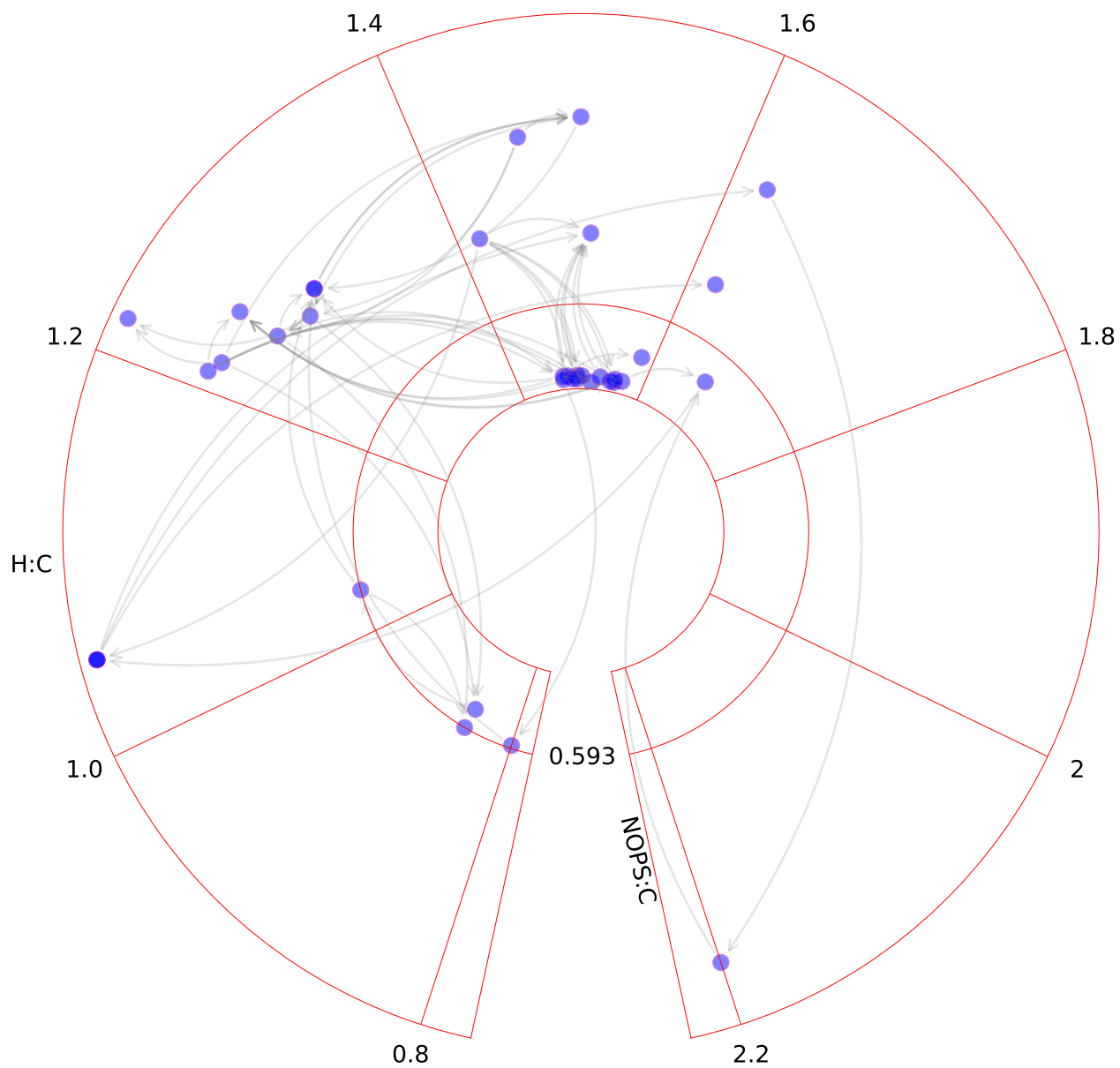

Supplement: Supplement 1 [file media-1.zip › Suppl_File_all_pathways/nolabel/Ubiquinone synthesis.pdf]

# NAD metabolism

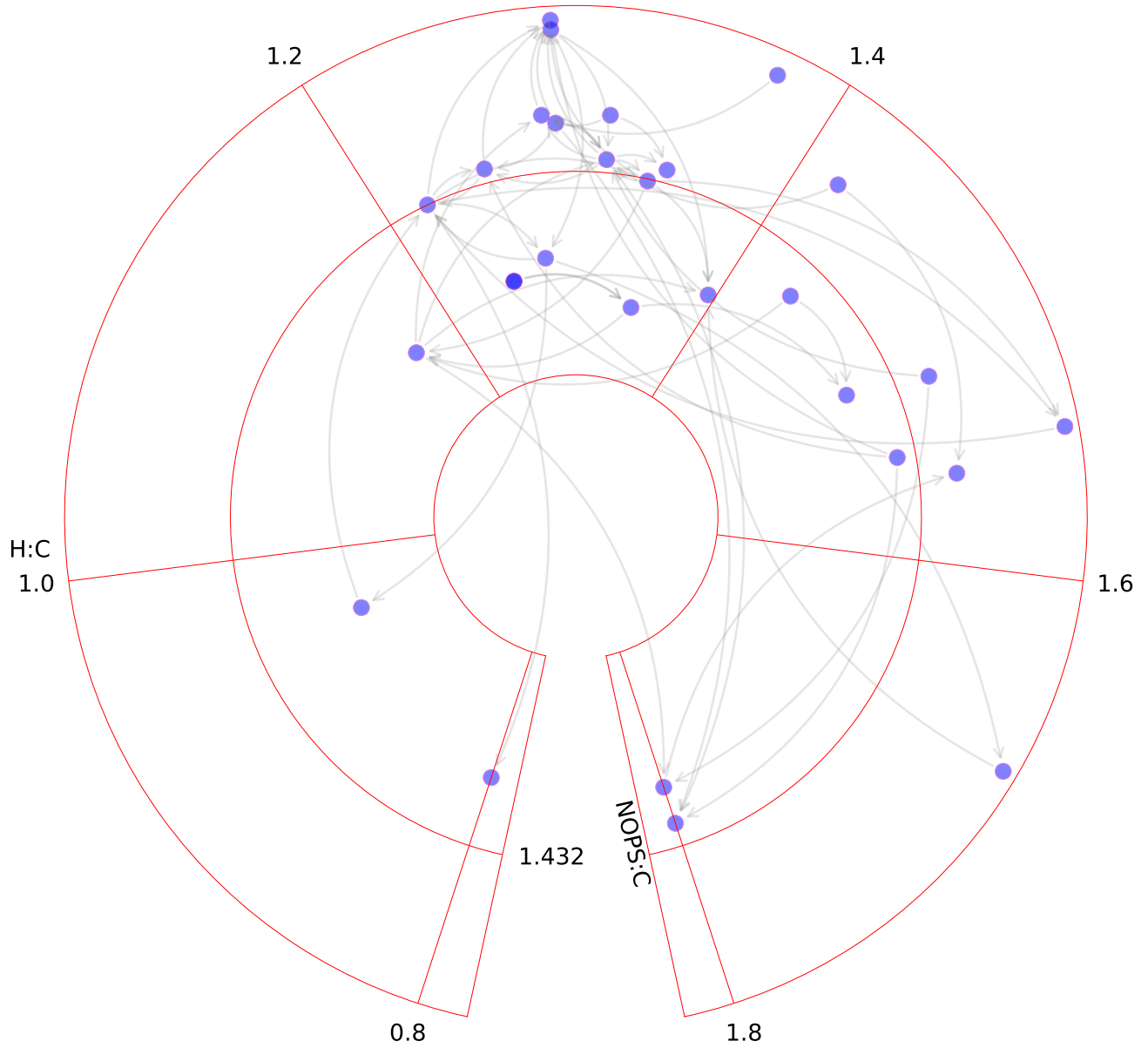

Supplement: Supplement 1 [file media-1.zip › Suppl_File_all_pathways/nolabel/NAD metabolism.pdf]

# Stilbene, coumarine and lignin synthesis

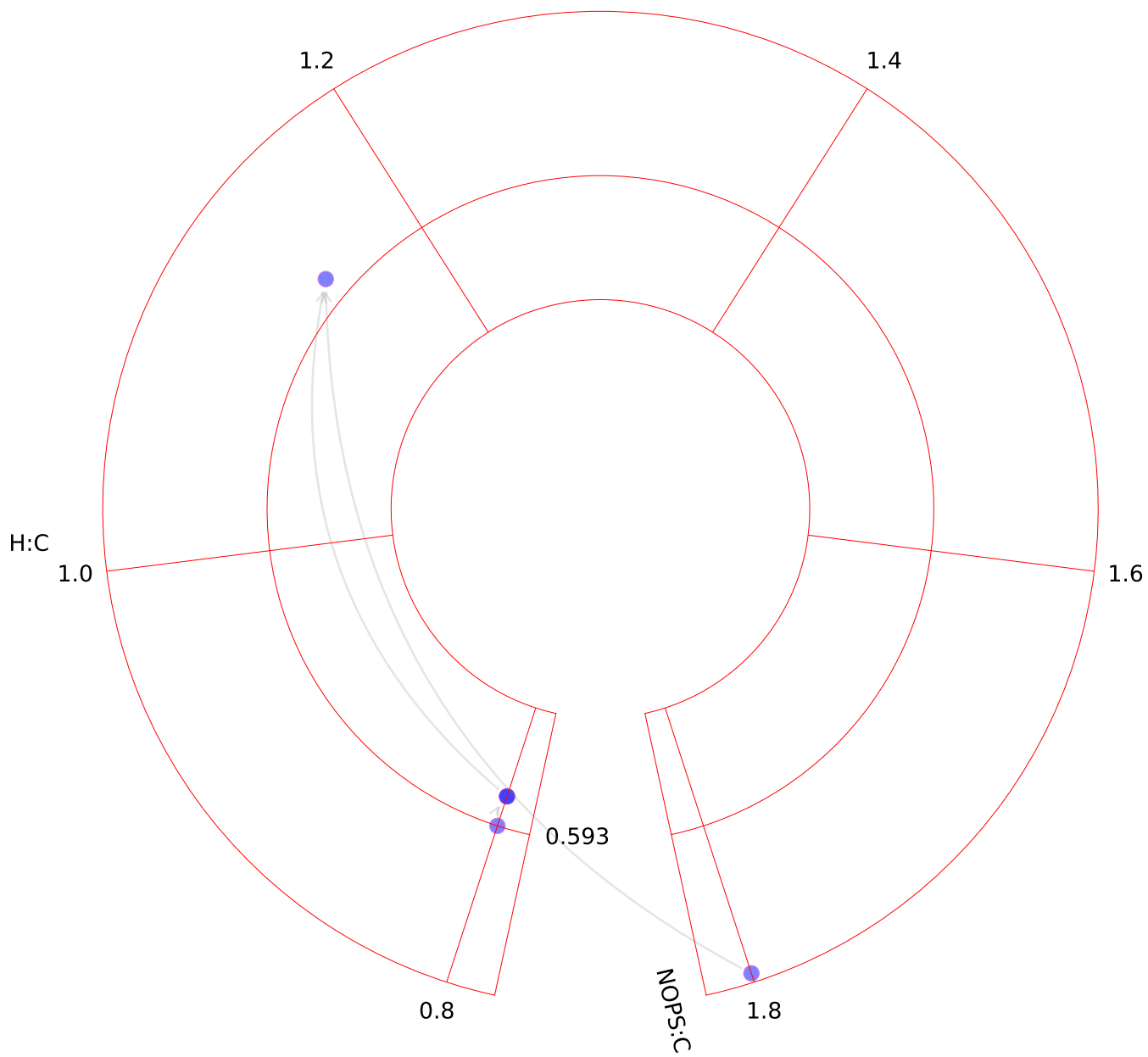

Supplement: Supplement 1 [file media-1.zip › Suppl_File_all_pathways/nolabel/Stilbene, coumarine and lignin synthesis.pdf]

## 2

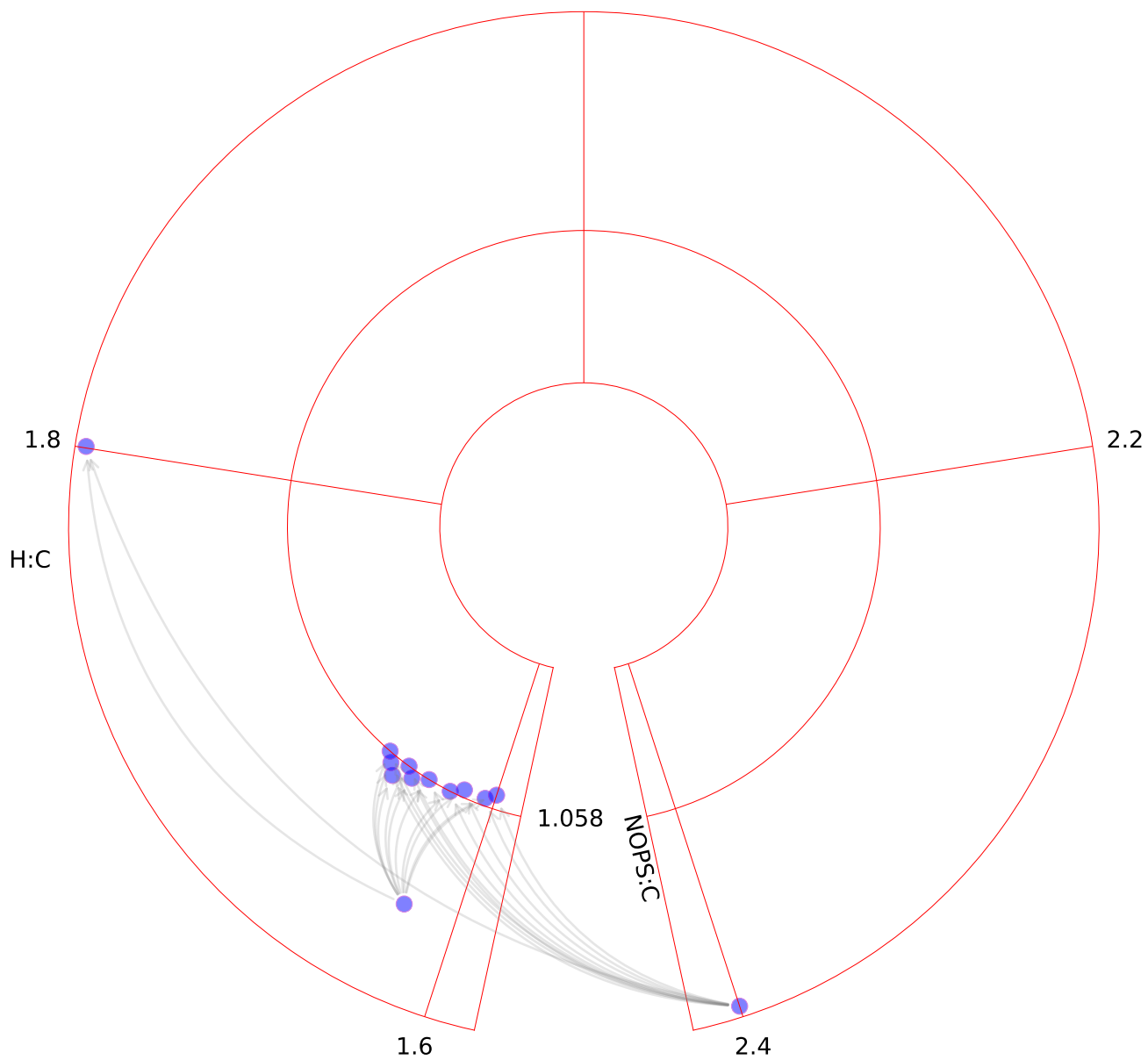

Supplement: Supplement 1 [file media-1.zip › Suppl_File_all_pathways/nolabel/Triglycerides formation.pdf]

# Tyrosine metabolism

1.6

1.4

1.8

2

1.2

H:C

1.0

0.667

2.2

0.8

2.4

NOP5:C

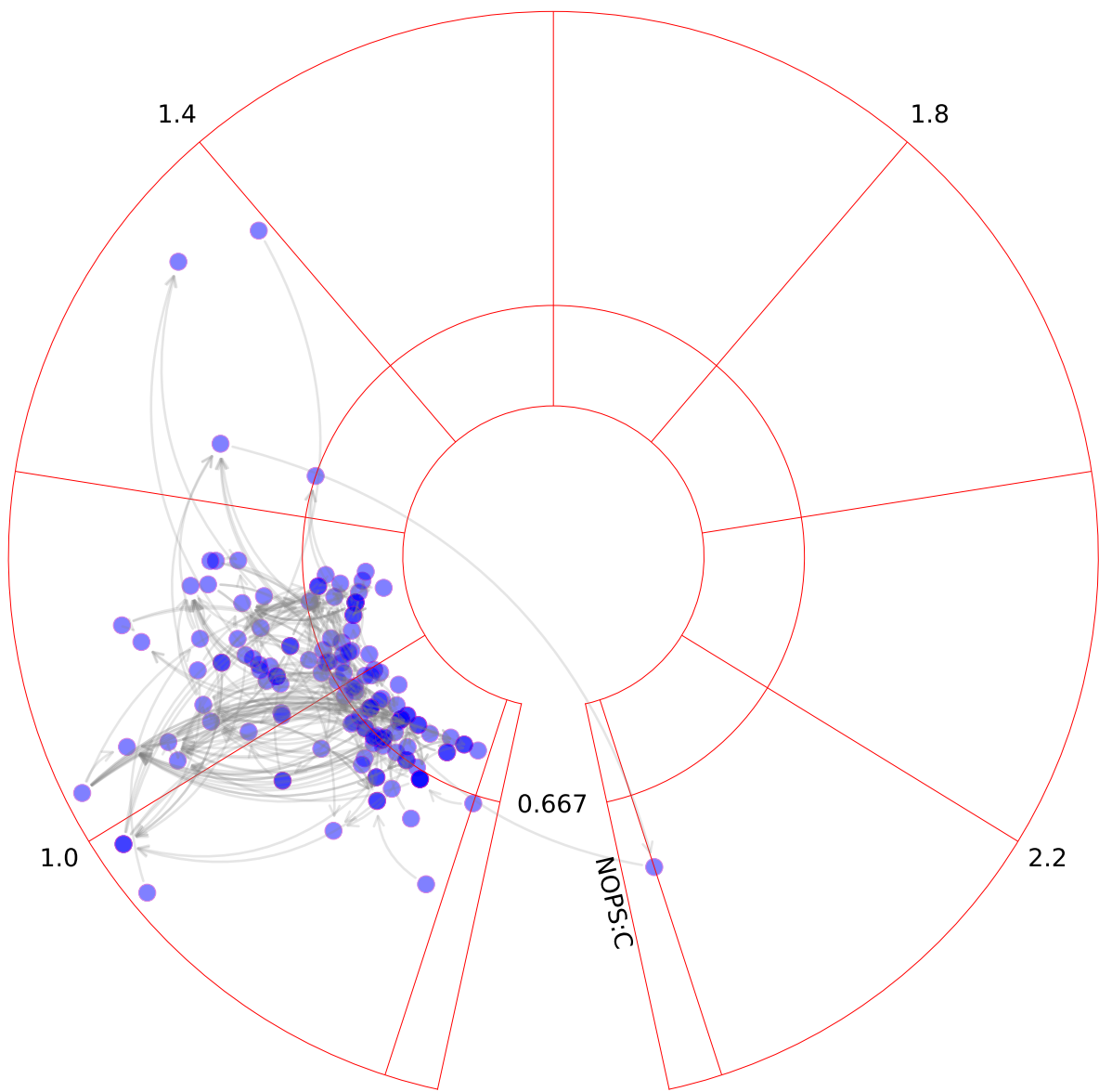

Supplement: Supplement 1 [file media-1.zip › Suppl_File_all_pathways/nolabel/Tyrosine metabolism.pdf]

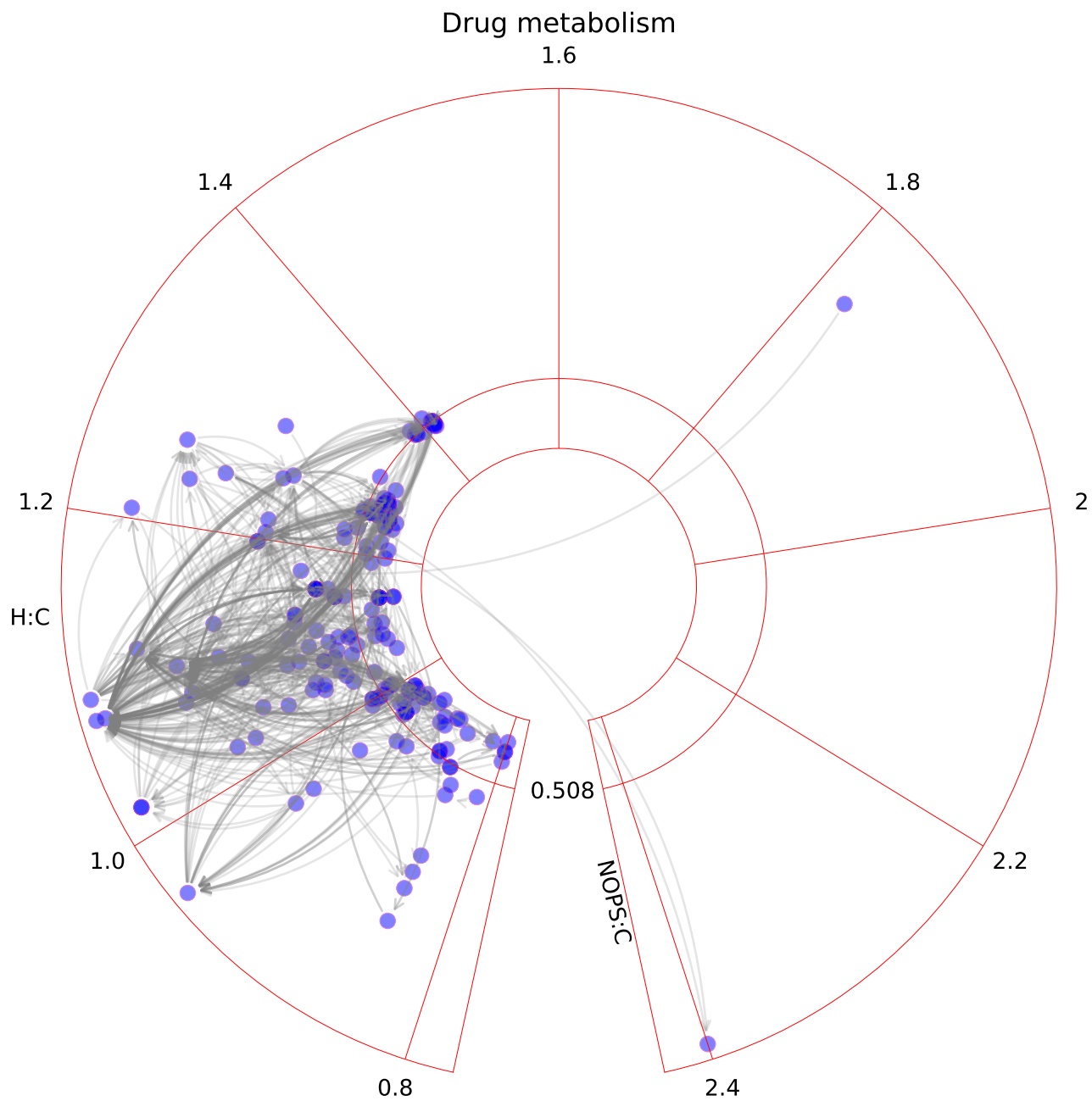

Supplement: Supplement 1 [file media-1.zip › Suppl_File_all_pathways/nolabel/Drug metabolism.pdf]

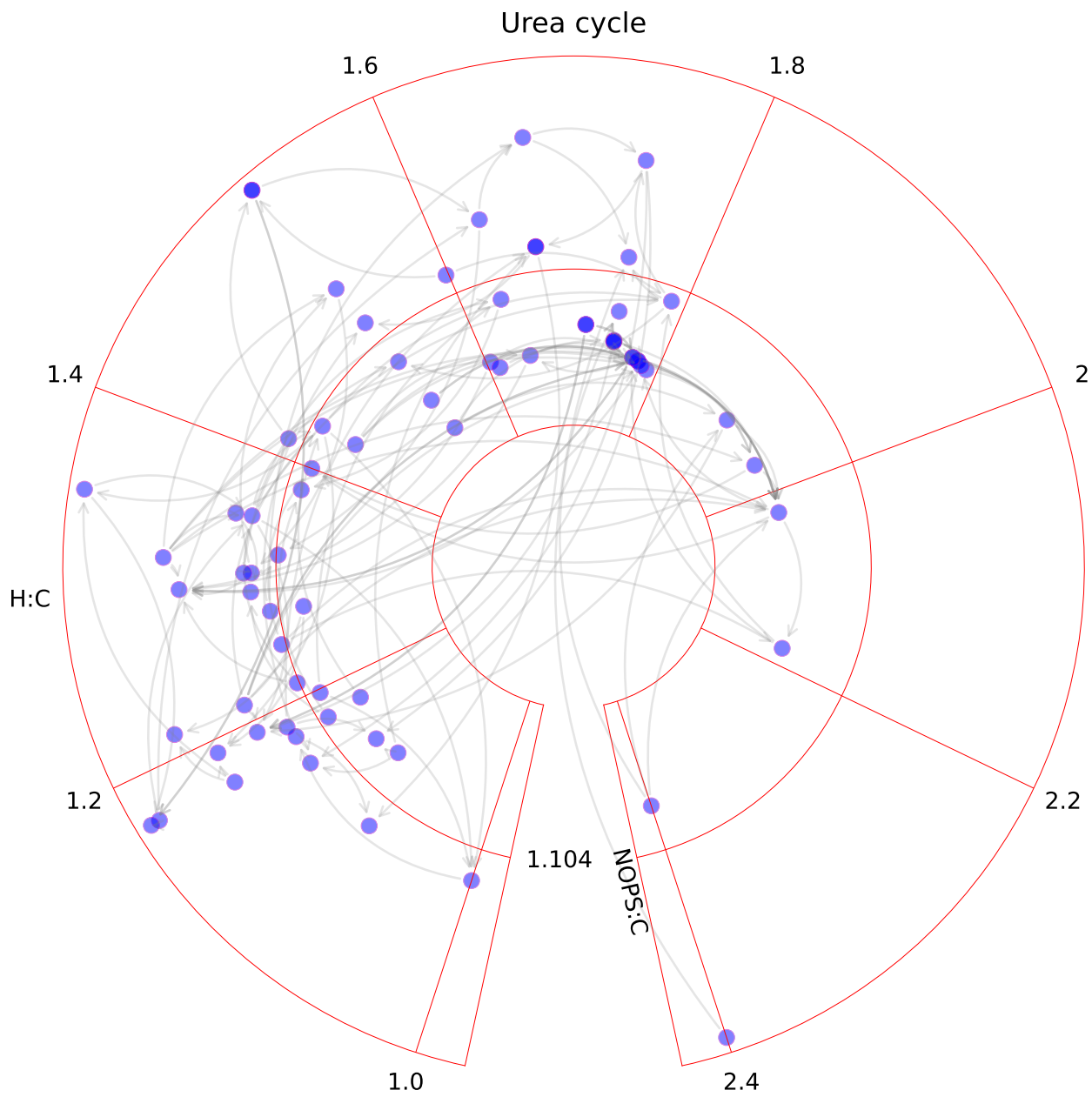

Supplement: Supplement 1 [file media-1.zip › Suppl_File_all_pathways/nolabel/Urea cycle.pdf]

# Vitamin B12 metabolism

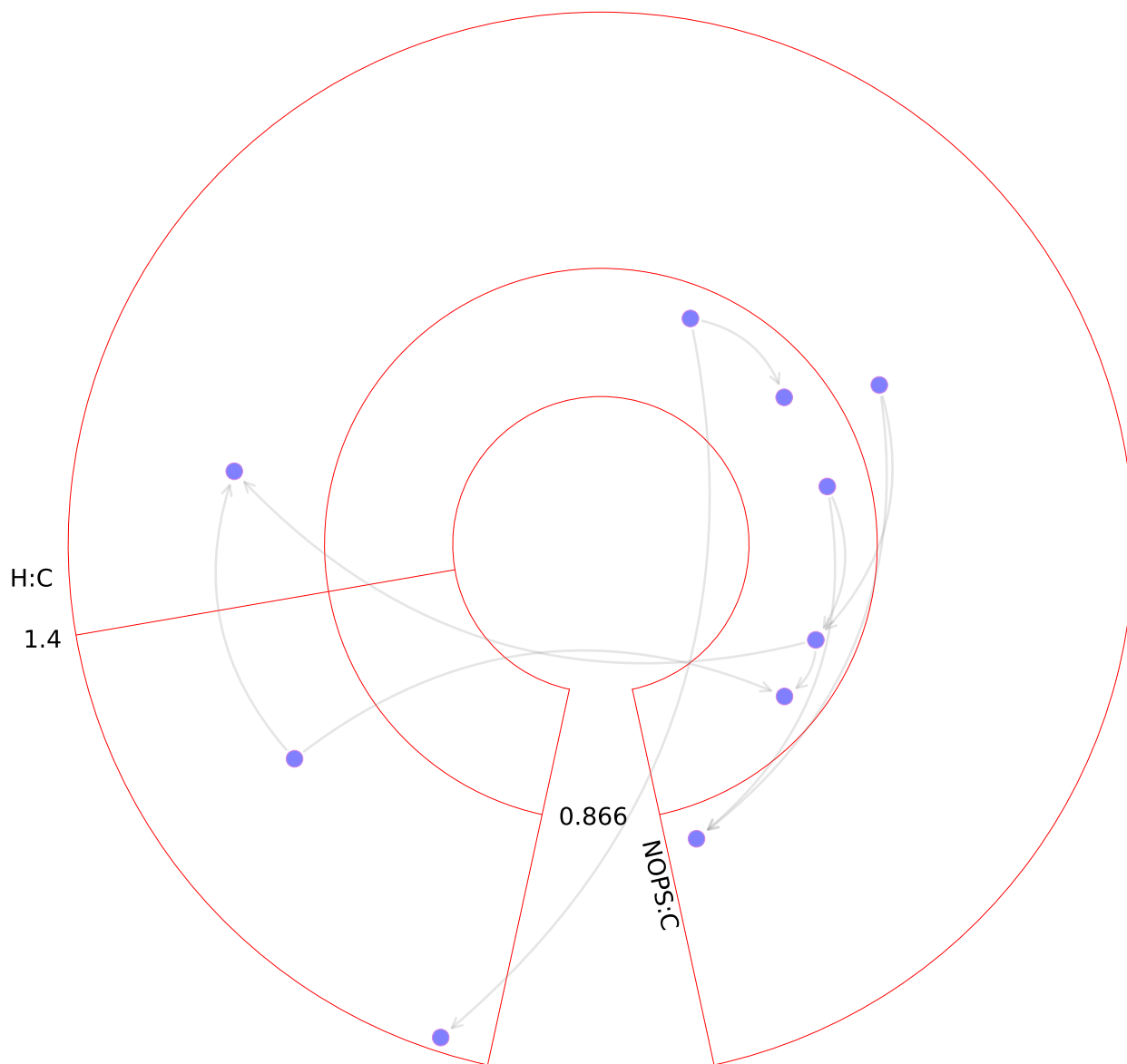

Supplement: Supplement 1 [file media-1.zip › Suppl_File_all_pathways/nolabel/Vitamin B12 metabolism.pdf]

# C5-branched dibasic acid metabolism

1.2

H:C

1.0

1.767

NOPS:C

1.4

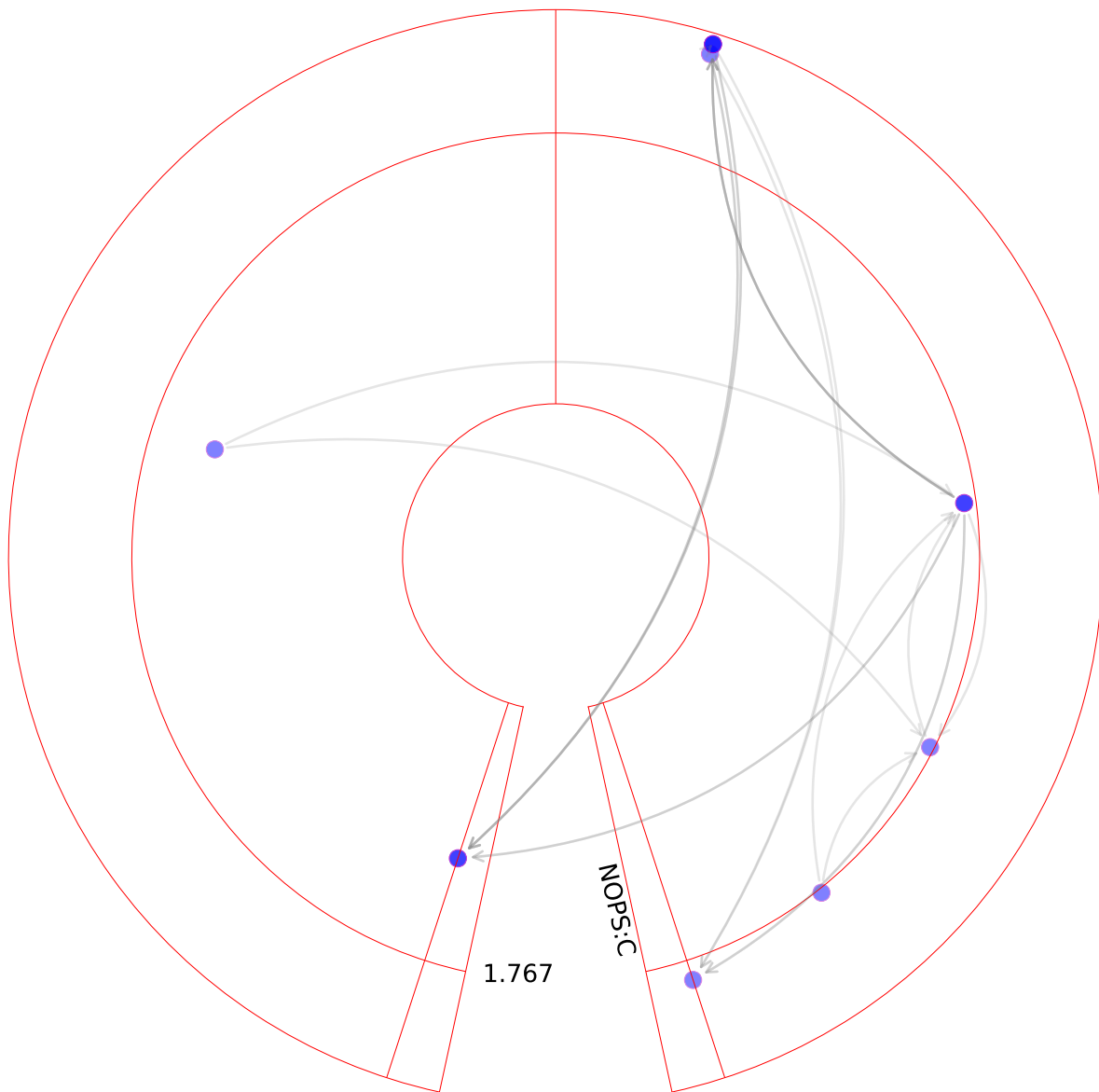

Supplement: Supplement 1 [file media-1.zip › Suppl_File_all_pathways/nolabel/C5-branched dibasic acid metabolism.pdf]

Nucleotide salvage pathway

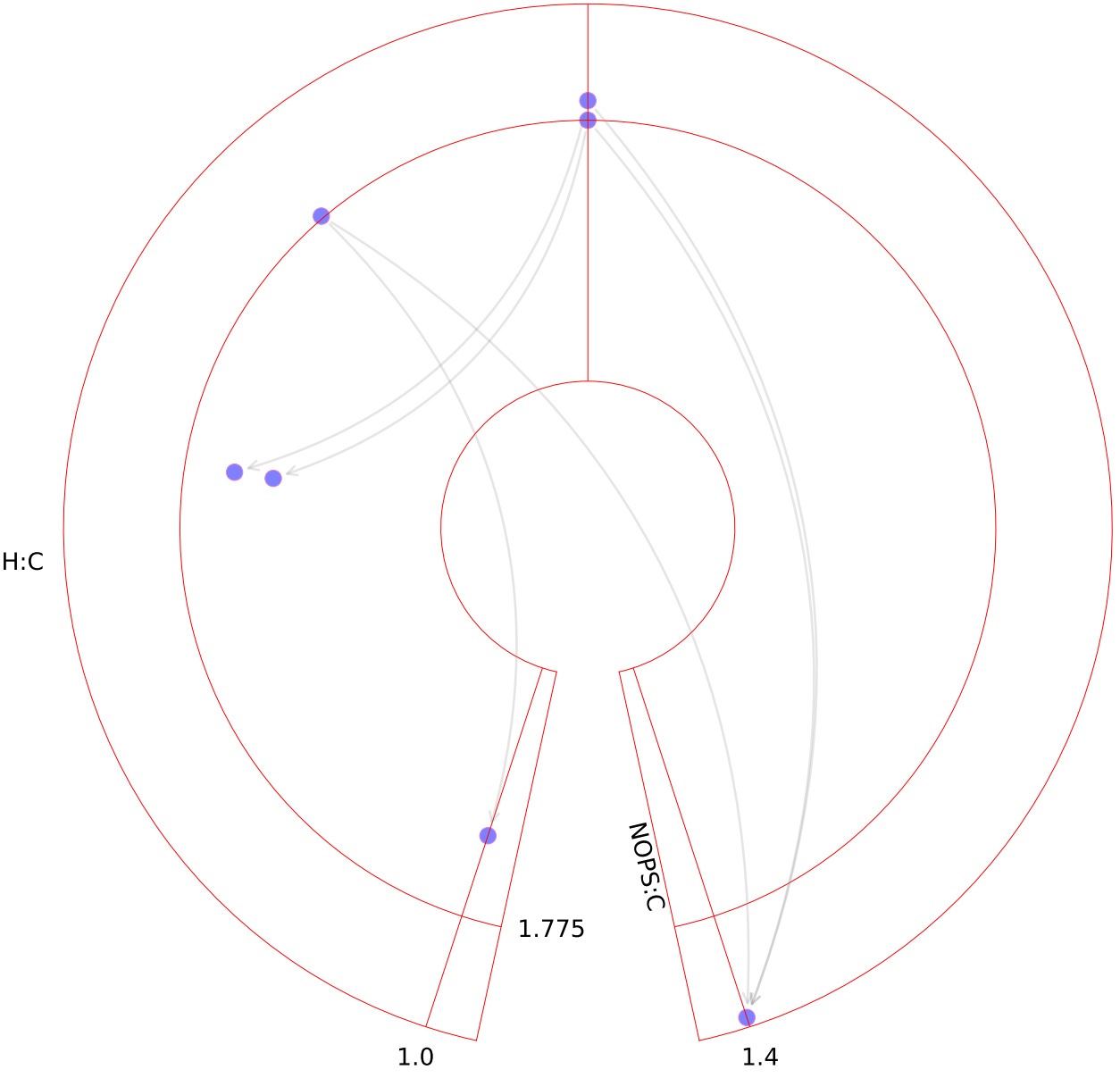

Supplement: Supplement 1 [file media-1.zip › Suppl_File_all_pathways/nolabel/Nucleotide salvage pathway.pdf]

# Sphingolipid metabolism

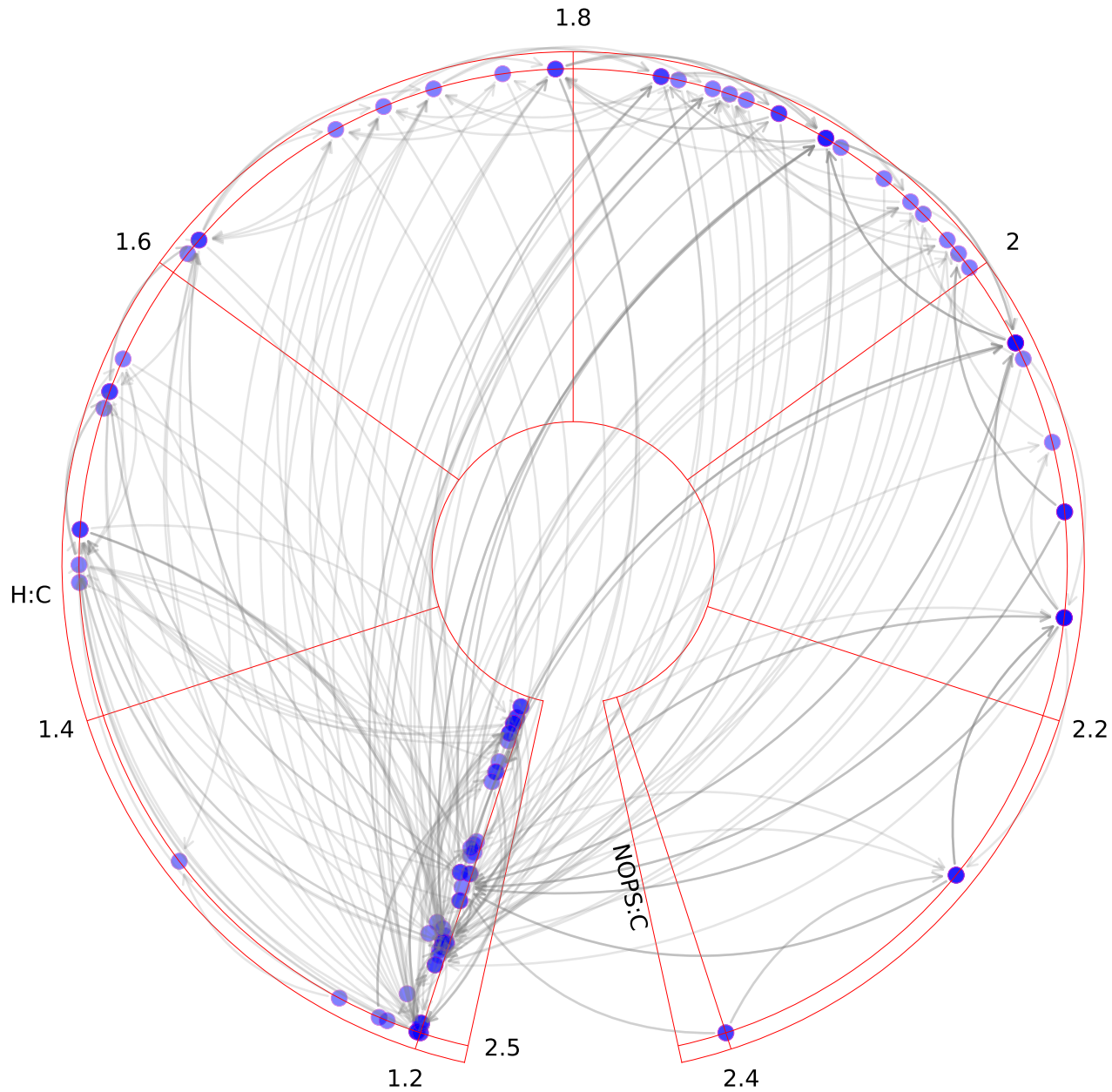

Supplement: Supplement 1 [file media-1.zip › Suppl_File_all_pathways/nolabel/Sphingolipid metabolism.pdf]

# Xenobiotics metabolism

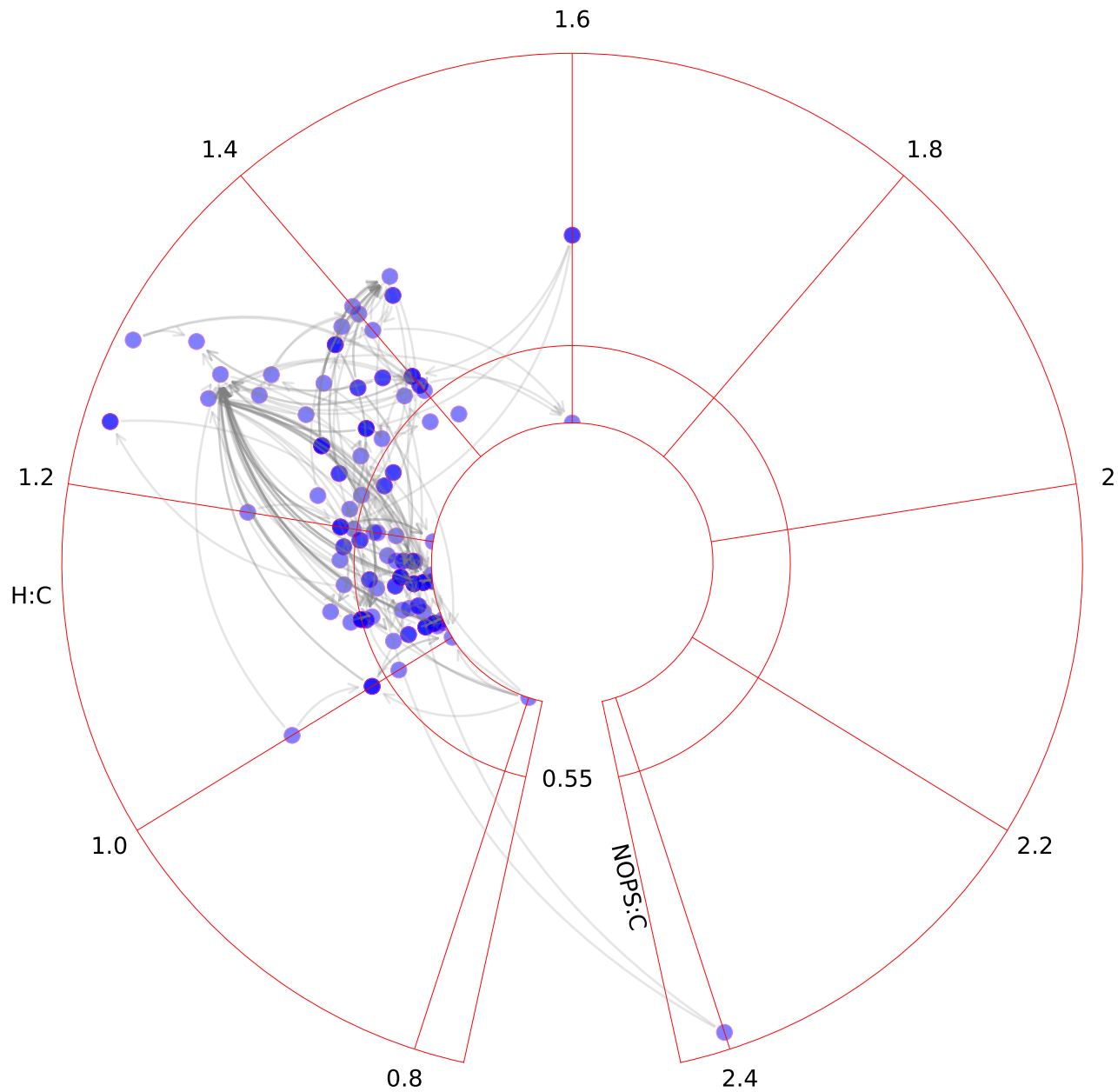

Supplement: Supplement 1 [file media-1.zip › Suppl_File_all_pathways/nolabel/Xenobiotics metabolism.pdf]

# N-glycan metabolism

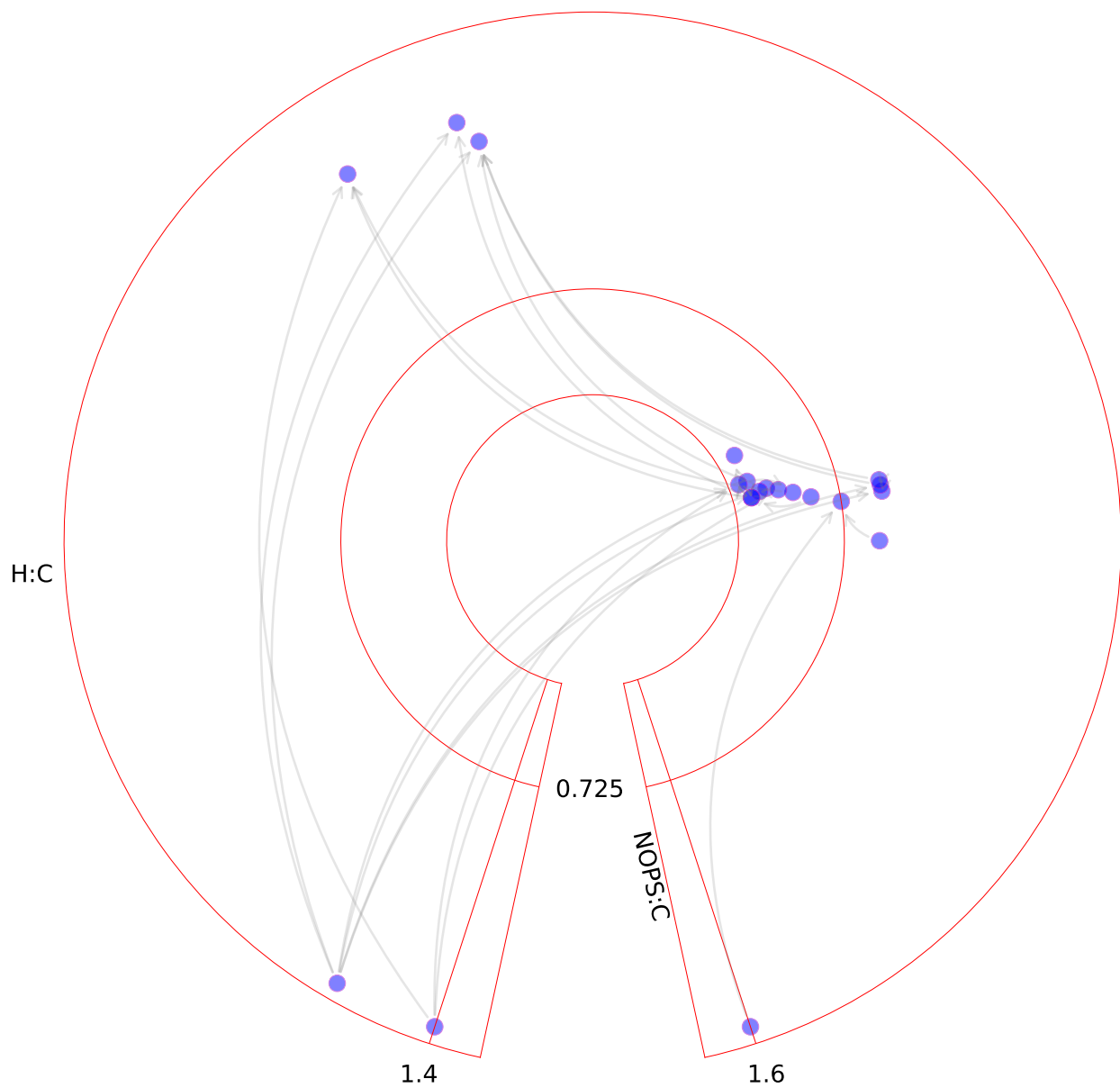

Supplement: Supplement 1 [file media-1.zip › Suppl_File_all_pathways/nolabel/N-glycan metabolism.pdf]

# Leukotriene metabolism

1.8

2

2.2

2.4

0.4

1.2

1.4

1.6

H:C

NO<sub>2</sub>:C

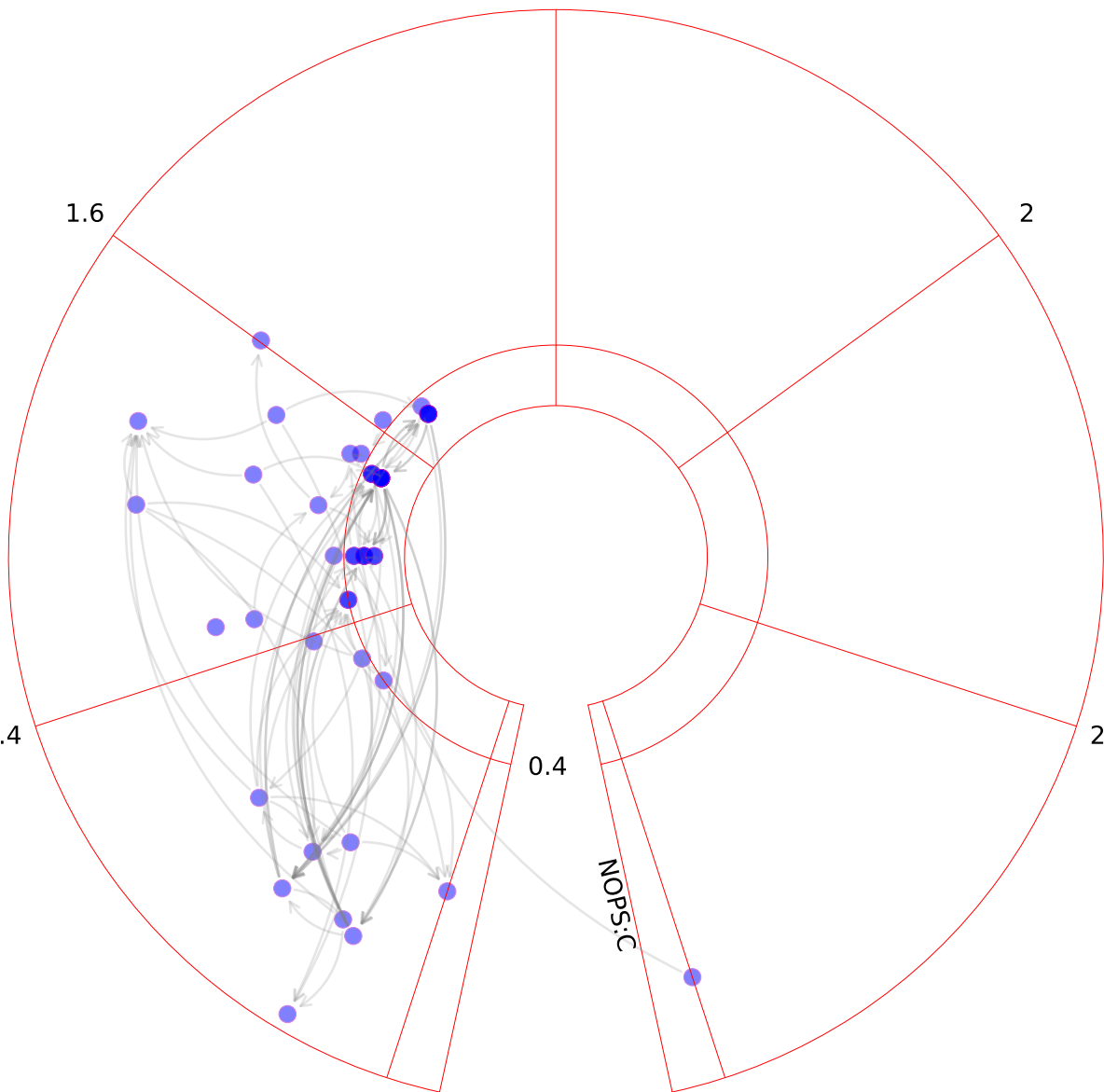

Supplement: Supplement 1 [file media-1.zip › Suppl_File_all_pathways/nolabel/Leukotriene metabolism.pdf]

# Lipoate metabolism

1.8

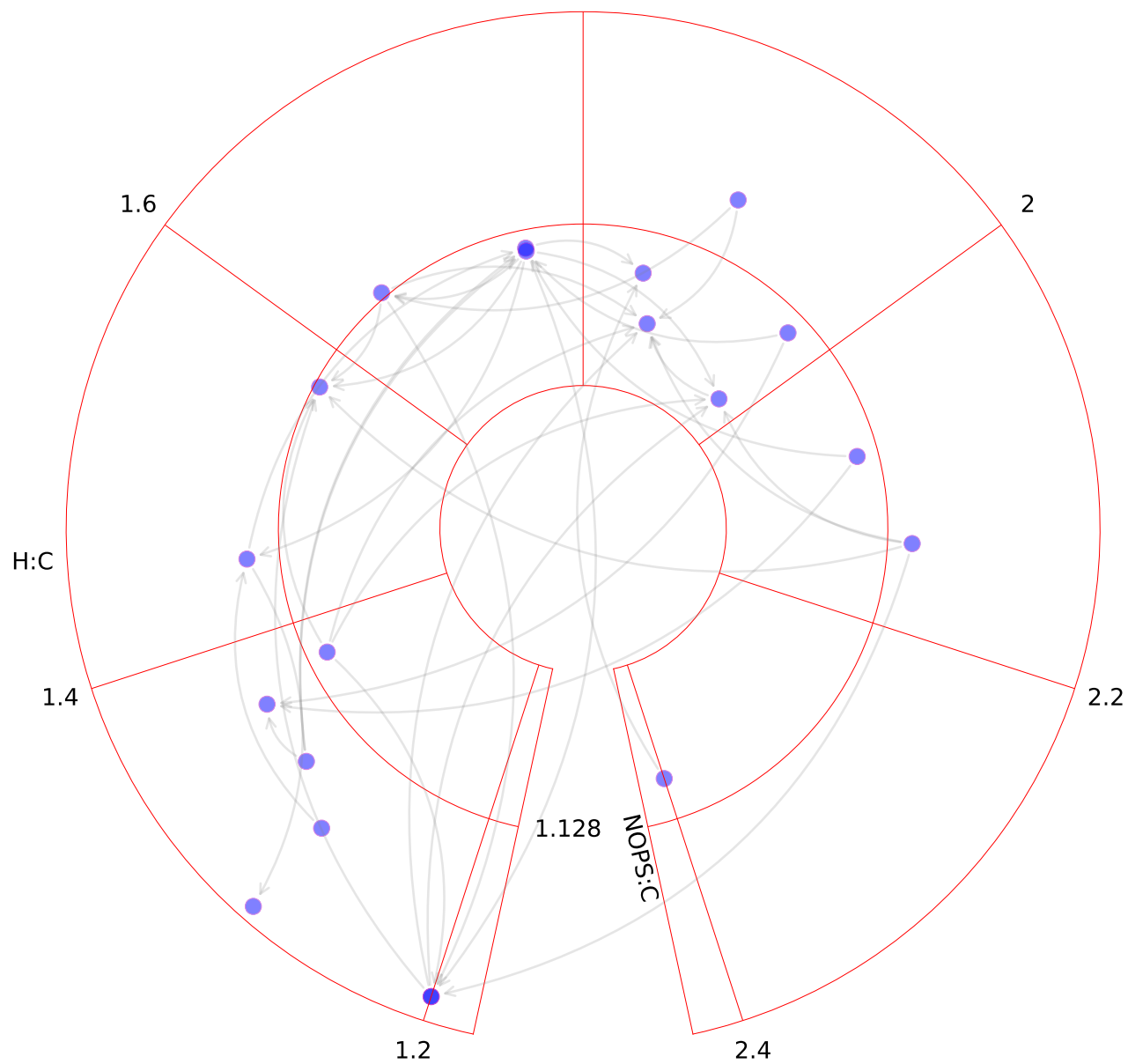

Supplement: Supplement 1 [file media-1.zip › Suppl_File_all_pathways/nolabel/Lipoate metabolism.pdf]

# Androgen and estrogen synthesis and metabolism

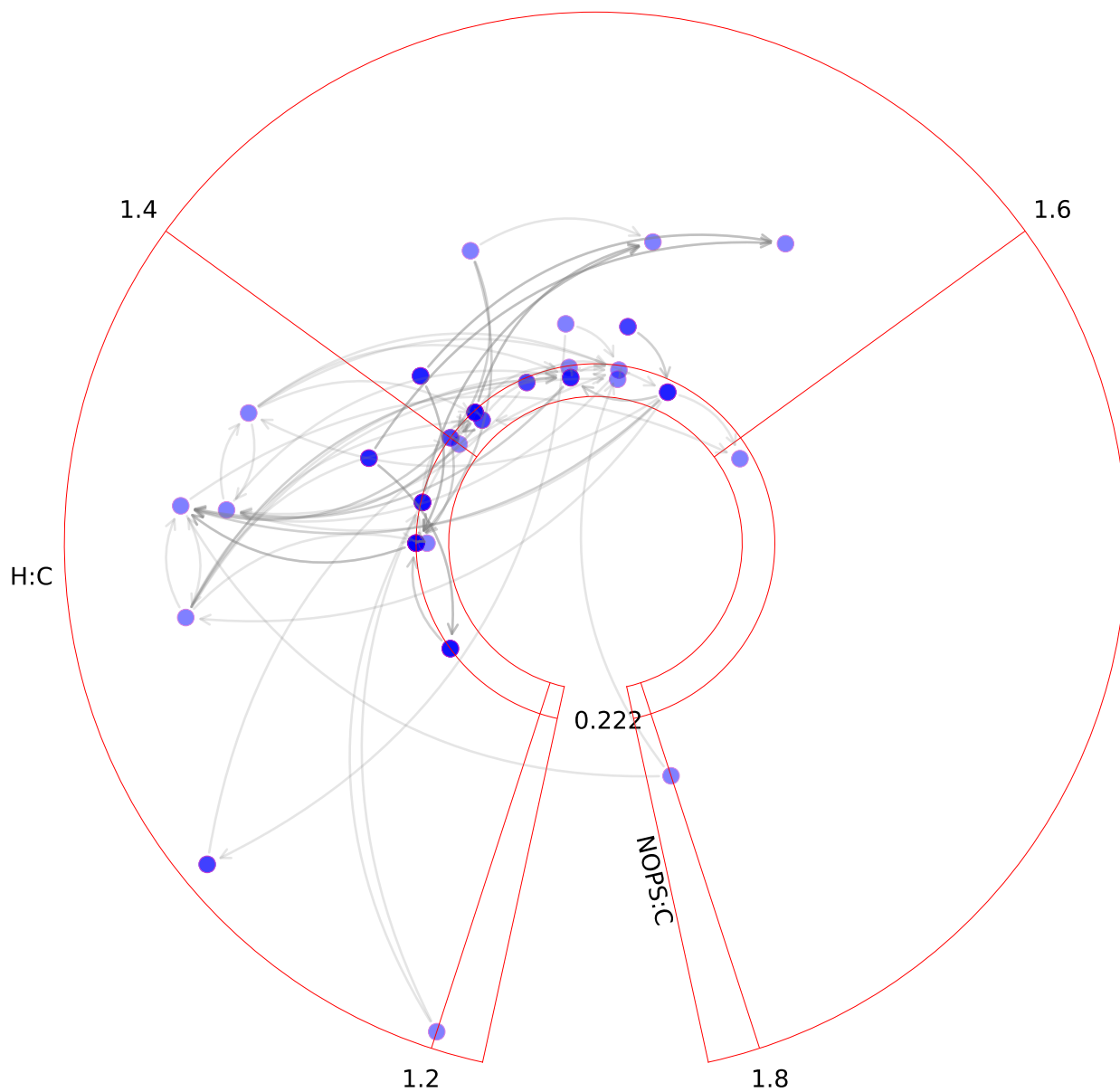

Supplement: Supplement 1 [file media-1.zip › Suppl_File_all_pathways/nolabel/Androgen and estrogen synthesis and metabolism.pdf]

Purine catabolism  
1.6

1.8

2

2.2

2.4

1.775

0.8

1.0

1.2

1.4

H:C

NOPS:C

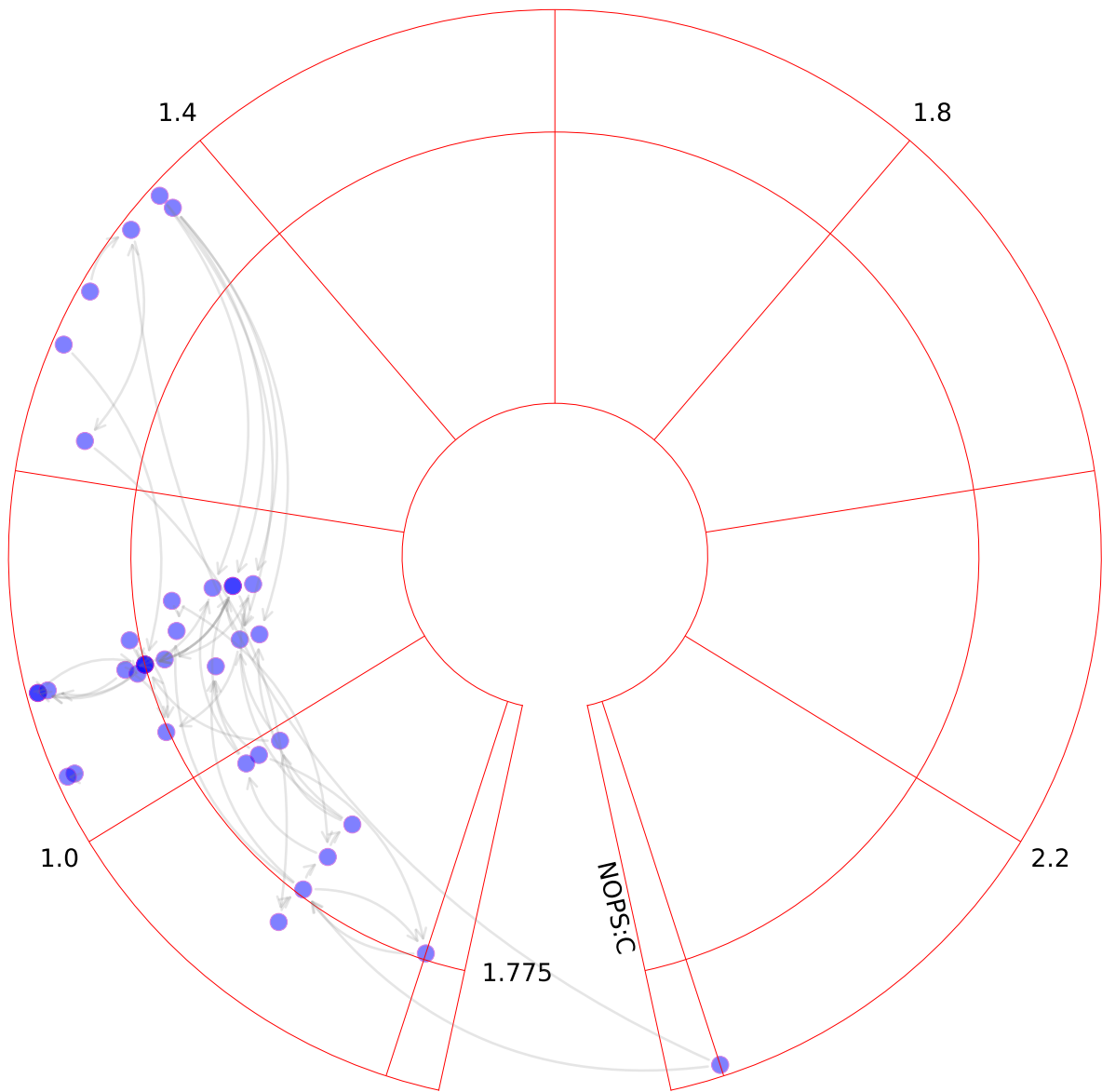

Supplement: Supplement 1 [file media-1.zip › Suppl_File_all_pathways/nolabel/Purine catabolism.pdf]

# Starch and sucrose metabolism

1.6

H:C

1.176

NOPS:C

1.4

1.8

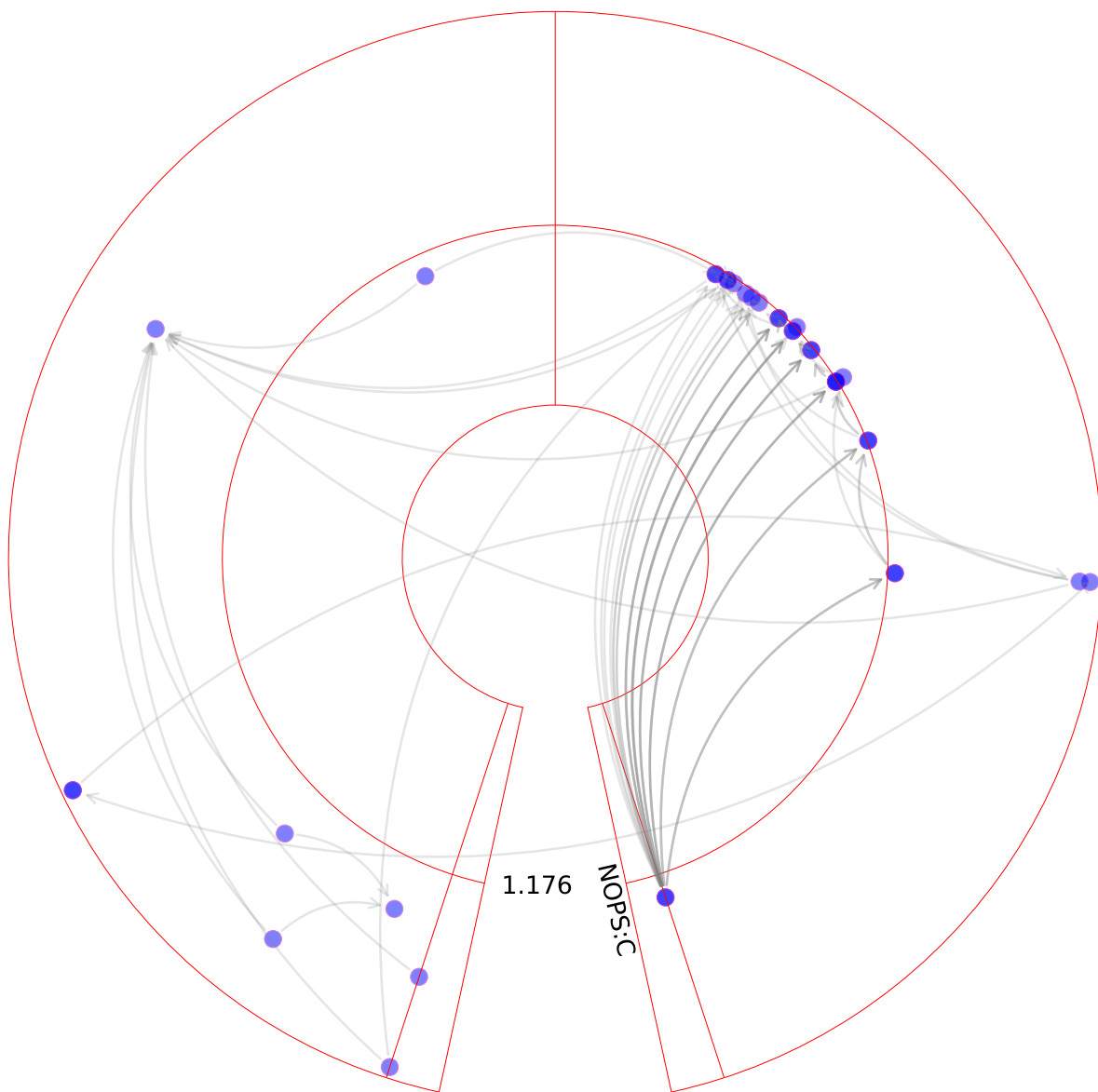

Supplement: Supplement 1 [file media-1.zip › Suppl_File_all_pathways/nolabel/Starch and sucrose metabolism.pdf]

# Valine, leucine, and isoleucine metabolism

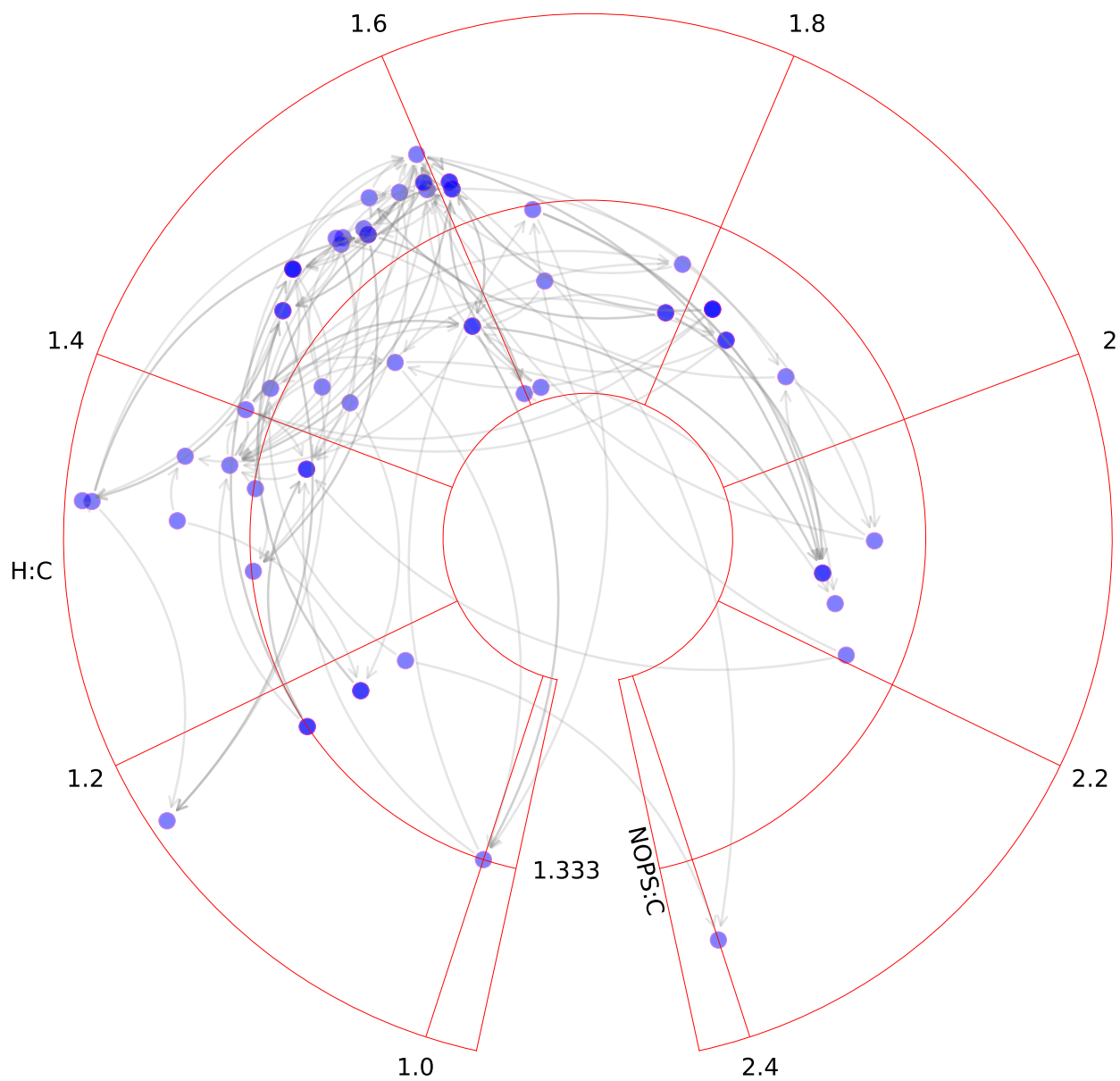

Supplement: Supplement 1 [file media-1.zip › Suppl_File_all_pathways/nolabel/Valine, leucine, and isoleucine metabolism.pdf]

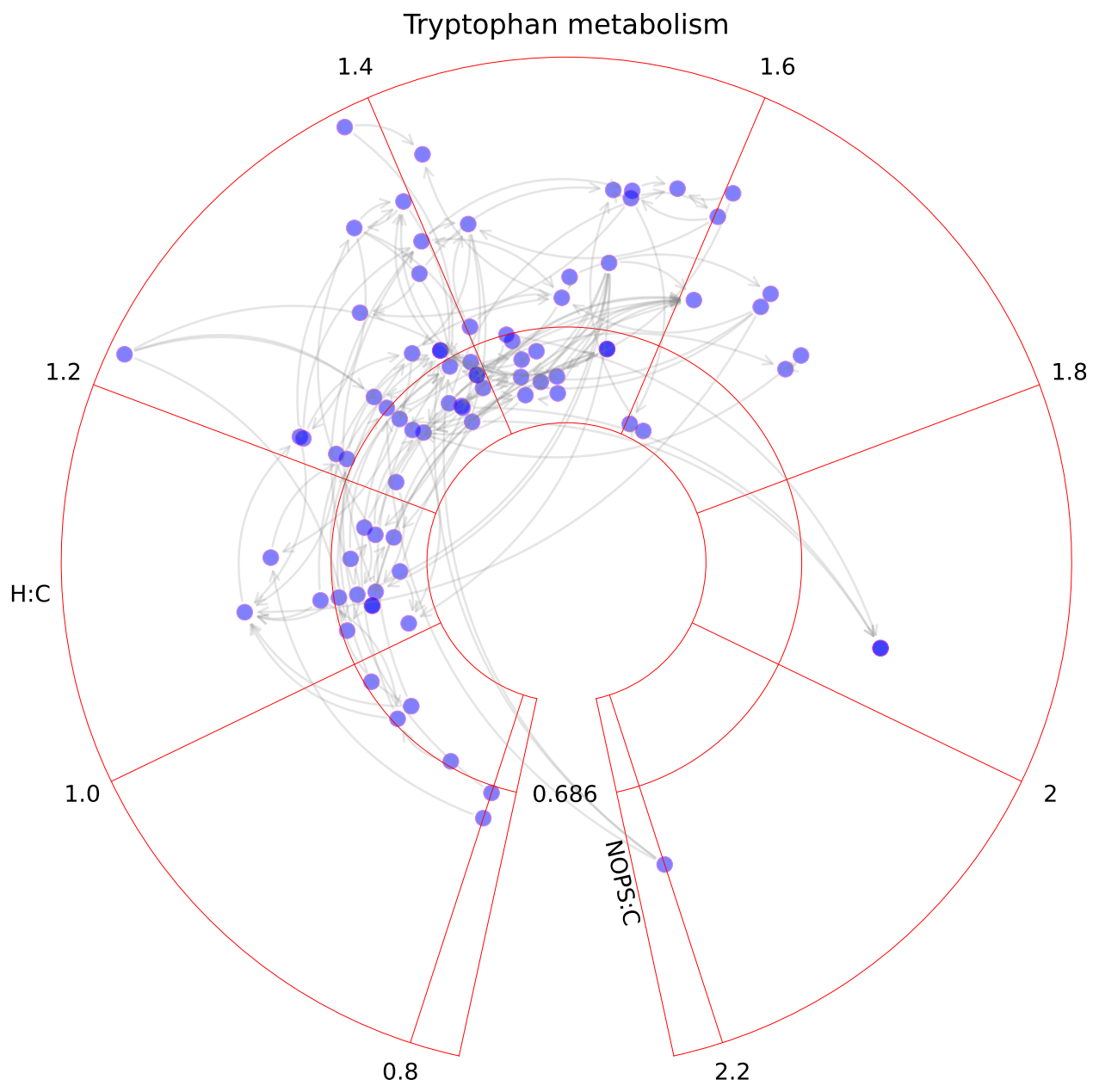

Supplement: Supplement 1 [file media-1.zip › Suppl_File_all_pathways/nolabel/Tryptophan metabolism.pdf]

# Glycosphingolipid metabolism

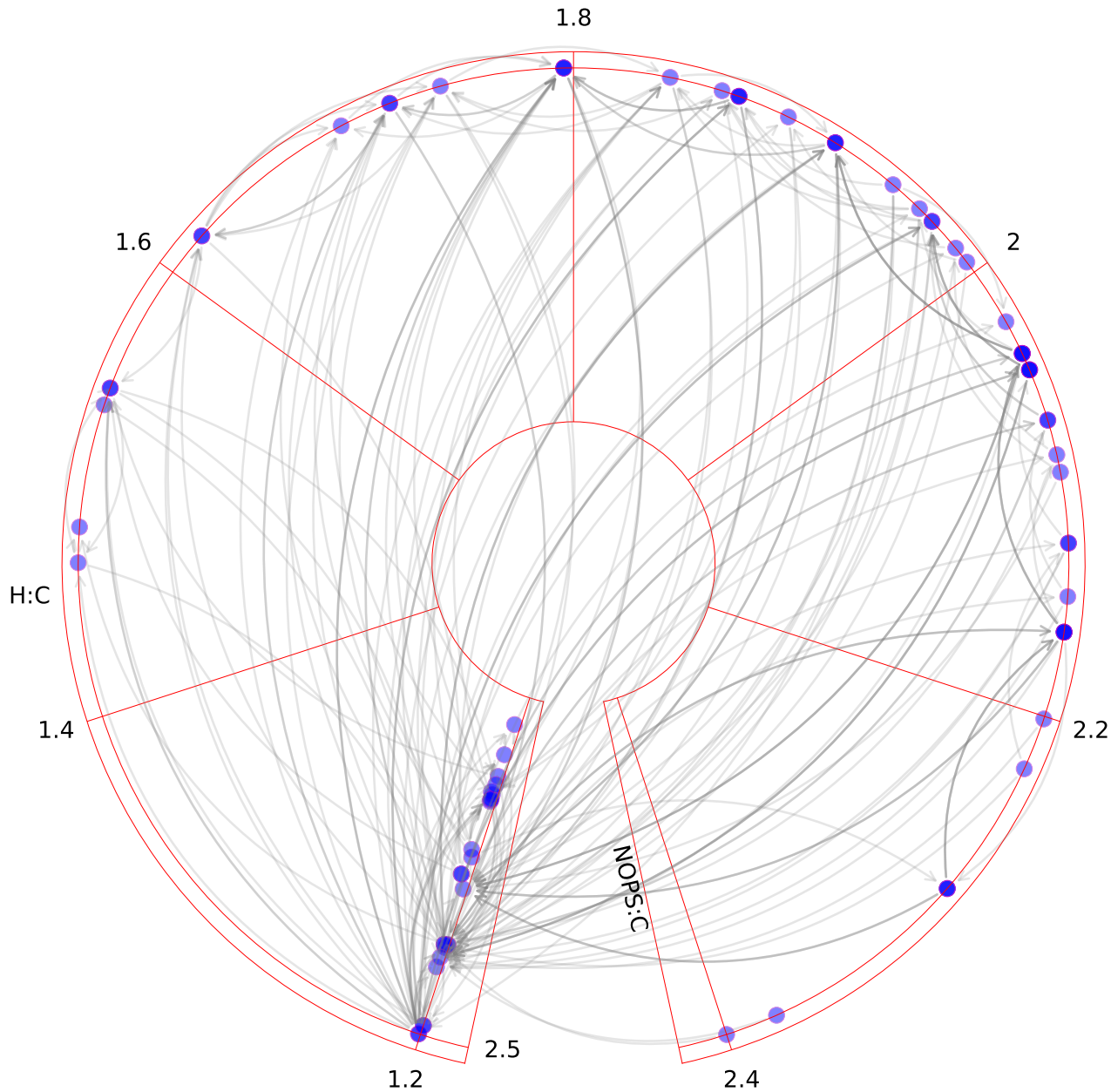

Supplement: Supplement 1 [file media-1.zip › Suppl_File_all_pathways/nolabel/Glycosphingolipid metabolism.pdf]

# Methionine and cysteine metabolism

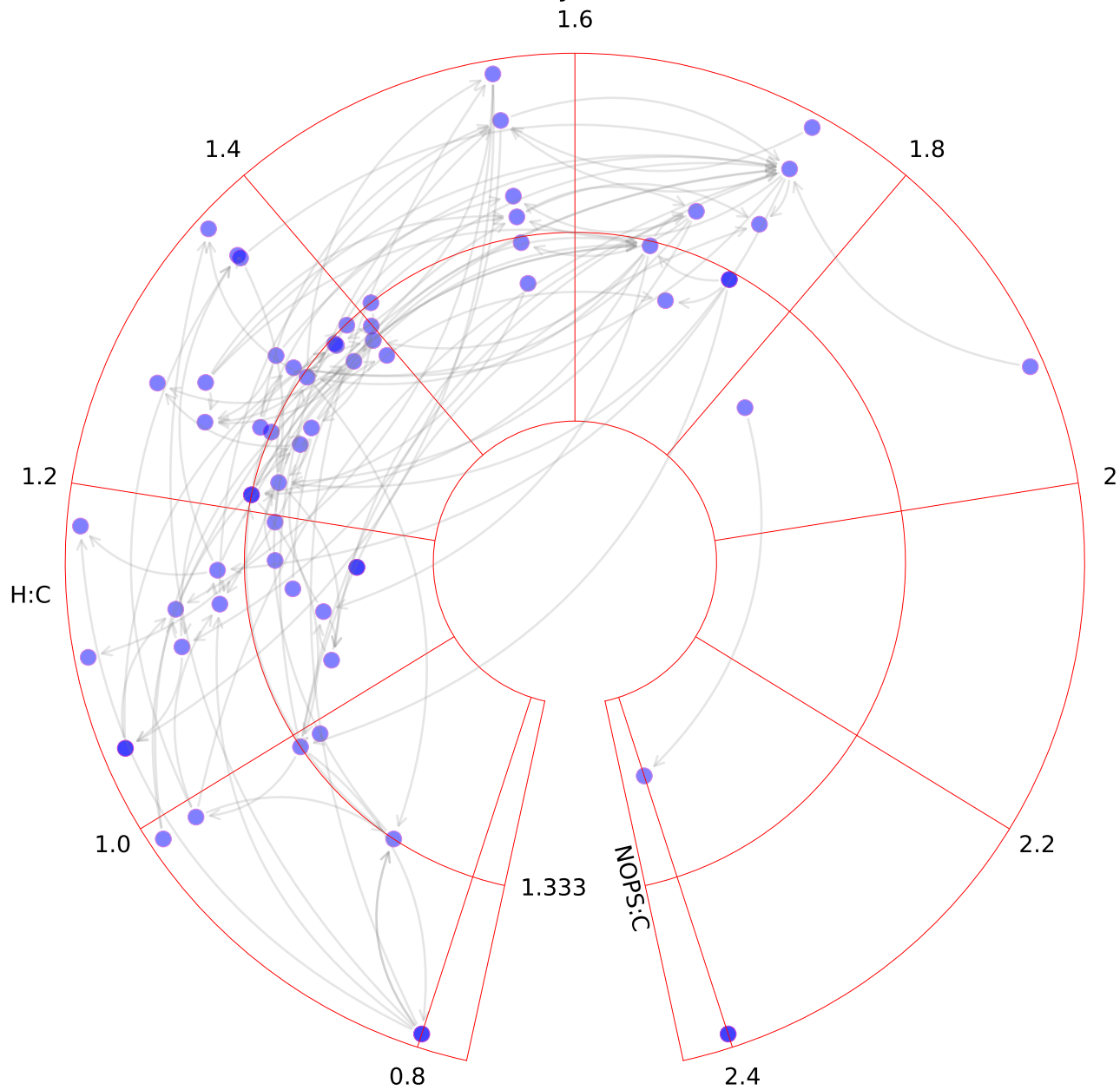

Supplement: Supplement 1 [file media-1.zip › Suppl_File_all_pathways/nolabel/Methionine and cysteine metabolism.pdf]

# Glycolysis/gluconeogenesis

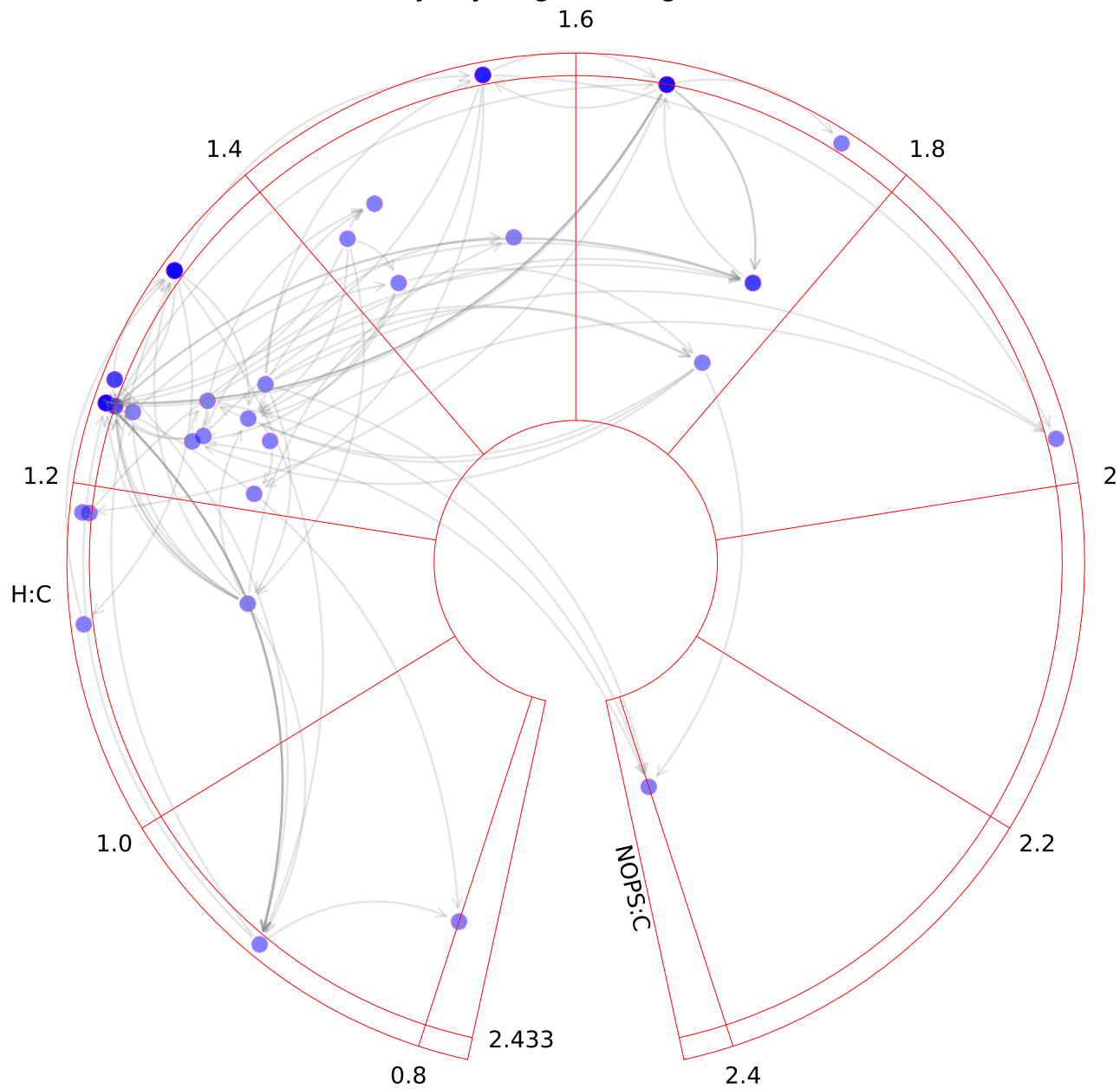

Supplement: Supplement 1 [file media-1.zip › Suppl_File_all_pathways/nolabel/Glycolysis_gluconeogenesis.pdf]

# Cholesterol metabolism

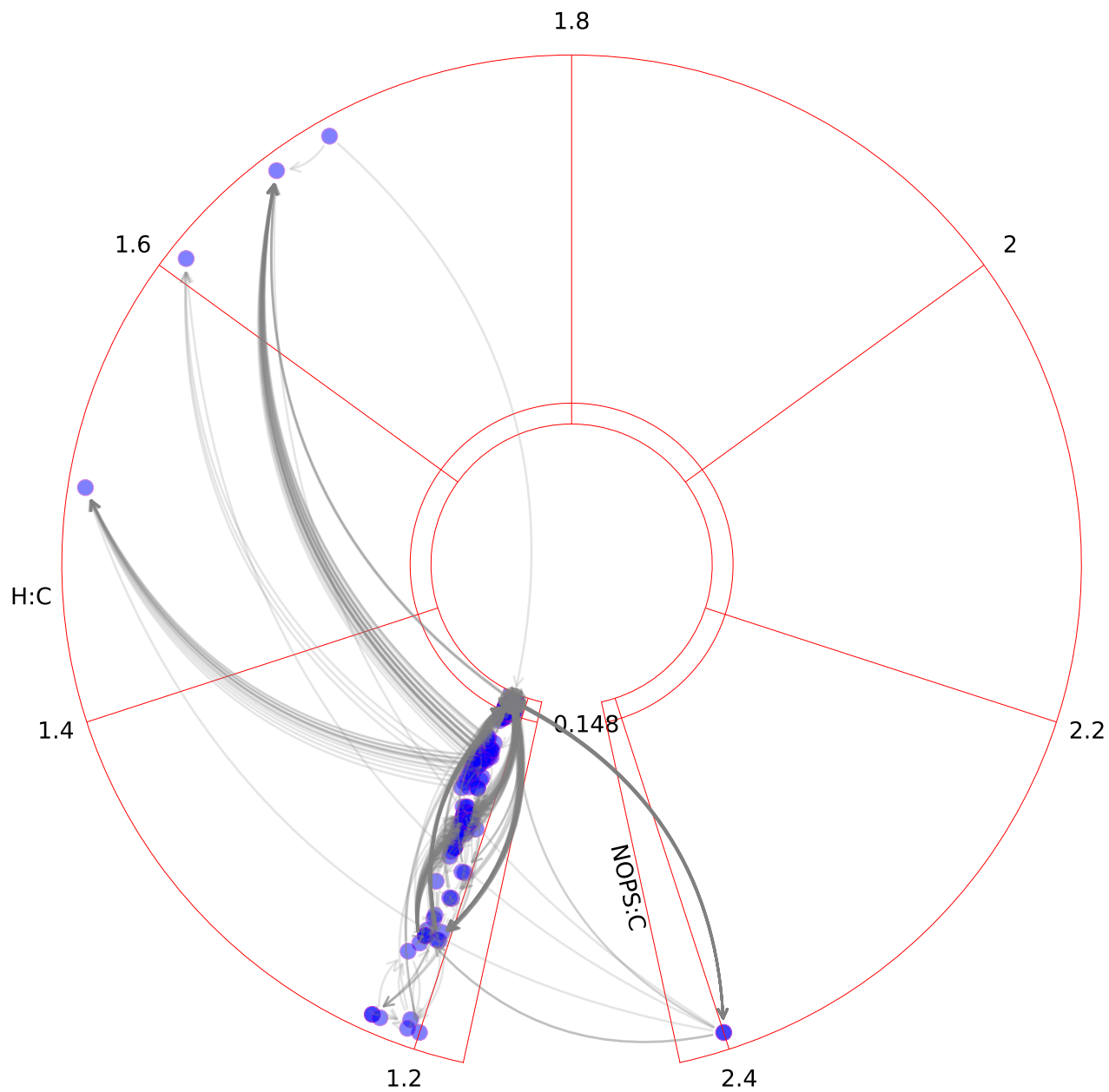

Supplement: Supplement 1 [file media-1.zip › Suppl_File_all_pathways/nolabel/Cholesterol metabolism.pdf]

# Thiamine metabolism

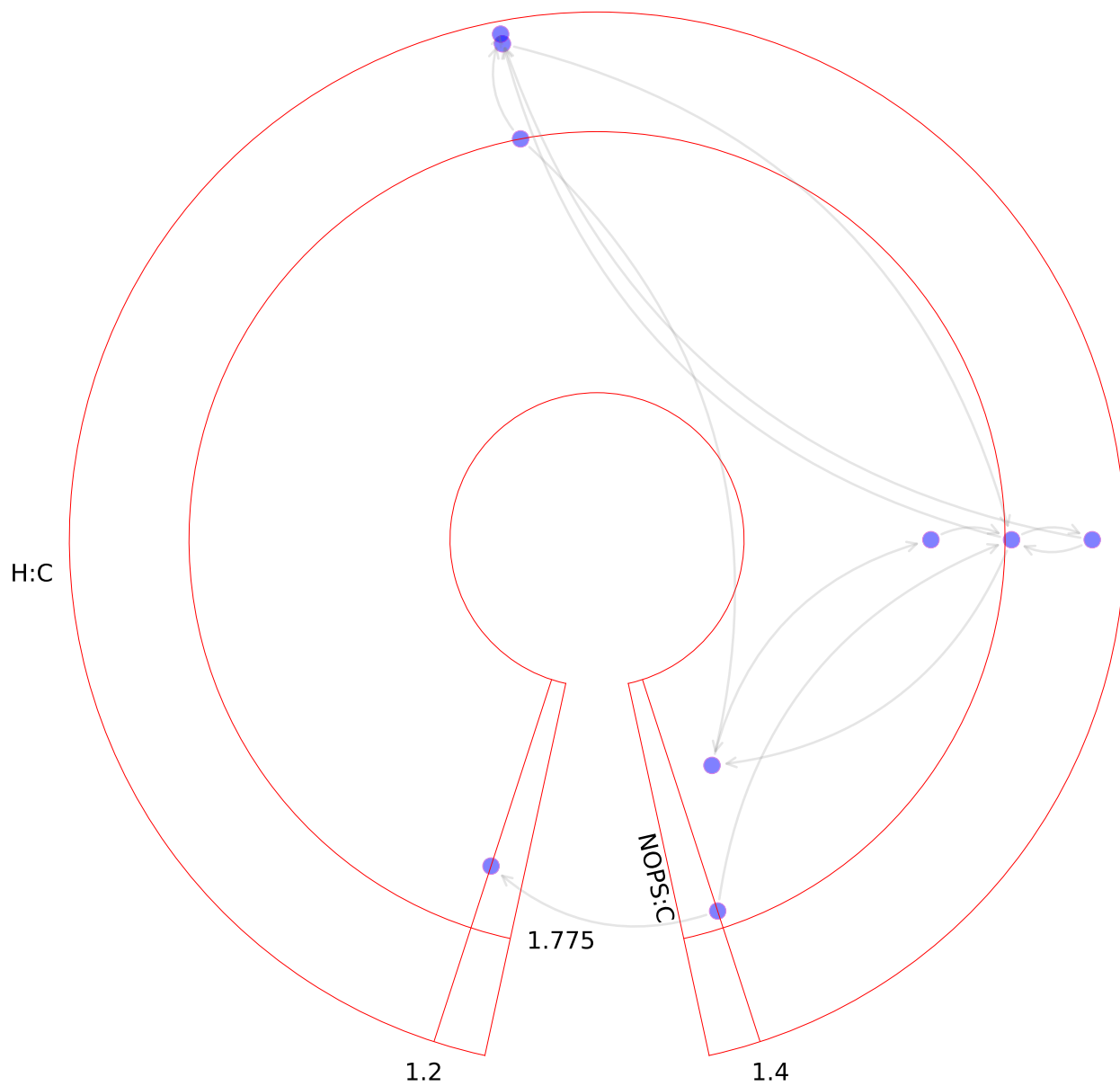

Supplement: Supplement 1 [file media-1.zip › Suppl_File_all_pathways/nolabel/Thiamine metabolism.pdf]

# Arginine and proline metabolism

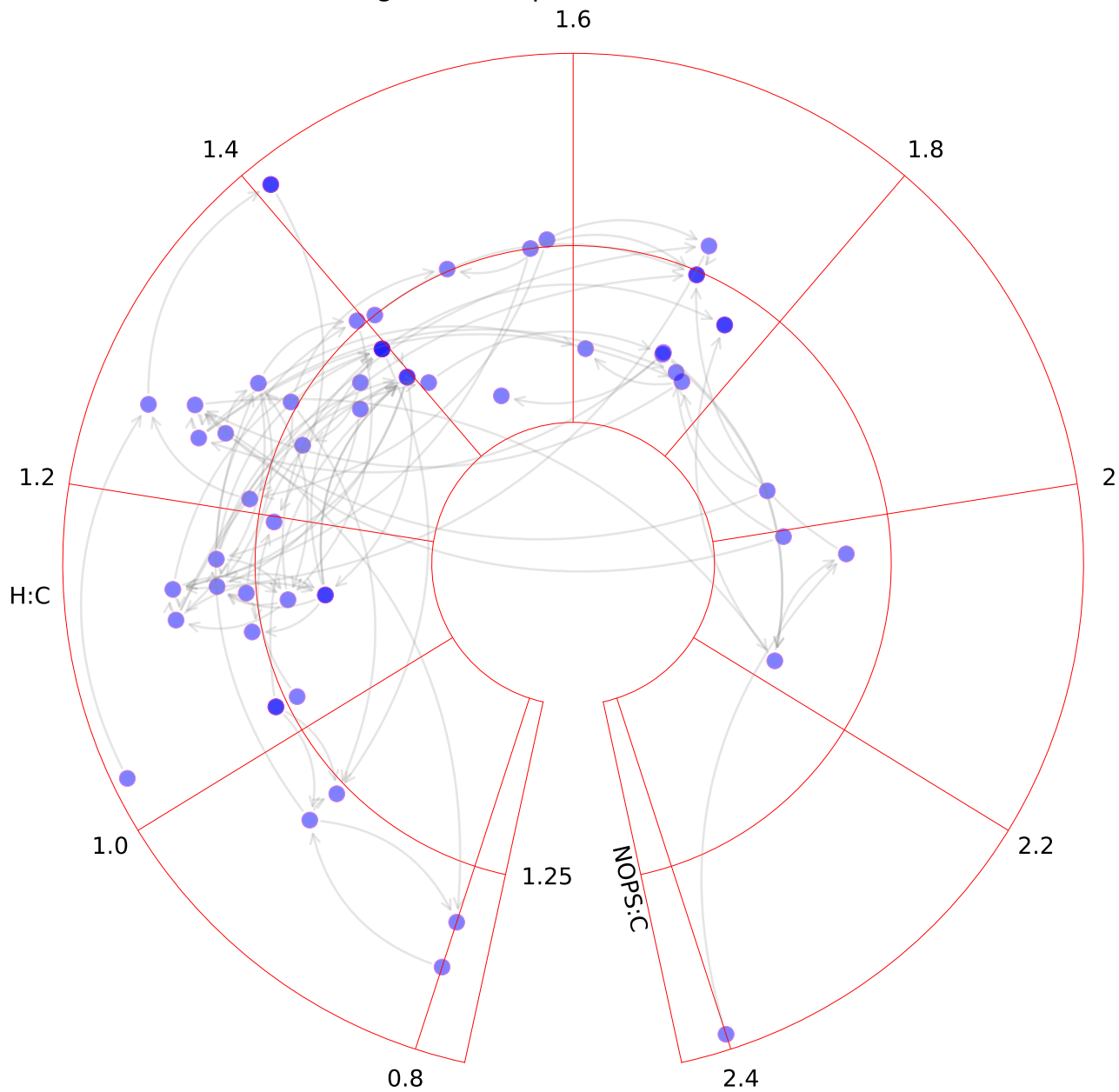

Supplement: Supplement 1 [file media-1.zip › Suppl_File_all_pathways/nolabel/Arginine and proline metabolism.pdf]

# Triacylglycerol synthesis

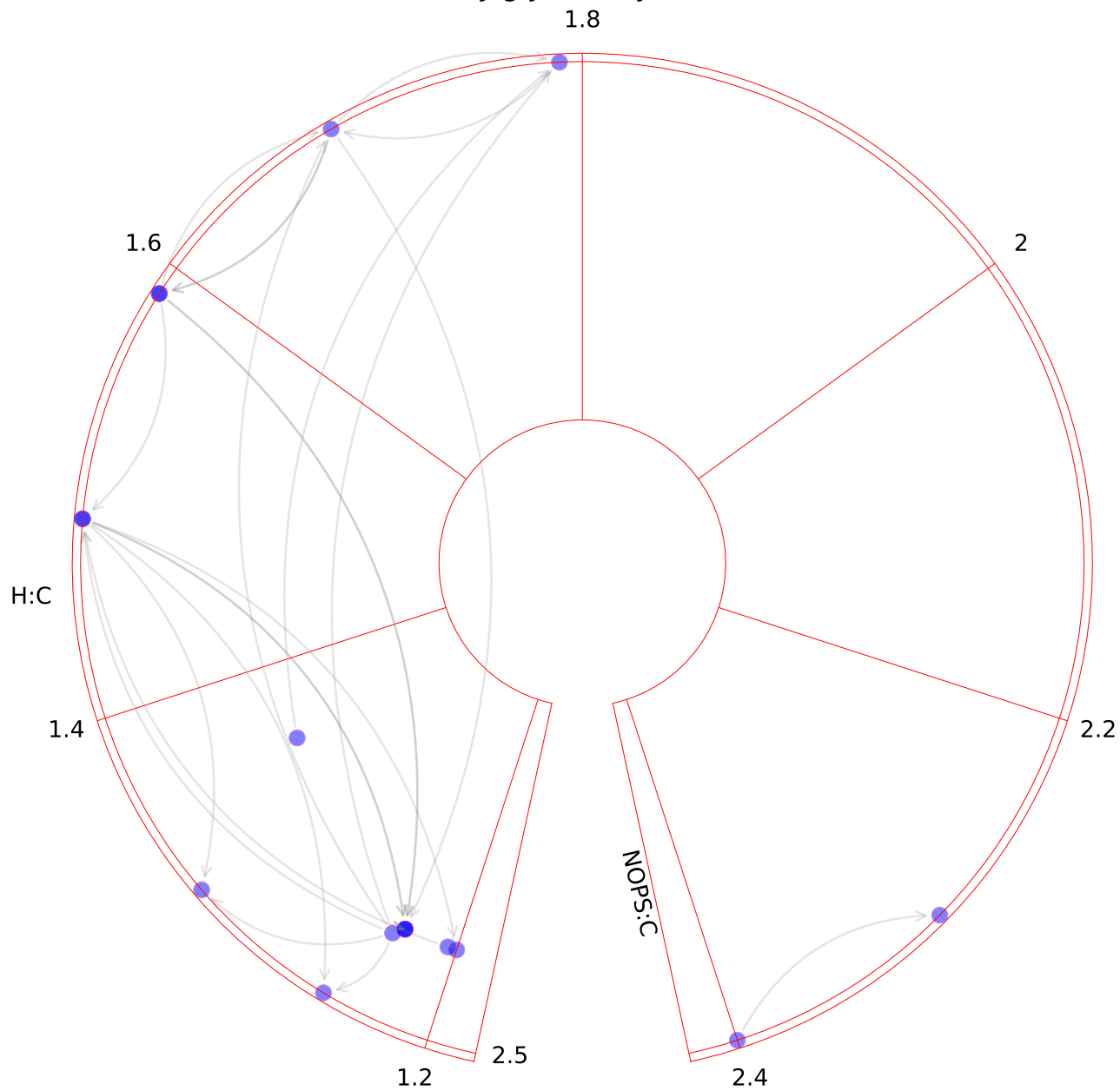

Supplement: Supplement 1 [file media-1.zip › Suppl_File_all_pathways/nolabel/Triacylglycerol synthesis.pdf]

# Glutathione metabolism

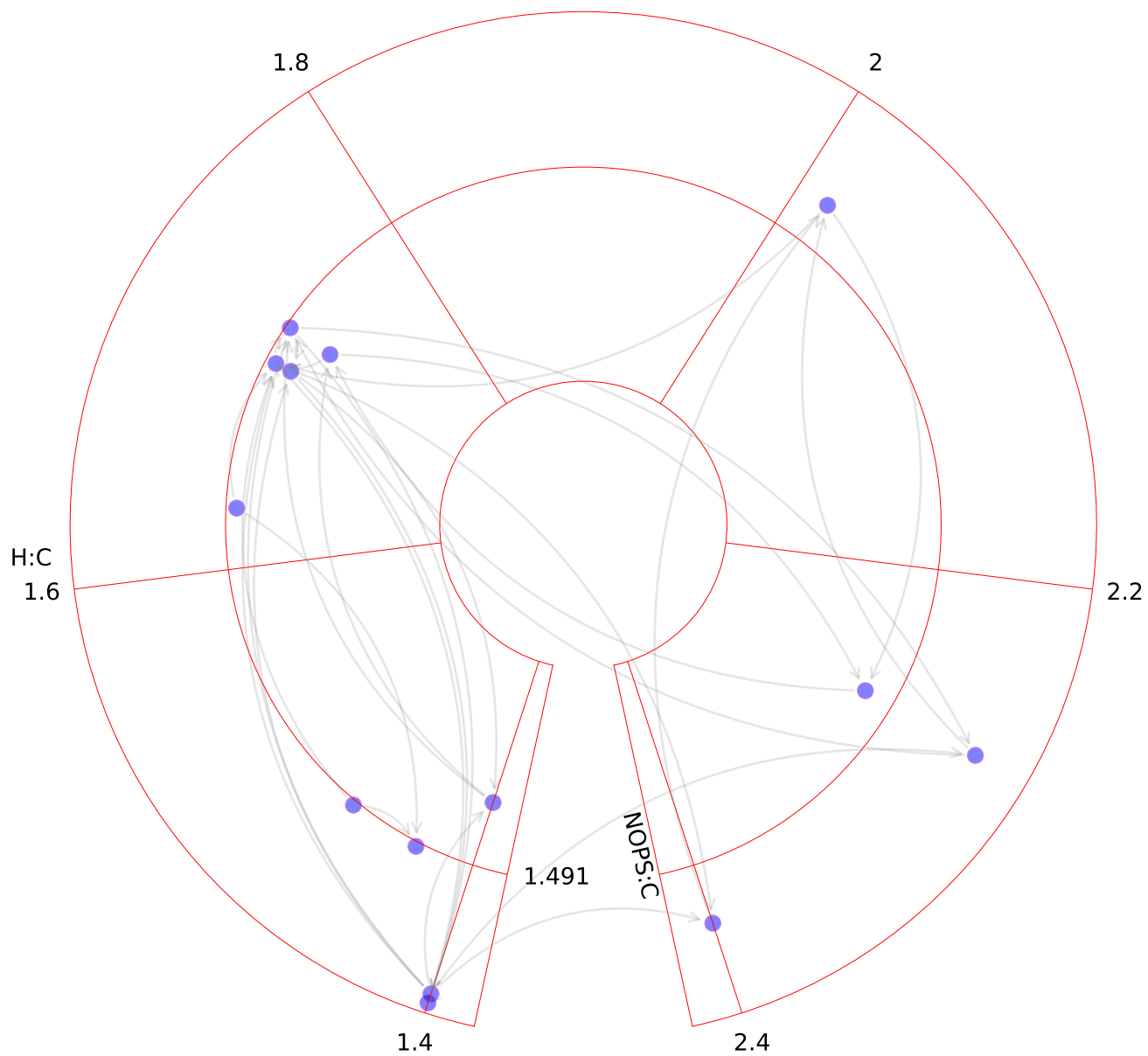

Supplement: Supplement 1 [file media-1.zip › Suppl_File_all_pathways/nolabel/Glutathione metabolism.pdf]

# Bile acid synthesis

1.8

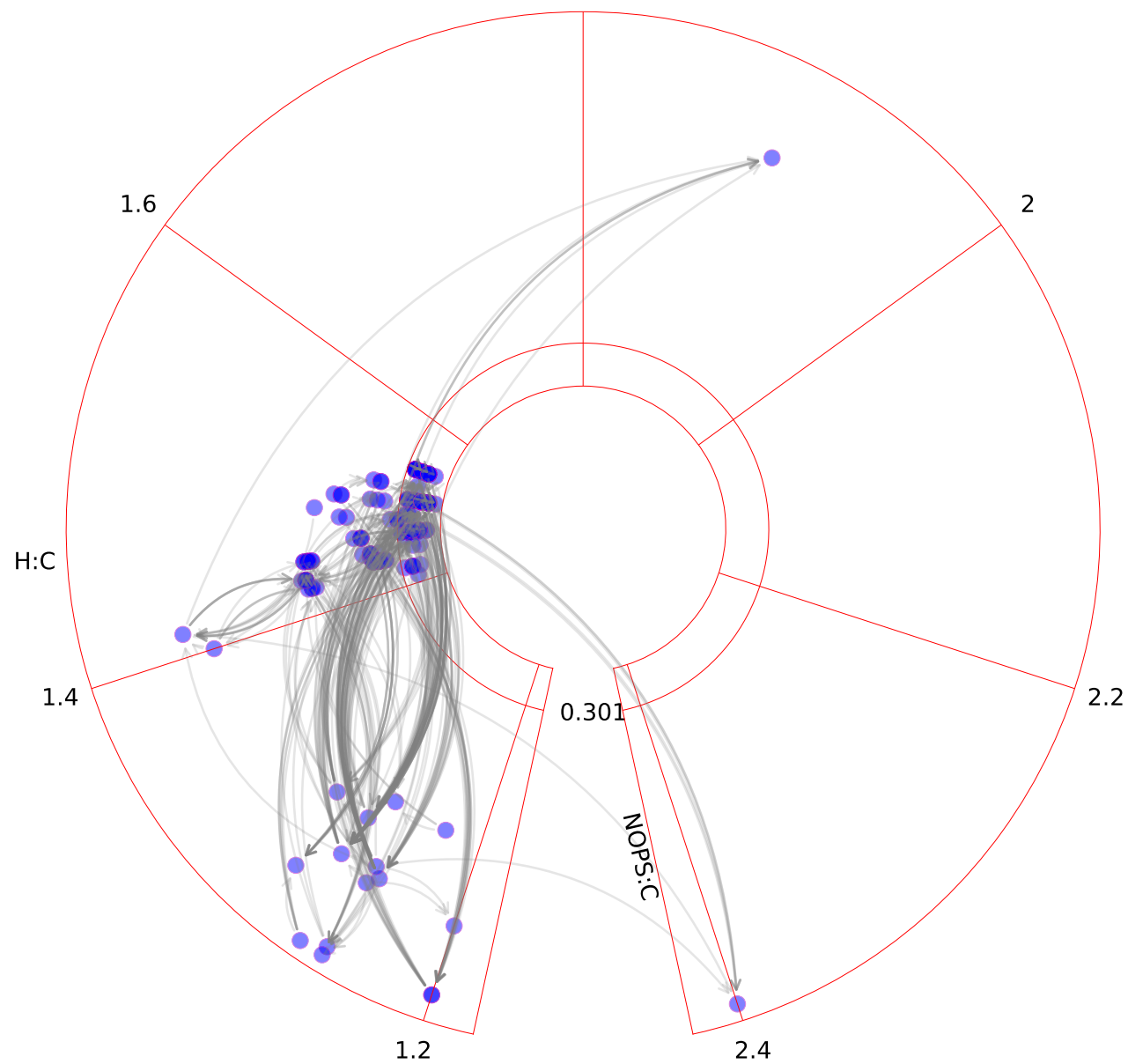

Supplement: Supplement 1 [file media-1.zip › Suppl_File_all_pathways/nolabel/Bile acid synthesis.pdf]

# Keratan sulfate degradation

1.6

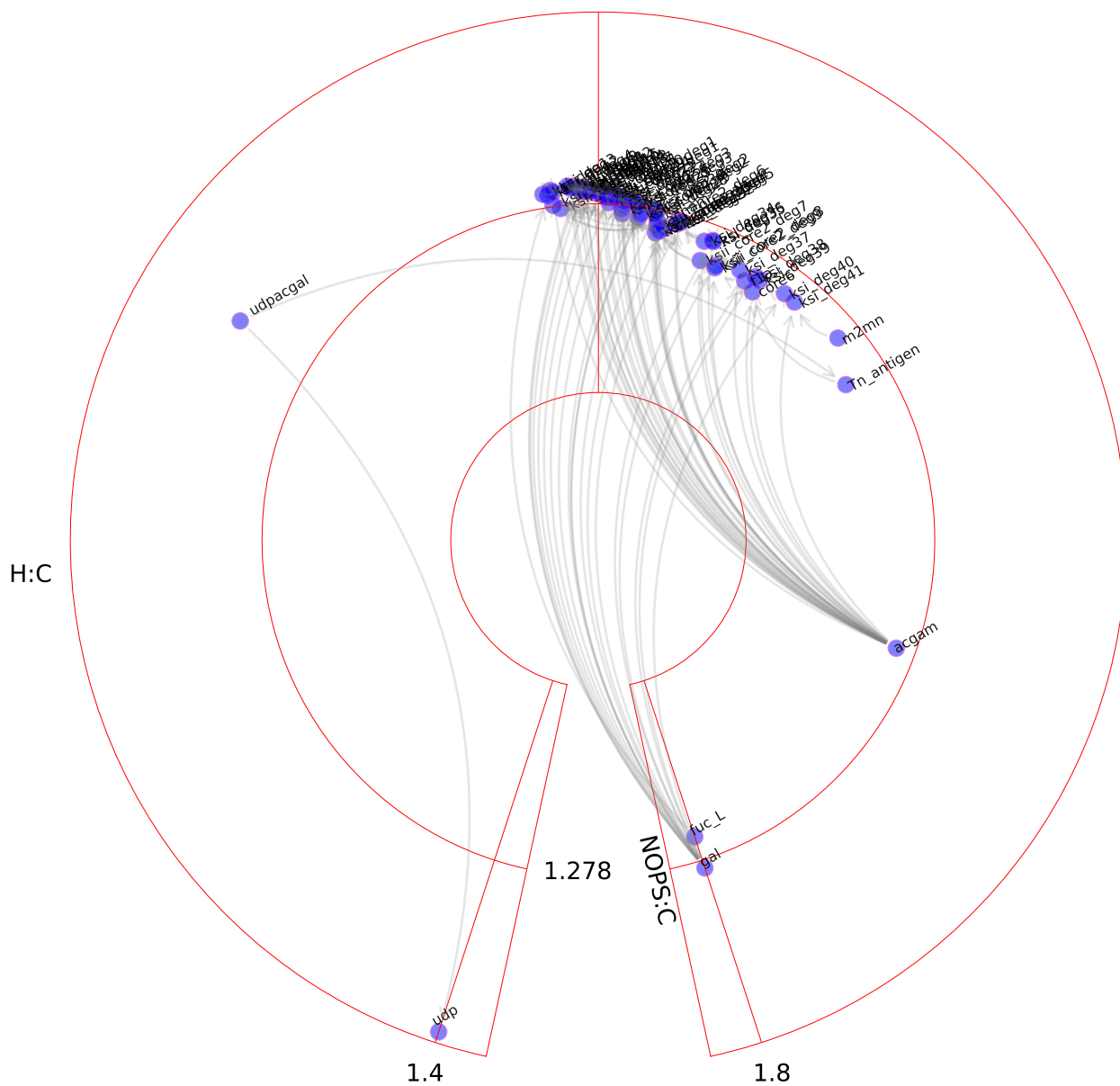

Supplement: Supplement 1 [file media-1.zip › Suppl_File_all_pathways/labeled/Keratan sulfate degradation.pdf]

# Nucleotide metabolism

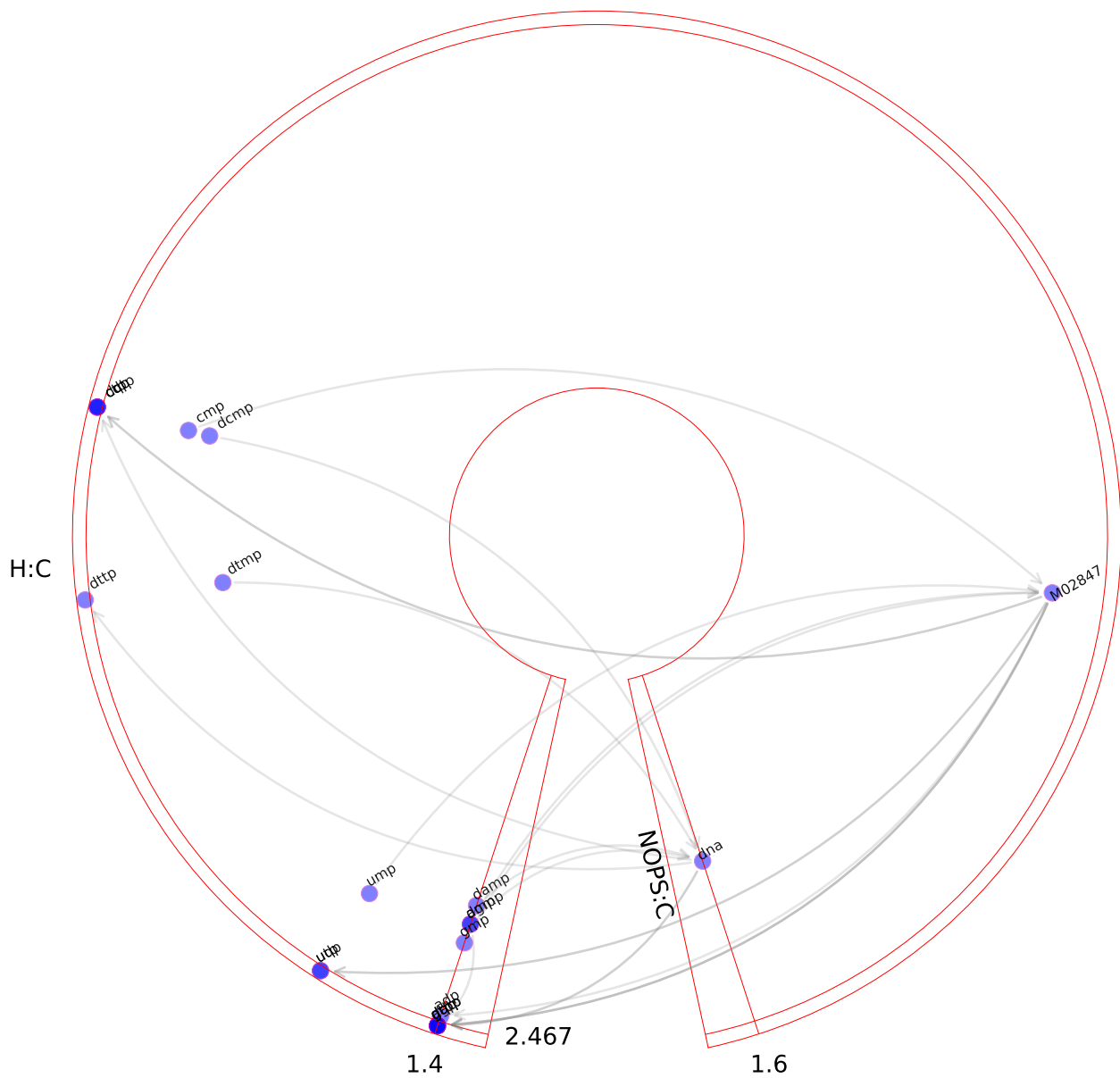

Supplement: Supplement 1 [file media-1.zip › Suppl_File_all_pathways/labeled/Nucleotide metabolism.pdf]
