# Supplementary material for: Circular van Krevelen diagram for visualizing metabolic pathways
Source: bioRxiv. 2025 Jun 3:2025.05.31.657198. Preprint. [Version 1] doi: 10.1101/2025.05.31.657198 (PMC12157561; doi:10.1101/2025.05.31.657198)

bioRxiv preprint doi: https://doi.org/10.1101/2025.05.31.657198; this version posted June 3, 2025. The copyright holder for this preprint (which was not certified by peer review) is the author/funder, who has granted bioRxiv a license to display the preprint in perpetuity. It is made available under aCC-BY 4.0 International license.

Supplemental Information

Supplemental File S1. Full list of pathway figures in Recon3 by default lcvk plot function.

Supplemental Figure S1: Scalable and zoomable pathway and network visualization. Optimization of this plot (**a**) by rescaling H:C range (**b**), by changing figure size (**c**), and by an optimization function to avoid overlap nodes (**d**).

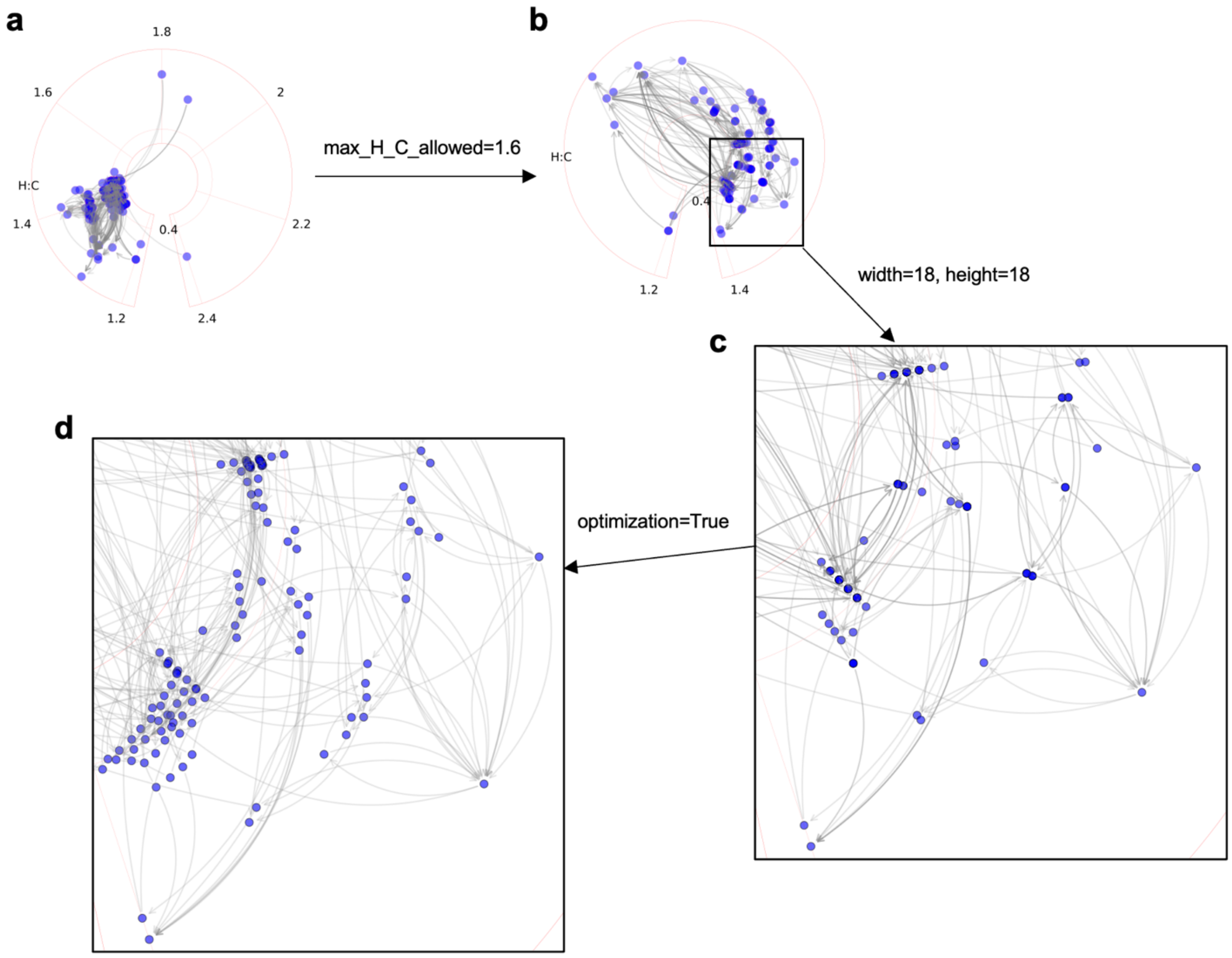

Supplement: 1 [file NIHPP2025.05.31.657198V1-supplement-1.pdf]
