# Supplementary material for: Circular van Krevelen diagram for visualizing metabolic pathways
Source: bioRxiv. 2025 Jun 3:2025.05.31.657198. Preprint. [Version 1] doi: 10.1101/2025.05.31.657198 (PMC12157561; doi:10.1101/2025.05.31.657198)
Supplement: Supplement 1 [file Thiamine metabolism.pdf › Suppl_File_all_pathways/labeled/Thiamine metabolism.pdf]

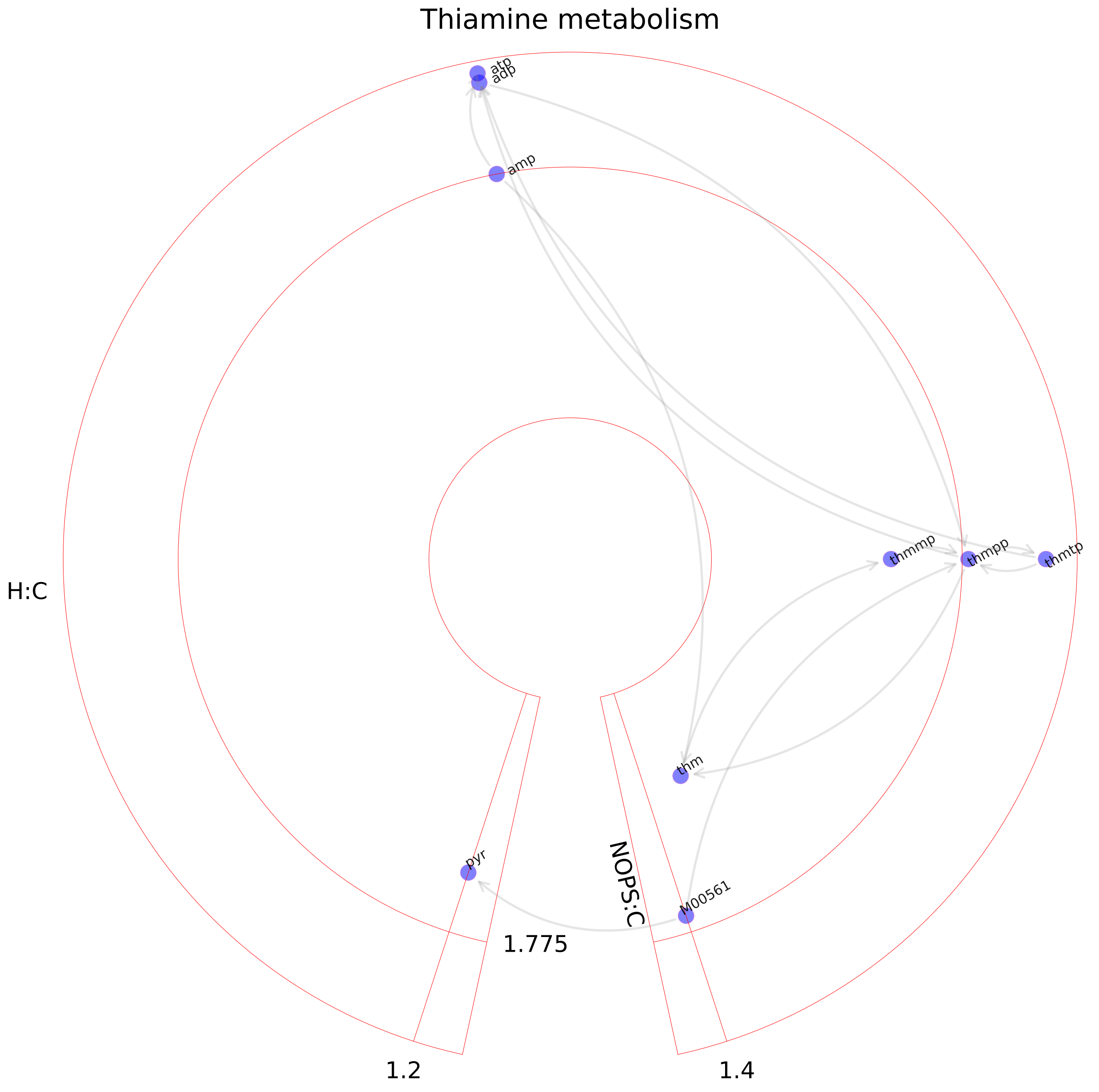

Thiamine metabolism
atp
adp
amp
thmmp
thmpp
thmtp
thm
pyr
M00561
H:C
NOPS:C
1.775
1.2
1.4
